# Grounding annotations in published literature with an emphasis on the functional roles used in metabolic models

**DOI:** 10.1007/s13205-011-0039-z

**Published:** 2011-12-14

**Authors:** Erik Binter, Scott Binter, Terry Disz, Elizabeth Kalmanek, Alexander Powers, Gordon D. Pusch, Julie Turgeon

**Affiliations:** 1 Mathematics and Computer Science Division, Argonne National Laboratory, 9700 S Cass Avenue, Argonne, IL 60439 USA; 2 Fellowship for Interpretation of Genomes, Burr Ridge, IL 60527 USA

**Keywords:** Genome annotations, Protein function, Evidence of function

## Abstract

Accurate genome annotations in databases are a critical resource available to the scientific community for analysis and research. Inaccurate and inconsistent annotations exist as a result of errors generated from mass automated annotation, and currently act as a barrier to the application of bioinformatics. The purpose of this effort was to improve the SEED by improving the connection of functional roles to literature references. Direct literature references (DLits), found through searches of PubMed and other online databases such as SwissProt, were attached to protein sequences within the PubSEED to provide literature support for the roughly 2,500 distinct functional roles used to construct metabolic models within the Model SEED. Only DLits in which a researcher asserted the function of a protein were attached to sequences. Starting from a list of 1,072 functional roles that did not previously have DLit support, we were able to connect sequences to literature for 655 functional roles, at least 484 of which were in the original list of unsupported roles. When added to the existing set of sequences having DLits, the resulting set of DLit-sequence pairs (the foundation set) now connects approximately 4,300 DLits to approximately 5,600 distinct protein sequences obtained from approximately 16,000 genes (some of these genes have identical protein sequences). From the foundation set, we construct projection sets such that each set contains one member of the foundation set and projections of its functional role onto similar genes. The projection sets revealed 120 inconsistent annotations within the SEED. Two types of inconsistencies were corrected through manual annotation in the PubSEED: instances in which two identical protein sequences had been annotated with different functions, and instances when projected functions contradicted previous annotations. 26,785 changes to gene function assignment, 219 of which were to previously uncharacterized proteins, resulted in a more consistent and accurate set of input data from which to construct revised metabolic models within the Model SEED.

**Electronic supplementary material:**

The online version of this article (doi:10.1007/s13205-011-0039-z) contains supplementary material, which is available to authorized users.

## Introduction

The SEED database (Overbeek et al. 2004; Disz et al. 2010) was started in 2003 by the Fellowship for Interpretation of Genomes (FIG) (2011) as a collection of tools and resources that mainly serves as an environment for comparative gene analysis. Our project was a part of the ongoing collaborative effort to expand the SEED and to improve the accuracy of functions projected onto genes of different organisms within the database. Two systems built using SEED technology (Overbeek et al. 2004; Disz et al. 2010), the Model SEED (Henry et al. 2010) and the PubSEED (http://pubseed.theseed.org/seedviewer.cgi), were fundamental in this project. The PubSEED is a publicly accessible genomic database and subsystem-based annotation framework (Overbeek et al. 2005) that provides information about genes and their annotated functions for nearly 4,000 published genomes. The Model SEED is a web-based resource for high-throughput generation, optimization and analysis of genome-scale metabolic models; it currently allows public access to metabolic models for over 200 published genomes.

Construction of self-consistent and accurate metabolic models requires accurate annotations of the enzymatic reactions (DeJongh et al. 2007) and metabolic pathways (Schuster et al. 2000) present in a genome. A fundamental goal of our project was to provide evidence for the functional roles carried out by distinct protein sequences used in the Model SEED. We searched for literature evidence, referred to as Direct Literature References (DLits), that connected specific function to a protein sequence found in the PubSEED. We also strove to correct inconsistent functional annotations in the SEED, due either to inaccurate function assignments or lack of consistency in terms used.

The protein sequences in the PubSEED that have DLits attached to them constitute the core group known as the foundation set. To expand the foundation set, we searched through other databases, most notably the PubMed database (Roberts 2001), to find DLits that provided direct evidence for the function of specific genes and protein sequences in the PubSEED. Manual curation of such publications ensured that only the most relevant works were ultimately attached to the sequences in the SEED.

## Methods

### Expanding the foundation set

We began by generating a list of functional roles found in the Model SEED that were not grounded in literature with a DLit. Databases such as the National Institute of Health’s PubMed, the Kyoto Encyclopedia of Genes and Genomes (KEGG) (Kanehisa 2002), and the University of London’s E.C. Number Database (Recommendations of the Nomenclature Committee of the International Union of Biochemistry and Molecular Biology on the Nomenclature and Classification of Enzymes by the Reactions they Catalyse, http://www.chem.qmul.ac.uk/iubmb/enzyme) were searched to find articles supporting the role assignments. To expedite the process, another list was generated using information from the SwissProt database (Bairoch and Apweiler 2000). This list contained the functional roles from our original list and the set of PubMed articles that SwissProt had assigned to the corresponding sequences in their database. We then reviewed these references and attached those that met our criteria, attaching only the DLits that asserted an explicit connection between a specific sequence or gene and its function. Complete genome papers were generally excluded because they lacked the necessary specificity.

### Assigning new functions to genes

Our additions to the foundation set also enabled us to assign functions to genes that were not previously annotated with a functional role in the SEED. New annotations were assigned to these previously unannotated genes by projecting a functional role from a member of the foundation set onto all genes that met our similarity criteria, as described below. The resulting groups of genes and sequences with identical function that are generated through this process are called projection sets. Each projection set contains one member of the foundation set, and a set of projections that could be made from it using the criteria described below.

### Criteria for making projections

We are seeking to make reliable projections of function from genes in one genome to corresponding genes in another. We impose two primary constraints on such projections: similarity of sequence, and similarity of surrounding neighborhoods on each genome.

**Fig. 1:**
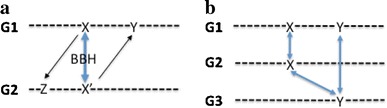
BBHs. **a***Light arrows* denote weak similarity, **b** illustration of the need for a “false positive BBH” filter

Our sequence similarity criterion is that the region of match between the compared genes must cover at least 80% of the total length of each gene, and that the similarity must be a clear bidirectional best hit. The minimum 80% coverage criterion eliminates spurious hits against single common domains, as well as hits against fused genes. A Bidirectional Best Hit (BBH) signifies that the candidate gene is more similar to the foundation set gene than to any other gene in the foundation set genome, and that the foundation set gene is more similar to the candidate gene than to any other gene in the candidate genome. Figure 1a illustrates the BBH relationship; the heavy double-headed arrow denotes the BBH, while the lighter single-headed arrows denote weaker similarities to other genes. A BBH is said to be a clear BBH if the difference between the percent identity of the BBH and the next highest percent identity between either gene and any gene in the other genome is ≥5%. (Requiring at least a 5% difference in percent identities is sufficient to rule out gene duplications from recently inserted mobile elements or prophages, which often display identity scores close to 100%.)

A filter is then applied to the collection of clear BBHs to remove false-positives due to gene duplication (Ohno 1970). These false-positives result when some genome has two similar genes that perform slightly different functions. If gene X is passed on to a second genome whereas Y is not, and gene Y is passed on to a third genome whereas gene X is not, the two genes may form a clear BBH between the second and third genomes. The genes, however, are performing different functions, so no projection should be made in this case (see Fig. 1b).

**Fig. 2:**
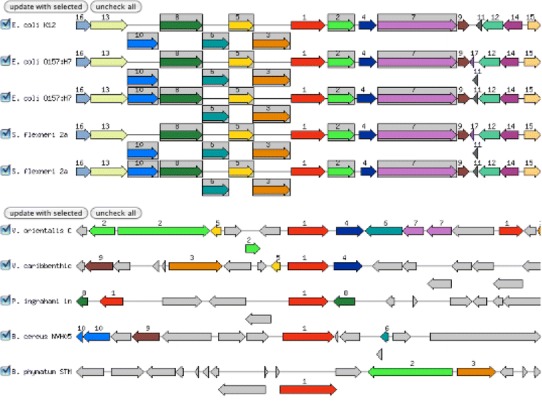
Gene context is conserved in the *upper* portion of this illustration, but not in the *lower* portion

Empirically, it is observed that genes that work together or carry out related functions are often found within close proximity to each other on the chromosome, and that this proximity is strongly conserved (Fig. 2)—a phenomenon known as “chromosomal clustering” (Overbeek et al. 1999a, b; Dandekar et al. 1998). Hence, once the “false-positive” BBHs are removed, we compute for each BBH a projection score (Overbeek and Xia 2011) that takes into account both the number of conserved neighbors and the percent identity of the BLAST (Altschul et al. 1997) computed similarity as follows:

1. Let X be a clear BBH of X′ (Fig. 1a),
2. Let *N* be the number of pairs of clear BBHs (up to a maximum of 10) in the chromosomal context region surrounding X and X′, and
3. Let *I* be the percent identity between sequences X and X′;

then we compute the score of the potential projection as

1 $$ {\text{Score}} = 0.8 \times \frac{{\log \left( {N + 1.5} \right)}}{{\log \left( {11.5} \right)}} + 0.2 \times \left( \frac{I}{100} \right)^{1.5} . $$

The weights and parameters in the above scoring function have been chosen, somewhat arbitrarily, to cause the scoring function to yield the following desirable properties:

1. It produces a value between 0 and 1 that reflects the weighted evidence supporting the potential projection.
2. It implements an assumed “law of diminishing returns” for additional context evidence by taking the logarithm.
3. It emphasizing higher percent identities by raising the fraction of identity to a positive power.
4. It places a heavier weight on chromosomal context than on percent identity, because conservation of chromosomal context provides very strong evidence for asserting functional similarity (see Fig. 2).

Potential projections scoring ≥0.5 are kept; again, our choice of threshold is somewhat arbitrary, but was guided by the empirical observation that a chromosomal cluster containing three or more clear BBHs within the context region represents highly cogent evidence for an assertion of function. (Note that Eq. 1 yields a minimum score of at least 0.49 given a conserved context *N* of 3, suggesting that our threshold choice of 0.5 is not unreasonable.)

For each sequence in the foundation set, we projected the foundation set sequence’s functional role onto each PubSEED sequence matching the above criteria, forming the projection sets. The projection sets revealed inconsistencies within the PubSEED, some of which were resolved manually by changing their annotations.

To get a feel for the constraints imposed on projection of the function of gene X in genome 1 onto gene X′ in genome 2, consider the following:

1. X and X′ must be BBHs that also do not violate the “Clear BBH” and gene duplication-filter constraints illustrated in Fig. 1; this is already a fairly restrictive criterion.
2. To achieve a score exceeding 0.5, there must be an absolute minimum of at least one other clear BBH between the 10-gene neighborhoods surrounding X and X′—and even this will only suffice in the very stringent case that X and X′ are more than 99% identical.
3. More typically, an accepted projection must have three or more clear BBHs between the neighborhoods surrounding X and X′.
4. With the selected weights and cutoff, accepted projections from X to X′ had an average context *N* of 5.7 clear BBHs between their respective neighborhoods, and the projections from X and X′ averaged 79% identity.

Because our scoring function weights the value of BBH clustering within the neighboring genes quite highly, and since it is unlikely that such BBH clusters would occur due to pure chance in significantly diverged genomes, we consider our choices of scoring weights and threshold to be quite restrictive.

In addition to identifying inconsistencies via projections, inconsistencies between annotation of proteins with identical sequences were also identified. We looked at all inconsistencies of this type that involved one of the functional roles from our initial list. We were able to resolve many of these inconsistencies manually, or by a database-wide role change; we referred the remaining inconsistencies to expert annotators for resolution.

## Results

Of the roughly 2,500 functional roles employed to build metabolic models within the Model SEED, 1,072 functional roles were not previously supported by DLits (sm1). For 655 of these previously unconnected functional roles (sm3), we were able to attach 2,478 DLits that connected to 1,242 unique protein sequences within the SEED. These 1,242 protein sequences correspond to 21,491 genes (sm2) which encode one of these unique protein sequences. Of the 655 roles for which we were able to attach a DLIT to a sequence, only 484 exactly matched a role taken from the Model SEED, and are thus guaranteed to be recognized during model building. The remaining 171 roles (sm5) were not exactly identical to one of the original roles, due to slight annotation differences of genes with identical sequences in different organisms. Eleven of the 171 changed from their original annotation in the list of 1,072 functional roles that we were looking for during the time that we were making the attachments, as a result of the ongoing SEED annotation effort.

When building the projection set, we found that 518 (sm7) functions met our criteria for projection. These were projected onto 20,336 (sm7) unique protein sequences, corresponding to 57,312 (sm7) genes. Many of the projected functions differed from previous annotations, resulting in 120 discrepancies between our projected annotations and the annotated function already in the databases. These were analyzed and corrected manually as described above. Of the 57,312 genes matching the projection criteria, the functions for 26,785 (sm6) of them were changed, 219 of which were to previously uncharacterized proteins.

The roles to which we attached DLits appear in all of the 214 public Model SEED models. The addition of DLits for the 655 roles provides a higher degree of confidence for the assignment given to these genes in the models, strengthening our overall confidence in the models.

## Discussion

Many difficulties and inconsistencies encountered stemmed from the larger nomenclature problems that plague the fields of biology and bioinformatics. Different databases and annotators inevitably assign different functional roles or levels of specificity to genes that perform the same functions. Even within the SEED, many synonyms exist that refer to identical functions. Such instances are picked up as inconsistencies, even when they are essentially identical, due to differences in vocabulary and formatting. For example, the function “Multidrug and toxin extrusion (MATE) family efflux pump YdhE/NorM” will be classified as not identical to “Multi antimicrobial extrusion protein (Na(+)/drug antiporter), MATE family of MDR efflux pumps” (http://pubseed.theseed.org/seedviewer.cgi?page=Annotation&feature=fig|83333.1.peg.1649) even though these two names refer to the same protein sequence. By examining inconsistencies such as these revealed in the databases by the projection sets, we were able to correct and standardize instances of misannotated functions; we have thus improved the overall quality of genomic data available to the community by correcting inconsistencies in the SEED.

Another factor to consider is the trade-off made by choosing to emphasize quality over quantity (or vice versa) in DLit attachment. Some databases choose to focus on quantity, and attach any research publication that mentions the functional role or gene in question, without any sort of filter. Others put heavy emphasis on quality, and only accept those publications pronouncing results directly from the original laboratory experiment. Our team adopted a moderate approach between the two extremes by searching for papers asserting an explicit connection between a gene and its function. We eliminated papers announcing the complete sequencing of a genome, for example, because these failed to assert specific connections between protein sequences and their respective functions. Had we chosen to emphasize either quality or quantity, the number of DLit attachments made would have been altered. Strengthening our criteria would have reduced the number of DLits attached, while loosening the criteria would have increased the number of attachments, albeit also including more false-positives.

A third major factor influencing our results was the thresholds set for determining similarity between two sequences. For example, only projections with a score assignment of 0.5 or greater were made after computation with Eq. 1 above. This score threshold was set to give us projections with a reasonable degree of confidence, since it typically requires at least three other clear BBHs within the context neighborhoods. A second threshold was the 80% length coverage required for the region of match, to eliminate spurious hits against single protein domains and against fused genes. We also chose to eliminate recent duplications by defining a “Clear BBH” as a match between two protein sequences such that the difference in percent identity between the BBH and the second best hit for either sequence was >5%. Increasing or decreasing any of these values would have effectively strengthened or loosened the criteria for projection, thereby having an effect on the number and accuracy of projections made.

## Conclusion

Overall, this project led to quality improvement in the following aspects of the SEED: the annotations in the PubSEED, the subsequent projections, and the metabolic models of the Model SEED. Expanding the foundation set led to new and corrected annotations in the PubSEED, which improved the databases on the whole, making them more reliable, current, and accurate. The improved foundation set served as the base for subsequent work, most notably the projections. Also, many nomenclature inconsistencies in the databases were resolved, refining the SEED by standardizing the names and punctuation format used for the functional roles that we looked at.

The improved annotations in the databases and expansion of the foundation set, in turn, led to a greater quantity of accurate projections. Since the projections are based on the annotated foundation set, the projections benefit from the improvement in the quality of the annotations. Thus, the projections made were more accurate, and were made with more confidence than previously possible. These two factors, improved annotations and projections, greatly influence the rate, accuracy, and ease with which genomes can be annotated. Most significantly, the overall improvement of these aspects of the SEED enhances our confidence in the metabolic models within the Model SEED.

This project represents a significant step toward the improvement of the quality of genomic information made available in the SEED, including the PubSEED and the Model SEED, because it resulted in better annotations, projections, and models.

## Electronic supplementary material

Below is the link to the electronic supplementary material.

Supplementary material 1 (TXT 39 kb)

Supplementary material 2 (TXT 24 kb)

Supplementary material 3 (TXT 14 kb)

Supplementary material 4 (HTM 2462 kb)

Supplementary material 5 (TXT 217 kb)

Supplementary material 6 (TXT 8388 kb)

Supplementary material 7 (TXT 55 kb)
