# Supplementary material for: Grounding annotations in published literature with an emphasis on the functional roles used in metabolic models
Source: 3 Biotech. 2011 Dec 14;2(2):135–40. doi: 10.1007/s13205-011-0039-z (PMC3376863; doi:10.1007/s13205-011-0039-z)
Supplement: Supplementary file 4 — Supplementary material 4 (HTM 2462 kb) [file 13205_2011_39_MOESM4_ESM.htm]

```
SEED Gene md5 identifier		SEED Gene Identier	Function
e66a01a1cfc113875b2036662f3c2281	fig|509173.8.peg.40	S-(hydroxymethyl)glutathione dehydrogenase (EC 1.1.1.284)
e66a01a1cfc113875b2036662f3c2281	fig|575564.3.peg.3236	S-(hydroxymethyl)glutathione dehydrogenase (EC 1.1.1.284)
e66a01a1cfc113875b2036662f3c2281	fig|575565.3.peg.3463	S-(hydroxymethyl)glutathione dehydrogenase (EC 1.1.1.284)
e66a01a1cfc113875b2036662f3c2281	fig|575586.4.peg.731	S-(hydroxymethyl)glutathione dehydrogenase (EC 1.1.1.284)
e66a01a1cfc113875b2036662f3c2281	fig|575589.3.peg.513	S-(hydroxymethyl)glutathione dehydrogenase (EC 1.1.1.284)
e66a01a1cfc113875b2036662f3c2281	fig|596318.3.peg.463	S-(hydroxymethyl)glutathione dehydrogenase (EC 1.1.1.284)
71f902b48111bc07f9cbfc3671e68ee4	fig|227377.1.peg.1338	Citrate synthase (si) (EC 2.3.3.1)
71f902b48111bc07f9cbfc3671e68ee4	fig|227377.7.peg.1410	Citrate synthase (si) (EC 2.3.3.1)
3d96cb1101bbc5af060f8eea8e42733a	fig|316385.5.peg.4142	Homoserine O-succinyltransferase (EC 2.3.1.46)
3d96cb1101bbc5af060f8eea8e42733a	fig|316385.7.peg.4228	Homoserine O-succinyltransferase (EC 2.3.1.46)
3d96cb1101bbc5af060f8eea8e42733a	fig|316407.3.peg.3850	Homoserine O-succinyltransferase (EC 2.3.1.46)
3d96cb1101bbc5af060f8eea8e42733a	fig|511145.12.peg.4125	Homoserine O-succinyltransferase (EC 2.3.1.46)
3d96cb1101bbc5af060f8eea8e42733a	fig|511145.6.peg.4107	Homoserine O-succinyltransferase (EC 2.3.1.46)
3d96cb1101bbc5af060f8eea8e42733a	fig|536056.3.peg.4235	Homoserine O-succinyltransferase (EC 2.3.1.46)
3d96cb1101bbc5af060f8eea8e42733a	fig|595496.3.peg.4009	Homoserine O-succinyltransferase (EC 2.3.1.46)
3d96cb1101bbc5af060f8eea8e42733a	fig|83333.1.peg.3922	Homoserine O-succinyltransferase (EC 2.3.1.46)
12c49188e28038b39c9508073edea871	fig|155864.1.peg.5305	Phosphopentomutase (EC 5.4.2.7)
12c49188e28038b39c9508073edea871	fig|155864.8.peg.5301	Phosphopentomutase (EC 5.4.2.7)
12c49188e28038b39c9508073edea871	fig|198214.1.peg.4163	Phosphopentomutase (EC 5.4.2.7)
12c49188e28038b39c9508073edea871	fig|198214.7.peg.5203	Phosphopentomutase (EC 5.4.2.7)
12c49188e28038b39c9508073edea871	fig|198215.1.peg.4050	Phosphopentomutase (EC 5.4.2.7)
12c49188e28038b39c9508073edea871	fig|198215.6.peg.5112	Phosphopentomutase (EC 5.4.2.7)
12c49188e28038b39c9508073edea871	fig|199310.1.peg.5356	Phosphopentomutase (EC 5.4.2.7)
12c49188e28038b39c9508073edea871	fig|199310.4.peg.5119	Phosphopentomutase (EC 5.4.2.7)
12c49188e28038b39c9508073edea871	fig|300268.10.peg.5259	Phosphopentomutase (EC 5.4.2.7)
12c49188e28038b39c9508073edea871	fig|300268.11.peg.5310	Phosphopentomutase (EC 5.4.2.7)
12c49188e28038b39c9508073edea871	fig|316385.5.peg.4490	Phosphopentomutase (EC 5.4.2.7)
12c49188e28038b39c9508073edea871	fig|316385.7.peg.4588	Phosphopentomutase (EC 5.4.2.7)
12c49188e28038b39c9508073edea871	fig|316401.4.peg.5400	Phosphopentomutase (EC 5.4.2.7)
12c49188e28038b39c9508073edea871	fig|316407.3.peg.4207	Phosphopentomutase (EC 5.4.2.7)
12c49188e28038b39c9508073edea871	fig|331111.12.peg.5176	Phosphopentomutase (EC 5.4.2.7)
12c49188e28038b39c9508073edea871	fig|331111.3.peg.2554	Phosphopentomutase (EC 5.4.2.7)
12c49188e28038b39c9508073edea871	fig|331112.3.peg.4323	Phosphopentomutase (EC 5.4.2.7)
12c49188e28038b39c9508073edea871	fig|331112.6.peg.4502	Phosphopentomutase (EC 5.4.2.7)
12c49188e28038b39c9508073edea871	fig|340185.3.peg.4002	Phosphopentomutase (EC 5.4.2.7)
12c49188e28038b39c9508073edea871	fig|340185.4.peg.4227	Phosphopentomutase (EC 5.4.2.7)
12c49188e28038b39c9508073edea871	fig|340186.3.peg.3798	Phosphopentomutase (EC 5.4.2.7)
12c49188e28038b39c9508073edea871	fig|340186.5.peg.3988	Phosphopentomutase (EC 5.4.2.7)
12c49188e28038b39c9508073edea871	fig|344601.3.peg.3492	Phosphopentomutase (EC 5.4.2.7)
12c49188e28038b39c9508073edea871	fig|344601.5.peg.3651	Phosphopentomutase (EC 5.4.2.7)
12c49188e28038b39c9508073edea871	fig|344609.11.peg.5380	Phosphopentomutase (EC 5.4.2.7)
12c49188e28038b39c9508073edea871	fig|344609.3.peg.4350	Phosphopentomutase (EC 5.4.2.7)
12c49188e28038b39c9508073edea871	fig|344610.3.peg.731	Phosphopentomutase (EC 5.4.2.7)
12c49188e28038b39c9508073edea871	fig|344610.7.peg.1765	Phosphopentomutase (EC 5.4.2.7)
12c49188e28038b39c9508073edea871	fig|358708.5.peg.3937	Phosphopentomutase (EC 5.4.2.7)
12c49188e28038b39c9508073edea871	fig|358709.5.peg.4015	Phosphopentomutase (EC 5.4.2.7)
12c49188e28038b39c9508073edea871	fig|373384.10.peg.4958	Phosphopentomutase (EC 5.4.2.7)
12c49188e28038b39c9508073edea871	fig|373384.11.peg.5010	Phosphopentomutase (EC 5.4.2.7)
12c49188e28038b39c9508073edea871	fig|386585.9.peg.5589	Phosphopentomutase (EC 5.4.2.7)
12c49188e28038b39c9508073edea871	fig|409438.11.peg.4848	Phosphopentomutase (EC 5.4.2.7)
12c49188e28038b39c9508073edea871	fig|431946.3.peg.4501	Phosphopentomutase (EC 5.4.2.7)
12c49188e28038b39c9508073edea871	fig|439855.10.peg.5029	Phosphopentomutase (EC 5.4.2.7)
12c49188e28038b39c9508073edea871	fig|444447.5.peg.2815	Phosphopentomutase (EC 5.4.2.7)
12c49188e28038b39c9508073edea871	fig|444448.5.peg.2649	Phosphopentomutase (EC 5.4.2.7)
12c49188e28038b39c9508073edea871	fig|444449.5.peg.3891	Phosphopentomutase (EC 5.4.2.7)
12c49188e28038b39c9508073edea871	fig|444450.8.peg.5729	Phosphopentomutase (EC 5.4.2.7)
12c49188e28038b39c9508073edea871	fig|444451.5.peg.4131	Phosphopentomutase (EC 5.4.2.7)
12c49188e28038b39c9508073edea871	fig|444452.5.peg.3888	Phosphopentomutase (EC 5.4.2.7)
12c49188e28038b39c9508073edea871	fig|444453.5.peg.3086	Phosphopentomutase (EC 5.4.2.7)
12c49188e28038b39c9508073edea871	fig|444454.5.peg.4442	Phosphopentomutase (EC 5.4.2.7)
12c49188e28038b39c9508073edea871	fig|457401.3.peg.3484	Phosphopentomutase (EC 5.4.2.7)
12c49188e28038b39c9508073edea871	fig|469598.5.peg.3761	Phosphopentomutase (EC 5.4.2.7)
12c49188e28038b39c9508073edea871	fig|478004.5.peg.3501	Phosphopentomutase (EC 5.4.2.7)
12c49188e28038b39c9508073edea871	fig|478005.5.peg.857	Phosphopentomutase (EC 5.4.2.7)
12c49188e28038b39c9508073edea871	fig|478006.5.peg.3169	Phosphopentomutase (EC 5.4.2.7)
12c49188e28038b39c9508073edea871	fig|478007.5.peg.799	Phosphopentomutase (EC 5.4.2.7)
12c49188e28038b39c9508073edea871	fig|478008.5.peg.409	Phosphopentomutase (EC 5.4.2.7)
12c49188e28038b39c9508073edea871	fig|481805.3.peg.3946	Phosphopentomutase (EC 5.4.2.7)
12c49188e28038b39c9508073edea871	fig|481805.6.peg.3928	Phosphopentomutase (EC 5.4.2.7)
12c49188e28038b39c9508073edea871	fig|502346.5.peg.1283	Phosphopentomutase (EC 5.4.2.7)
12c49188e28038b39c9508073edea871	fig|502347.3.peg.3736	Phosphopentomutase (EC 5.4.2.7)
12c49188e28038b39c9508073edea871	fig|511145.12.peg.4531	Phosphopentomutase (EC 5.4.2.7)
12c49188e28038b39c9508073edea871	fig|511145.6.peg.4508	Phosphopentomutase (EC 5.4.2.7)
12c49188e28038b39c9508073edea871	fig|525281.3.peg.1295	Phosphopentomutase (EC 5.4.2.7)
12c49188e28038b39c9508073edea871	fig|536056.3.peg.3825	Phosphopentomutase (EC 5.4.2.7)
12c49188e28038b39c9508073edea871	fig|544404.4.peg.5538	Phosphopentomutase (EC 5.4.2.7)
12c49188e28038b39c9508073edea871	fig|550672.3.peg.4252	Phosphopentomutase (EC 5.4.2.7)
12c49188e28038b39c9508073edea871	fig|550676.3.peg.4642	Phosphopentomutase (EC 5.4.2.7)
12c49188e28038b39c9508073edea871	fig|550677.3.peg.353	Phosphopentomutase (EC 5.4.2.7)
12c49188e28038b39c9508073edea871	fig|556266.3.peg.2815	Phosphopentomutase (EC 5.4.2.7)
12c49188e28038b39c9508073edea871	fig|562.371.peg.3908	Phosphopentomutase (EC 5.4.2.7)
12c49188e28038b39c9508073edea871	fig|562.372.peg.3148	Phosphopentomutase (EC 5.4.2.7)
12c49188e28038b39c9508073edea871	fig|562.373.peg.2297	Phosphopentomutase (EC 5.4.2.7)
12c49188e28038b39c9508073edea871	fig|562.374.peg.2920	Phosphopentomutase (EC 5.4.2.7)
12c49188e28038b39c9508073edea871	fig|562.375.peg.2686	Phosphopentomutase (EC 5.4.2.7)
12c49188e28038b39c9508073edea871	fig|562.376.peg.918	Phosphopentomutase (EC 5.4.2.7)
12c49188e28038b39c9508073edea871	fig|566546.3.peg.3687	Phosphopentomutase (EC 5.4.2.7)
12c49188e28038b39c9508073edea871	fig|566546.4.peg.4687	Phosphopentomutase (EC 5.4.2.7)
12c49188e28038b39c9508073edea871	fig|570506.3.peg.981	Phosphopentomutase (EC 5.4.2.7)
12c49188e28038b39c9508073edea871	fig|573235.3.peg.5786	Phosphopentomutase (EC 5.4.2.7)
12c49188e28038b39c9508073edea871	fig|585034.4.peg.4476	Phosphopentomutase (EC 5.4.2.7)
12c49188e28038b39c9508073edea871	fig|585034.5.peg.4472	Phosphopentomutase (EC 5.4.2.7)
12c49188e28038b39c9508073edea871	fig|585035.6.peg.4963	Phosphopentomutase (EC 5.4.2.7)
12c49188e28038b39c9508073edea871	fig|585054.5.peg.4306	Phosphopentomutase (EC 5.4.2.7)
12c49188e28038b39c9508073edea871	fig|585055.6.peg.5051	Phosphopentomutase (EC 5.4.2.7)
12c49188e28038b39c9508073edea871	fig|585055.8.peg.5056	Phosphopentomutase (EC 5.4.2.7)
12c49188e28038b39c9508073edea871	fig|585056.7.peg.5172	Phosphopentomutase (EC 5.4.2.7)
12c49188e28038b39c9508073edea871	fig|585057.4.peg.5065	Phosphopentomutase (EC 5.4.2.7)
12c49188e28038b39c9508073edea871	fig|585057.6.peg.5077	Phosphopentomutase (EC 5.4.2.7)
12c49188e28038b39c9508073edea871	fig|585395.4.peg.5507	Phosphopentomutase (EC 5.4.2.7)
12c49188e28038b39c9508073edea871	fig|585396.4.peg.5475	Phosphopentomutase (EC 5.4.2.7)
12c49188e28038b39c9508073edea871	fig|585397.7.peg.5226	Phosphopentomutase (EC 5.4.2.7)
12c49188e28038b39c9508073edea871	fig|585397.9.peg.5227	Phosphopentomutase (EC 5.4.2.7)
12c49188e28038b39c9508073edea871	fig|591020.3.peg.5169	Phosphopentomutase (EC 5.4.2.7)
12c49188e28038b39c9508073edea871	fig|595495.4.peg.4399	Phosphopentomutase (EC 5.4.2.7)
12c49188e28038b39c9508073edea871	fig|595496.3.peg.4476	Phosphopentomutase (EC 5.4.2.7)
12c49188e28038b39c9508073edea871	fig|621.8.peg.3835	Phosphopentomutase (EC 5.4.2.7)
12c49188e28038b39c9508073edea871	fig|622.8.peg.3508	Phosphopentomutase (EC 5.4.2.7)
12c49188e28038b39c9508073edea871	fig|623.7.peg.461	Phosphopentomutase (EC 5.4.2.7)
12c49188e28038b39c9508073edea871	fig|637388.3.peg.3819	Phosphopentomutase (EC 5.4.2.7)
12c49188e28038b39c9508073edea871	fig|655817.3.peg.5229	Phosphopentomutase (EC 5.4.2.7)
12c49188e28038b39c9508073edea871	fig|656379.3.peg.207	Phosphopentomutase (EC 5.4.2.7)
12c49188e28038b39c9508073edea871	fig|656380.3.peg.145	Phosphopentomutase (EC 5.4.2.7)
12c49188e28038b39c9508073edea871	fig|656408.3.peg.4924	Phosphopentomutase (EC 5.4.2.7)
12c49188e28038b39c9508073edea871	fig|656414.3.peg.89	Phosphopentomutase (EC 5.4.2.7)
12c49188e28038b39c9508073edea871	fig|656419.3.peg.129	Phosphopentomutase (EC 5.4.2.7)
12c49188e28038b39c9508073edea871	fig|656437.3.peg.18	Phosphopentomutase (EC 5.4.2.7)
12c49188e28038b39c9508073edea871	fig|656443.3.peg.98	Phosphopentomutase (EC 5.4.2.7)
12c49188e28038b39c9508073edea871	fig|656444.3.peg.288	Phosphopentomutase (EC 5.4.2.7)
12c49188e28038b39c9508073edea871	fig|6666666.5522.peg.4171	Phosphopentomutase (EC 5.4.2.7)
12c49188e28038b39c9508073edea871	fig|670888.3.peg.558	Phosphopentomutase (EC 5.4.2.7)
12c49188e28038b39c9508073edea871	fig|670897.3.peg.1792	Phosphopentomutase (EC 5.4.2.7)
12c49188e28038b39c9508073edea871	fig|679204.3.peg.2790	Phosphopentomutase (EC 5.4.2.7)
12c49188e28038b39c9508073edea871	fig|679205.4.peg.2007	Phosphopentomutase (EC 5.4.2.7)
12c49188e28038b39c9508073edea871	fig|679206.4.peg.4181	Phosphopentomutase (EC 5.4.2.7)
12c49188e28038b39c9508073edea871	fig|679207.4.peg.1011	Phosphopentomutase (EC 5.4.2.7)
12c49188e28038b39c9508073edea871	fig|701177.3.peg.5259	Phosphopentomutase (EC 5.4.2.7)
12c49188e28038b39c9508073edea871	fig|749527.3.peg.3964	Phosphopentomutase (EC 5.4.2.7)
12c49188e28038b39c9508073edea871	fig|749528.3.peg.2116	Phosphopentomutase (EC 5.4.2.7)
12c49188e28038b39c9508073edea871	fig|749531.3.peg.3919	Phosphopentomutase (EC 5.4.2.7)
12c49188e28038b39c9508073edea871	fig|749532.3.peg.4298	Phosphopentomutase (EC 5.4.2.7)
12c49188e28038b39c9508073edea871	fig|749533.3.peg.3255	Phosphopentomutase (EC 5.4.2.7)
12c49188e28038b39c9508073edea871	fig|749537.3.peg.1826	Phosphopentomutase (EC 5.4.2.7)
12c49188e28038b39c9508073edea871	fig|749538.3.peg.1035	Phosphopentomutase (EC 5.4.2.7)
12c49188e28038b39c9508073edea871	fig|749540.3.peg.3315	Phosphopentomutase (EC 5.4.2.7)
12c49188e28038b39c9508073edea871	fig|749544.3.peg.1454	Phosphopentomutase (EC 5.4.2.7)
12c49188e28038b39c9508073edea871	fig|749546.3.peg.2273	Phosphopentomutase (EC 5.4.2.7)
12c49188e28038b39c9508073edea871	fig|749548.3.peg.2320	Phosphopentomutase (EC 5.4.2.7)
12c49188e28038b39c9508073edea871	fig|749549.3.peg.997	Phosphopentomutase (EC 5.4.2.7)
12c49188e28038b39c9508073edea871	fig|753642.3.peg.3668	Phosphopentomutase (EC 5.4.2.7)
12c49188e28038b39c9508073edea871	fig|83333.1.peg.4291	Phosphopentomutase (EC 5.4.2.7)
12c49188e28038b39c9508073edea871	fig|83334.1.peg.5322	Phosphopentomutase (EC 5.4.2.7)
675ae57204f1bb21e5b32ecff04d97d5	fig|9606.3.peg.31528	Methylglutaconyl-CoA hydratase (EC 4.2.1.18)
8feb1487f865d2d21a0b8db53d7c859e	fig|269084.3.peg.746	1-deoxy-D-xylulose 5-phosphate synthase (EC 2.2.1.7)
8feb1487f865d2d21a0b8db53d7c859e	fig|269084.6.peg.1259	1-deoxy-D-xylulose 5-phosphate synthase (EC 2.2.1.7)
647d2ef21f1a437c8da5d84a3e65ab5a	fig|3702.1.peg.28091	Nitrate/nitrite transporter
647d2ef21f1a437c8da5d84a3e65ab5a	fig|3702.7.peg.27388	nitrate transporter 2.4
1e679e31a4025e71e592af82d9aec514	fig|216592.1.peg.2689	Ribonucleotide reductase of class III (anaerobic), large subunit (EC 1.17.4.2)
1e679e31a4025e71e592af82d9aec514	fig|216592.3.peg.4900	Ribonucleotide reductase of class III (anaerobic), large subunit (EC 1.17.4.2)
1e679e31a4025e71e592af82d9aec514	fig|316385.7.peg.4477	Ribonucleotide reductase of class III (anaerobic), large subunit (EC 1.17.4.2)
1e679e31a4025e71e592af82d9aec514	fig|316407.3.peg.4072	Ribonucleotide reductase of class III (anaerobic), large subunit (EC 1.17.4.2)
1e679e31a4025e71e592af82d9aec514	fig|331112.3.peg.4204	Ribonucleotide reductase of class III (anaerobic), large subunit (EC 1.17.4.2)
1e679e31a4025e71e592af82d9aec514	fig|331112.6.peg.4376	Ribonucleotide reductase of class III (anaerobic), large subunit (EC 1.17.4.2)
1e679e31a4025e71e592af82d9aec514	fig|340185.3.peg.1991	Ribonucleotide reductase of class III (anaerobic), large subunit (EC 1.17.4.2)
1e679e31a4025e71e592af82d9aec514	fig|340185.4.peg.2107	Ribonucleotide reductase of class III (anaerobic), large subunit (EC 1.17.4.2)
1e679e31a4025e71e592af82d9aec514	fig|344601.3.peg.891	Ribonucleotide reductase of class III (anaerobic), large subunit (EC 1.17.4.2)
1e679e31a4025e71e592af82d9aec514	fig|344601.5.peg.942	Ribonucleotide reductase of class III (anaerobic), large subunit (EC 1.17.4.2)
1e679e31a4025e71e592af82d9aec514	fig|358709.5.peg.2440	Ribonucleotide reductase of class III (anaerobic), large subunit (EC 1.17.4.2)
1e679e31a4025e71e592af82d9aec514	fig|409438.11.peg.4725	Ribonucleotide reductase of class III (anaerobic), large subunit (EC 1.17.4.2)
1e679e31a4025e71e592af82d9aec514	fig|413997.3.peg.4332	Ribonucleotide reductase of class III (anaerobic), large subunit (EC 1.17.4.2)
1e679e31a4025e71e592af82d9aec514	fig|457400.3.peg.523	Ribonucleotide reductase of class III (anaerobic), large subunit (EC 1.17.4.2)
1e679e31a4025e71e592af82d9aec514	fig|469008.4.peg.3937	Ribonucleotide reductase of class III (anaerobic), large subunit (EC 1.17.4.2)
1e679e31a4025e71e592af82d9aec514	fig|511145.12.peg.4369	Ribonucleotide reductase of class III (anaerobic), large subunit (EC 1.17.4.2)
1e679e31a4025e71e592af82d9aec514	fig|511145.6.peg.4347	Ribonucleotide reductase of class III (anaerobic), large subunit (EC 1.17.4.2)
1e679e31a4025e71e592af82d9aec514	fig|511693.5.peg.4354	Ribonucleotide reductase of class III (anaerobic), large subunit (EC 1.17.4.2)
1e679e31a4025e71e592af82d9aec514	fig|536056.3.peg.3989	Ribonucleotide reductase of class III (anaerobic), large subunit (EC 1.17.4.2)
1e679e31a4025e71e592af82d9aec514	fig|550672.3.peg.4589	Ribonucleotide reductase of class III (anaerobic), large subunit (EC 1.17.4.2)
1e679e31a4025e71e592af82d9aec514	fig|550676.3.peg.4514	Ribonucleotide reductase of class III (anaerobic), large subunit (EC 1.17.4.2)
1e679e31a4025e71e592af82d9aec514	fig|556266.3.peg.2693	Ribonucleotide reductase of class III (anaerobic), large subunit (EC 1.17.4.2)
1e679e31a4025e71e592af82d9aec514	fig|573235.3.peg.5589	Ribonucleotide reductase of class III (anaerobic), large subunit (EC 1.17.4.2)
1e679e31a4025e71e592af82d9aec514	fig|585034.4.peg.4347	Ribonucleotide reductase of class III (anaerobic), large subunit (EC 1.17.4.2)
1e679e31a4025e71e592af82d9aec514	fig|585034.5.peg.4344	Ribonucleotide reductase of class III (anaerobic), large subunit (EC 1.17.4.2)
1e679e31a4025e71e592af82d9aec514	fig|585056.7.peg.4935	Ribonucleotide reductase of class III (anaerobic), large subunit (EC 1.17.4.2)
1e679e31a4025e71e592af82d9aec514	fig|585396.4.peg.5339	Ribonucleotide reductase of class III (anaerobic), large subunit (EC 1.17.4.2)
1e679e31a4025e71e592af82d9aec514	fig|595496.3.peg.4312	Ribonucleotide reductase of class III (anaerobic), large subunit (EC 1.17.4.2)
1e679e31a4025e71e592af82d9aec514	fig|622.8.peg.3039	Ribonucleotide reductase of class III (anaerobic), large subunit (EC 1.17.4.2)
1e679e31a4025e71e592af82d9aec514	fig|656379.3.peg.615	Ribonucleotide reductase of class III (anaerobic), large subunit (EC 1.17.4.2)
1e679e31a4025e71e592af82d9aec514	fig|656380.3.peg.606	Ribonucleotide reductase of class III (anaerobic), large subunit (EC 1.17.4.2)
1e679e31a4025e71e592af82d9aec514	fig|656408.3.peg.4748	Ribonucleotide reductase of class III (anaerobic), large subunit (EC 1.17.4.2)
1e679e31a4025e71e592af82d9aec514	fig|656414.3.peg.4809	Ribonucleotide reductase of class III (anaerobic), large subunit (EC 1.17.4.2)
1e679e31a4025e71e592af82d9aec514	fig|656419.3.peg.5493	Ribonucleotide reductase of class III (anaerobic), large subunit (EC 1.17.4.2)
1e679e31a4025e71e592af82d9aec514	fig|670897.3.peg.1613	Ribonucleotide reductase of class III (anaerobic), large subunit (EC 1.17.4.2)
1e679e31a4025e71e592af82d9aec514	fig|679205.4.peg.3187	Ribonucleotide reductase of class III (anaerobic), large subunit (EC 1.17.4.2)
1e679e31a4025e71e592af82d9aec514	fig|679206.4.peg.1110	Ribonucleotide reductase of class III (anaerobic), large subunit (EC 1.17.4.2)
1e679e31a4025e71e592af82d9aec514	fig|749533.3.peg.3870	Ribonucleotide reductase of class III (anaerobic), large subunit (EC 1.17.4.2)
1e679e31a4025e71e592af82d9aec514	fig|749548.3.peg.3803	Ribonucleotide reductase of class III (anaerobic), large subunit (EC 1.17.4.2)
1e679e31a4025e71e592af82d9aec514	fig|749549.3.peg.3284	Ribonucleotide reductase of class III (anaerobic), large subunit (EC 1.17.4.2)
1e679e31a4025e71e592af82d9aec514	fig|83333.1.peg.4148	Ribonucleotide reductase of class III (anaerobic), large subunit (EC 1.17.4.2)
09afa515ef6c69d3d0c074b4a8db9de0	fig|196620.1.peg.1487	Glycine dehydrogenase [decarboxylating] (glycine cleavage system P2 protein) (EC 1.4.4.2)
09afa515ef6c69d3d0c074b4a8db9de0	fig|196620.5.peg.1553	Glycine dehydrogenase [decarboxylating] (glycine cleavage system P2 protein) (EC 1.4.4.2)
09afa515ef6c69d3d0c074b4a8db9de0	fig|282459.1.peg.1459	Glycine dehydrogenase [decarboxylating] (glycine cleavage system P2 protein) (EC 1.4.4.2)
09afa515ef6c69d3d0c074b4a8db9de0	fig|282459.5.peg.1533	Glycine dehydrogenase [decarboxylating] (glycine cleavage system P2 protein) (EC 1.4.4.2)
09afa515ef6c69d3d0c074b4a8db9de0	fig|367830.3.peg.2506	Glycine dehydrogenase [decarboxylating] (glycine cleavage system P2 protein) (EC 1.4.4.2)
09afa515ef6c69d3d0c074b4a8db9de0	fig|451515.3.peg.1636	Glycine dehydrogenase [decarboxylating] (glycine cleavage system P2 protein) (EC 1.4.4.2)
09afa515ef6c69d3d0c074b4a8db9de0	fig|451516.9.peg.1626	Glycine dehydrogenase [decarboxylating] (glycine cleavage system P2 protein) (EC 1.4.4.2)
09afa515ef6c69d3d0c074b4a8db9de0	fig|455227.3.peg.1121	Glycine dehydrogenase [decarboxylating] (glycine cleavage system P2 protein) (EC 1.4.4.2)
09afa515ef6c69d3d0c074b4a8db9de0	fig|546342.3.peg.823	Glycine dehydrogenase [decarboxylating] (glycine cleavage system P2 protein) (EC 1.4.4.2)
09afa515ef6c69d3d0c074b4a8db9de0	fig|546342.4.peg.1588	Glycine dehydrogenase [decarboxylating] (glycine cleavage system P2 protein) (EC 1.4.4.2)
09afa515ef6c69d3d0c074b4a8db9de0	fig|546343.3.peg.49	Glycine dehydrogenase [decarboxylating] (glycine cleavage system P2 protein) (EC 1.4.4.2)
09afa515ef6c69d3d0c074b4a8db9de0	fig|548475.3.peg.524	Glycine dehydrogenase [decarboxylating] (glycine cleavage system P2 protein) (EC 1.4.4.2)
09afa515ef6c69d3d0c074b4a8db9de0	fig|553567.3.peg.678	Glycine dehydrogenase [decarboxylating] (glycine cleavage system P2 protein) (EC 1.4.4.2)
09afa515ef6c69d3d0c074b4a8db9de0	fig|553590.4.peg.623	Glycine dehydrogenase [decarboxylating] (glycine cleavage system P2 protein) (EC 1.4.4.2)
09afa515ef6c69d3d0c074b4a8db9de0	fig|644279.4.peg.1686	Glycine dehydrogenase [decarboxylating] (glycine cleavage system P2 protein) (EC 1.4.4.2)
09afa515ef6c69d3d0c074b4a8db9de0	fig|663951.4.peg.1638	Glycine dehydrogenase [decarboxylating] (glycine cleavage system P2 protein) (EC 1.4.4.2)
09afa515ef6c69d3d0c074b4a8db9de0	fig|762962.3.peg.451	Glycine dehydrogenase [decarboxylating] (glycine cleavage system P2 protein) (EC 1.4.4.2)
09afa515ef6c69d3d0c074b4a8db9de0	fig|862516.3.peg.1750	Glycine dehydrogenase [decarboxylating] (glycine cleavage system P2 protein) (EC 1.4.4.2)
09afa515ef6c69d3d0c074b4a8db9de0	fig|93061.3.peg.1581	Glycine dehydrogenase [decarboxylating] (glycine cleavage system P2 protein) (EC 1.4.4.2)
09afa515ef6c69d3d0c074b4a8db9de0	fig|93061.5.peg.1485	Glycine dehydrogenase [decarboxylating] (glycine cleavage system P2 protein) (EC 1.4.4.2)
06afe329477ff58e8681ec4343242eb0	fig|10665.1.peg.44	Deoxycytidylate 5-hydroxymethyltransferase (EC 2.1.2.8)
bedd03002b8cc4f2c282bc01f83c4ff2	fig|316385.7.peg.2615	PTS system, sucrose-specific IIB component (EC 2.7.1.69) / PTS system, sucrose-specific IIC component (EC 2.7.1.69)
bedd03002b8cc4f2c282bc01f83c4ff2	fig|316401.4.peg.2904	PTS system, sucrose-specific IIB component (EC 2.7.1.69) / PTS system, sucrose-specific IIC component (EC 2.7.1.69)
bedd03002b8cc4f2c282bc01f83c4ff2	fig|316407.3.peg.2359	PTS system, sucrose-specific IIB component (EC 2.7.1.69) / PTS system, sucrose-specific IIC component (EC 2.7.1.69)
bedd03002b8cc4f2c282bc01f83c4ff2	fig|331112.3.peg.2404	Putative PTS enzyme II
bedd03002b8cc4f2c282bc01f83c4ff2	fig|331112.6.peg.2509	PTS system, sucrose-specific IIB component (EC 2.7.1.69) / PTS system, sucrose-specific IIC component (EC 2.7.1.69)
bedd03002b8cc4f2c282bc01f83c4ff2	fig|344610.3.peg.379	Putative PTS enzyme II
bedd03002b8cc4f2c282bc01f83c4ff2	fig|344610.7.peg.3903	PTS system, sucrose-specific IIB component (EC 2.7.1.69) / PTS system, sucrose-specific IIC component (EC 2.7.1.69)
bedd03002b8cc4f2c282bc01f83c4ff2	fig|457401.3.peg.965	PTS system, sucrose-specific IIB component (EC 2.7.1.69) / PTS system, sucrose-specific IIC component (EC 2.7.1.69)
bedd03002b8cc4f2c282bc01f83c4ff2	fig|481805.3.peg.1338	PTS system, sucrose-specific IIB component (EC 2.7.1.69) / PTS system, sucrose-specific IIC component (EC 2.7.1.69)
bedd03002b8cc4f2c282bc01f83c4ff2	fig|481805.6.peg.1339	PTS system, sucrose-specific IIB component (EC 2.7.1.69) / PTS system, sucrose-specific IIC component (EC 2.7.1.69)
bedd03002b8cc4f2c282bc01f83c4ff2	fig|511145.12.peg.2524	PTS system, sucrose-specific IIB component (EC 2.7.1.69) / PTS system, sucrose-specific IIC component (EC 2.7.1.69)
bedd03002b8cc4f2c282bc01f83c4ff2	fig|511145.6.peg.2509	PTS system, sucrose-specific IIB component (EC 2.7.1.69) / PTS system, sucrose-specific IIC component (EC 2.7.1.69)
bedd03002b8cc4f2c282bc01f83c4ff2	fig|536056.3.peg.1296	PTS system, sucrose-specific IIB component (EC 2.7.1.69) / PTS system, sucrose-specific IIC component (EC 2.7.1.69)
bedd03002b8cc4f2c282bc01f83c4ff2	fig|595496.3.peg.2405	PTS system, sucrose-specific IIB component (EC 2.7.1.69) / PTS system, sucrose-specific IIC component (EC 2.7.1.69)
bedd03002b8cc4f2c282bc01f83c4ff2	fig|656414.3.peg.2801	PTS system, sucrose-specific IIB component (EC 2.7.1.69) / PTS system, sucrose-specific IIC component (EC 2.7.1.69)
bedd03002b8cc4f2c282bc01f83c4ff2	fig|670888.3.peg.3862	PTS system, sucrose-specific IIB component (EC 2.7.1.69) / PTS system, sucrose-specific IIC component (EC 2.7.1.69)
bedd03002b8cc4f2c282bc01f83c4ff2	fig|749537.3.peg.4730	PTS system, sucrose-specific IIB component (EC 2.7.1.69) / PTS system, sucrose-specific IIC component (EC 2.7.1.69)
bedd03002b8cc4f2c282bc01f83c4ff2	fig|749540.3.peg.4387	PTS system, sucrose-specific IIB component (EC 2.7.1.69) / PTS system, sucrose-specific IIC component (EC 2.7.1.69)
bedd03002b8cc4f2c282bc01f83c4ff2	fig|749548.3.peg.1962	PTS system, sucrose-specific IIB component (EC 2.7.1.69) / PTS system, sucrose-specific IIC component (EC 2.7.1.69)
bedd03002b8cc4f2c282bc01f83c4ff2	fig|83333.1.peg.2397	PTS system, sucrose-specific IIB component (EC 2.7.1.69) / PTS system, sucrose-specific IIC component (EC 2.7.1.69)
08d2d762732503ae9967349781cc7df9	fig|266834.11.peg.641	Acetyl-CoA synthetase (ADP-forming) alpha and beta chains, putative
08d2d762732503ae9967349781cc7df9	fig|266834.1.peg.619	Acetyl-CoA synthetase (ADP-forming) alpha and beta chains, putative
8f67f9bf0ea7987c5edf036c3a6801fb	fig|85962.1.peg.69	Urea channel UreI
8f67f9bf0ea7987c5edf036c3a6801fb	fig|85962.8.peg.73	Urea channel UreI
0a97130d8b9dad338086f6a9716d89d4	fig|316385.5.peg.2412	NADH-ubiquinone oxidoreductase chain G (EC 1.6.5.3)
0a97130d8b9dad338086f6a9716d89d4	fig|316385.7.peg.2468	NADH-ubiquinone oxidoreductase chain G (EC 1.6.5.3)
0a97130d8b9dad338086f6a9716d89d4	fig|316401.4.peg.2765	NADH-ubiquinone oxidoreductase chain G (EC 1.6.5.3)
0a97130d8b9dad338086f6a9716d89d4	fig|457401.3.peg.1594	NADH-ubiquinone oxidoreductase chain G (EC 1.6.5.3)
0a97130d8b9dad338086f6a9716d89d4	fig|481805.6.peg.1462	NADH-ubiquinone oxidoreductase chain G (EC 1.6.5.3)
0a97130d8b9dad338086f6a9716d89d4	fig|511145.12.peg.2376	NADH-ubiquinone oxidoreductase chain G (EC 1.6.5.3)
0a97130d8b9dad338086f6a9716d89d4	fig|511145.6.peg.2360	NADH-ubiquinone oxidoreductase chain G (EC 1.6.5.3)
0a97130d8b9dad338086f6a9716d89d4	fig|536056.3.peg.1444	NADH-ubiquinone oxidoreductase chain G (EC 1.6.5.3)
0a97130d8b9dad338086f6a9716d89d4	fig|595496.3.peg.2258	NADH-ubiquinone oxidoreductase chain G (EC 1.6.5.3)
0a97130d8b9dad338086f6a9716d89d4	fig|670888.3.peg.3992	NADH-ubiquinone oxidoreductase chain G (EC 1.6.5.3)
0a97130d8b9dad338086f6a9716d89d4	fig|749540.3.peg.3958	NADH-ubiquinone oxidoreductase chain G (EC 1.6.5.3)
0a97130d8b9dad338086f6a9716d89d4	fig|749544.3.peg.2189	NADH-ubiquinone oxidoreductase chain G (EC 1.6.5.3)
0a97130d8b9dad338086f6a9716d89d4	fig|749548.3.peg.3135	NADH-ubiquinone oxidoreductase chain G (EC 1.6.5.3)
0a97130d8b9dad338086f6a9716d89d4	fig|83333.1.peg.2257	NADH-ubiquinone oxidoreductase chain G (EC 1.6.5.3)
84044b10cc38bdc6b5a13d1ae107b0b9	fig|216592.3.peg.3246	Aminomethyltransferase (glycine cleavage system T protein) (EC 2.1.2.10)
84044b10cc38bdc6b5a13d1ae107b0b9	fig|316385.7.peg.3098	Aminomethyltransferase (glycine cleavage system T protein) (EC 2.1.2.10)
84044b10cc38bdc6b5a13d1ae107b0b9	fig|316401.4.peg.3504	Aminomethyltransferase (glycine cleavage system T protein) (EC 2.1.2.10)
84044b10cc38bdc6b5a13d1ae107b0b9	fig|316407.3.peg.2800	Aminomethyltransferase (glycine cleavage system T protein) (EC 2.1.2.10)
84044b10cc38bdc6b5a13d1ae107b0b9	fig|331111.12.peg.3505	Aminomethyltransferase (glycine cleavage system T protein) (EC 2.1.2.10)
84044b10cc38bdc6b5a13d1ae107b0b9	fig|331111.3.peg.934	Aminomethyltransferase (glycine cleavage system T protein) (EC 2.1.2.10)
84044b10cc38bdc6b5a13d1ae107b0b9	fig|331112.3.peg.2860	Aminomethyltransferase (glycine cleavage system T protein) (EC 2.1.2.10)
84044b10cc38bdc6b5a13d1ae107b0b9	fig|331112.6.peg.2993	Aminomethyltransferase (glycine cleavage system T protein) (EC 2.1.2.10)
84044b10cc38bdc6b5a13d1ae107b0b9	fig|340184.3.peg.4599	Aminomethyltransferase (glycine cleavage system T protein) (EC 2.1.2.10)
84044b10cc38bdc6b5a13d1ae107b0b9	fig|340184.6.peg.4828	Aminomethyltransferase (glycine cleavage system T protein) (EC 2.1.2.10)
84044b10cc38bdc6b5a13d1ae107b0b9	fig|340185.3.peg.1886	Aminomethyltransferase (glycine cleavage system T protein) (EC 2.1.2.10)
84044b10cc38bdc6b5a13d1ae107b0b9	fig|340185.4.peg.1997	Aminomethyltransferase (glycine cleavage system T protein) (EC 2.1.2.10)
84044b10cc38bdc6b5a13d1ae107b0b9	fig|340186.3.peg.3779	Aminomethyltransferase (glycine cleavage system T protein) (EC 2.1.2.10)
84044b10cc38bdc6b5a13d1ae107b0b9	fig|340186.5.peg.3968	Aminomethyltransferase (glycine cleavage system T protein) (EC 2.1.2.10)
84044b10cc38bdc6b5a13d1ae107b0b9	fig|344601.3.peg.621	Aminomethyltransferase (glycine cleavage system T protein) (EC 2.1.2.10)
84044b10cc38bdc6b5a13d1ae107b0b9	fig|344601.5.peg.634	Aminomethyltransferase (glycine cleavage system T protein) (EC 2.1.2.10)
84044b10cc38bdc6b5a13d1ae107b0b9	fig|358709.5.peg.1411	Aminomethyltransferase (glycine cleavage system T protein) (EC 2.1.2.10)
84044b10cc38bdc6b5a13d1ae107b0b9	fig|409438.11.peg.3339	Aminomethyltransferase (glycine cleavage system T protein) (EC 2.1.2.10)
84044b10cc38bdc6b5a13d1ae107b0b9	fig|413997.3.peg.2871	Aminomethyltransferase (glycine cleavage system T protein) (EC 2.1.2.10)
84044b10cc38bdc6b5a13d1ae107b0b9	fig|457401.3.peg.2633	Aminomethyltransferase (glycine cleavage system T protein) (EC 2.1.2.10)
84044b10cc38bdc6b5a13d1ae107b0b9	fig|469008.4.peg.849	Aminomethyltransferase (glycine cleavage system T protein) (EC 2.1.2.10)
84044b10cc38bdc6b5a13d1ae107b0b9	fig|481805.3.peg.856	Aminomethyltransferase (glycine cleavage system T protein) (EC 2.1.2.10)
84044b10cc38bdc6b5a13d1ae107b0b9	fig|481805.6.peg.852	Aminomethyltransferase (glycine cleavage system T protein) (EC 2.1.2.10)
84044b10cc38bdc6b5a13d1ae107b0b9	fig|511145.12.peg.3000	Aminomethyltransferase (glycine cleavage system T protein) (EC 2.1.2.10)
84044b10cc38bdc6b5a13d1ae107b0b9	fig|511145.6.peg.2985	Aminomethyltransferase (glycine cleavage system T protein) (EC 2.1.2.10)
84044b10cc38bdc6b5a13d1ae107b0b9	fig|511693.5.peg.2891	Aminomethyltransferase (glycine cleavage system T protein) (EC 2.1.2.10)
84044b10cc38bdc6b5a13d1ae107b0b9	fig|536056.3.peg.826	Aminomethyltransferase (glycine cleavage system T protein) (EC 2.1.2.10)
84044b10cc38bdc6b5a13d1ae107b0b9	fig|556266.3.peg.1698	Aminomethyltransferase (glycine cleavage system T protein) (EC 2.1.2.10)
84044b10cc38bdc6b5a13d1ae107b0b9	fig|562.375.peg.1274	Aminomethyltransferase (glycine cleavage system T protein) (EC 2.1.2.10)
84044b10cc38bdc6b5a13d1ae107b0b9	fig|566546.3.peg.731	Aminomethyltransferase (glycine cleavage system T protein) (EC 2.1.2.10)
84044b10cc38bdc6b5a13d1ae107b0b9	fig|566546.4.peg.3121	Aminomethyltransferase (glycine cleavage system T protein) (EC 2.1.2.10)
84044b10cc38bdc6b5a13d1ae107b0b9	fig|573235.3.peg.4081	Aminomethyltransferase (glycine cleavage system T protein) (EC 2.1.2.10)
84044b10cc38bdc6b5a13d1ae107b0b9	fig|585034.4.peg.2966	Aminomethyltransferase (glycine cleavage system T protein) (EC 2.1.2.10)
84044b10cc38bdc6b5a13d1ae107b0b9	fig|585034.5.peg.2964	Aminomethyltransferase (glycine cleavage system T protein) (EC 2.1.2.10)
84044b10cc38bdc6b5a13d1ae107b0b9	fig|585055.6.peg.3228	Aminomethyltransferase (glycine cleavage system T protein) (EC 2.1.2.10)
84044b10cc38bdc6b5a13d1ae107b0b9	fig|585055.8.peg.3231	Aminomethyltransferase (glycine cleavage system T protein) (EC 2.1.2.10)
84044b10cc38bdc6b5a13d1ae107b0b9	fig|585395.4.peg.3635	Aminomethyltransferase (glycine cleavage system T protein) (EC 2.1.2.10)
84044b10cc38bdc6b5a13d1ae107b0b9	fig|585396.4.peg.3765	Aminomethyltransferase (glycine cleavage system T protein) (EC 2.1.2.10)
84044b10cc38bdc6b5a13d1ae107b0b9	fig|595495.4.peg.1314	Aminomethyltransferase (glycine cleavage system T protein) (EC 2.1.2.10)
84044b10cc38bdc6b5a13d1ae107b0b9	fig|595496.3.peg.2883	Aminomethyltransferase (glycine cleavage system T protein) (EC 2.1.2.10)
84044b10cc38bdc6b5a13d1ae107b0b9	fig|623.7.peg.2574	Aminomethyltransferase (glycine cleavage system T protein) (EC 2.1.2.10)
84044b10cc38bdc6b5a13d1ae107b0b9	fig|656408.3.peg.3206	Aminomethyltransferase (glycine cleavage system T protein) (EC 2.1.2.10)
84044b10cc38bdc6b5a13d1ae107b0b9	fig|656414.3.peg.3350	Aminomethyltransferase (glycine cleavage system T protein) (EC 2.1.2.10)
84044b10cc38bdc6b5a13d1ae107b0b9	fig|656443.3.peg.3684	Aminomethyltransferase (glycine cleavage system T protein) (EC 2.1.2.10)
84044b10cc38bdc6b5a13d1ae107b0b9	fig|670888.3.peg.3376	Aminomethyltransferase (glycine cleavage system T protein) (EC 2.1.2.10)
84044b10cc38bdc6b5a13d1ae107b0b9	fig|679204.3.peg.1130	Aminomethyltransferase (glycine cleavage system T protein) (EC 2.1.2.10)
84044b10cc38bdc6b5a13d1ae107b0b9	fig|679205.4.peg.1586	Aminomethyltransferase (glycine cleavage system T protein) (EC 2.1.2.10)
84044b10cc38bdc6b5a13d1ae107b0b9	fig|679206.4.peg.495	Aminomethyltransferase (glycine cleavage system T protein) (EC 2.1.2.10)
84044b10cc38bdc6b5a13d1ae107b0b9	fig|679207.4.peg.656	Aminomethyltransferase (glycine cleavage system T protein) (EC 2.1.2.10)
84044b10cc38bdc6b5a13d1ae107b0b9	fig|749532.3.peg.2087	Aminomethyltransferase (glycine cleavage system T protein) (EC 2.1.2.10)
84044b10cc38bdc6b5a13d1ae107b0b9	fig|749533.3.peg.4478	Aminomethyltransferase (glycine cleavage system T protein) (EC 2.1.2.10)
84044b10cc38bdc6b5a13d1ae107b0b9	fig|749537.3.peg.4621	Aminomethyltransferase (glycine cleavage system T protein) (EC 2.1.2.10)
84044b10cc38bdc6b5a13d1ae107b0b9	fig|749538.3.peg.1161	Aminomethyltransferase (glycine cleavage system T protein) (EC 2.1.2.10)
84044b10cc38bdc6b5a13d1ae107b0b9	fig|749540.3.peg.408	Aminomethyltransferase (glycine cleavage system T protein) (EC 2.1.2.10)
84044b10cc38bdc6b5a13d1ae107b0b9	fig|749544.3.peg.188	Aminomethyltransferase (glycine cleavage system T protein) (EC 2.1.2.10)
84044b10cc38bdc6b5a13d1ae107b0b9	fig|749545.3.peg.3087	Aminomethyltransferase (glycine cleavage system T protein) (EC 2.1.2.10)
84044b10cc38bdc6b5a13d1ae107b0b9	fig|749547.3.peg.508	Aminomethyltransferase (glycine cleavage system T protein) (EC 2.1.2.10)
84044b10cc38bdc6b5a13d1ae107b0b9	fig|749548.3.peg.3528	Aminomethyltransferase (glycine cleavage system T protein) (EC 2.1.2.10)
84044b10cc38bdc6b5a13d1ae107b0b9	fig|83333.1.peg.2857	Aminomethyltransferase (glycine cleavage system T protein) (EC 2.1.2.10)
3cd76051606de3e464e0f61495ab38f4	fig|5141.1.peg.798	Malate synthase (EC 2.3.3.9)
2266164365ada7a365e89ed88e8dd353	fig|351746.4.peg.2841	4-hydroxy-2-oxovalerate aldolase (EC 4.1.3.-)
2266164365ada7a365e89ed88e8dd353	fig|351746.6.peg.2944	4-hydroxy-2-oxovalerate aldolase (EC 4.1.3.-)
e48d1c1f351c266723f9809664d91861	fig|316385.5.peg.1919	Tartrate dehydrogenase (EC 1.1.1.93) / Tartrate decarboxylase (EC 4.1.1.73) / D-malic enzyme (EC 1.1.1.83)
e48d1c1f351c266723f9809664d91861	fig|316385.7.peg.1964	Tartrate dehydrogenase (EC 1.1.1.93) / Tartrate decarboxylase (EC 4.1.1.73) / D-malic enzyme (EC 1.1.1.83)
e48d1c1f351c266723f9809664d91861	fig|316401.4.peg.2098	Tartrate dehydrogenase (EC 1.1.1.93) / Tartrate decarboxylase (EC 4.1.1.73) / D-malic enzyme (EC 1.1.1.83)
e48d1c1f351c266723f9809664d91861	fig|316407.3.peg.1755	Tartrate dehydrogenase (EC 1.1.1.93) / Tartrate decarboxylase (EC 4.1.1.73) / D-malic enzyme (EC 1.1.1.83)
e48d1c1f351c266723f9809664d91861	fig|331111.12.peg.2304	Tartrate dehydrogenase (EC 1.1.1.93) / Tartrate decarboxylase (EC 4.1.1.73) / D-malic enzyme (EC 1.1.1.83)
e48d1c1f351c266723f9809664d91861	fig|331111.3.peg.4447	Tartrate dehydrogenase (EC 1.1.1.93) / Tartrate decarboxylase (EC 4.1.1.73) / D-malic enzyme (EC 1.1.1.83)
e48d1c1f351c266723f9809664d91861	fig|331112.3.peg.1778	Tartrate dehydrogenase (EC 1.1.1.93) / Tartrate decarboxylase (EC 4.1.1.73) / D-malic enzyme (EC 1.1.1.83)
e48d1c1f351c266723f9809664d91861	fig|331112.6.peg.1848	Tartrate dehydrogenase (EC 1.1.1.93) / Tartrate decarboxylase (EC 4.1.1.73) / D-malic enzyme (EC 1.1.1.83)
e48d1c1f351c266723f9809664d91861	fig|340184.3.peg.3950	Tartrate dehydrogenase (EC 1.1.1.93) / Tartrate decarboxylase (EC 4.1.1.73) / D-malic enzyme (EC 1.1.1.83)
e48d1c1f351c266723f9809664d91861	fig|340184.6.peg.4130	Tartrate dehydrogenase (EC 1.1.1.93) / Tartrate decarboxylase (EC 4.1.1.73) / D-malic enzyme (EC 1.1.1.83)
e48d1c1f351c266723f9809664d91861	fig|340186.3.peg.4509	Tartrate dehydrogenase (EC 1.1.1.93) / Tartrate decarboxylase (EC 4.1.1.73) / D-malic enzyme (EC 1.1.1.83)
e48d1c1f351c266723f9809664d91861	fig|340186.5.peg.4744	Tartrate dehydrogenase (EC 1.1.1.93) / Tartrate decarboxylase (EC 4.1.1.73) / D-malic enzyme (EC 1.1.1.83)
e48d1c1f351c266723f9809664d91861	fig|344610.3.peg.2336	Tartrate dehydrogenase (EC 1.1.1.93) / Tartrate decarboxylase (EC 4.1.1.73) / D-malic enzyme (EC 1.1.1.83)
e48d1c1f351c266723f9809664d91861	fig|344610.7.peg.5465	Tartrate dehydrogenase (EC 1.1.1.93) / Tartrate decarboxylase (EC 4.1.1.73) / D-malic enzyme (EC 1.1.1.83)
e48d1c1f351c266723f9809664d91861	fig|358708.5.peg.3072	Tartrate dehydrogenase (EC 1.1.1.93) / Tartrate decarboxylase (EC 4.1.1.73) / D-malic enzyme (EC 1.1.1.83)
e48d1c1f351c266723f9809664d91861	fig|409438.11.peg.2124	Tartrate dehydrogenase (EC 1.1.1.93) / Tartrate decarboxylase (EC 4.1.1.73) / D-malic enzyme (EC 1.1.1.83)
e48d1c1f351c266723f9809664d91861	fig|457401.3.peg.255	Tartrate dehydrogenase (EC 1.1.1.93) / Tartrate decarboxylase (EC 4.1.1.73) / D-malic enzyme (EC 1.1.1.83)
e48d1c1f351c266723f9809664d91861	fig|481805.3.peg.1976	Tartrate dehydrogenase (EC 1.1.1.93) / Tartrate decarboxylase (EC 4.1.1.73) / D-malic enzyme (EC 1.1.1.83)
e48d1c1f351c266723f9809664d91861	fig|481805.6.peg.1970	Tartrate dehydrogenase (EC 1.1.1.93) / Tartrate decarboxylase (EC 4.1.1.73) / D-malic enzyme (EC 1.1.1.83)
e48d1c1f351c266723f9809664d91861	fig|511145.12.peg.1876	Tartrate dehydrogenase (EC 1.1.1.93) / Tartrate decarboxylase (EC 4.1.1.73) / D-malic enzyme (EC 1.1.1.83)
e48d1c1f351c266723f9809664d91861	fig|511145.6.peg.1860	Tartrate dehydrogenase (EC 1.1.1.93) / Tartrate decarboxylase (EC 4.1.1.73) / D-malic enzyme (EC 1.1.1.83)
e48d1c1f351c266723f9809664d91861	fig|536056.3.peg.1948	Tartrate dehydrogenase (EC 1.1.1.93) / Tartrate decarboxylase (EC 4.1.1.73) / D-malic enzyme (EC 1.1.1.83)
e48d1c1f351c266723f9809664d91861	fig|550672.3.peg.1530	Tartrate dehydrogenase (EC 1.1.1.93) / Tartrate decarboxylase (EC 4.1.1.73) / D-malic enzyme (EC 1.1.1.83)
e48d1c1f351c266723f9809664d91861	fig|550677.3.peg.1286	Tartrate dehydrogenase (EC 1.1.1.93) / Tartrate decarboxylase (EC 4.1.1.73) / D-malic enzyme (EC 1.1.1.83)
e48d1c1f351c266723f9809664d91861	fig|562.375.peg.1915	Tartrate dehydrogenase (EC 1.1.1.93) / Tartrate decarboxylase (EC 4.1.1.73) / D-malic enzyme (EC 1.1.1.83)
e48d1c1f351c266723f9809664d91861	fig|566546.3.peg.4035	Tartrate dehydrogenase (EC 1.1.1.93) / Tartrate decarboxylase (EC 4.1.1.73) / D-malic enzyme (EC 1.1.1.83)
e48d1c1f351c266723f9809664d91861	fig|566546.4.peg.1963	Tartrate dehydrogenase (EC 1.1.1.93) / Tartrate decarboxylase (EC 4.1.1.73) / D-malic enzyme (EC 1.1.1.83)
e48d1c1f351c266723f9809664d91861	fig|573235.3.peg.2612	Tartrate dehydrogenase (EC 1.1.1.93) / Tartrate decarboxylase (EC 4.1.1.73) / D-malic enzyme (EC 1.1.1.83)
e48d1c1f351c266723f9809664d91861	fig|585055.6.peg.2003	Tartrate dehydrogenase (EC 1.1.1.93) / Tartrate decarboxylase (EC 4.1.1.73) / D-malic enzyme (EC 1.1.1.83)
e48d1c1f351c266723f9809664d91861	fig|585055.8.peg.2006	Tartrate dehydrogenase (EC 1.1.1.93) / Tartrate decarboxylase (EC 4.1.1.73) / D-malic enzyme (EC 1.1.1.83)
e48d1c1f351c266723f9809664d91861	fig|585396.4.peg.2396	Tartrate dehydrogenase (EC 1.1.1.93) / Tartrate decarboxylase (EC 4.1.1.73) / D-malic enzyme (EC 1.1.1.83)
e48d1c1f351c266723f9809664d91861	fig|595495.4.peg.3858	Tartrate dehydrogenase (EC 1.1.1.93) / Tartrate decarboxylase (EC 4.1.1.73) / D-malic enzyme (EC 1.1.1.83)
e48d1c1f351c266723f9809664d91861	fig|595496.3.peg.1766	Tartrate dehydrogenase (EC 1.1.1.93) / Tartrate decarboxylase (EC 4.1.1.73) / D-malic enzyme (EC 1.1.1.83)
e48d1c1f351c266723f9809664d91861	fig|656408.3.peg.1966	Tartrate dehydrogenase (EC 1.1.1.93) / Tartrate decarboxylase (EC 4.1.1.73) / D-malic enzyme (EC 1.1.1.83)
e48d1c1f351c266723f9809664d91861	fig|656414.3.peg.2133	Tartrate dehydrogenase (EC 1.1.1.93) / Tartrate decarboxylase (EC 4.1.1.73) / D-malic enzyme (EC 1.1.1.83)
e48d1c1f351c266723f9809664d91861	fig|656444.3.peg.2723	Tartrate dehydrogenase (EC 1.1.1.93) / Tartrate decarboxylase (EC 4.1.1.73) / D-malic enzyme (EC 1.1.1.83)
e48d1c1f351c266723f9809664d91861	fig|6666666.5522.peg.3748	Tartrate dehydrogenase (EC 1.1.1.93) / Tartrate decarboxylase (EC 4.1.1.73) / D-malic enzyme (EC 1.1.1.83)
e48d1c1f351c266723f9809664d91861	fig|670888.3.peg.313	Tartrate dehydrogenase (EC 1.1.1.93) / Tartrate decarboxylase (EC 4.1.1.73) / D-malic enzyme (EC 1.1.1.83)
e48d1c1f351c266723f9809664d91861	fig|679205.4.peg.4355	Tartrate dehydrogenase (EC 1.1.1.93) / Tartrate decarboxylase (EC 4.1.1.73) / D-malic enzyme (EC 1.1.1.83)
e48d1c1f351c266723f9809664d91861	fig|679206.4.peg.2926	Tartrate dehydrogenase (EC 1.1.1.93) / Tartrate decarboxylase (EC 4.1.1.73) / D-malic enzyme (EC 1.1.1.83)
e48d1c1f351c266723f9809664d91861	fig|679207.4.peg.1323	Tartrate dehydrogenase (EC 1.1.1.93) / Tartrate decarboxylase (EC 4.1.1.73) / D-malic enzyme (EC 1.1.1.83)
e48d1c1f351c266723f9809664d91861	fig|749533.3.peg.2766	Tartrate dehydrogenase (EC 1.1.1.93) / Tartrate decarboxylase (EC 4.1.1.73) / D-malic enzyme (EC 1.1.1.83)
e48d1c1f351c266723f9809664d91861	fig|749538.3.peg.4411	Tartrate dehydrogenase (EC 1.1.1.93) / Tartrate decarboxylase (EC 4.1.1.73) / D-malic enzyme (EC 1.1.1.83)
e48d1c1f351c266723f9809664d91861	fig|749540.3.peg.2207	Tartrate dehydrogenase (EC 1.1.1.93) / Tartrate decarboxylase (EC 4.1.1.73) / D-malic enzyme (EC 1.1.1.83)
e48d1c1f351c266723f9809664d91861	fig|749544.3.peg.2277	Tartrate dehydrogenase (EC 1.1.1.93) / Tartrate decarboxylase (EC 4.1.1.73) / D-malic enzyme (EC 1.1.1.83)
e48d1c1f351c266723f9809664d91861	fig|749545.3.peg.2847	Tartrate dehydrogenase (EC 1.1.1.93) / Tartrate decarboxylase (EC 4.1.1.73) / D-malic enzyme (EC 1.1.1.83)
e48d1c1f351c266723f9809664d91861	fig|749548.3.peg.2539	Tartrate dehydrogenase (EC 1.1.1.93) / Tartrate decarboxylase (EC 4.1.1.73) / D-malic enzyme (EC 1.1.1.83)
e48d1c1f351c266723f9809664d91861	fig|83333.1.peg.1783	Tartrate dehydrogenase (EC 1.1.1.93) / Tartrate decarboxylase (EC 4.1.1.73) / D-malic enzyme (EC 1.1.1.83)
ade04a94dce1d4507f8c626bc12c19c3	fig|262724.1.peg.1666	Cytochrome c oxidase polypeptide II (EC 1.9.3.1)
ade04a94dce1d4507f8c626bc12c19c3	fig|262724.6.peg.1669	Cytochrome c oxidase polypeptide II (EC 1.9.3.1)
0762870914ef40373e4e093826019e4b	fig|209261.1.peg.483	Histidine ABC transporter, permease protein HisQ (TC 3.A.1.3.1)
0762870914ef40373e4e093826019e4b	fig|209261.6.peg.558	Histidine ABC transporter, permease protein HisQ (TC 3.A.1.3.1)
0762870914ef40373e4e093826019e4b	fig|216597.6.peg.2582	Histidine ABC transporter, permease protein HisQ (TC 3.A.1.3.1)
0762870914ef40373e4e093826019e4b	fig|220341.1.peg.2285	Histidine ABC transporter, permease protein HisQ (TC 3.A.1.3.1)
0762870914ef40373e4e093826019e4b	fig|220341.7.peg.2615	Histidine ABC transporter, permease protein HisQ (TC 3.A.1.3.1)
0762870914ef40373e4e093826019e4b	fig|272994.5.peg.524	Histidine ABC transporter, permease protein HisQ (TC 3.A.1.3.1)
0762870914ef40373e4e093826019e4b	fig|28901.42.peg.2345	Histidine ABC transporter, permease protein HisQ (TC 3.A.1.3.1)
0762870914ef40373e4e093826019e4b	fig|295319.15.peg.544	Histidine ABC transporter, permease protein HisQ (TC 3.A.1.3.1)
0762870914ef40373e4e093826019e4b	fig|295319.3.peg.2108	Histidine ABC transporter, permease protein HisQ (TC 3.A.1.3.1)
0762870914ef40373e4e093826019e4b	fig|321314.4.peg.2820	Histidine ABC transporter, permease protein HisQ (TC 3.A.1.3.1)
0762870914ef40373e4e093826019e4b	fig|321314.9.peg.2823	Histidine ABC transporter, permease protein HisQ (TC 3.A.1.3.1)
0762870914ef40373e4e093826019e4b	fig|423368.6.peg.2646	Histidine ABC transporter, permease protein HisQ (TC 3.A.1.3.1)
0762870914ef40373e4e093826019e4b	fig|423368.8.peg.2620	Histidine ABC transporter, permease protein HisQ (TC 3.A.1.3.1)
0762870914ef40373e4e093826019e4b	fig|439843.6.peg.2627	Histidine ABC transporter, permease protein HisQ (TC 3.A.1.3.1)
0762870914ef40373e4e093826019e4b	fig|439843.8.peg.2639	Histidine ABC transporter, permease protein HisQ (TC 3.A.1.3.1)
0762870914ef40373e4e093826019e4b	fig|439846.4.peg.2899	Histidine ABC transporter, permease protein HisQ (TC 3.A.1.3.1)
0762870914ef40373e4e093826019e4b	fig|439851.5.peg.2724	Histidine ABC transporter, permease protein HisQ (TC 3.A.1.3.1)
0762870914ef40373e4e093826019e4b	fig|439851.8.peg.2700	Histidine ABC transporter, permease protein HisQ (TC 3.A.1.3.1)
0762870914ef40373e4e093826019e4b	fig|440534.5.peg.2871	Histidine ABC transporter, permease protein HisQ (TC 3.A.1.3.1)
0762870914ef40373e4e093826019e4b	fig|454164.6.peg.2187	Histidine ABC transporter, permease protein HisQ (TC 3.A.1.3.1)
0762870914ef40373e4e093826019e4b	fig|454165.5.peg.2530	Histidine ABC transporter, permease protein HisQ (TC 3.A.1.3.1)
0762870914ef40373e4e093826019e4b	fig|454167.5.peg.2683	Histidine ABC transporter, permease protein HisQ (TC 3.A.1.3.1)
0762870914ef40373e4e093826019e4b	fig|454168.5.peg.3022	Histidine ABC transporter, permease protein HisQ (TC 3.A.1.3.1)
0762870914ef40373e4e093826019e4b	fig|454169.6.peg.2615	Histidine ABC transporter, permease protein HisQ (TC 3.A.1.3.1)
0762870914ef40373e4e093826019e4b	fig|465516.5.peg.1935	Histidine ABC transporter, permease protein HisQ (TC 3.A.1.3.1)
0762870914ef40373e4e093826019e4b	fig|465517.10.peg.2581	Histidine ABC transporter, permease protein HisQ (TC 3.A.1.3.1)
0762870914ef40373e4e093826019e4b	fig|496064.4.peg.3558	Histidine ABC transporter, permease protein HisQ (TC 3.A.1.3.1)
0762870914ef40373e4e093826019e4b	fig|497974.4.peg.3299	Histidine ABC transporter, permease protein HisQ (TC 3.A.1.3.1)
0762870914ef40373e4e093826019e4b	fig|550537.3.peg.2483	Histidine ABC transporter, permease protein HisQ (TC 3.A.1.3.1)
0762870914ef40373e4e093826019e4b	fig|554290.7.peg.532	Histidine ABC transporter, permease protein HisQ (TC 3.A.1.3.1)
0762870914ef40373e4e093826019e4b	fig|554290.9.peg.545	Histidine ABC transporter, permease protein HisQ (TC 3.A.1.3.1)
0762870914ef40373e4e093826019e4b	fig|568708.3.peg.2514	Histidine ABC transporter, permease protein HisQ (TC 3.A.1.3.1)
0762870914ef40373e4e093826019e4b	fig|573395.3.peg.3013	Histidine ABC transporter, permease protein HisQ (TC 3.A.1.3.1)
0762870914ef40373e4e093826019e4b	fig|588858.6.peg.2695	Histidine ABC transporter, permease protein HisQ (TC 3.A.1.3.1)
0762870914ef40373e4e093826019e4b	fig|99287.12.peg.2490	Histidine ABC transporter, permease protein HisQ (TC 3.A.1.3.1)
0762870914ef40373e4e093826019e4b	fig|99287.1.peg.2276	Histidine ABC transporter, permease protein HisQ (TC 3.A.1.3.1)
70563109a451dc689384814cec661e65	fig|155864.1.peg.941	Arginine ABC transporter, ATP-binding protein ArtP
70563109a451dc689384814cec661e65	fig|155864.8.peg.971	Arginine ABC transporter, ATP-binding protein ArtP
70563109a451dc689384814cec661e65	fig|198214.7.peg.947	Arginine ABC transporter, ATP-binding protein ArtP
70563109a451dc689384814cec661e65	fig|198215.1.peg.775	Arginine ABC transporter, ATP-binding protein ArtP
70563109a451dc689384814cec661e65	fig|198215.6.peg.928	Arginine ABC transporter, ATP-binding protein ArtP
70563109a451dc689384814cec661e65	fig|199310.4.peg.950	Arginine ABC transporter, ATP-binding protein ArtP
70563109a451dc689384814cec661e65	fig|216592.3.peg.986	Arginine ABC transporter, ATP-binding protein ArtP
70563109a451dc689384814cec661e65	fig|300267.13.peg.2901	Arginine ABC transporter, ATP-binding protein ArtP
70563109a451dc689384814cec661e65	fig|300268.10.peg.1081	Arginine ABC transporter, ATP-binding protein ArtP
70563109a451dc689384814cec661e65	fig|300268.11.peg.1094	Arginine ABC transporter, ATP-binding protein ArtP
70563109a451dc689384814cec661e65	fig|300269.11.peg.957	Arginine ABC transporter, ATP-binding protein ArtP
70563109a451dc689384814cec661e65	fig|300269.12.peg.989	Arginine ABC transporter, ATP-binding protein ArtP
70563109a451dc689384814cec661e65	fig|316385.5.peg.931	Arginine ABC transporter, ATP-binding protein ArtP
70563109a451dc689384814cec661e65	fig|316385.7.peg.944	Arginine ABC transporter, ATP-binding protein ArtP
70563109a451dc689384814cec661e65	fig|316401.4.peg.1076	Arginine ABC transporter, ATP-binding protein ArtP
70563109a451dc689384814cec661e65	fig|316407.3.peg.831	Arginine ABC transporter, ATP-binding protein ArtP
70563109a451dc689384814cec661e65	fig|331111.12.peg.1228	Arginine ABC transporter, ATP-binding protein ArtP
70563109a451dc689384814cec661e65	fig|331111.3.peg.3434	Arginine ABC transporter, ATP-binding protein ArtP
70563109a451dc689384814cec661e65	fig|331112.3.peg.897	Arginine ABC transporter, ATP-binding protein ArtP
70563109a451dc689384814cec661e65	fig|331112.6.peg.936	Arginine ABC transporter, ATP-binding protein ArtP
70563109a451dc689384814cec661e65	fig|340185.3.peg.2790	Arginine ABC transporter, ATP-binding protein ArtP
70563109a451dc689384814cec661e65	fig|340185.4.peg.2940	Arginine ABC transporter, ATP-binding protein ArtP
70563109a451dc689384814cec661e65	fig|340186.3.peg.2520	Arginine ABC transporter, ATP-binding protein ArtP
70563109a451dc689384814cec661e65	fig|340186.5.peg.2604	Arginine ABC transporter, ATP-binding protein ArtP
70563109a451dc689384814cec661e65	fig|340197.3.peg.2155	Arginine ABC transporter, ATP-binding protein ArtP
70563109a451dc689384814cec661e65	fig|340197.5.peg.2269	Arginine ABC transporter, ATP-binding protein ArtP
70563109a451dc689384814cec661e65	fig|344601.3.peg.2470	Arginine ABC transporter, ATP-binding protein ArtP
70563109a451dc689384814cec661e65	fig|344601.5.peg.2573	Arginine ABC transporter, ATP-binding protein ArtP
70563109a451dc689384814cec661e65	fig|344609.11.peg.2730	Arginine ABC transporter, ATP-binding protein ArtP
70563109a451dc689384814cec661e65	fig|344609.3.peg.1841	Arginine ABC transporter, ATP-binding protein ArtP
70563109a451dc689384814cec661e65	fig|344610.3.peg.3303	Arginine ABC transporter, ATP-binding protein ArtP
70563109a451dc689384814cec661e65	fig|358708.5.peg.2725	Arginine ABC transporter, ATP-binding protein ArtP
70563109a451dc689384814cec661e65	fig|362663.8.peg.887	Arginine ABC transporter, ATP-binding protein ArtP
70563109a451dc689384814cec661e65	fig|362663.9.peg.887	Arginine ABC transporter, ATP-binding protein ArtP
70563109a451dc689384814cec661e65	fig|364106.7.peg.954	Arginine ABC transporter, ATP-binding protein ArtP
70563109a451dc689384814cec661e65	fig|364106.8.peg.953	Arginine ABC transporter, ATP-binding protein ArtP
70563109a451dc689384814cec661e65	fig|373384.10.peg.953	Arginine ABC transporter, ATP-binding protein ArtP
70563109a451dc689384814cec661e65	fig|373384.11.peg.963	Arginine ABC transporter, ATP-binding protein ArtP
70563109a451dc689384814cec661e65	fig|386585.9.peg.1065	Arginine ABC transporter, ATP-binding protein ArtP
70563109a451dc689384814cec661e65	fig|405955.9.peg.696	Arginine ABC transporter, ATP-binding protein ArtP
70563109a451dc689384814cec661e65	fig|409438.11.peg.1052	Arginine ABC transporter, ATP-binding protein ArtP
70563109a451dc689384814cec661e65	fig|413997.3.peg.910	Arginine ABC transporter, ATP-binding protein ArtP
70563109a451dc689384814cec661e65	fig|431946.3.peg.827	Arginine ABC transporter, ATP-binding protein ArtP
70563109a451dc689384814cec661e65	fig|439855.10.peg.1049	Arginine ABC transporter, ATP-binding protein ArtP
70563109a451dc689384814cec661e65	fig|444447.5.peg.3849	Arginine ABC transporter, ATP-binding protein ArtP
70563109a451dc689384814cec661e65	fig|444448.5.peg.3653	Arginine ABC transporter, ATP-binding protein ArtP
70563109a451dc689384814cec661e65	fig|444449.5.peg.4376	Arginine ABC transporter, ATP-binding protein ArtP
70563109a451dc689384814cec661e65	fig|444450.8.peg.1110	Arginine ABC transporter, ATP-binding protein ArtP
70563109a451dc689384814cec661e65	fig|444451.5.peg.3923	Arginine ABC transporter, ATP-binding protein ArtP
70563109a451dc689384814cec661e65	fig|444452.5.peg.3038	Arginine ABC transporter, ATP-binding protein ArtP
70563109a451dc689384814cec661e65	fig|444453.5.peg.993	Arginine ABC transporter, ATP-binding protein ArtP
70563109a451dc689384814cec661e65	fig|444454.5.peg.5443	Arginine ABC transporter, ATP-binding protein ArtP
70563109a451dc689384814cec661e65	fig|457401.3.peg.1416	Arginine ABC transporter, ATP-binding protein ArtP
70563109a451dc689384814cec661e65	fig|469598.5.peg.151	Arginine ABC transporter, ATP-binding protein ArtP
70563109a451dc689384814cec661e65	fig|478004.5.peg.3370	Arginine ABC transporter, ATP-binding protein ArtP
70563109a451dc689384814cec661e65	fig|478005.5.peg.1492	Arginine ABC transporter, ATP-binding protein ArtP
70563109a451dc689384814cec661e65	fig|478006.5.peg.3681	Arginine ABC transporter, ATP-binding protein ArtP
70563109a451dc689384814cec661e65	fig|478007.5.peg.2561	Arginine ABC transporter, ATP-binding protein ArtP
70563109a451dc689384814cec661e65	fig|478008.5.peg.2344	Arginine ABC transporter, ATP-binding protein ArtP
70563109a451dc689384814cec661e65	fig|481805.3.peg.2936	Arginine ABC transporter, ATP-binding protein ArtP
70563109a451dc689384814cec661e65	fig|481805.6.peg.2926	Arginine ABC transporter, ATP-binding protein ArtP
70563109a451dc689384814cec661e65	fig|502346.5.peg.240	Arginine ABC transporter, ATP-binding protein ArtP
70563109a451dc689384814cec661e65	fig|511145.12.peg.893	Arginine ABC transporter, ATP-binding protein ArtP
70563109a451dc689384814cec661e65	fig|511145.6.peg.886	Arginine ABC transporter, ATP-binding protein ArtP
70563109a451dc689384814cec661e65	fig|525281.3.peg.2088	Arginine ABC transporter, ATP-binding protein ArtP
70563109a451dc689384814cec661e65	fig|536056.3.peg.2932	Arginine ABC transporter, ATP-binding protein ArtP
70563109a451dc689384814cec661e65	fig|544404.4.peg.976	Arginine ABC transporter, ATP-binding protein ArtP
70563109a451dc689384814cec661e65	fig|550672.3.peg.1139	Arginine ABC transporter, ATP-binding protein ArtP
70563109a451dc689384814cec661e65	fig|550676.3.peg.331	Arginine ABC transporter, ATP-binding protein ArtP
70563109a451dc689384814cec661e65	fig|550677.3.peg.2136	Arginine ABC transporter, ATP-binding protein ArtP
70563109a451dc689384814cec661e65	fig|556266.3.peg.3865	Arginine ABC transporter, ATP-binding protein ArtP
70563109a451dc689384814cec661e65	fig|562.371.peg.2176	Arginine ABC transporter, ATP-binding protein ArtP
70563109a451dc689384814cec661e65	fig|562.372.peg.1023	Arginine ABC transporter, ATP-binding protein ArtP
70563109a451dc689384814cec661e65	fig|562.374.peg.2557	Arginine ABC transporter, ATP-binding protein ArtP
70563109a451dc689384814cec661e65	fig|562.375.peg.4512	Arginine ABC transporter, ATP-binding protein ArtP
70563109a451dc689384814cec661e65	fig|562.376.peg.4738	Arginine ABC transporter, ATP-binding protein ArtP
70563109a451dc689384814cec661e65	fig|566546.3.peg.1914	Arginine ABC transporter, ATP-binding protein ArtP
70563109a451dc689384814cec661e65	fig|573235.3.peg.1012	Arginine ABC transporter, ATP-binding protein ArtP
70563109a451dc689384814cec661e65	fig|574521.7.peg.882	Arginine ABC transporter, ATP-binding protein ArtP
70563109a451dc689384814cec661e65	fig|585034.4.peg.893	Arginine ABC transporter, ATP-binding protein ArtP
70563109a451dc689384814cec661e65	fig|585034.5.peg.889	Arginine ABC transporter, ATP-binding protein ArtP
70563109a451dc689384814cec661e65	fig|585035.6.peg.885	Arginine ABC transporter, ATP-binding protein ArtP
70563109a451dc689384814cec661e65	fig|585055.6.peg.923	Arginine ABC transporter, ATP-binding protein ArtP
70563109a451dc689384814cec661e65	fig|585055.8.peg.926	Arginine ABC transporter, ATP-binding protein ArtP
70563109a451dc689384814cec661e65	fig|585057.4.peg.894	Arginine ABC transporter, ATP-binding protein ArtP
70563109a451dc689384814cec661e65	fig|585057.6.peg.890	Arginine ABC transporter, ATP-binding protein ArtP
70563109a451dc689384814cec661e65	fig|585396.4.peg.970	Arginine ABC transporter, ATP-binding protein ArtP
70563109a451dc689384814cec661e65	fig|585397.7.peg.839	Arginine ABC transporter, ATP-binding protein ArtP
70563109a451dc689384814cec661e65	fig|585397.9.peg.839	Arginine ABC transporter, ATP-binding protein ArtP
70563109a451dc689384814cec661e65	fig|591020.3.peg.950	Arginine ABC transporter, ATP-binding protein ArtP
70563109a451dc689384814cec661e65	fig|595496.3.peg.791	Arginine ABC transporter, ATP-binding protein ArtP
70563109a451dc689384814cec661e65	fig|621.8.peg.5246	Arginine ABC transporter, ATP-binding protein ArtP
70563109a451dc689384814cec661e65	fig|622.8.peg.1979	Arginine ABC transporter, ATP-binding protein ArtP
70563109a451dc689384814cec661e65	fig|623.7.peg.3991	Arginine ABC transporter, ATP-binding protein ArtP
70563109a451dc689384814cec661e65	fig|655817.3.peg.953	Arginine ABC transporter, ATP-binding protein ArtP
70563109a451dc689384814cec661e65	fig|656379.3.peg.1712	Arginine ABC transporter, ATP-binding protein ArtP
70563109a451dc689384814cec661e65	fig|656393.3.peg.1624	Arginine ABC transporter, ATP-binding protein ArtP
70563109a451dc689384814cec661e65	fig|656408.3.peg.921	Arginine ABC transporter, ATP-binding protein ArtP
70563109a451dc689384814cec661e65	fig|656414.3.peg.1070	Arginine ABC transporter, ATP-binding protein ArtP
70563109a451dc689384814cec661e65	fig|656417.3.peg.1102	Arginine ABC transporter, ATP-binding protein ArtP
70563109a451dc689384814cec661e65	fig|656419.3.peg.1157	Arginine ABC transporter, ATP-binding protein ArtP
70563109a451dc689384814cec661e65	fig|656437.3.peg.997	Arginine ABC transporter, ATP-binding protein ArtP
70563109a451dc689384814cec661e65	fig|6666666.5522.peg.1454	Arginine ABC transporter, ATP-binding protein ArtP
70563109a451dc689384814cec661e65	fig|679204.3.peg.2474	Arginine ABC transporter, ATP-binding protein ArtP
70563109a451dc689384814cec661e65	fig|679205.4.peg.3843	Arginine ABC transporter, ATP-binding protein ArtP
70563109a451dc689384814cec661e65	fig|679206.4.peg.3770	Arginine ABC transporter, ATP-binding protein ArtP
70563109a451dc689384814cec661e65	fig|685038.3.peg.790	Arginine ABC transporter, ATP-binding protein ArtP
70563109a451dc689384814cec661e65	fig|701177.3.peg.1080	Arginine ABC transporter, ATP-binding protein ArtP
70563109a451dc689384814cec661e65	fig|714962.3.peg.873	Arginine ABC transporter, ATP-binding protein ArtP
70563109a451dc689384814cec661e65	fig|749527.3.peg.113	Arginine ABC transporter, ATP-binding protein ArtP
70563109a451dc689384814cec661e65	fig|749528.3.peg.4606	Arginine ABC transporter, ATP-binding protein ArtP
70563109a451dc689384814cec661e65	fig|749532.3.peg.860	Arginine ABC transporter, ATP-binding protein ArtP
70563109a451dc689384814cec661e65	fig|749533.3.peg.533	Arginine ABC transporter, ATP-binding protein ArtP
70563109a451dc689384814cec661e65	fig|749537.3.peg.4370	Arginine ABC transporter, ATP-binding protein ArtP
70563109a451dc689384814cec661e65	fig|749538.3.peg.619	Arginine ABC transporter, ATP-binding protein ArtP
70563109a451dc689384814cec661e65	fig|749540.3.peg.2806	Arginine ABC transporter, ATP-binding protein ArtP
70563109a451dc689384814cec661e65	fig|749544.3.peg.2752	Arginine ABC transporter, ATP-binding protein ArtP
70563109a451dc689384814cec661e65	fig|749546.3.peg.4894	Arginine ABC transporter, ATP-binding protein ArtP
70563109a451dc689384814cec661e65	fig|749547.3.peg.1580	Arginine ABC transporter, ATP-binding protein ArtP
70563109a451dc689384814cec661e65	fig|749548.3.peg.3370	Arginine ABC transporter, ATP-binding protein ArtP
70563109a451dc689384814cec661e65	fig|753642.3.peg.749	Arginine ABC transporter, ATP-binding protein ArtP
70563109a451dc689384814cec661e65	fig|83333.1.peg.850	Arginine ABC transporter, ATP-binding protein ArtP
70563109a451dc689384814cec661e65	fig|83334.1.peg.1015	Arginine ABC transporter, ATP-binding protein ArtP
78261d16ff6656ac479f4550b2ddd7e2	fig|316385.5.peg.3256	D-galactarate dehydratase (EC 4.2.1.42)
78261d16ff6656ac479f4550b2ddd7e2	fig|316385.7.peg.3326	D-galactarate dehydratase (EC 4.2.1.42)
78261d16ff6656ac479f4550b2ddd7e2	fig|316401.4.peg.3860	D-galactarate dehydratase (EC 4.2.1.42)
78261d16ff6656ac479f4550b2ddd7e2	fig|316407.3.peg.3010	D-galactarate dehydratase (EC 4.2.1.42)
78261d16ff6656ac479f4550b2ddd7e2	fig|457400.3.peg.1080	D-galactarate dehydratase (EC 4.2.1.42)
78261d16ff6656ac479f4550b2ddd7e2	fig|457401.3.peg.3061	D-galactarate dehydratase (EC 4.2.1.42)
78261d16ff6656ac479f4550b2ddd7e2	fig|511145.12.peg.3222	D-galactarate dehydratase (EC 4.2.1.42)
78261d16ff6656ac479f4550b2ddd7e2	fig|511145.6.peg.3207	D-galactarate dehydratase (EC 4.2.1.42)
78261d16ff6656ac479f4550b2ddd7e2	fig|595496.3.peg.3106	D-galactarate dehydratase (EC 4.2.1.42)
78261d16ff6656ac479f4550b2ddd7e2	fig|656414.3.peg.3600	D-galactarate dehydratase (EC 4.2.1.42)
78261d16ff6656ac479f4550b2ddd7e2	fig|670888.3.peg.3123	D-galactarate dehydratase (EC 4.2.1.42)
78261d16ff6656ac479f4550b2ddd7e2	fig|749537.3.peg.502	D-galactarate dehydratase (EC 4.2.1.42)
78261d16ff6656ac479f4550b2ddd7e2	fig|749540.3.peg.1413	D-galactarate dehydratase (EC 4.2.1.42)
78261d16ff6656ac479f4550b2ddd7e2	fig|749548.3.peg.1139	D-galactarate dehydratase (EC 4.2.1.42)
78261d16ff6656ac479f4550b2ddd7e2	fig|83333.1.peg.3073	D-galactarate dehydratase (EC 4.2.1.42)
9b97d84d0c1be9af2b2da2212f59b622	fig|272620.9.peg.524	PTS system, sucrose-specific IIB component (EC 2.7.1.69) / PTS system, sucrose-specific IIC component (EC 2.7.1.69)
9b97d84d0c1be9af2b2da2212f59b622	fig|484021.4.peg.1243	PTS system, sucrose-specific IIB component (EC 2.7.1.69) / PTS system, sucrose-specific IIC component (EC 2.7.1.69)
accc3df3d3e132f71faf40e952ee93e3	fig|416870.7.peg.1538	Ribonucleotide reduction protein NrdI
accc3df3d3e132f71faf40e952ee93e3	fig|416870.9.peg.1579	Ribonucleotide reduction protein NrdI
accc3df3d3e132f71faf40e952ee93e3	fig|746361.3.peg.1595	Ribonucleotide reduction protein NrdI
ba9cb03bc99686ab9293c29d1d43315e	fig|316385.7.peg.4307	Proton/glutamate symport protein @ Proton/aspartate symport protein
ba9cb03bc99686ab9293c29d1d43315e	fig|316407.3.peg.3914	Proton/glutamate symport protein @ Proton/aspartate symport protein
ba9cb03bc99686ab9293c29d1d43315e	fig|511145.12.peg.4201	Proton/glutamate symport protein @ Proton/aspartate symport protein
ba9cb03bc99686ab9293c29d1d43315e	fig|511145.6.peg.4183	Proton/glutamate symport protein @ Proton/aspartate symport protein
ba9cb03bc99686ab9293c29d1d43315e	fig|536056.3.peg.4158	Proton/glutamate symport protein @ Proton/aspartate symport protein
ba9cb03bc99686ab9293c29d1d43315e	fig|595495.4.peg.3118	Proton/glutamate symport protein @ Proton/aspartate symport protein
ba9cb03bc99686ab9293c29d1d43315e	fig|595496.3.peg.4148	Proton/glutamate symport protein @ Proton/aspartate symport protein
ba9cb03bc99686ab9293c29d1d43315e	fig|83333.1.peg.3987	Proton/glutamate symport protein @ Proton/aspartate symport protein
444f031c1e505e659c0dca6cca7640aa	fig|4932.3.peg.4257	L-asparaginase (EC 3.5.1.1)
444f031c1e505e659c0dca6cca7640aa	fig|4932.3.peg.4260	L-asparaginase (EC 3.5.1.1)
444f031c1e505e659c0dca6cca7640aa	fig|4932.3.peg.4266	L-asparaginase (EC 3.5.1.1)
444f031c1e505e659c0dca6cca7640aa	fig|4932.3.peg.4269	L-asparaginase (EC 3.5.1.1)
444f031c1e505e659c0dca6cca7640aa	fig|559292.3.peg.3714	Cell-wall L-asparaginase II involved in asparagine catabolism; expression induced during nitrogen starvation; ORF contains a short non-coding RNA that enhances expression of full-length gene; reference strain S288C has four copies of ASP3
444f031c1e505e659c0dca6cca7640aa	fig|559292.3.peg.3717	Cell-wall L-asparaginase II involved in asparagine catabolism; expression induced during nitrogen starvation; ORF contains a short non-coding RNA that enhances expression of full-length gene; reference strain S288C has four copies of ASP3
444f031c1e505e659c0dca6cca7640aa	fig|559292.3.peg.3723	Cell-wall L-asparaginase II involved in asparagine catabolism; expression induced during nitrogen starvation; ORF contains a short non-coding RNA that enhances expression of full-length gene; reference strain S288C has four copies of ASP3
444f031c1e505e659c0dca6cca7640aa	fig|559292.3.peg.3726	Cell-wall L-asparaginase II involved in asparagine catabolism; expression induced during nitrogen starvation; ORF contains a short non-coding RNA that enhances expression of full-length gene; reference strain S288C has four copies of ASP3
6e79ecad88759492333652e1ecffa78d	fig|39947.3.peg.59654	Aldehyde dehydrogenase (EC 1.2.1.3)
cb3550bc173b4ca25d8c43b4aa43b171	fig|76869.3.peg.3944	1-pyrroline-4-hydroxy-2-carboxylate deaminase (EC 3.5.4.22) # predicted
cb3550bc173b4ca25d8c43b4aa43b171	fig|76869.5.peg.3979	1-pyrroline-4-hydroxy-2-carboxylate deaminase (EC 3.5.4.22)
b73f65d5a69a1f422ff5c8766c357485	fig|3702.1.peg.19041	identical to SP|P54144 High affinity ammonium transporter (AtAMT1;1) {Arabidopsis thaliana}; go_component: membrane [goid 0016020]; go_function: ammonium transporter activity [goid 0008519]; go_process: transport [goid 0006810] / ammonium transporter 1, member 1 (AMT1.1)
b73f65d5a69a1f422ff5c8766c357485	fig|3702.7.peg.10309	Ammonium transporter
7e636f91be97d7064704004cc69a361c	fig|6239.3.peg.15424	Peptidyl prolyl 4-hydroxylase, alpha subunit (EC 1.14.11.2)
c4b6a4d8d8903c4b80ae1fd78ec2bbd8	fig|292415.3.peg.915	Cytochrome cd1 nitrite reductase (EC:1.7.2.1)
c4b6a4d8d8903c4b80ae1fd78ec2bbd8	fig|292415.6.peg.77	Cytochrome cd1 nitrite reductase (EC:1.7.2.1)
2931cc9d77f9b98049ada7f809299e11	fig|155864.8.peg.4489	Phosphopantothenoylcysteine decarboxylase (EC 4.1.1.36) / Phosphopantothenoylcysteine synthetase (EC 6.3.2.5)
2931cc9d77f9b98049ada7f809299e11	fig|198215.6.peg.4453	Phosphopantothenoylcysteine decarboxylase (EC 4.1.1.36) / Phosphopantothenoylcysteine synthetase (EC 6.3.2.5)
2931cc9d77f9b98049ada7f809299e11	fig|199310.4.peg.4188	Phosphopantothenoylcysteine decarboxylase (EC 4.1.1.36) / Phosphopantothenoylcysteine synthetase (EC 6.3.2.5)
2931cc9d77f9b98049ada7f809299e11	fig|316385.5.peg.3773	Phosphopantothenoylcysteine decarboxylase (EC 4.1.1.36) / Phosphopantothenoylcysteine synthetase (EC 6.3.2.5)
2931cc9d77f9b98049ada7f809299e11	fig|316385.7.peg.3859	Phosphopantothenoylcysteine decarboxylase (EC 4.1.1.36) / Phosphopantothenoylcysteine synthetase (EC 6.3.2.5)
2931cc9d77f9b98049ada7f809299e11	fig|316401.4.peg.4409	Phosphopantothenoylcysteine decarboxylase (EC 4.1.1.36) / Phosphopantothenoylcysteine synthetase (EC 6.3.2.5)
2931cc9d77f9b98049ada7f809299e11	fig|316407.3.peg.3488	Phosphopantothenoylcysteine decarboxylase (EC 4.1.1.36) / Phosphopantothenoylcysteine synthetase (EC 6.3.2.5)
2931cc9d77f9b98049ada7f809299e11	fig|331112.6.peg.3760	Phosphopantothenoylcysteine decarboxylase (EC 4.1.1.36) / Phosphopantothenoylcysteine synthetase (EC 6.3.2.5)
2931cc9d77f9b98049ada7f809299e11	fig|340184.6.peg.3022	Phosphopantothenoylcysteine decarboxylase (EC 4.1.1.36) / Phosphopantothenoylcysteine synthetase (EC 6.3.2.5)
2931cc9d77f9b98049ada7f809299e11	fig|340186.5.peg.3716	Phosphopantothenoylcysteine decarboxylase (EC 4.1.1.36) / Phosphopantothenoylcysteine synthetase (EC 6.3.2.5)
2931cc9d77f9b98049ada7f809299e11	fig|340197.5.peg.550	Phosphopantothenoylcysteine decarboxylase (EC 4.1.1.36) / Phosphopantothenoylcysteine synthetase (EC 6.3.2.5)
2931cc9d77f9b98049ada7f809299e11	fig|358709.5.peg.151	Phosphopantothenoylcysteine decarboxylase (EC 4.1.1.36) / Phosphopantothenoylcysteine synthetase (EC 6.3.2.5)
2931cc9d77f9b98049ada7f809299e11	fig|362663.8.peg.3772	Phosphopantothenoylcysteine decarboxylase (EC 4.1.1.36) / Phosphopantothenoylcysteine synthetase (EC 6.3.2.5)
2931cc9d77f9b98049ada7f809299e11	fig|362663.9.peg.3786	Phosphopantothenoylcysteine decarboxylase (EC 4.1.1.36) / Phosphopantothenoylcysteine synthetase (EC 6.3.2.5)
2931cc9d77f9b98049ada7f809299e11	fig|386585.9.peg.4730	Phosphopantothenoylcysteine decarboxylase (EC 4.1.1.36) / Phosphopantothenoylcysteine synthetase (EC 6.3.2.5)
2931cc9d77f9b98049ada7f809299e11	fig|413997.3.peg.3672	Phosphopantothenoylcysteine decarboxylase (EC 4.1.1.36) / Phosphopantothenoylcysteine synthetase (EC 6.3.2.5)
2931cc9d77f9b98049ada7f809299e11	fig|444447.5.peg.1988	Phosphopantothenoylcysteine decarboxylase (EC 4.1.1.36) / Phosphopantothenoylcysteine synthetase (EC 6.3.2.5)
2931cc9d77f9b98049ada7f809299e11	fig|444448.5.peg.1824	Phosphopantothenoylcysteine decarboxylase (EC 4.1.1.36) / Phosphopantothenoylcysteine synthetase (EC 6.3.2.5)
2931cc9d77f9b98049ada7f809299e11	fig|444449.5.peg.3069	Phosphopantothenoylcysteine decarboxylase (EC 4.1.1.36) / Phosphopantothenoylcysteine synthetase (EC 6.3.2.5)
2931cc9d77f9b98049ada7f809299e11	fig|444450.8.peg.4905	Phosphopantothenoylcysteine decarboxylase (EC 4.1.1.36) / Phosphopantothenoylcysteine synthetase (EC 6.3.2.5)
2931cc9d77f9b98049ada7f809299e11	fig|444452.5.peg.1745	Phosphopantothenoylcysteine decarboxylase (EC 4.1.1.36) / Phosphopantothenoylcysteine synthetase (EC 6.3.2.5)
2931cc9d77f9b98049ada7f809299e11	fig|457400.3.peg.1704	Phosphopantothenoylcysteine decarboxylase (EC 4.1.1.36) / Phosphopantothenoylcysteine synthetase (EC 6.3.2.5)
2931cc9d77f9b98049ada7f809299e11	fig|469008.4.peg.91	Phosphopantothenoylcysteine decarboxylase (EC 4.1.1.36) / Phosphopantothenoylcysteine synthetase (EC 6.3.2.5)
2931cc9d77f9b98049ada7f809299e11	fig|478005.5.peg.333	Phosphopantothenoylcysteine decarboxylase (EC 4.1.1.36) / Phosphopantothenoylcysteine synthetase (EC 6.3.2.5)
2931cc9d77f9b98049ada7f809299e11	fig|478006.5.peg.347	Phosphopantothenoylcysteine decarboxylase (EC 4.1.1.36) / Phosphopantothenoylcysteine synthetase (EC 6.3.2.5)
2931cc9d77f9b98049ada7f809299e11	fig|478007.5.peg.1405	Phosphopantothenoylcysteine decarboxylase (EC 4.1.1.36) / Phosphopantothenoylcysteine synthetase (EC 6.3.2.5)
2931cc9d77f9b98049ada7f809299e11	fig|478008.5.peg.876	Phosphopantothenoylcysteine decarboxylase (EC 4.1.1.36) / Phosphopantothenoylcysteine synthetase (EC 6.3.2.5)
2931cc9d77f9b98049ada7f809299e11	fig|481805.6.peg.78	Phosphopantothenoylcysteine decarboxylase (EC 4.1.1.36) / Phosphopantothenoylcysteine synthetase (EC 6.3.2.5)
2931cc9d77f9b98049ada7f809299e11	fig|502346.5.peg.2113	Phosphopantothenoylcysteine decarboxylase (EC 4.1.1.36) / Phosphopantothenoylcysteine synthetase (EC 6.3.2.5)
2931cc9d77f9b98049ada7f809299e11	fig|511145.12.peg.3759	Phosphopantothenoylcysteine decarboxylase (EC 4.1.1.36) / Phosphopantothenoylcysteine synthetase (EC 6.3.2.5)
2931cc9d77f9b98049ada7f809299e11	fig|511145.6.peg.3741	Phosphopantothenoylcysteine decarboxylase (EC 4.1.1.36) / Phosphopantothenoylcysteine synthetase (EC 6.3.2.5)
2931cc9d77f9b98049ada7f809299e11	fig|511693.5.peg.3689	Phosphopantothenoylcysteine decarboxylase (EC 4.1.1.36) / Phosphopantothenoylcysteine synthetase (EC 6.3.2.5)
2931cc9d77f9b98049ada7f809299e11	fig|525281.3.peg.3094	Phosphopantothenoylcysteine decarboxylase (EC 4.1.1.36) / Phosphopantothenoylcysteine synthetase (EC 6.3.2.5)
2931cc9d77f9b98049ada7f809299e11	fig|536056.3.peg.68	Phosphopantothenoylcysteine decarboxylase (EC 4.1.1.36) / Phosphopantothenoylcysteine synthetase (EC 6.3.2.5)
2931cc9d77f9b98049ada7f809299e11	fig|544404.4.peg.4716	Phosphopantothenoylcysteine decarboxylase (EC 4.1.1.36) / Phosphopantothenoylcysteine synthetase (EC 6.3.2.5)
2931cc9d77f9b98049ada7f809299e11	fig|550672.3.peg.3706	Phosphopantothenoylcysteine decarboxylase (EC 4.1.1.36) / Phosphopantothenoylcysteine synthetase (EC 6.3.2.5)
2931cc9d77f9b98049ada7f809299e11	fig|550676.3.peg.3833	Phosphopantothenoylcysteine decarboxylase (EC 4.1.1.36) / Phosphopantothenoylcysteine synthetase (EC 6.3.2.5)
2931cc9d77f9b98049ada7f809299e11	fig|550677.3.peg.4213	Phosphopantothenoylcysteine decarboxylase (EC 4.1.1.36) / Phosphopantothenoylcysteine synthetase (EC 6.3.2.5)
2931cc9d77f9b98049ada7f809299e11	fig|562.372.peg.4039	Phosphopantothenoylcysteine decarboxylase (EC 4.1.1.36) / Phosphopantothenoylcysteine synthetase (EC 6.3.2.5)
2931cc9d77f9b98049ada7f809299e11	fig|562.373.peg.1571	Phosphopantothenoylcysteine decarboxylase (EC 4.1.1.36) / Phosphopantothenoylcysteine synthetase (EC 6.3.2.5)
2931cc9d77f9b98049ada7f809299e11	fig|562.374.peg.1163	Phosphopantothenoylcysteine decarboxylase (EC 4.1.1.36) / Phosphopantothenoylcysteine synthetase (EC 6.3.2.5)
2931cc9d77f9b98049ada7f809299e11	fig|573235.3.peg.5111	Phosphopantothenoylcysteine decarboxylase (EC 4.1.1.36) / Phosphopantothenoylcysteine synthetase (EC 6.3.2.5)
2931cc9d77f9b98049ada7f809299e11	fig|585057.4.peg.4300	Phosphopantothenoylcysteine decarboxylase (EC 4.1.1.36) / Phosphopantothenoylcysteine synthetase (EC 6.3.2.5)
2931cc9d77f9b98049ada7f809299e11	fig|585057.6.peg.4308	Phosphopantothenoylcysteine decarboxylase (EC 4.1.1.36) / Phosphopantothenoylcysteine synthetase (EC 6.3.2.5)
2931cc9d77f9b98049ada7f809299e11	fig|591020.3.peg.4304	Phosphopantothenoylcysteine decarboxylase (EC 4.1.1.36) / Phosphopantothenoylcysteine synthetase (EC 6.3.2.5)
2931cc9d77f9b98049ada7f809299e11	fig|595496.3.peg.3641	Phosphopantothenoylcysteine decarboxylase (EC 4.1.1.36) / Phosphopantothenoylcysteine synthetase (EC 6.3.2.5)
2931cc9d77f9b98049ada7f809299e11	fig|637388.3.peg.1252	Phosphopantothenoylcysteine decarboxylase (EC 4.1.1.36) / Phosphopantothenoylcysteine synthetase (EC 6.3.2.5)
2931cc9d77f9b98049ada7f809299e11	fig|655817.3.peg.4260	Phosphopantothenoylcysteine decarboxylase (EC 4.1.1.36) / Phosphopantothenoylcysteine synthetase (EC 6.3.2.5)
2931cc9d77f9b98049ada7f809299e11	fig|656393.3.peg.4732	Phosphopantothenoylcysteine decarboxylase (EC 4.1.1.36) / Phosphopantothenoylcysteine synthetase (EC 6.3.2.5)
2931cc9d77f9b98049ada7f809299e11	fig|656408.3.peg.4090	Phosphopantothenoylcysteine decarboxylase (EC 4.1.1.36) / Phosphopantothenoylcysteine synthetase (EC 6.3.2.5)
2931cc9d77f9b98049ada7f809299e11	fig|656414.3.peg.4159	Phosphopantothenoylcysteine decarboxylase (EC 4.1.1.36) / Phosphopantothenoylcysteine synthetase (EC 6.3.2.5)
2931cc9d77f9b98049ada7f809299e11	fig|656419.3.peg.4672	Phosphopantothenoylcysteine decarboxylase (EC 4.1.1.36) / Phosphopantothenoylcysteine synthetase (EC 6.3.2.5)
2931cc9d77f9b98049ada7f809299e11	fig|656444.3.peg.4955	Phosphopantothenoylcysteine decarboxylase (EC 4.1.1.36) / Phosphopantothenoylcysteine synthetase (EC 6.3.2.5)
2931cc9d77f9b98049ada7f809299e11	fig|679204.3.peg.4684	Phosphopantothenoylcysteine decarboxylase (EC 4.1.1.36) / Phosphopantothenoylcysteine synthetase (EC 6.3.2.5)
2931cc9d77f9b98049ada7f809299e11	fig|679207.4.peg.137	Phosphopantothenoylcysteine decarboxylase (EC 4.1.1.36) / Phosphopantothenoylcysteine synthetase (EC 6.3.2.5)
2931cc9d77f9b98049ada7f809299e11	fig|701177.3.peg.4402	Phosphopantothenoylcysteine decarboxylase (EC 4.1.1.36) / Phosphopantothenoylcysteine synthetase (EC 6.3.2.5)
2931cc9d77f9b98049ada7f809299e11	fig|749528.3.peg.2956	Phosphopantothenoylcysteine decarboxylase (EC 4.1.1.36) / Phosphopantothenoylcysteine synthetase (EC 6.3.2.5)
2931cc9d77f9b98049ada7f809299e11	fig|749538.3.peg.1504	Phosphopantothenoylcysteine decarboxylase (EC 4.1.1.36) / Phosphopantothenoylcysteine synthetase (EC 6.3.2.5)
2931cc9d77f9b98049ada7f809299e11	fig|749540.3.peg.924	Phosphopantothenoylcysteine decarboxylase (EC 4.1.1.36) / Phosphopantothenoylcysteine synthetase (EC 6.3.2.5)
2931cc9d77f9b98049ada7f809299e11	fig|749544.3.peg.391	Phosphopantothenoylcysteine decarboxylase (EC 4.1.1.36) / Phosphopantothenoylcysteine synthetase (EC 6.3.2.5)
2931cc9d77f9b98049ada7f809299e11	fig|749546.3.peg.1398	Phosphopantothenoylcysteine decarboxylase (EC 4.1.1.36) / Phosphopantothenoylcysteine synthetase (EC 6.3.2.5)
2931cc9d77f9b98049ada7f809299e11	fig|749547.3.peg.299	Phosphopantothenoylcysteine decarboxylase (EC 4.1.1.36) / Phosphopantothenoylcysteine synthetase (EC 6.3.2.5)
2931cc9d77f9b98049ada7f809299e11	fig|749548.3.peg.4081	Phosphopantothenoylcysteine decarboxylase (EC 4.1.1.36) / Phosphopantothenoylcysteine synthetase (EC 6.3.2.5)
2931cc9d77f9b98049ada7f809299e11	fig|749550.3.peg.2319	Phosphopantothenoylcysteine decarboxylase (EC 4.1.1.36) / Phosphopantothenoylcysteine synthetase (EC 6.3.2.5)
2931cc9d77f9b98049ada7f809299e11	fig|753642.3.peg.4293	Phosphopantothenoylcysteine decarboxylase (EC 4.1.1.36) / Phosphopantothenoylcysteine synthetase (EC 6.3.2.5)
2931cc9d77f9b98049ada7f809299e11	fig|83333.1.peg.3575	Phosphopantothenoylcysteine decarboxylase (EC 4.1.1.36) / Phosphopantothenoylcysteine synthetase (EC 6.3.2.5)
3ae4d43505c990161419f906e48968a9	fig|316385.7.peg.4356	Fumarate hydratase class I, anaerobic (EC 4.2.1.2)
3ae4d43505c990161419f906e48968a9	fig|316401.4.peg.5037	Fumarate hydratase class I, anaerobic (EC 4.2.1.2)
3ae4d43505c990161419f906e48968a9	fig|316407.3.peg.3959	Fumarate hydratase class I, anaerobic (EC 4.2.1.2)
3ae4d43505c990161419f906e48968a9	fig|457400.3.peg.404	Fumarate hydratase class I, anaerobic (EC 4.2.1.2)
3ae4d43505c990161419f906e48968a9	fig|457401.3.peg.3721	Fumarate hydratase class I, anaerobic (EC 4.2.1.2)
3ae4d43505c990161419f906e48968a9	fig|511145.12.peg.4253	Fumarate hydratase class I, anaerobic (EC 4.2.1.2)
3ae4d43505c990161419f906e48968a9	fig|511145.6.peg.4232	Fumarate hydratase class I, anaerobic (EC 4.2.1.2)
3ae4d43505c990161419f906e48968a9	fig|536056.3.peg.4109	Fumarate hydratase class I, anaerobic (EC 4.2.1.2)
3ae4d43505c990161419f906e48968a9	fig|595495.4.peg.4117	Fumarate hydratase class I, anaerobic (EC 4.2.1.2)
3ae4d43505c990161419f906e48968a9	fig|595496.3.peg.4197	Fumarate hydratase class I, anaerobic (EC 4.2.1.2)
3ae4d43505c990161419f906e48968a9	fig|656414.3.peg.4682	Fumarate hydratase class I, anaerobic (EC 4.2.1.2)
3ae4d43505c990161419f906e48968a9	fig|749538.3.peg.1244	Fumarate hydratase class I, anaerobic (EC 4.2.1.2)
3ae4d43505c990161419f906e48968a9	fig|749540.3.peg.1310	Fumarate hydratase class I, anaerobic (EC 4.2.1.2)
3ae4d43505c990161419f906e48968a9	fig|749544.3.peg.105	Fumarate hydratase class I, anaerobic (EC 4.2.1.2)
3ae4d43505c990161419f906e48968a9	fig|749548.3.peg.3639	Fumarate hydratase class I, anaerobic (EC 4.2.1.2)
3ae4d43505c990161419f906e48968a9	fig|83333.1.peg.4034	Fumarate hydratase class I, anaerobic (EC 4.2.1.2)
26eaf1df9fdf15cadf981e7caf74a214	fig|3702.1.peg.8702	Isocitrate dehydrogenase [NAD] subunit II, mitochondrial precursor (EC 1.1.1.41)
26eaf1df9fdf15cadf981e7caf74a214	fig|3702.7.peg.7878	Isocitrate dehydrogenase [NAD] (EC 1.1.1.41)
9ad6e3a84e5b73ad38cfa41cb2fe708d	fig|224308.43.peg.920	Thiamin biosynthesis protein ThiC
9ad6e3a84e5b73ad38cfa41cb2fe708d	fig|224308.49.peg.904	Thiamin biosynthesis protein ThiC
9ad6e3a84e5b73ad38cfa41cb2fe708d	fig|535024.3.peg.4151	Thiamin biosynthesis protein ThiC
9ad6e3a84e5b73ad38cfa41cb2fe708d	fig|535025.4.peg.2013	Thiamin biosynthesis protein ThiC
9ad6e3a84e5b73ad38cfa41cb2fe708d	fig|535026.3.peg.946	Thiamin biosynthesis protein ThiC
0fbb4e4327a0778f49df12c491039489	fig|316385.7.peg.4121	Rhamnulose-1-phosphate aldolase (EC 4.1.2.19)
0fbb4e4327a0778f49df12c491039489	fig|316401.4.peg.4733	Rhamnulose-1-phosphate aldolase (EC 4.1.2.19)
0fbb4e4327a0778f49df12c491039489	fig|316407.3.peg.3242	Rhamnulose-1-phosphate aldolase (EC 4.1.2.19)
0fbb4e4327a0778f49df12c491039489	fig|331112.3.peg.3856	Rhamnulose-1-phosphate aldolase (EC 4.1.2.19)
0fbb4e4327a0778f49df12c491039489	fig|331112.6.peg.4022	Rhamnulose-1-phosphate aldolase (EC 4.1.2.19)
0fbb4e4327a0778f49df12c491039489	fig|344610.3.peg.2262	Rhamnulose-1-phosphate aldolase (EC 4.1.2.19)
0fbb4e4327a0778f49df12c491039489	fig|344610.7.peg.2290	Rhamnulose-1-phosphate aldolase (EC 4.1.2.19)
0fbb4e4327a0778f49df12c491039489	fig|457400.3.peg.1965	Rhamnulose-1-phosphate aldolase (EC 4.1.2.19)
0fbb4e4327a0778f49df12c491039489	fig|457401.3.peg.4497	Rhamnulose-1-phosphate aldolase (EC 4.1.2.19)
0fbb4e4327a0778f49df12c491039489	fig|481805.3.peg.4431	Rhamnulose-1-phosphate aldolase (EC 4.1.2.19)
0fbb4e4327a0778f49df12c491039489	fig|481805.6.peg.4410	Rhamnulose-1-phosphate aldolase (EC 4.1.2.19)
0fbb4e4327a0778f49df12c491039489	fig|511145.12.peg.4017	Rhamnulose-1-phosphate aldolase (EC 4.1.2.19)
0fbb4e4327a0778f49df12c491039489	fig|511145.6.peg.4000	Rhamnulose-1-phosphate aldolase (EC 4.1.2.19)
0fbb4e4327a0778f49df12c491039489	fig|536056.3.peg.4343	Rhamnulose-1-phosphate aldolase (EC 4.1.2.19)
0fbb4e4327a0778f49df12c491039489	fig|595496.3.peg.3902	Rhamnulose-1-phosphate aldolase (EC 4.1.2.19)
0fbb4e4327a0778f49df12c491039489	fig|656414.3.peg.4437	Rhamnulose-1-phosphate aldolase (EC 4.1.2.19)
0fbb4e4327a0778f49df12c491039489	fig|670888.3.peg.2321	Rhamnulose-1-phosphate aldolase (EC 4.1.2.19)
0fbb4e4327a0778f49df12c491039489	fig|749540.3.peg.1649	Rhamnulose-1-phosphate aldolase (EC 4.1.2.19)
0fbb4e4327a0778f49df12c491039489	fig|749548.3.peg.799	Rhamnulose-1-phosphate aldolase (EC 4.1.2.19)
0fbb4e4327a0778f49df12c491039489	fig|83333.1.peg.3823	Rhamnulose-1-phosphate aldolase (EC 4.1.2.19)
3133b183438b1574006ba1d1d4458b3e	fig|316385.5.peg.2886	Phosphoadenylyl-sulfate reductase [thioredoxin] (EC 1.8.4.8)
3133b183438b1574006ba1d1d4458b3e	fig|316385.7.peg.2953	Phosphoadenylyl-sulfate reductase [thioredoxin] (EC 1.8.4.8)
3133b183438b1574006ba1d1d4458b3e	fig|316407.3.peg.2665	Phosphoadenylyl-sulfate reductase [thioredoxin] (EC 1.8.4.8)
3133b183438b1574006ba1d1d4458b3e	fig|481805.3.peg.1012	Phosphoadenylyl-sulfate reductase [thioredoxin] (EC 1.8.4.8)
3133b183438b1574006ba1d1d4458b3e	fig|481805.6.peg.1009	Phosphoadenylyl-sulfate reductase [thioredoxin] (EC 1.8.4.8)
3133b183438b1574006ba1d1d4458b3e	fig|511145.12.peg.2860	Phosphoadenylyl-sulfate reductase [thioredoxin] (EC 1.8.4.8)
3133b183438b1574006ba1d1d4458b3e	fig|511145.6.peg.2843	Phosphoadenylyl-sulfate reductase [thioredoxin] (EC 1.8.4.8)
3133b183438b1574006ba1d1d4458b3e	fig|536056.3.peg.969	Phosphoadenylyl-sulfate reductase [thioredoxin] (EC 1.8.4.8)
3133b183438b1574006ba1d1d4458b3e	fig|585054.5.peg.297	Phosphoadenylyl-sulfate reductase [thioredoxin] (EC 1.8.4.8)
3133b183438b1574006ba1d1d4458b3e	fig|585056.7.peg.3267	Phosphoadenylyl-sulfate reductase [thioredoxin] (EC 1.8.4.8)
3133b183438b1574006ba1d1d4458b3e	fig|595496.3.peg.2739	Phosphoadenylyl-sulfate reductase [thioredoxin] (EC 1.8.4.8)
3133b183438b1574006ba1d1d4458b3e	fig|656379.3.peg.4124	Phosphoadenylyl-sulfate reductase [thioredoxin] (EC 1.8.4.8)
3133b183438b1574006ba1d1d4458b3e	fig|656380.3.peg.4040	Phosphoadenylyl-sulfate reductase [thioredoxin] (EC 1.8.4.8)
3133b183438b1574006ba1d1d4458b3e	fig|656444.3.peg.3867	Phosphoadenylyl-sulfate reductase [thioredoxin] (EC 1.8.4.8)
3133b183438b1574006ba1d1d4458b3e	fig|749538.3.peg.3636	Phosphoadenylyl-sulfate reductase [thioredoxin] (EC 1.8.4.8)
3133b183438b1574006ba1d1d4458b3e	fig|749548.3.peg.927	Phosphoadenylyl-sulfate reductase [thioredoxin] (EC 1.8.4.8)
3133b183438b1574006ba1d1d4458b3e	fig|749549.3.peg.4726	Phosphoadenylyl-sulfate reductase [thioredoxin] (EC 1.8.4.8)
3133b183438b1574006ba1d1d4458b3e	fig|83333.1.peg.2718	Phosphoadenylyl-sulfate reductase [thioredoxin] (EC 1.8.4.8)
a4ac8e3dfc104265600f1930652ff66e	fig|9606.3.peg.2728	Glutamine synthetase type II, eukaryotic (EC 6.3.1.2)
a4ac8e3dfc104265600f1930652ff66e	fig|9606.3.peg.2729	Glutamine synthetase type II, eukaryotic (EC 6.3.1.2)
93d81da1b1f52df389eea97274a51a5c	fig|272947.1.peg.427	Glycerol-3-phosphate dehydrogenase [NAD(P)+] (EC 1.1.1.94)
93d81da1b1f52df389eea97274a51a5c	fig|272947.5.peg.455	Glycerol-3-phosphate dehydrogenase [NAD(P)+] (EC 1.1.1.94)
93d81da1b1f52df389eea97274a51a5c	fig|449216.3.peg.452	Glycerol-3-phosphate dehydrogenase [NAD(P)+] (EC 1.1.1.94)
d8a5707091f9eab15266614036183235	fig|3702.1.peg.10119	3-hydroxyisobutyryl-CoA hydrolase (EC 3.1.2.4)
d8a5707091f9eab15266614036183235	fig|3702.7.peg.4153	3-hydroxyisobutyryl-CoA hydrolase (EC 3.1.2.4)
1412cded298392a937e631db3653f453	fig|103690.10.peg.5399	Glucose-1-phosphate adenylyltransferase (EC 2.7.7.27)
1412cded298392a937e631db3653f453	fig|103690.1.peg.4952	Glucose-1-phosphate adenylyltransferase (EC 2.7.7.27)
e18726a68a2b896287606399d5c1aa10	fig|272620.3.peg.3529	Glycerol dehydratase medium subunit (EC 4.2.1.30)
e18726a68a2b896287606399d5c1aa10	fig|272620.9.peg.3548	Glycerol dehydratase medium subunit (EC 4.2.1.30)
e18726a68a2b896287606399d5c1aa10	fig|469608.3.peg.3402	Glycerol dehydratase medium subunit (EC 4.2.1.30)
e18726a68a2b896287606399d5c1aa10	fig|484021.4.peg.4191	Glycerol dehydratase medium subunit (EC 4.2.1.30)
e18726a68a2b896287606399d5c1aa10	fig|507522.6.peg.839	Glycerol dehydratase medium subunit (EC 4.2.1.30)
e18726a68a2b896287606399d5c1aa10	fig|667127.3.peg.2822	Glycerol dehydratase medium subunit (EC 4.2.1.30)
4f300ca4ddd9e8400429797eb3efb34f	fig|322710.5.peg.2816	Citrate synthase (si) (EC 2.3.3.1)
4f300ca4ddd9e8400429797eb3efb34f	fig|354.1.peg.3338	Citrate synthase (si) (EC 2.3.3.1)
eb5d54b2e67953f4262a23806209fe9d	fig|300852.3.peg.1894	2-amino-3-ketobutyrate coenzyme A ligase (EC 2.3.1.29)
eb5d54b2e67953f4262a23806209fe9d	fig|300852.9.peg.1553	2-amino-3-ketobutyrate coenzyme A ligase (EC 2.3.1.29)
231cde29fd90ffdeb0d36f6deea14d2e	fig|316385.7.peg.1566	Aldehyde dehydrogenase A (EC 1.2.1.22) / Glycolaldehyde dehydrogenase (EC 1.2.1.21)
231cde29fd90ffdeb0d36f6deea14d2e	fig|316407.3.peg.1376	Aldehyde dehydrogenase A (EC 1.2.1.22) / Glycolaldehyde dehydrogenase (EC 1.2.1.21)
231cde29fd90ffdeb0d36f6deea14d2e	fig|457401.3.peg.629	Aldehyde dehydrogenase A (EC 1.2.1.22) / Glycolaldehyde dehydrogenase (EC 1.2.1.21)
231cde29fd90ffdeb0d36f6deea14d2e	fig|511145.12.peg.1478	Aldehyde dehydrogenase A (EC 1.2.1.22) / Glycolaldehyde dehydrogenase (EC 1.2.1.21)
231cde29fd90ffdeb0d36f6deea14d2e	fig|511145.6.peg.1465	Aldehyde dehydrogenase A (EC 1.2.1.22) / Glycolaldehyde dehydrogenase (EC 1.2.1.21)
231cde29fd90ffdeb0d36f6deea14d2e	fig|536056.3.peg.2343	Aldehyde dehydrogenase A (EC 1.2.1.22) / Glycolaldehyde dehydrogenase (EC 1.2.1.21)
231cde29fd90ffdeb0d36f6deea14d2e	fig|595496.3.peg.1367	Aldehyde dehydrogenase A (EC 1.2.1.22) / Glycolaldehyde dehydrogenase (EC 1.2.1.21)
231cde29fd90ffdeb0d36f6deea14d2e	fig|749538.3.peg.3483	Aldehyde dehydrogenase A (EC 1.2.1.22) / Glycolaldehyde dehydrogenase (EC 1.2.1.21)
231cde29fd90ffdeb0d36f6deea14d2e	fig|749544.3.peg.30	Aldehyde dehydrogenase A (EC 1.2.1.22) / Glycolaldehyde dehydrogenase (EC 1.2.1.21)
231cde29fd90ffdeb0d36f6deea14d2e	fig|749548.3.peg.48	Aldehyde dehydrogenase A (EC 1.2.1.22) / Glycolaldehyde dehydrogenase (EC 1.2.1.21)
231cde29fd90ffdeb0d36f6deea14d2e	fig|83333.1.peg.1403	Aldehyde dehydrogenase A (EC 1.2.1.22) / Glycolaldehyde dehydrogenase (EC 1.2.1.21)
a92f08eac05f8bf7334986e2bdafdb75	fig|3702.1.peg.23147	identical to sulfate transporter [Arabidopsis thaliana] GI:2114104; go_function: sulfate transporter activity [goid 0015116]; go_process: sulfate transport [goid 0008272] / sulfate transporter
a92f08eac05f8bf7334986e2bdafdb75	fig|3702.7.peg.12224	Sulfate permease
7d583e96767adba541aec100a7975574	fig|190486.1.peg.1759	Glucose-6-phosphate isomerase (EC 5.3.1.9)
7d583e96767adba541aec100a7975574	fig|190486.7.peg.1871	Glucose-6-phosphate isomerase (EC 5.3.1.9)
124c6ffa0fb608b2f175247458f097f9	fig|155864.1.peg.2904	PTS system, galactitol-specific IIA component (EC 2.7.1.69)
124c6ffa0fb608b2f175247458f097f9	fig|155864.8.peg.2789	PTS system, galactitol-specific IIA component (EC 2.7.1.69)
124c6ffa0fb608b2f175247458f097f9	fig|199310.1.peg.2550	PTS system, galactitol-specific IIA component (EC 2.7.1.69)
124c6ffa0fb608b2f175247458f097f9	fig|199310.4.peg.2464	PTS system, galactitol-specific IIA component (EC 2.7.1.69)
124c6ffa0fb608b2f175247458f097f9	fig|316385.5.peg.2209	PTS system, galactitol-specific IIA component (EC 2.7.1.69)
124c6ffa0fb608b2f175247458f097f9	fig|316385.7.peg.2259	PTS system, galactitol-specific IIA component (EC 2.7.1.69)
124c6ffa0fb608b2f175247458f097f9	fig|331111.12.peg.2663	PTS system, galactitol-specific IIA component (EC 2.7.1.69)
124c6ffa0fb608b2f175247458f097f9	fig|331111.3.peg.124	PTS system, galactitol-specific IIA component (EC 2.7.1.69)
124c6ffa0fb608b2f175247458f097f9	fig|331112.3.peg.2087	PTS system, galactitol-specific IIA component (EC 2.7.1.69)
124c6ffa0fb608b2f175247458f097f9	fig|331112.6.peg.2182	PTS system, galactitol-specific IIA component (EC 2.7.1.69)
124c6ffa0fb608b2f175247458f097f9	fig|340185.3.peg.1249	PTS system, galactitol-specific IIA component (EC 2.7.1.69)
124c6ffa0fb608b2f175247458f097f9	fig|340185.4.peg.1318	PTS system, galactitol-specific IIA component (EC 2.7.1.69)
124c6ffa0fb608b2f175247458f097f9	fig|344601.3.peg.3223	PTS system, galactitol-specific IIA component (EC 2.7.1.69)
124c6ffa0fb608b2f175247458f097f9	fig|344601.5.peg.3368	PTS system, galactitol-specific IIA component (EC 2.7.1.69)
124c6ffa0fb608b2f175247458f097f9	fig|386585.9.peg.3029	PTS system, galactitol-specific IIA component (EC 2.7.1.69)
124c6ffa0fb608b2f175247458f097f9	fig|444447.5.peg.175	PTS system, galactitol-specific IIA component (EC 2.7.1.69)
124c6ffa0fb608b2f175247458f097f9	fig|444448.5.peg.43	PTS system, galactitol-specific IIA component (EC 2.7.1.69)
124c6ffa0fb608b2f175247458f097f9	fig|444449.5.peg.1280	PTS system, galactitol-specific IIA component (EC 2.7.1.69)
124c6ffa0fb608b2f175247458f097f9	fig|444450.8.peg.3074	PTS system, galactitol-specific IIA component (EC 2.7.1.69)
124c6ffa0fb608b2f175247458f097f9	fig|444451.5.peg.3493	PTS system, galactitol-specific IIA component (EC 2.7.1.69)
124c6ffa0fb608b2f175247458f097f9	fig|444452.5.peg.3118	PTS system, galactitol-specific IIA component (EC 2.7.1.69)
124c6ffa0fb608b2f175247458f097f9	fig|444453.5.peg.3773	PTS system, galactitol-specific IIA component (EC 2.7.1.69)
124c6ffa0fb608b2f175247458f097f9	fig|444454.5.peg.1805	PTS system, galactitol-specific IIA component (EC 2.7.1.69)
124c6ffa0fb608b2f175247458f097f9	fig|478004.5.peg.3081	PTS system, galactitol-specific IIA component (EC 2.7.1.69)
124c6ffa0fb608b2f175247458f097f9	fig|478005.5.peg.3895	PTS system, galactitol-specific IIA component (EC 2.7.1.69)
124c6ffa0fb608b2f175247458f097f9	fig|478006.5.peg.2316	PTS system, galactitol-specific IIA component (EC 2.7.1.69)
124c6ffa0fb608b2f175247458f097f9	fig|478007.5.peg.1761	PTS system, galactitol-specific IIA component (EC 2.7.1.69)
124c6ffa0fb608b2f175247458f097f9	fig|478008.5.peg.4167	PTS system, galactitol-specific IIA component (EC 2.7.1.69)
124c6ffa0fb608b2f175247458f097f9	fig|481805.3.peg.1664	PTS system, galactitol-specific IIA component (EC 2.7.1.69)
124c6ffa0fb608b2f175247458f097f9	fig|481805.6.peg.1659	PTS system, galactitol-specific IIA component (EC 2.7.1.69)
124c6ffa0fb608b2f175247458f097f9	fig|502346.5.peg.4488	PTS system, galactitol-specific IIA component (EC 2.7.1.69)
124c6ffa0fb608b2f175247458f097f9	fig|511145.12.peg.2171	PTS system, galactitol-specific IIA component (EC 2.7.1.69)
124c6ffa0fb608b2f175247458f097f9	fig|511145.6.peg.2156	PTS system, galactitol-specific IIA component (EC 2.7.1.69)
124c6ffa0fb608b2f175247458f097f9	fig|525281.3.peg.3893	PTS system, galactitol-specific IIA component (EC 2.7.1.69)
124c6ffa0fb608b2f175247458f097f9	fig|536056.3.peg.1649	PTS system, galactitol-specific IIA component (EC 2.7.1.69)
124c6ffa0fb608b2f175247458f097f9	fig|544404.4.peg.2936	PTS system, galactitol-specific IIA component (EC 2.7.1.69)
124c6ffa0fb608b2f175247458f097f9	fig|562.371.peg.4295	PTS system, galactitol-specific IIA component (EC 2.7.1.69)
124c6ffa0fb608b2f175247458f097f9	fig|562.372.peg.5766	PTS system, galactitol-specific IIA component (EC 2.7.1.69)
124c6ffa0fb608b2f175247458f097f9	fig|562.373.peg.4593	PTS system, galactitol-specific IIA component (EC 2.7.1.69)
124c6ffa0fb608b2f175247458f097f9	fig|562.374.peg.1805	PTS system, galactitol-specific IIA component (EC 2.7.1.69)
124c6ffa0fb608b2f175247458f097f9	fig|566546.3.peg.4657	PTS system, galactitol-specific IIA component (EC 2.7.1.69)
124c6ffa0fb608b2f175247458f097f9	fig|566546.4.peg.2275	PTS system, galactitol-specific IIA component (EC 2.7.1.69)
124c6ffa0fb608b2f175247458f097f9	fig|570506.3.peg.3528	PTS system, galactitol-specific IIA component (EC 2.7.1.69)
124c6ffa0fb608b2f175247458f097f9	fig|585395.4.peg.2686	PTS system, galactitol-specific IIA component (EC 2.7.1.69)
124c6ffa0fb608b2f175247458f097f9	fig|595495.4.peg.351	PTS system, galactitol-specific IIA component (EC 2.7.1.69)
124c6ffa0fb608b2f175247458f097f9	fig|595496.3.peg.2062	PTS system, galactitol-specific IIA component (EC 2.7.1.69)
124c6ffa0fb608b2f175247458f097f9	fig|637388.3.peg.2138	PTS system, galactitol-specific IIA component (EC 2.7.1.69)
124c6ffa0fb608b2f175247458f097f9	fig|655817.3.peg.2534	PTS system, galactitol-specific IIA component (EC 2.7.1.69)
124c6ffa0fb608b2f175247458f097f9	fig|656408.3.peg.2336	PTS system, galactitol-specific IIA component (EC 2.7.1.69)
124c6ffa0fb608b2f175247458f097f9	fig|656440.3.peg.2074	PTS system, galactitol-specific IIA component (EC 2.7.1.69)
124c6ffa0fb608b2f175247458f097f9	fig|656443.3.peg.2791	PTS system, galactitol-specific IIA component (EC 2.7.1.69)
124c6ffa0fb608b2f175247458f097f9	fig|656444.3.peg.3064	PTS system, galactitol-specific IIA component (EC 2.7.1.69)
124c6ffa0fb608b2f175247458f097f9	fig|679204.3.peg.122	PTS system, galactitol-specific IIA component (EC 2.7.1.69)
124c6ffa0fb608b2f175247458f097f9	fig|679206.4.peg.1577	PTS system, galactitol-specific IIA component (EC 2.7.1.69)
124c6ffa0fb608b2f175247458f097f9	fig|679207.4.peg.3852	PTS system, galactitol-specific IIA component (EC 2.7.1.69)
124c6ffa0fb608b2f175247458f097f9	fig|749528.3.peg.1588	PTS system, galactitol-specific IIA component (EC 2.7.1.69)
124c6ffa0fb608b2f175247458f097f9	fig|749531.3.peg.2056	PTS system, galactitol-specific IIA component (EC 2.7.1.69)
124c6ffa0fb608b2f175247458f097f9	fig|749538.3.peg.1311	PTS system, galactitol-specific IIA component (EC 2.7.1.69)
124c6ffa0fb608b2f175247458f097f9	fig|749546.3.peg.2852	PTS system, galactitol-specific IIA component (EC 2.7.1.69)
124c6ffa0fb608b2f175247458f097f9	fig|83333.1.peg.2069	PTS system, galactitol-specific IIA component (EC 2.7.1.69)
124c6ffa0fb608b2f175247458f097f9	fig|83334.1.peg.2901	PTS system, galactitol-specific IIA component (EC 2.7.1.69)
6d0413038d610571069b4ad580691162	fig|216597.6.peg.2245	Propanediol dehydratase large subunit (EC 4.2.1.28)
6d0413038d610571069b4ad580691162	fig|272994.5.peg.910	Propanediol dehydratase large subunit (EC 4.2.1.28) @ Glycerol dehydratase large subunit (EC 4.2.1.30)
6d0413038d610571069b4ad580691162	fig|28901.42.peg.2007	Propanediol dehydratase large subunit (EC 4.2.1.28)
6d0413038d610571069b4ad580691162	fig|295319.15.peg.883	Propanediol dehydratase large subunit (EC 4.2.1.28)
6d0413038d610571069b4ad580691162	fig|295319.3.peg.2123	Propanediol dehydratase large subunit (EC 4.2.1.28)
6d0413038d610571069b4ad580691162	fig|423368.6.peg.2327	Propanediol dehydratase large subunit (EC 4.2.1.28) @ Glycerol dehydratase large subunit (EC 4.2.1.30)
6d0413038d610571069b4ad580691162	fig|423368.8.peg.2307	Propanediol dehydratase large subunit (EC 4.2.1.28)
6d0413038d610571069b4ad580691162	fig|439842.7.peg.1833	Propanediol dehydratase large subunit (EC 4.2.1.28) @ Glycerol dehydratase large subunit (EC 4.2.1.30)
6d0413038d610571069b4ad580691162	fig|439843.6.peg.2260	Propanediol dehydratase large subunit (EC 4.2.1.28) @ Glycerol dehydratase large subunit (EC 4.2.1.30)
6d0413038d610571069b4ad580691162	fig|439851.5.peg.2399	Propanediol dehydratase large subunit (EC 4.2.1.28) @ Glycerol dehydratase large subunit (EC 4.2.1.30)
6d0413038d610571069b4ad580691162	fig|439851.8.peg.2378	Propanediol dehydratase large subunit (EC 4.2.1.28)
6d0413038d610571069b4ad580691162	fig|465516.5.peg.194	Propanediol dehydratase large subunit (EC 4.2.1.28)
6d0413038d610571069b4ad580691162	fig|550537.3.peg.2161	Propanediol dehydratase large subunit (EC 4.2.1.28) @ Glycerol dehydratase large subunit (EC 4.2.1.30)
6d0413038d610571069b4ad580691162	fig|554290.7.peg.863	Propanediol dehydratase large subunit (EC 4.2.1.28) @ Glycerol dehydratase large subunit (EC 4.2.1.30)
6d0413038d610571069b4ad580691162	fig|554290.9.peg.880	Propanediol dehydratase large subunit (EC 4.2.1.28)
6d0413038d610571069b4ad580691162	fig|99287.1.peg.1965	Propanediol dehydratase large subunit (EC 4.2.1.28)
9a8522c154b66a69b4c03fc2a82ec13c	fig|555970.3.peg.969	Xylulose-5-phosphate phosphoketolase (EC 4.1.2.9); Fructose-6-phosphate phosphoketolase (EC 4.1.2.22)
ae36dc89898b673b0d5fbeadbd22eb79	fig|9606.3.peg.6616	6-pyruvoyl tetrahydrobiopterin synthase (EC 4.2.3.12)
cda8b4eb723ba844345e1f004a174646	fig|348776.4.peg.2634	Sulfate and thiosulfate import ATP-binding protein CysA (EC 3.6.3.25)
cda8b4eb723ba844345e1f004a174646	fig|395095.3.peg.2133	Sulfate and thiosulfate import ATP-binding protein CysA (EC 3.6.3.25)
cda8b4eb723ba844345e1f004a174646	fig|515617.4.peg.3161	Sulfate and thiosulfate import ATP-binding protein CysA (EC 3.6.3.25)
cda8b4eb723ba844345e1f004a174646	fig|537209.3.peg.2511	Sulfate and thiosulfate import ATP-binding protein CysA (EC 3.6.3.25)
cda8b4eb723ba844345e1f004a174646	fig|537210.3.peg.2902	Sulfate and thiosulfate import ATP-binding protein CysA (EC 3.6.3.25)
cda8b4eb723ba844345e1f004a174646	fig|555461.3.peg.2513	Sulfate and thiosulfate import ATP-binding protein CysA (EC 3.6.3.25)
cda8b4eb723ba844345e1f004a174646	fig|83331.1.peg.2541	Sulfate and thiosulfate import ATP-binding protein CysA (EC 3.6.3.25)
3dbf2e28e039acdb27aba57a7e14a764	fig|198214.1.peg.2635	L-fuculose phosphate aldolase (EC 4.1.2.17)
3dbf2e28e039acdb27aba57a7e14a764	fig|198214.7.peg.3348	L-fuculose phosphate aldolase (EC 4.1.2.17)
3dbf2e28e039acdb27aba57a7e14a764	fig|198215.1.peg.2575	L-fuculose phosphate aldolase (EC 4.1.2.17)
3dbf2e28e039acdb27aba57a7e14a764	fig|198215.6.peg.3302	L-fuculose phosphate aldolase (EC 4.1.2.17)
3dbf2e28e039acdb27aba57a7e14a764	fig|216598.1.peg.54	L-fuculose phosphate aldolase (EC 4.1.2.17)
3dbf2e28e039acdb27aba57a7e14a764	fig|300267.13.peg.3621	L-fuculose phosphate aldolase (EC 4.1.2.17)
3dbf2e28e039acdb27aba57a7e14a764	fig|316385.7.peg.2997	L-fuculose phosphate aldolase (EC 4.1.2.17)
3dbf2e28e039acdb27aba57a7e14a764	fig|316401.4.peg.3389	L-fuculose phosphate aldolase (EC 4.1.2.17)
3dbf2e28e039acdb27aba57a7e14a764	fig|316407.3.peg.2702	L-fuculose phosphate aldolase (EC 4.1.2.17)
3dbf2e28e039acdb27aba57a7e14a764	fig|344610.3.peg.4082	L-fuculose phosphate aldolase (EC 4.1.2.17)
3dbf2e28e039acdb27aba57a7e14a764	fig|344610.7.peg.3470	L-fuculose phosphate aldolase (EC 4.1.2.17)
3dbf2e28e039acdb27aba57a7e14a764	fig|358708.5.peg.4568	L-fuculose phosphate aldolase (EC 4.1.2.17)
3dbf2e28e039acdb27aba57a7e14a764	fig|373384.10.peg.3260	L-fuculose phosphate aldolase (EC 4.1.2.17)
3dbf2e28e039acdb27aba57a7e14a764	fig|373384.11.peg.3295	L-fuculose phosphate aldolase (EC 4.1.2.17)
3dbf2e28e039acdb27aba57a7e14a764	fig|457400.3.peg.792	L-fuculose phosphate aldolase (EC 4.1.2.17)
3dbf2e28e039acdb27aba57a7e14a764	fig|457401.3.peg.2746	L-fuculose phosphate aldolase (EC 4.1.2.17)
3dbf2e28e039acdb27aba57a7e14a764	fig|511145.12.peg.2900	L-fuculose phosphate aldolase (EC 4.1.2.17)
3dbf2e28e039acdb27aba57a7e14a764	fig|511145.6.peg.2885	L-fuculose phosphate aldolase (EC 4.1.2.17)
3dbf2e28e039acdb27aba57a7e14a764	fig|536056.3.peg.927	L-fuculose phosphate aldolase (EC 4.1.2.17)
3dbf2e28e039acdb27aba57a7e14a764	fig|591020.3.peg.3340	L-fuculose phosphate aldolase (EC 4.1.2.17)
3dbf2e28e039acdb27aba57a7e14a764	fig|595496.3.peg.2783	L-fuculose phosphate aldolase (EC 4.1.2.17)
3dbf2e28e039acdb27aba57a7e14a764	fig|621.8.peg.1874	L-fuculose phosphate aldolase (EC 4.1.2.17)
3dbf2e28e039acdb27aba57a7e14a764	fig|656414.3.peg.3235	L-fuculose phosphate aldolase (EC 4.1.2.17)
3dbf2e28e039acdb27aba57a7e14a764	fig|749540.3.peg.1804	L-fuculose phosphate aldolase (EC 4.1.2.17)
3dbf2e28e039acdb27aba57a7e14a764	fig|749548.3.peg.2869	L-fuculose phosphate aldolase (EC 4.1.2.17)
3dbf2e28e039acdb27aba57a7e14a764	fig|754093.3.peg.2773	L-fuculose phosphate aldolase (EC 4.1.2.17)
3dbf2e28e039acdb27aba57a7e14a764	fig|83333.1.peg.2756	L-fuculose phosphate aldolase (EC 4.1.2.17)
02e191c9876af184d9507aa85d7d6638	fig|1140.3.peg.1798	Sulfate transport system permease protein CysT
02e191c9876af184d9507aa85d7d6638	fig|1140.7.peg.1907	Sulfate transport system permease protein CysT
f7aaab4cd298ad419f65f6dfd595d619	fig|216599.1.peg.4548	Soluble aldose sugar dehydrogenase, PQQ-dependent (EC 1.1.5.-)
f7aaab4cd298ad419f65f6dfd595d619	fig|316385.5.peg.902	Soluble aldose sugar dehydrogenase, PQQ-dependent (EC 1.1.5.-)
f7aaab4cd298ad419f65f6dfd595d619	fig|316401.4.peg.1045	Soluble aldose sugar dehydrogenase, PQQ-dependent (EC 1.1.5.-)
f7aaab4cd298ad419f65f6dfd595d619	fig|316407.3.peg.804	Soluble aldose sugar dehydrogenase, PQQ-dependent (EC 1.1.5.-)
f7aaab4cd298ad419f65f6dfd595d619	fig|457401.3.peg.1681	Soluble aldose sugar dehydrogenase, PQQ-dependent (EC 1.1.5.-)
f7aaab4cd298ad419f65f6dfd595d619	fig|481805.3.peg.3013	Soluble aldose sugar dehydrogenase, PQQ-dependent (EC 1.1.5.-)
f7aaab4cd298ad419f65f6dfd595d619	fig|550677.3.peg.2107	Soluble aldose sugar dehydrogenase, PQQ-dependent (EC 1.1.5.-)
f7aaab4cd298ad419f65f6dfd595d619	fig|622.8.peg.1900	Soluble aldose sugar dehydrogenase, PQQ-dependent (EC 1.1.5.-)
f7aaab4cd298ad419f65f6dfd595d619	fig|623.7.peg.1815	Soluble aldose sugar dehydrogenase, PQQ-dependent (EC 1.1.5.-)
f7aaab4cd298ad419f65f6dfd595d619	fig|656414.3.peg.1040	Soluble aldose sugar dehydrogenase, PQQ-dependent (EC 1.1.5.-)
f7aaab4cd298ad419f65f6dfd595d619	fig|670888.3.peg.1401	Soluble aldose sugar dehydrogenase, PQQ-dependent (EC 1.1.5.-)
f7aaab4cd298ad419f65f6dfd595d619	fig|749538.3.peg.692	Soluble aldose sugar dehydrogenase, PQQ-dependent (EC 1.1.5.-)
f7aaab4cd298ad419f65f6dfd595d619	fig|749540.3.peg.2775	Soluble aldose sugar dehydrogenase, PQQ-dependent (EC 1.1.5.-)
f7aaab4cd298ad419f65f6dfd595d619	fig|749544.3.peg.2724	Soluble aldose sugar dehydrogenase, PQQ-dependent (EC 1.1.5.-)
f7aaab4cd298ad419f65f6dfd595d619	fig|749548.3.peg.3177	Soluble aldose sugar dehydrogenase, PQQ-dependent (EC 1.1.5.-)
f7aaab4cd298ad419f65f6dfd595d619	fig|83333.1.peg.823	Soluble aldose sugar dehydrogenase, PQQ-dependent (EC 1.1.5.-)
b2d92ef8bbc9c4d42558552351a33811	fig|316385.5.peg.2841	Formate hydrogenlyase complex 3 iron-sulfur protein; Formate hydrogenlyase subunit 6; Ni,Fe-hydrogenase III medium subunit
b2d92ef8bbc9c4d42558552351a33811	fig|316385.7.peg.2907	Formate hydrogenlyase complex 3 iron-sulfur protein; Formate hydrogenlyase subunit 6; Ni,Fe-hydrogenase III medium subunit
b2d92ef8bbc9c4d42558552351a33811	fig|316407.3.peg.2623	Formate hydrogenlyase complex 3 iron-sulfur protein; Formate hydrogenlyase subunit 6; Ni,Fe-hydrogenase III medium subunit
b2d92ef8bbc9c4d42558552351a33811	fig|457401.3.peg.2824	Formate hydrogenlyase complex 3 iron-sulfur protein; Formate hydrogenlyase subunit 6; Ni,Fe-hydrogenase III medium subunit
b2d92ef8bbc9c4d42558552351a33811	fig|511145.12.peg.2812	Formate hydrogenlyase complex 3 iron-sulfur protein; Formate hydrogenlyase subunit 6; Ni,Fe-hydrogenase III medium subunit
b2d92ef8bbc9c4d42558552351a33811	fig|511145.6.peg.2796	Formate hydrogenlyase complex 3 iron-sulfur protein; Formate hydrogenlyase subunit 6; Ni,Fe-hydrogenase III medium subunit
b2d92ef8bbc9c4d42558552351a33811	fig|536056.3.peg.1015	Formate hydrogenlyase complex 3 iron-sulfur protein; Formate hydrogenlyase subunit 6; Ni,Fe-hydrogenase III medium subunit
b2d92ef8bbc9c4d42558552351a33811	fig|595496.3.peg.2693	Formate hydrogenlyase complex 3 iron-sulfur protein; Formate hydrogenlyase subunit 6; Ni,Fe-hydrogenase III medium subunit
b2d92ef8bbc9c4d42558552351a33811	fig|656414.3.peg.3153	Formate hydrogenlyase complex 3 iron-sulfur protein; Formate hydrogenlyase subunit 6; Ni,Fe-hydrogenase III medium subunit
b2d92ef8bbc9c4d42558552351a33811	fig|749538.3.peg.581	Formate hydrogenlyase complex 3 iron-sulfur protein; Formate hydrogenlyase subunit 6; Ni,Fe-hydrogenase III medium subunit
b2d92ef8bbc9c4d42558552351a33811	fig|749540.3.peg.1870	Formate hydrogenlyase complex 3 iron-sulfur protein; Formate hydrogenlyase subunit 6; Ni,Fe-hydrogenase III medium subunit
b2d92ef8bbc9c4d42558552351a33811	fig|749544.3.peg.837	Formate hydrogenlyase complex 3 iron-sulfur protein; Formate hydrogenlyase subunit 6; Ni,Fe-hydrogenase III medium subunit
b2d92ef8bbc9c4d42558552351a33811	fig|749548.3.peg.3321	Formate hydrogenlyase complex 3 iron-sulfur protein; Formate hydrogenlyase subunit 6; Ni,Fe-hydrogenase III medium subunit
b2d92ef8bbc9c4d42558552351a33811	fig|83333.1.peg.2675	Formate hydrogenlyase complex 3 iron-sulfur protein; Formate hydrogenlyase subunit 6; Ni,Fe-hydrogenase III medium subunit
ab8baeeb1c1d5326711501154205bae5	fig|9606.3.peg.223	Glucose-6-phosphate 1-dehydrogenase (EC 1.1.1.49)
d389c518dc90eb3c04723d6eec4fa049	fig|224308.1.peg.3979	5-keto-2-deoxygluconokinase (EC 2.7.1.92)
d389c518dc90eb3c04723d6eec4fa049	fig|224308.43.peg.4168	5-keto-2-deoxygluconokinase (EC 2.7.1.92)
d389c518dc90eb3c04723d6eec4fa049	fig|224308.49.peg.3981	5-keto-2-deoxygluconokinase (EC 2.7.1.92)
d389c518dc90eb3c04723d6eec4fa049	fig|535024.3.peg.3210	5-keto-2-deoxygluconokinase (EC 2.7.1.92)
d389c518dc90eb3c04723d6eec4fa049	fig|535025.4.peg.1093	5-keto-2-deoxygluconokinase (EC 2.7.1.92)
d389c518dc90eb3c04723d6eec4fa049	fig|535026.3.peg.4238	5-keto-2-deoxygluconokinase (EC 2.7.1.92)
6b24fb3340b8945dd5466735721eb575	fig|3702.1.peg.5896	nearly identical to SP|Q9ZPJ8 Ammonium transporter 1, member 2 (AtAMT1;2) {Arabidopsis thaliana}; go_component: membrane [goid 0016020]; go_function: ammonium transporter activity [goid 0008519]; go_process: transport [goid 0006810] / ammonium transporter 1, member 2 (AMT1.2)
6b24fb3340b8945dd5466735721eb575	fig|3702.7.peg.23809	Ammonium transporter
74b3fa5912b35f1c9cbc3a1b471600c6	fig|3702.1.peg.4315	Glutamine synthetase (EC 6.3.1.2), cytosolic
74b3fa5912b35f1c9cbc3a1b471600c6	fig|3702.7.peg.18148	Glutamine synthetase type II, eukaryotic (EC 6.3.1.2)
4d38ebd6845ab47b3973a567e7410848	fig|3702.1.peg.26652	identical to SP|P55228; go_function: glucose-1-phosphate adenylyltransferase activity [goid 0008878]; go_process: starch biosynthesis [goid 0019252] / glucose-1-phosphate adenylyltransferase small subunit 1 (APS1) / ADP-glucose pyrophosphorylase (ADG1)
4d38ebd6845ab47b3973a567e7410848	fig|3702.7.peg.16367	Glucose-1-phosphate adenylyltransferase (EC 2.7.7.27)
12e008b723248effb2f6b92b592314a5	fig|499177.3.peg.2219	D-proline reductase, 45 kDa subunit (EC 1.21.4.1) / D-proline reductase, 23 kDa subunit (EC 1.21.4.1)
7e62d7067b7891818c6a800475588545	fig|316407.3.peg.791	Pyruvate formate-lyase activating enzyme (EC 1.97.1.4)
7e62d7067b7891818c6a800475588545	fig|83333.1.peg.810	Pyruvate formate-lyase activating enzyme (EC 1.97.1.4)
a29555aebeedc02f8e439afdb844e21e	fig|316385.5.peg.4413	L-idonate, D-gluconate, 5-keto-D-gluconate transporter
a29555aebeedc02f8e439afdb844e21e	fig|316385.7.peg.4504	L-idonate, D-gluconate, 5-keto-D-gluconate transporter
a29555aebeedc02f8e439afdb844e21e	fig|316407.3.peg.4097	L-idonate, D-gluconate, 5-keto-D-gluconate transporter
a29555aebeedc02f8e439afdb844e21e	fig|331112.3.peg.4231	L-idonate, D-gluconate, 5-keto-D-gluconate transporter
a29555aebeedc02f8e439afdb844e21e	fig|331112.6.peg.4404	L-idonate, D-gluconate, 5-keto-D-gluconate transporter
a29555aebeedc02f8e439afdb844e21e	fig|431946.3.peg.4323	L-idonate, D-gluconate, 5-keto-D-gluconate transporter
a29555aebeedc02f8e439afdb844e21e	fig|511145.12.peg.4396	L-idonate, D-gluconate, 5-keto-D-gluconate transporter
a29555aebeedc02f8e439afdb844e21e	fig|511145.6.peg.4374	L-idonate, D-gluconate, 5-keto-D-gluconate transporter
a29555aebeedc02f8e439afdb844e21e	fig|536056.3.peg.3962	L-idonate, D-gluconate, 5-keto-D-gluconate transporter
a29555aebeedc02f8e439afdb844e21e	fig|562.375.peg.2798	L-idonate, D-gluconate, 5-keto-D-gluconate transporter
a29555aebeedc02f8e439afdb844e21e	fig|566546.3.peg.3046	L-idonate, D-gluconate, 5-keto-D-gluconate transporter
a29555aebeedc02f8e439afdb844e21e	fig|566546.4.peg.4563	L-idonate, D-gluconate, 5-keto-D-gluconate transporter
a29555aebeedc02f8e439afdb844e21e	fig|585055.6.peg.4846	L-idonate, D-gluconate, 5-keto-D-gluconate transporter
a29555aebeedc02f8e439afdb844e21e	fig|585055.8.peg.4850	L-idonate, D-gluconate, 5-keto-D-gluconate transporter
a29555aebeedc02f8e439afdb844e21e	fig|585056.7.peg.4965	L-idonate, D-gluconate, 5-keto-D-gluconate transporter
a29555aebeedc02f8e439afdb844e21e	fig|595495.4.peg.2170	L-idonate, D-gluconate, 5-keto-D-gluconate transporter
a29555aebeedc02f8e439afdb844e21e	fig|595496.3.peg.4339	L-idonate, D-gluconate, 5-keto-D-gluconate transporter
a29555aebeedc02f8e439afdb844e21e	fig|656379.3.peg.646	L-idonate, D-gluconate, 5-keto-D-gluconate transporter
a29555aebeedc02f8e439afdb844e21e	fig|656380.3.peg.635	L-idonate, D-gluconate, 5-keto-D-gluconate transporter
a29555aebeedc02f8e439afdb844e21e	fig|656419.3.peg.5523	L-idonate, D-gluconate, 5-keto-D-gluconate transporter
a29555aebeedc02f8e439afdb844e21e	fig|749540.3.peg.57	L-idonate, D-gluconate, 5-keto-D-gluconate transporter
a29555aebeedc02f8e439afdb844e21e	fig|749549.3.peg.3315	L-idonate, D-gluconate, 5-keto-D-gluconate transporter
a29555aebeedc02f8e439afdb844e21e	fig|83333.1.peg.4175	L-idonate, D-gluconate, 5-keto-D-gluconate transporter
4fb159bd103dbaf0e750ffb1d7efc7c4	fig|155864.8.peg.2792	Fructose-bisphosphate aldolase class I (EC 4.1.2.13)
4fb159bd103dbaf0e750ffb1d7efc7c4	fig|199310.4.peg.2467	Fructose-bisphosphate aldolase class I (EC 4.1.2.13)
4fb159bd103dbaf0e750ffb1d7efc7c4	fig|300267.13.peg.2743	Fructose-bisphosphate aldolase class I (EC 4.1.2.13)
4fb159bd103dbaf0e750ffb1d7efc7c4	fig|316385.7.peg.2263	Fructose-bisphosphate aldolase class I (EC 4.1.2.13)
4fb159bd103dbaf0e750ffb1d7efc7c4	fig|316407.3.peg.2031	Fructose-bisphosphate aldolase class I (EC 4.1.2.13)
4fb159bd103dbaf0e750ffb1d7efc7c4	fig|331111.12.peg.2666	Fructose-bisphosphate aldolase class I (EC 4.1.2.13)
4fb159bd103dbaf0e750ffb1d7efc7c4	fig|331112.6.peg.2185	Fructose-bisphosphate aldolase class I (EC 4.1.2.13)
4fb159bd103dbaf0e750ffb1d7efc7c4	fig|340184.6.peg.3235	Fructose-bisphosphate aldolase class I (EC 4.1.2.13)
4fb159bd103dbaf0e750ffb1d7efc7c4	fig|340185.4.peg.1321	Fructose-bisphosphate aldolase class I (EC 4.1.2.13)
4fb159bd103dbaf0e750ffb1d7efc7c4	fig|340197.5.peg.160	Fructose-bisphosphate aldolase class I (EC 4.1.2.13)
4fb159bd103dbaf0e750ffb1d7efc7c4	fig|344601.5.peg.3365	Fructose-bisphosphate aldolase class I (EC 4.1.2.13)
4fb159bd103dbaf0e750ffb1d7efc7c4	fig|344609.11.peg.1261	Fructose-bisphosphate aldolase class I (EC 4.1.2.13)
4fb159bd103dbaf0e750ffb1d7efc7c4	fig|344610.7.peg.4211	Fructose-bisphosphate aldolase class I (EC 4.1.2.13)
4fb159bd103dbaf0e750ffb1d7efc7c4	fig|362663.8.peg.2148	Fructose-bisphosphate aldolase class I (EC 4.1.2.13)
4fb159bd103dbaf0e750ffb1d7efc7c4	fig|362663.9.peg.2153	Fructose-bisphosphate aldolase class I (EC 4.1.2.13)
4fb159bd103dbaf0e750ffb1d7efc7c4	fig|386585.9.peg.3032	Fructose-bisphosphate aldolase class I (EC 4.1.2.13)
4fb159bd103dbaf0e750ffb1d7efc7c4	fig|405955.13.peg.2311	Fructose-bisphosphate aldolase class I (EC 4.1.2.13)
4fb159bd103dbaf0e750ffb1d7efc7c4	fig|409438.11.peg.2518	Fructose-bisphosphate aldolase class I (EC 4.1.2.13)
4fb159bd103dbaf0e750ffb1d7efc7c4	fig|413997.3.peg.2117	Fructose-bisphosphate aldolase class I (EC 4.1.2.13)
4fb159bd103dbaf0e750ffb1d7efc7c4	fig|431946.3.peg.2065	Fructose-bisphosphate aldolase class I (EC 4.1.2.13)
4fb159bd103dbaf0e750ffb1d7efc7c4	fig|439855.10.peg.1125	Fructose-bisphosphate aldolase class I (EC 4.1.2.13)
4fb159bd103dbaf0e750ffb1d7efc7c4	fig|444447.5.peg.178	Fructose-bisphosphate aldolase class I (EC 4.1.2.13)
4fb159bd103dbaf0e750ffb1d7efc7c4	fig|444448.5.peg.46	Fructose-bisphosphate aldolase class I (EC 4.1.2.13)
4fb159bd103dbaf0e750ffb1d7efc7c4	fig|444449.5.peg.1283	Fructose-bisphosphate aldolase class I (EC 4.1.2.13)
4fb159bd103dbaf0e750ffb1d7efc7c4	fig|444450.8.peg.3077	Fructose-bisphosphate aldolase class I (EC 4.1.2.13)
4fb159bd103dbaf0e750ffb1d7efc7c4	fig|444451.5.peg.3490	Fructose-bisphosphate aldolase class I (EC 4.1.2.13)
4fb159bd103dbaf0e750ffb1d7efc7c4	fig|444452.5.peg.3115	Fructose-bisphosphate aldolase class I (EC 4.1.2.13)
4fb159bd103dbaf0e750ffb1d7efc7c4	fig|444453.5.peg.3776	Fructose-bisphosphate aldolase class I (EC 4.1.2.13)
4fb159bd103dbaf0e750ffb1d7efc7c4	fig|444454.5.peg.1808	Fructose-bisphosphate aldolase class I (EC 4.1.2.13)
4fb159bd103dbaf0e750ffb1d7efc7c4	fig|457401.3.peg.2057	Fructose-bisphosphate aldolase class I (EC 4.1.2.13)
4fb159bd103dbaf0e750ffb1d7efc7c4	fig|469008.4.peg.1609	Fructose-bisphosphate aldolase class I (EC 4.1.2.13)
4fb159bd103dbaf0e750ffb1d7efc7c4	fig|469598.5.peg.2681	Fructose-bisphosphate aldolase class I (EC 4.1.2.13)
4fb159bd103dbaf0e750ffb1d7efc7c4	fig|478004.5.peg.3084	Fructose-bisphosphate aldolase class I (EC 4.1.2.13)
4fb159bd103dbaf0e750ffb1d7efc7c4	fig|478006.5.peg.2319	Fructose-bisphosphate aldolase class I (EC 4.1.2.13)
4fb159bd103dbaf0e750ffb1d7efc7c4	fig|478007.5.peg.1758	Fructose-bisphosphate aldolase class I (EC 4.1.2.13)
4fb159bd103dbaf0e750ffb1d7efc7c4	fig|478008.5.peg.4170	Fructose-bisphosphate aldolase class I (EC 4.1.2.13)
4fb159bd103dbaf0e750ffb1d7efc7c4	fig|481805.6.peg.1656	Fructose-bisphosphate aldolase class I (EC 4.1.2.13)
4fb159bd103dbaf0e750ffb1d7efc7c4	fig|502346.5.peg.4485	Fructose-bisphosphate aldolase class I (EC 4.1.2.13)
4fb159bd103dbaf0e750ffb1d7efc7c4	fig|511145.12.peg.2174	Fructose-bisphosphate aldolase class I (EC 4.1.2.13)
4fb159bd103dbaf0e750ffb1d7efc7c4	fig|511145.6.peg.2159	Fructose-bisphosphate aldolase class I (EC 4.1.2.13)
4fb159bd103dbaf0e750ffb1d7efc7c4	fig|525281.3.peg.3896	Fructose-bisphosphate aldolase class I (EC 4.1.2.13)
4fb159bd103dbaf0e750ffb1d7efc7c4	fig|536056.3.peg.1646	Fructose-bisphosphate aldolase class I (EC 4.1.2.13)
4fb159bd103dbaf0e750ffb1d7efc7c4	fig|544404.4.peg.2939	Fructose-bisphosphate aldolase class I (EC 4.1.2.13)
4fb159bd103dbaf0e750ffb1d7efc7c4	fig|550676.3.peg.2391	Fructose-bisphosphate aldolase class I (EC 4.1.2.13)
4fb159bd103dbaf0e750ffb1d7efc7c4	fig|550677.3.peg.1640	Fructose-bisphosphate aldolase class I (EC 4.1.2.13)
4fb159bd103dbaf0e750ffb1d7efc7c4	fig|556266.3.peg.1301	Fructose-bisphosphate aldolase class I (EC 4.1.2.13)
4fb159bd103dbaf0e750ffb1d7efc7c4	fig|562.371.peg.4298	Fructose-bisphosphate aldolase class I (EC 4.1.2.13)
4fb159bd103dbaf0e750ffb1d7efc7c4	fig|562.372.peg.5763	Fructose-bisphosphate aldolase class I (EC 4.1.2.13)
4fb159bd103dbaf0e750ffb1d7efc7c4	fig|562.373.peg.4590	Fructose-bisphosphate aldolase class I (EC 4.1.2.13)
4fb159bd103dbaf0e750ffb1d7efc7c4	fig|562.374.peg.1808	Fructose-bisphosphate aldolase class I (EC 4.1.2.13)
4fb159bd103dbaf0e750ffb1d7efc7c4	fig|562.375.peg.2356	Fructose-bisphosphate aldolase class I (EC 4.1.2.13)
4fb159bd103dbaf0e750ffb1d7efc7c4	fig|562.376.peg.4011	Fructose-bisphosphate aldolase class I (EC 4.1.2.13)
4fb159bd103dbaf0e750ffb1d7efc7c4	fig|566546.3.peg.4654	Fructose-bisphosphate aldolase class I (EC 4.1.2.13)
4fb159bd103dbaf0e750ffb1d7efc7c4	fig|566546.4.peg.2278	Fructose-bisphosphate aldolase class I (EC 4.1.2.13)
4fb159bd103dbaf0e750ffb1d7efc7c4	fig|570506.3.peg.3531	Fructose-bisphosphate aldolase class I (EC 4.1.2.13)
4fb159bd103dbaf0e750ffb1d7efc7c4	fig|573235.3.peg.3071	Fructose-bisphosphate aldolase class I (EC 4.1.2.13)
4fb159bd103dbaf0e750ffb1d7efc7c4	fig|585035.6.peg.2219	Fructose-bisphosphate aldolase class I (EC 4.1.2.13)
4fb159bd103dbaf0e750ffb1d7efc7c4	fig|585056.7.peg.2610	Fructose-bisphosphate aldolase class I (EC 4.1.2.13)
4fb159bd103dbaf0e750ffb1d7efc7c4	fig|585395.4.peg.2692	Fructose-bisphosphate aldolase class I (EC 4.1.2.13)
4fb159bd103dbaf0e750ffb1d7efc7c4	fig|585396.4.peg.2907	Fructose-bisphosphate aldolase class I (EC 4.1.2.13)
4fb159bd103dbaf0e750ffb1d7efc7c4	fig|595495.4.peg.348	Fructose-bisphosphate aldolase class I (EC 4.1.2.13)
4fb159bd103dbaf0e750ffb1d7efc7c4	fig|595496.3.peg.2065	Fructose-bisphosphate aldolase class I (EC 4.1.2.13)
4fb159bd103dbaf0e750ffb1d7efc7c4	fig|621.8.peg.1255	Fructose-bisphosphate aldolase class I (EC 4.1.2.13)
4fb159bd103dbaf0e750ffb1d7efc7c4	fig|637388.3.peg.2135	Fructose-bisphosphate aldolase class I (EC 4.1.2.13)
4fb159bd103dbaf0e750ffb1d7efc7c4	fig|637912.3.peg.2906	Fructose-bisphosphate aldolase class I (EC 4.1.2.13)
4fb159bd103dbaf0e750ffb1d7efc7c4	fig|655817.3.peg.2537	Fructose-bisphosphate aldolase class I (EC 4.1.2.13)
4fb159bd103dbaf0e750ffb1d7efc7c4	fig|656380.3.peg.2184	Fructose-bisphosphate aldolase class I (EC 4.1.2.13)
4fb159bd103dbaf0e750ffb1d7efc7c4	fig|656393.3.peg.3121	Fructose-bisphosphate aldolase class I (EC 4.1.2.13)
4fb159bd103dbaf0e750ffb1d7efc7c4	fig|656408.3.peg.2339	Fructose-bisphosphate aldolase class I (EC 4.1.2.13)
4fb159bd103dbaf0e750ffb1d7efc7c4	fig|656417.3.peg.2760	Fructose-bisphosphate aldolase class I (EC 4.1.2.13)
4fb159bd103dbaf0e750ffb1d7efc7c4	fig|656419.3.peg.2850	Fructose-bisphosphate aldolase class I (EC 4.1.2.13)
4fb159bd103dbaf0e750ffb1d7efc7c4	fig|656437.3.peg.2354	Fructose-bisphosphate aldolase class I (EC 4.1.2.13)
4fb159bd103dbaf0e750ffb1d7efc7c4	fig|656440.3.peg.2077	Fructose-bisphosphate aldolase class I (EC 4.1.2.13)
4fb159bd103dbaf0e750ffb1d7efc7c4	fig|656443.3.peg.2794	Fructose-bisphosphate aldolase class I (EC 4.1.2.13)
4fb159bd103dbaf0e750ffb1d7efc7c4	fig|656444.3.peg.3067	Fructose-bisphosphate aldolase class I (EC 4.1.2.13)
4fb159bd103dbaf0e750ffb1d7efc7c4	fig|670888.3.peg.4194	Fructose-bisphosphate aldolase class I (EC 4.1.2.13)
4fb159bd103dbaf0e750ffb1d7efc7c4	fig|670897.3.peg.3018	Fructose-bisphosphate aldolase class I (EC 4.1.2.13)
4fb159bd103dbaf0e750ffb1d7efc7c4	fig|679204.3.peg.125	Fructose-bisphosphate aldolase class I (EC 4.1.2.13)
4fb159bd103dbaf0e750ffb1d7efc7c4	fig|679205.4.peg.2953	Fructose-bisphosphate aldolase class I (EC 4.1.2.13)
4fb159bd103dbaf0e750ffb1d7efc7c4	fig|679206.4.peg.1574	Fructose-bisphosphate aldolase class I (EC 4.1.2.13)
4fb159bd103dbaf0e750ffb1d7efc7c4	fig|679207.4.peg.3849	Fructose-bisphosphate aldolase class I (EC 4.1.2.13)
4fb159bd103dbaf0e750ffb1d7efc7c4	fig|701177.3.peg.2647	Fructose-bisphosphate aldolase class I (EC 4.1.2.13)
4fb159bd103dbaf0e750ffb1d7efc7c4	fig|714962.3.peg.2370	Fructose-bisphosphate aldolase class I (EC 4.1.2.13)
4fb159bd103dbaf0e750ffb1d7efc7c4	fig|749528.3.peg.1585	Fructose-bisphosphate aldolase class I (EC 4.1.2.13)
4fb159bd103dbaf0e750ffb1d7efc7c4	fig|749531.3.peg.2052	Fructose-bisphosphate aldolase class I (EC 4.1.2.13)
4fb159bd103dbaf0e750ffb1d7efc7c4	fig|749532.3.peg.4084	Fructose-bisphosphate aldolase class I (EC 4.1.2.13)
4fb159bd103dbaf0e750ffb1d7efc7c4	fig|749533.3.peg.417	Fructose-bisphosphate aldolase class I (EC 4.1.2.13)
4fb159bd103dbaf0e750ffb1d7efc7c4	fig|749538.3.peg.1315	Fructose-bisphosphate aldolase class I (EC 4.1.2.13)
4fb159bd103dbaf0e750ffb1d7efc7c4	fig|749545.3.peg.1680	Fructose-bisphosphate aldolase class I (EC 4.1.2.13)
4fb159bd103dbaf0e750ffb1d7efc7c4	fig|749547.3.peg.3100	Fructose-bisphosphate aldolase class I (EC 4.1.2.13)
4fb159bd103dbaf0e750ffb1d7efc7c4	fig|749548.3.peg.2950	Fructose-bisphosphate aldolase class I (EC 4.1.2.13)
4fb159bd103dbaf0e750ffb1d7efc7c4	fig|749549.3.peg.5000	Fructose-bisphosphate aldolase class I (EC 4.1.2.13)
4fb159bd103dbaf0e750ffb1d7efc7c4	fig|749550.3.peg.1567	Fructose-bisphosphate aldolase class I (EC 4.1.2.13)
4fb159bd103dbaf0e750ffb1d7efc7c4	fig|753642.3.peg.2999	Fructose-bisphosphate aldolase class I (EC 4.1.2.13)
4fb159bd103dbaf0e750ffb1d7efc7c4	fig|754093.3.peg.4815	Fructose-bisphosphate aldolase class I (EC 4.1.2.13)
4fb159bd103dbaf0e750ffb1d7efc7c4	fig|83333.1.peg.2072	Fructose-bisphosphate aldolase class I (EC 4.1.2.13)
4fb159bd103dbaf0e750ffb1d7efc7c4	fig|83334.1.peg.2904	Fructose-bisphosphate aldolase class I (EC 4.1.2.13)
ab54f55c772e5fd1b292b35dcd2c69e7	fig|224308.1.peg.1491	Cytochrome c oxidase polypeptide II (EC 1.9.3.1)
ab54f55c772e5fd1b292b35dcd2c69e7	fig|224308.43.peg.1581	Cytochrome c oxidase polypeptide II (EC 1.9.3.1)
ab54f55c772e5fd1b292b35dcd2c69e7	fig|535024.3.peg.596	Cytochrome c oxidase polypeptide II (EC 1.9.3.1)
ab54f55c772e5fd1b292b35dcd2c69e7	fig|535025.4.peg.2684	Cytochrome c oxidase polypeptide II (EC 1.9.3.1)
ab54f55c772e5fd1b292b35dcd2c69e7	fig|535026.3.peg.1617	Cytochrome c oxidase polypeptide II (EC 1.9.3.1)
63066040d9634663c2a27c6bf4936278	fig|431943.4.peg.3048	NAD-dependent 4-hydroxybutyrate dehydrogenase (EC 1.1.1.61)
63066040d9634663c2a27c6bf4936278	fig|431943.8.peg.3118	NAD-dependent 4-hydroxybutyrate dehydrogenase (EC 1.1.1.61)
63066040d9634663c2a27c6bf4936278	fig|583346.6.peg.3015	NAD-dependent 4-hydroxybutyrate dehydrogenase (EC 1.1.1.61)
d420ec64e1ef900f7a6cf9cdb1cd1e1f	fig|316385.5.peg.597	DNA polymerase III delta subunit (EC 2.7.7.7)
d420ec64e1ef900f7a6cf9cdb1cd1e1f	fig|316385.5.peg.708	DNA polymerase III delta subunit (EC 2.7.7.7)
d420ec64e1ef900f7a6cf9cdb1cd1e1f	fig|316385.7.peg.606	DNA polymerase III delta subunit (EC 2.7.7.7)
d420ec64e1ef900f7a6cf9cdb1cd1e1f	fig|316385.7.peg.717	DNA polymerase III delta subunit (EC 2.7.7.7)
d420ec64e1ef900f7a6cf9cdb1cd1e1f	fig|316401.4.peg.766	DNA polymerase III delta subunit (EC 2.7.7.7)
d420ec64e1ef900f7a6cf9cdb1cd1e1f	fig|316407.3.peg.625	DNA polymerase III delta subunit (EC 2.7.7.7)
d420ec64e1ef900f7a6cf9cdb1cd1e1f	fig|340185.3.peg.1695	DNA polymerase III delta subunit (EC 2.7.7.7)
d420ec64e1ef900f7a6cf9cdb1cd1e1f	fig|344601.3.peg.4086	DNA polymerase III delta subunit (EC 2.7.7.7)
d420ec64e1ef900f7a6cf9cdb1cd1e1f	fig|457401.3.peg.1868	DNA polymerase III delta subunit (EC 2.7.7.7)
d420ec64e1ef900f7a6cf9cdb1cd1e1f	fig|511145.12.peg.671	DNA polymerase III delta subunit (EC 2.7.7.7)
d420ec64e1ef900f7a6cf9cdb1cd1e1f	fig|511145.6.peg.662	DNA polymerase III delta subunit (EC 2.7.7.7)
d420ec64e1ef900f7a6cf9cdb1cd1e1f	fig|536056.3.peg.3156	DNA polymerase III delta subunit (EC 2.7.7.7)
d420ec64e1ef900f7a6cf9cdb1cd1e1f	fig|556266.3.peg.3417	DNA polymerase III delta subunit (EC 2.7.7.7)
d420ec64e1ef900f7a6cf9cdb1cd1e1f	fig|573235.3.peg.723	DNA polymerase III delta subunit (EC 2.7.7.7)
d420ec64e1ef900f7a6cf9cdb1cd1e1f	fig|595496.3.peg.567	DNA polymerase III delta subunit (EC 2.7.7.7)
d420ec64e1ef900f7a6cf9cdb1cd1e1f	fig|656414.3.peg.821	DNA polymerase III delta subunit (EC 2.7.7.7)
d420ec64e1ef900f7a6cf9cdb1cd1e1f	fig|83333.1.peg.641	DNA polymerase III delta subunit (EC 2.7.7.7)
c2905f5a9ecdcb9219901ab76662b83a	fig|158878.14.peg.1969	Sodium/proline symporter
c2905f5a9ecdcb9219901ab76662b83a	fig|158878.1.peg.1902	Sodium/proline symporter
c2905f5a9ecdcb9219901ab76662b83a	fig|158879.11.peg.1854	Sodium/proline symporter
c2905f5a9ecdcb9219901ab76662b83a	fig|158879.1.peg.1771	Sodium/proline symporter
c2905f5a9ecdcb9219901ab76662b83a	fig|196620.1.peg.1843	Sodium/proline symporter
c2905f5a9ecdcb9219901ab76662b83a	fig|196620.5.peg.1917	Sodium/proline symporter
c2905f5a9ecdcb9219901ab76662b83a	fig|282459.1.peg.1809	Sodium/proline symporter
c2905f5a9ecdcb9219901ab76662b83a	fig|282459.5.peg.1902	Sodium/proline symporter
c2905f5a9ecdcb9219901ab76662b83a	fig|359786.13.peg.2071	Sodium/proline symporter
c2905f5a9ecdcb9219901ab76662b83a	fig|359786.3.peg.561	Sodium/proline symporter
c2905f5a9ecdcb9219901ab76662b83a	fig|359787.11.peg.2042	Sodium/proline symporter
c2905f5a9ecdcb9219901ab76662b83a	fig|359787.3.peg.2666	Sodium/proline symporter
c2905f5a9ecdcb9219901ab76662b83a	fig|418127.4.peg.1759	Sodium/proline symporter
c2905f5a9ecdcb9219901ab76662b83a	fig|418127.6.peg.1941	Sodium/proline symporter
c2905f5a9ecdcb9219901ab76662b83a	fig|450394.6.peg.2421	Sodium/proline symporter
c2905f5a9ecdcb9219901ab76662b83a	fig|505321.3.peg.2462	Sodium/proline symporter
c2905f5a9ecdcb9219901ab76662b83a	fig|548474.3.peg.2576	Sodium/proline symporter
c2905f5a9ecdcb9219901ab76662b83a	fig|548475.3.peg.2504	Sodium/proline symporter
c2905f5a9ecdcb9219901ab76662b83a	fig|553565.3.peg.45	Sodium/proline symporter
c2905f5a9ecdcb9219901ab76662b83a	fig|553568.3.peg.203	Sodium/proline symporter
c2905f5a9ecdcb9219901ab76662b83a	fig|553571.3.peg.370	Sodium/proline symporter
c2905f5a9ecdcb9219901ab76662b83a	fig|553573.3.peg.1257	Sodium/proline symporter
c2905f5a9ecdcb9219901ab76662b83a	fig|553577.3.peg.2241	Sodium/proline symporter
c2905f5a9ecdcb9219901ab76662b83a	fig|553580.3.peg.2425	Sodium/proline symporter
c2905f5a9ecdcb9219901ab76662b83a	fig|553581.3.peg.406	Sodium/proline symporter
c2905f5a9ecdcb9219901ab76662b83a	fig|553588.3.peg.1333	Sodium/proline symporter
c2905f5a9ecdcb9219901ab76662b83a	fig|553592.3.peg.1183	Sodium/proline symporter
c2905f5a9ecdcb9219901ab76662b83a	fig|553596.3.peg.610	Sodium/proline symporter
c2905f5a9ecdcb9219901ab76662b83a	fig|553601.3.peg.1590	Sodium/proline symporter
c2905f5a9ecdcb9219901ab76662b83a	fig|585891.3.peg.1967	Sodium/proline symporter
c2905f5a9ecdcb9219901ab76662b83a	fig|681288.4.peg.1938	Sodium/proline symporter
c2905f5a9ecdcb9219901ab76662b83a	fig|685039.3.peg.2051	Sodium/proline symporter
c2905f5a9ecdcb9219901ab76662b83a	fig|703339.3.peg.1898	Sodium/proline symporter
c2905f5a9ecdcb9219901ab76662b83a	fig|762962.3.peg.2492	Sodium/proline symporter
c2905f5a9ecdcb9219901ab76662b83a	fig|869816.3.peg.1921	Sodium/proline symporter
c2905f5a9ecdcb9219901ab76662b83a	fig|93061.3.peg.2003	Sodium/proline symporter
c2905f5a9ecdcb9219901ab76662b83a	fig|93061.5.peg.1923	Sodium/proline symporter
c2905f5a9ecdcb9219901ab76662b83a	fig|93062.19.peg.1917	Sodium/proline symporter
c2905f5a9ecdcb9219901ab76662b83a	fig|93062.4.peg.381	Sodium/proline symporter
4b7bc2261168ea9bc711301006375b8c	fig|10090.3.peg.17679	Selenide,water dikinase (EC 2.7.9.3)
2171ec785adabb63808d814a0dfe6615	fig|10090.3.peg.25319	Phosphopantetheine adenylyltransferase, type II eukaryotic (EC 2.7.7.3) / Dephospho-CoA kinase (EC 2.7.1.24)
7505463cb360f11d88a21ab508dfee53	fig|3702.1.peg.8979	Hexokinase (EC 2.7.1.1)
7505463cb360f11d88a21ab508dfee53	fig|3702.7.peg.8223	hexokinase 2
7b4b05ecd008c26e2f71dc1605fbbbd3	fig|3702.1.peg.14712	Aldehyde dehydrogenase 2C4, cytosolic (EC 1.2.1.3) (ALDH1a) (Protein REDUCED EPIDERMAL FLUORESCENCE 1)
7b4b05ecd008c26e2f71dc1605fbbbd3	fig|3702.7.peg.22130	Aldehyde dehydrogenase (EC 1.2.1.3)
880cb195f659b241621dd44fb21c4c7d	fig|164513.4.peg.1482	Pyridoxamine 5'-phosphate oxidase (EC 1.4.3.5)
880cb195f659b241621dd44fb21c4c7d	fig|233413.1.peg.2615	Pyridoxamine 5'-phosphate oxidase (EC 1.4.3.5)
880cb195f659b241621dd44fb21c4c7d	fig|233413.5.peg.2900	Pyridoxamine 5'-phosphate oxidase (EC 1.4.3.5)
880cb195f659b241621dd44fb21c4c7d	fig|336982.3.peg.2780	Pyridoxamine 5'-phosphate oxidase (EC 1.4.3.5)
880cb195f659b241621dd44fb21c4c7d	fig|336982.7.peg.2933	Pyridoxamine 5'-phosphate oxidase (EC 1.4.3.5)
880cb195f659b241621dd44fb21c4c7d	fig|395095.3.peg.2591	Pyridoxamine 5'-phosphate oxidase (EC 1.4.3.5)
880cb195f659b241621dd44fb21c4c7d	fig|410289.13.peg.2609	Pyridoxamine 5'-phosphate oxidase (EC 1.4.3.5)
880cb195f659b241621dd44fb21c4c7d	fig|410289.15.peg.2874	Pyridoxamine 5'-phosphate oxidase (EC 1.4.3.5)
880cb195f659b241621dd44fb21c4c7d	fig|419947.3.peg.2636	Pyridoxamine 5'-phosphate oxidase (EC 1.4.3.5)
880cb195f659b241621dd44fb21c4c7d	fig|419947.8.peg.2955	Pyridoxamine 5'-phosphate oxidase (EC 1.4.3.5)
880cb195f659b241621dd44fb21c4c7d	fig|419947.9.peg.119	Pyridoxamine 5'-phosphate oxidase (EC 1.4.3.5)
880cb195f659b241621dd44fb21c4c7d	fig|478433.3.peg.2963	Pyridoxamine 5'-phosphate oxidase (EC 1.4.3.5)
880cb195f659b241621dd44fb21c4c7d	fig|478434.4.peg.1510	Pyridoxamine 5'-phosphate oxidase (EC 1.4.3.5)
880cb195f659b241621dd44fb21c4c7d	fig|515615.3.peg.3348	Pyridoxamine 5'-phosphate oxidase (EC 1.4.3.5)
880cb195f659b241621dd44fb21c4c7d	fig|515616.3.peg.2933	Pyridoxamine 5'-phosphate oxidase (EC 1.4.3.5)
880cb195f659b241621dd44fb21c4c7d	fig|515617.4.peg.3534	Pyridoxamine 5'-phosphate oxidase (EC 1.4.3.5)
880cb195f659b241621dd44fb21c4c7d	fig|520140.3.peg.3048	Pyridoxamine 5'-phosphate oxidase (EC 1.4.3.5)
880cb195f659b241621dd44fb21c4c7d	fig|520141.3.peg.2650	Pyridoxamine 5'-phosphate oxidase (EC 1.4.3.5)
880cb195f659b241621dd44fb21c4c7d	fig|537209.3.peg.3010	Pyridoxamine 5'-phosphate oxidase (EC 1.4.3.5)
880cb195f659b241621dd44fb21c4c7d	fig|537210.3.peg.3102	Pyridoxamine 5'-phosphate oxidase (EC 1.4.3.5)
880cb195f659b241621dd44fb21c4c7d	fig|555461.3.peg.3097	Pyridoxamine 5'-phosphate oxidase (EC 1.4.3.5)
880cb195f659b241621dd44fb21c4c7d	fig|561275.4.peg.2871	Pyridoxamine 5'-phosphate oxidase (EC 1.4.3.5)
880cb195f659b241621dd44fb21c4c7d	fig|611302.3.peg.3550	Pyridoxamine 5'-phosphate oxidase (EC 1.4.3.5)
880cb195f659b241621dd44fb21c4c7d	fig|611303.3.peg.3477	Pyridoxamine 5'-phosphate oxidase (EC 1.4.3.5)
880cb195f659b241621dd44fb21c4c7d	fig|611304.3.peg.1404	Pyridoxamine 5'-phosphate oxidase (EC 1.4.3.5)
880cb195f659b241621dd44fb21c4c7d	fig|663886.3.peg.2964	Pyridoxamine 5'-phosphate oxidase (EC 1.4.3.5)
880cb195f659b241621dd44fb21c4c7d	fig|663887.3.peg.2963	Pyridoxamine 5'-phosphate oxidase (EC 1.4.3.5)
880cb195f659b241621dd44fb21c4c7d	fig|675512.3.peg.3003	Pyridoxamine 5'-phosphate oxidase (EC 1.4.3.5)
880cb195f659b241621dd44fb21c4c7d	fig|675513.3.peg.2521	Pyridoxamine 5'-phosphate oxidase (EC 1.4.3.5)
880cb195f659b241621dd44fb21c4c7d	fig|675514.3.peg.3040	Pyridoxamine 5'-phosphate oxidase (EC 1.4.3.5)
880cb195f659b241621dd44fb21c4c7d	fig|675515.3.peg.3435	Pyridoxamine 5'-phosphate oxidase (EC 1.4.3.5)
880cb195f659b241621dd44fb21c4c7d	fig|675516.3.peg.3291	Pyridoxamine 5'-phosphate oxidase (EC 1.4.3.5)
880cb195f659b241621dd44fb21c4c7d	fig|675517.3.peg.3540	Pyridoxamine 5'-phosphate oxidase (EC 1.4.3.5)
880cb195f659b241621dd44fb21c4c7d	fig|675518.3.peg.2449	Pyridoxamine 5'-phosphate oxidase (EC 1.4.3.5)
880cb195f659b241621dd44fb21c4c7d	fig|675519.3.peg.3522	Pyridoxamine 5'-phosphate oxidase (EC 1.4.3.5)
880cb195f659b241621dd44fb21c4c7d	fig|675521.3.peg.2776	Pyridoxamine 5'-phosphate oxidase (EC 1.4.3.5)
880cb195f659b241621dd44fb21c4c7d	fig|675522.3.peg.2818	Pyridoxamine 5'-phosphate oxidase (EC 1.4.3.5)
880cb195f659b241621dd44fb21c4c7d	fig|675523.3.peg.3137	Pyridoxamine 5'-phosphate oxidase (EC 1.4.3.5)
880cb195f659b241621dd44fb21c4c7d	fig|83331.1.peg.2775	Pyridoxamine 5'-phosphate oxidase (EC 1.4.3.5)
880cb195f659b241621dd44fb21c4c7d	fig|83331.22.peg.2925	Pyridoxamine 5'-phosphate oxidase (EC 1.4.3.5)
880cb195f659b241621dd44fb21c4c7d	fig|83332.12.peg.2917	Pyridoxamine 5'-phosphate oxidase (EC 1.4.3.5)
880cb195f659b241621dd44fb21c4c7d	fig|83332.1.peg.2609	Pyridoxamine 5'-phosphate oxidase (EC 1.4.3.5)
ead48e902887ead0d73b75039853439e	fig|199310.1.peg.1309	Ribosomal large subunit pseudouridine synthase C (EC 4.2.1.70) ## LSU Psi955, Psi2504 and Psi2580
ead48e902887ead0d73b75039853439e	fig|199310.4.peg.1287	Ribosomal large subunit pseudouridine synthase C (EC 4.2.1.70) ## LSU Psi955, Psi2504 and Psi2580
ead48e902887ead0d73b75039853439e	fig|525281.3.peg.390	Ribosomal large subunit pseudouridine synthase C (EC 4.2.1.70) ## LSU Psi955, Psi2504 and Psi2580
ead48e902887ead0d73b75039853439e	fig|655817.3.peg.1373	Ribosomal large subunit pseudouridine synthase C (EC 4.2.1.70) ## LSU Psi955, Psi2504 and Psi2580
ead48e902887ead0d73b75039853439e	fig|656440.3.peg.999	Ribosomal large subunit pseudouridine synthase C (EC 4.2.1.70) ## LSU Psi955, Psi2504 and Psi2580
ead48e902887ead0d73b75039853439e	fig|749528.3.peg.729	Ribosomal large subunit pseudouridine synthase C (EC 4.2.1.70) ## LSU Psi955, Psi2504 and Psi2580
ead48e902887ead0d73b75039853439e	fig|749546.3.peg.4810	Ribosomal large subunit pseudouridine synthase C (EC 4.2.1.70) ## LSU Psi955, Psi2504 and Psi2580
3f04cf37ab5fc835a48e336ed18a4098	fig|216597.6.peg.2229	Cobalt-precorrin-2 C20-methyltransferase (EC 2.1.1.130)
3f04cf37ab5fc835a48e336ed18a4098	fig|28901.42.peg.1991	Cobalt-precorrin-2 C20-methyltransferase (EC 2.1.1.130)
3f04cf37ab5fc835a48e336ed18a4098	fig|423368.6.peg.2311	Cobalt-precorrin-2 C20-methyltransferase (EC 2.1.1.130)
3f04cf37ab5fc835a48e336ed18a4098	fig|423368.8.peg.2291	Cobalt-precorrin-2 C20-methyltransferase (EC 2.1.1.130)
3f04cf37ab5fc835a48e336ed18a4098	fig|439846.4.peg.2557	Cobalt-precorrin-2 C20-methyltransferase (EC 2.1.1.130)
3f04cf37ab5fc835a48e336ed18a4098	fig|440534.5.peg.1020	Cobalt-precorrin-2 C20-methyltransferase (EC 2.1.1.130)
3f04cf37ab5fc835a48e336ed18a4098	fig|454167.5.peg.2351	Cobalt-precorrin-2 C20-methyltransferase (EC 2.1.1.130)
3f04cf37ab5fc835a48e336ed18a4098	fig|465516.5.peg.210	Cobalt-precorrin-2 C20-methyltransferase (EC 2.1.1.130)
3f04cf37ab5fc835a48e336ed18a4098	fig|568708.3.peg.2173	Cobalt-precorrin-2 C20-methyltransferase (EC 2.1.1.130)
3f04cf37ab5fc835a48e336ed18a4098	fig|588858.6.peg.2349	Cobalt-precorrin-2 C20-methyltransferase (EC 2.1.1.130)
3f04cf37ab5fc835a48e336ed18a4098	fig|99287.12.peg.2146	Cobalt-precorrin-2 C20-methyltransferase (EC 2.1.1.130)
3f04cf37ab5fc835a48e336ed18a4098	fig|99287.1.peg.1949	Cobalt-precorrin-2 C20-methyltransferase (EC 2.1.1.130)
b9fbb044f23fb2680cccd796ccce5885	fig|754252.3.peg.1211	Cobyrinic acid A,C-diamide synthase
67dd31f4066279851a09c50d4ff597d8	fig|208963.12.peg.4582	Chromate transport protein ChrA
67dd31f4066279851a09c50d4ff597d8	fig|208963.3.peg.1845	Chromate transport protein ChrA
2e4bc6fda5935a51d4995315157b7789	fig|10090.3.peg.25709	Kynurenine formamidase (EC 3.5.1.9)
e970c8795336d74cf934714bd57ecce7	fig|9606.3.peg.8199	D-amino-acid oxidase (EC 1.4.3.3)
3607f6f9c208adc97dedb2d49bc8d3c3	fig|3702.1.peg.21603	Isocitrate dehydrogenase [NAD] subunit I, mitochondrial precursor (EC 1.1.1.41)
3607f6f9c208adc97dedb2d49bc8d3c3	fig|3702.7.peg.2506	Isocitrate dehydrogenase [NAD] (EC 1.1.1.41)
bf5f9b71b5e04692aae928a18f2e4dd9	fig|3702.1.peg.23790	Biotin carboxyl carrier protein
bf5f9b71b5e04692aae928a18f2e4dd9	fig|3702.7.peg.13005	Biotin carboxyl carrier protein of acetyl-CoA carboxylase
946b252864ef5bf887ea03370b63e7e8	fig|216597.6.peg.2358	Phosphomethylpyrimidine kinase (EC 2.7.4.7)
946b252864ef5bf887ea03370b63e7e8	fig|272994.5.peg.744	Phosphomethylpyrimidine kinase (EC 2.7.4.7)
946b252864ef5bf887ea03370b63e7e8	fig|272994.6.peg.731	Phosphomethylpyrimidine kinase (EC 2.7.4.7)
946b252864ef5bf887ea03370b63e7e8	fig|28901.42.peg.2122	Phosphomethylpyrimidine kinase (EC 2.7.4.7)
946b252864ef5bf887ea03370b63e7e8	fig|439846.4.peg.2683	Phosphomethylpyrimidine kinase (EC 2.7.4.7)
946b252864ef5bf887ea03370b63e7e8	fig|440534.5.peg.1147	Phosphomethylpyrimidine kinase (EC 2.7.4.7)
946b252864ef5bf887ea03370b63e7e8	fig|454164.6.peg.1970	Phosphomethylpyrimidine kinase (EC 2.7.4.7)
946b252864ef5bf887ea03370b63e7e8	fig|454169.6.peg.2398	Phosphomethylpyrimidine kinase (EC 2.7.4.7)
946b252864ef5bf887ea03370b63e7e8	fig|454169.8.peg.2293	Phosphomethylpyrimidine kinase (EC 2.7.4.7)
946b252864ef5bf887ea03370b63e7e8	fig|465516.5.peg.63	Phosphomethylpyrimidine kinase (EC 2.7.4.7)
946b252864ef5bf887ea03370b63e7e8	fig|465517.10.peg.2785	Phosphomethylpyrimidine kinase (EC 2.7.4.7)
946b252864ef5bf887ea03370b63e7e8	fig|568708.3.peg.2299	Phosphomethylpyrimidine kinase (EC 2.7.4.7)
946b252864ef5bf887ea03370b63e7e8	fig|588858.6.peg.2475	Phosphomethylpyrimidine kinase (EC 2.7.4.7)
946b252864ef5bf887ea03370b63e7e8	fig|99287.12.peg.2271	Phosphomethylpyrimidine kinase (EC 2.7.4.7)
946b252864ef5bf887ea03370b63e7e8	fig|99287.1.peg.2071	Phosphomethylpyrimidine kinase (EC 2.7.4.7)
d9de303ae2074b98d705aed5e46516c1	fig|199310.4.peg.1311	NADH dehydrogenase (EC 1.6.99.3)
d9de303ae2074b98d705aed5e46516c1	fig|340197.5.peg.3317	NADH dehydrogenase (EC 1.6.99.3)
d9de303ae2074b98d705aed5e46516c1	fig|358708.5.peg.3368	NADH dehydrogenase (EC 1.6.99.3)
d9de303ae2074b98d705aed5e46516c1	fig|362663.8.peg.1122	NADH dehydrogenase (EC 1.6.99.3)
d9de303ae2074b98d705aed5e46516c1	fig|362663.9.peg.1122	NADH dehydrogenase (EC 1.6.99.3)
d9de303ae2074b98d705aed5e46516c1	fig|364106.7.peg.1313	NADH dehydrogenase (EC 1.6.99.3)
d9de303ae2074b98d705aed5e46516c1	fig|364106.8.peg.1312	NADH dehydrogenase (EC 1.6.99.3)
d9de303ae2074b98d705aed5e46516c1	fig|405955.13.peg.1113	NADH dehydrogenase (EC 1.6.99.3)
d9de303ae2074b98d705aed5e46516c1	fig|431946.3.peg.1058	NADH dehydrogenase (EC 1.6.99.3)
d9de303ae2074b98d705aed5e46516c1	fig|469598.5.peg.829	NADH dehydrogenase (EC 1.6.99.3)
d9de303ae2074b98d705aed5e46516c1	fig|525281.3.peg.366	NADH dehydrogenase (EC 1.6.99.3)
d9de303ae2074b98d705aed5e46516c1	fig|585035.6.peg.1130	NADH dehydrogenase (EC 1.6.99.3)
d9de303ae2074b98d705aed5e46516c1	fig|585397.7.peg.1264	NADH dehydrogenase (EC 1.6.99.3)
d9de303ae2074b98d705aed5e46516c1	fig|585397.9.peg.1260	NADH dehydrogenase (EC 1.6.99.3)
d9de303ae2074b98d705aed5e46516c1	fig|655817.3.peg.1397	NADH dehydrogenase (EC 1.6.99.3)
d9de303ae2074b98d705aed5e46516c1	fig|656393.3.peg.1885	NADH dehydrogenase (EC 1.6.99.3)
d9de303ae2074b98d705aed5e46516c1	fig|656437.3.peg.1254	NADH dehydrogenase (EC 1.6.99.3)
d9de303ae2074b98d705aed5e46516c1	fig|685038.3.peg.1078	NADH dehydrogenase (EC 1.6.99.3)
d9de303ae2074b98d705aed5e46516c1	fig|714962.3.peg.1240	NADH dehydrogenase (EC 1.6.99.3)
d9de303ae2074b98d705aed5e46516c1	fig|749528.3.peg.753	NADH dehydrogenase (EC 1.6.99.3)
d9de303ae2074b98d705aed5e46516c1	fig|749546.3.peg.4786	NADH dehydrogenase (EC 1.6.99.3)
d9de303ae2074b98d705aed5e46516c1	fig|749550.3.peg.2148	NADH dehydrogenase (EC 1.6.99.3)
d9de303ae2074b98d705aed5e46516c1	fig|753642.3.peg.2202	NADH dehydrogenase (EC 1.6.99.3)
d9de303ae2074b98d705aed5e46516c1	fig|869729.3.peg.2511	NADH dehydrogenase (EC 1.6.99.3)
6f91688466d73c4782eaf6eca809e2fc	fig|155864.1.peg.1943	Trans-aconitate 2-methyltransferase (EC 2.1.1.144)
6f91688466d73c4782eaf6eca809e2fc	fig|155864.8.peg.1765	Trans-aconitate 2-methyltransferase (EC 2.1.1.144)
6f91688466d73c4782eaf6eca809e2fc	fig|386585.9.peg.2232	Trans-aconitate 2-methyltransferase (EC 2.1.1.144)
6f91688466d73c4782eaf6eca809e2fc	fig|444447.5.peg.5629	Trans-aconitate 2-methyltransferase (EC 2.1.1.144)
6f91688466d73c4782eaf6eca809e2fc	fig|444448.5.peg.4719	Trans-aconitate 2-methyltransferase (EC 2.1.1.144)
6f91688466d73c4782eaf6eca809e2fc	fig|444449.5.peg.360	Trans-aconitate 2-methyltransferase (EC 2.1.1.144)
6f91688466d73c4782eaf6eca809e2fc	fig|444450.8.peg.2178	Trans-aconitate 2-methyltransferase (EC 2.1.1.144)
6f91688466d73c4782eaf6eca809e2fc	fig|444451.5.peg.1894	Trans-aconitate 2-methyltransferase (EC 2.1.1.144)
6f91688466d73c4782eaf6eca809e2fc	fig|444452.5.peg.1901	Trans-aconitate 2-methyltransferase (EC 2.1.1.144)
6f91688466d73c4782eaf6eca809e2fc	fig|444453.5.peg.2915	Trans-aconitate 2-methyltransferase (EC 2.1.1.144)
6f91688466d73c4782eaf6eca809e2fc	fig|444454.5.peg.1035	Trans-aconitate 2-methyltransferase (EC 2.1.1.144)
6f91688466d73c4782eaf6eca809e2fc	fig|478004.5.peg.2904	Trans-aconitate 2-methyltransferase (EC 2.1.1.144)
6f91688466d73c4782eaf6eca809e2fc	fig|478005.5.peg.2920	Trans-aconitate 2-methyltransferase (EC 2.1.1.144)
6f91688466d73c4782eaf6eca809e2fc	fig|478006.5.peg.1887	Trans-aconitate 2-methyltransferase (EC 2.1.1.144)
6f91688466d73c4782eaf6eca809e2fc	fig|478007.5.peg.2091	Trans-aconitate 2-methyltransferase (EC 2.1.1.144)
6f91688466d73c4782eaf6eca809e2fc	fig|502346.5.peg.5240	Trans-aconitate 2-methyltransferase (EC 2.1.1.144)
6f91688466d73c4782eaf6eca809e2fc	fig|544404.4.peg.2041	Trans-aconitate 2-methyltransferase (EC 2.1.1.144)
6f91688466d73c4782eaf6eca809e2fc	fig|562.371.peg.1681	Trans-aconitate 2-methyltransferase (EC 2.1.1.144)
6f91688466d73c4782eaf6eca809e2fc	fig|562.372.peg.1166	Trans-aconitate 2-methyltransferase (EC 2.1.1.144)
6f91688466d73c4782eaf6eca809e2fc	fig|562.373.peg.5027	Trans-aconitate 2-methyltransferase (EC 2.1.1.144)
6f91688466d73c4782eaf6eca809e2fc	fig|562.374.peg.2418	Trans-aconitate 2-methyltransferase (EC 2.1.1.144)
6f91688466d73c4782eaf6eca809e2fc	fig|83334.1.peg.2153	Trans-aconitate 2-methyltransferase (EC 2.1.1.144)
7d0432115166265cc2747384916301c0	fig|4896.1.peg.1030	Isopentenyl-diphosphate delta-isomerase (EC 5.3.3.2)
3f891fd2fa175397656369a95e76a1a3	fig|316385.5.peg.1822	Vitamin B12 ABC transporter, ATPase component BtuD
3f891fd2fa175397656369a95e76a1a3	fig|316385.7.peg.1868	Vitamin B12 ABC transporter, ATPase component BtuD
3f891fd2fa175397656369a95e76a1a3	fig|316407.3.peg.1665	Vitamin B12 ABC transporter, ATPase component BtuD
3f891fd2fa175397656369a95e76a1a3	fig|511145.12.peg.1780	Vitamin B12 ABC transporter, ATPase component BtuD
3f891fd2fa175397656369a95e76a1a3	fig|511145.6.peg.1765	Vitamin B12 ABC transporter, ATPase component BtuD
3f891fd2fa175397656369a95e76a1a3	fig|536056.3.peg.2044	Vitamin B12 ABC transporter, ATPase component BtuD
3f891fd2fa175397656369a95e76a1a3	fig|595496.3.peg.1670	Vitamin B12 ABC transporter, ATPase component BtuD
3f891fd2fa175397656369a95e76a1a3	fig|83333.1.peg.1693	Vitamin B12 ABC transporter, ATPase component BtuD
8a2d4bdfd18bf8b30cf5426d05a5b344	fig|316385.5.peg.1002	Membrane alanine aminopeptidase N (EC 3.4.11.2)
8a2d4bdfd18bf8b30cf5426d05a5b344	fig|316385.7.peg.1017	Membrane alanine aminopeptidase N (EC 3.4.11.2)
8a2d4bdfd18bf8b30cf5426d05a5b344	fig|316407.3.peg.898	Membrane alanine aminopeptidase N (EC 3.4.11.2)
8a2d4bdfd18bf8b30cf5426d05a5b344	fig|331112.3.peg.971	Membrane alanine aminopeptidase N (EC 3.4.11.2)
8a2d4bdfd18bf8b30cf5426d05a5b344	fig|331112.6.peg.1010	Membrane alanine aminopeptidase N (EC 3.4.11.2)
8a2d4bdfd18bf8b30cf5426d05a5b344	fig|358709.5.peg.3450	Membrane alanine aminopeptidase N (EC 3.4.11.2)
8a2d4bdfd18bf8b30cf5426d05a5b344	fig|413997.3.peg.983	Membrane alanine aminopeptidase N (EC 3.4.11.2)
8a2d4bdfd18bf8b30cf5426d05a5b344	fig|457401.3.peg.1262	Membrane alanine aminopeptidase N (EC 3.4.11.2)
8a2d4bdfd18bf8b30cf5426d05a5b344	fig|469008.4.peg.2754	Membrane alanine aminopeptidase N (EC 3.4.11.2)
8a2d4bdfd18bf8b30cf5426d05a5b344	fig|511145.12.peg.966	Membrane alanine aminopeptidase N (EC 3.4.11.2)
8a2d4bdfd18bf8b30cf5426d05a5b344	fig|511145.6.peg.959	Membrane alanine aminopeptidase N (EC 3.4.11.2)
8a2d4bdfd18bf8b30cf5426d05a5b344	fig|511693.5.peg.1007	Membrane alanine aminopeptidase N (EC 3.4.11.2)
8a2d4bdfd18bf8b30cf5426d05a5b344	fig|536056.3.peg.2860	Membrane alanine aminopeptidase N (EC 3.4.11.2)
8a2d4bdfd18bf8b30cf5426d05a5b344	fig|595496.3.peg.865	Membrane alanine aminopeptidase N (EC 3.4.11.2)
8a2d4bdfd18bf8b30cf5426d05a5b344	fig|656414.3.peg.1152	Membrane alanine aminopeptidase N (EC 3.4.11.2)
8a2d4bdfd18bf8b30cf5426d05a5b344	fig|749538.3.peg.2841	Membrane alanine aminopeptidase N (EC 3.4.11.2)
8a2d4bdfd18bf8b30cf5426d05a5b344	fig|749540.3.peg.126	Membrane alanine aminopeptidase N (EC 3.4.11.2)
8a2d4bdfd18bf8b30cf5426d05a5b344	fig|749544.3.peg.1239	Membrane alanine aminopeptidase N (EC 3.4.11.2)
8a2d4bdfd18bf8b30cf5426d05a5b344	fig|749548.3.peg.1440	Membrane alanine aminopeptidase N (EC 3.4.11.2)
8a2d4bdfd18bf8b30cf5426d05a5b344	fig|83333.1.peg.917	Membrane alanine aminopeptidase N (EC 3.4.11.2)
c96e8785bb2786aa98d65d387d21827c	fig|4932.3.peg.6639	Phosphoadenylyl-sulfate reductase [thioredoxin] (EC 1.8.4.8)
c96e8785bb2786aa98d65d387d21827c	fig|559292.3.peg.5829	Phosphoadenylyl-sulfate reductase [thioredoxin] (EC 1.8.4.8)
e15cb0b6fa18a363e0a0a5dc0ed0a343	fig|10090.3.peg.30413	tRNA (5-methylaminomethyl-2-thiouridylate)-methyltransferase (EC 2.1.1.61)
411405b610696c93d210c34800289bf8	fig|316385.7.peg.2607	Cysteine synthase B (EC 2.5.1.47)
411405b610696c93d210c34800289bf8	fig|316401.4.peg.2896	Cysteine synthase B (EC 2.5.1.47)
411405b610696c93d210c34800289bf8	fig|316407.3.peg.2351	Cysteine synthase B (EC 2.5.1.47)
411405b610696c93d210c34800289bf8	fig|511145.12.peg.2516	Cysteine synthase B (EC 2.5.1.47)
411405b610696c93d210c34800289bf8	fig|511145.6.peg.2501	Cysteine synthase B (EC 2.5.1.47)
411405b610696c93d210c34800289bf8	fig|536056.3.peg.1304	Cysteine synthase B (EC 2.5.1.47)
411405b610696c93d210c34800289bf8	fig|595496.3.peg.2397	Cysteine synthase B (EC 2.5.1.47)
411405b610696c93d210c34800289bf8	fig|749538.3.peg.4803	Cysteine synthase B (EC 2.5.1.47)
411405b610696c93d210c34800289bf8	fig|83333.1.peg.2389	Cysteine synthase B (EC 2.5.1.47)
015f05d8533aad86820e8f7385ab6602	fig|208964.12.peg.5249	Lipopolysaccharide core biosynthesis protein WaaP (EC 2.7.-.-), heptosyl-I-kinase
015f05d8533aad86820e8f7385ab6602	fig|208964.1.peg.5006	Lipopolysaccharide core biosynthesis protein WaaP (EC 2.7.-.-), heptosyl-I-kinase
80831e5897dcdbfa195d6398bb4b3eea	fig|199310.1.peg.769	Citrate synthase (si) (EC 2.3.3.1)
80831e5897dcdbfa195d6398bb4b3eea	fig|199310.4.peg.761	Citrate synthase (si) (EC 2.3.3.1)
80831e5897dcdbfa195d6398bb4b3eea	fig|216592.1.peg.468	Citrate synthase (si) (EC 2.3.3.1)
80831e5897dcdbfa195d6398bb4b3eea	fig|216592.3.peg.771	Citrate synthase (si) (EC 2.3.3.1)
80831e5897dcdbfa195d6398bb4b3eea	fig|216593.1.peg.2911	Citrate synthase (si) (EC 2.3.3.1)
80831e5897dcdbfa195d6398bb4b3eea	fig|216599.1.peg.5035	Citrate synthase (si) (EC 2.3.3.1)
80831e5897dcdbfa195d6398bb4b3eea	fig|300268.10.peg.833	Citrate synthase (si) (EC 2.3.3.1)
80831e5897dcdbfa195d6398bb4b3eea	fig|300268.11.peg.844	Citrate synthase (si) (EC 2.3.3.1)
80831e5897dcdbfa195d6398bb4b3eea	fig|300269.11.peg.742	Citrate synthase (si) (EC 2.3.3.1)
80831e5897dcdbfa195d6398bb4b3eea	fig|300269.12.peg.770	Citrate synthase (si) (EC 2.3.3.1)
80831e5897dcdbfa195d6398bb4b3eea	fig|316385.5.peg.783	Citrate synthase (si) (EC 2.3.3.1)
80831e5897dcdbfa195d6398bb4b3eea	fig|316385.7.peg.795	Citrate synthase (si) (EC 2.3.3.1)
80831e5897dcdbfa195d6398bb4b3eea	fig|316401.4.peg.849	Citrate synthase (si) (EC 2.3.3.1)
80831e5897dcdbfa195d6398bb4b3eea	fig|316407.3.peg.694	Citrate synthase (si) (EC 2.3.3.1)
80831e5897dcdbfa195d6398bb4b3eea	fig|331111.12.peg.1042	Citrate synthase (si) (EC 2.3.3.1)
80831e5897dcdbfa195d6398bb4b3eea	fig|331111.3.peg.3261	Citrate synthase (si) (EC 2.3.3.1)
80831e5897dcdbfa195d6398bb4b3eea	fig|331112.3.peg.714	Citrate synthase (si) (EC 2.3.3.1)
80831e5897dcdbfa195d6398bb4b3eea	fig|331112.6.peg.745	Citrate synthase (si) (EC 2.3.3.1)
80831e5897dcdbfa195d6398bb4b3eea	fig|340184.3.peg.2280	Citrate synthase (si) (EC 2.3.3.1)
80831e5897dcdbfa195d6398bb4b3eea	fig|340184.6.peg.2394	Citrate synthase (si) (EC 2.3.3.1)
80831e5897dcdbfa195d6398bb4b3eea	fig|340185.3.peg.3909	Citrate synthase (si) (EC 2.3.3.1)
80831e5897dcdbfa195d6398bb4b3eea	fig|340185.4.peg.4119	Citrate synthase (si) (EC 2.3.3.1)
80831e5897dcdbfa195d6398bb4b3eea	fig|340186.3.peg.3853	Citrate synthase (si) (EC 2.3.3.1)
80831e5897dcdbfa195d6398bb4b3eea	fig|340186.5.peg.4046	Citrate synthase (si) (EC 2.3.3.1)
80831e5897dcdbfa195d6398bb4b3eea	fig|340197.3.peg.4187	Citrate synthase (si) (EC 2.3.3.1)
80831e5897dcdbfa195d6398bb4b3eea	fig|340197.5.peg.4369	Citrate synthase (si) (EC 2.3.3.1)
80831e5897dcdbfa195d6398bb4b3eea	fig|344601.3.peg.3386	Citrate synthase (si) (EC 2.3.3.1)
80831e5897dcdbfa195d6398bb4b3eea	fig|344601.5.peg.3543	Citrate synthase (si) (EC 2.3.3.1)
80831e5897dcdbfa195d6398bb4b3eea	fig|344609.11.peg.732	Citrate synthase (si) (EC 2.3.3.1)
80831e5897dcdbfa195d6398bb4b3eea	fig|344609.3.peg.4424	Citrate synthase (si) (EC 2.3.3.1)
80831e5897dcdbfa195d6398bb4b3eea	fig|344610.3.peg.4408	Citrate synthase (si) (EC 2.3.3.1)
80831e5897dcdbfa195d6398bb4b3eea	fig|344610.7.peg.965	Citrate synthase (si) (EC 2.3.3.1)
80831e5897dcdbfa195d6398bb4b3eea	fig|358708.5.peg.3739	Citrate synthase (si) (EC 2.3.3.1)
80831e5897dcdbfa195d6398bb4b3eea	fig|358709.5.peg.3963	Citrate synthase (si) (EC 2.3.3.1)
80831e5897dcdbfa195d6398bb4b3eea	fig|362663.8.peg.743	Citrate synthase (si) (EC 2.3.3.1)
80831e5897dcdbfa195d6398bb4b3eea	fig|362663.9.peg.743	Citrate synthase (si) (EC 2.3.3.1)
80831e5897dcdbfa195d6398bb4b3eea	fig|364106.7.peg.812	Citrate synthase (si) (EC 2.3.3.1)
80831e5897dcdbfa195d6398bb4b3eea	fig|364106.8.peg.811	Citrate synthase (si) (EC 2.3.3.1)
80831e5897dcdbfa195d6398bb4b3eea	fig|405955.13.peg.683	Citrate synthase (si) (EC 2.3.3.1)
80831e5897dcdbfa195d6398bb4b3eea	fig|405955.9.peg.568	Citrate synthase (si) (EC 2.3.3.1)
80831e5897dcdbfa195d6398bb4b3eea	fig|409438.11.peg.905	Citrate synthase (si) (EC 2.3.3.1)
80831e5897dcdbfa195d6398bb4b3eea	fig|413997.3.peg.723	Citrate synthase (si) (EC 2.3.3.1)
80831e5897dcdbfa195d6398bb4b3eea	fig|439855.10.peg.893	Citrate synthase (si) (EC 2.3.3.1)
80831e5897dcdbfa195d6398bb4b3eea	fig|457401.3.peg.1795	Citrate synthase (si) (EC 2.3.3.1)
80831e5897dcdbfa195d6398bb4b3eea	fig|469008.4.peg.3036	Citrate synthase (si) (EC 2.3.3.1)
80831e5897dcdbfa195d6398bb4b3eea	fig|469598.5.peg.296	Citrate synthase (si) (EC 2.3.3.1)
80831e5897dcdbfa195d6398bb4b3eea	fig|481805.3.peg.3147	Citrate synthase (si) (EC 2.3.3.1)
80831e5897dcdbfa195d6398bb4b3eea	fig|481805.6.peg.3132	Citrate synthase (si) (EC 2.3.3.1)
80831e5897dcdbfa195d6398bb4b3eea	fig|511145.12.peg.750	Citrate synthase (si) (EC 2.3.3.1)
80831e5897dcdbfa195d6398bb4b3eea	fig|511145.6.peg.741	Citrate synthase (si) (EC 2.3.3.1)
80831e5897dcdbfa195d6398bb4b3eea	fig|511693.5.peg.720	Citrate synthase (si) (EC 2.3.3.1)
80831e5897dcdbfa195d6398bb4b3eea	fig|525281.3.peg.2231	Citrate synthase (si) (EC 2.3.3.1)
80831e5897dcdbfa195d6398bb4b3eea	fig|536056.3.peg.3077	Citrate synthase (si) (EC 2.3.3.1)
80831e5897dcdbfa195d6398bb4b3eea	fig|550672.3.peg.963	Citrate synthase (si) (EC 2.3.3.1)
80831e5897dcdbfa195d6398bb4b3eea	fig|550677.3.peg.1125	Citrate synthase (si) (EC 2.3.3.1)
80831e5897dcdbfa195d6398bb4b3eea	fig|556266.3.peg.4052	Citrate synthase (si) (EC 2.3.3.1)
80831e5897dcdbfa195d6398bb4b3eea	fig|562.375.peg.1436	Citrate synthase (si) (EC 2.3.3.1)
80831e5897dcdbfa195d6398bb4b3eea	fig|562.376.peg.1909	Citrate synthase (si) (EC 2.3.3.1)
80831e5897dcdbfa195d6398bb4b3eea	fig|566546.3.peg.4580	Citrate synthase (si) (EC 2.3.3.1)
80831e5897dcdbfa195d6398bb4b3eea	fig|566546.4.peg.774	Citrate synthase (si) (EC 2.3.3.1)
80831e5897dcdbfa195d6398bb4b3eea	fig|573235.3.peg.795	Citrate synthase (si) (EC 2.3.3.1)
80831e5897dcdbfa195d6398bb4b3eea	fig|574521.7.peg.645	Citrate synthase (si) (EC 2.3.3.1)
80831e5897dcdbfa195d6398bb4b3eea	fig|585034.4.peg.690	Citrate synthase (si) (EC 2.3.3.1)
80831e5897dcdbfa195d6398bb4b3eea	fig|585034.5.peg.689	Citrate synthase (si) (EC 2.3.3.1)
80831e5897dcdbfa195d6398bb4b3eea	fig|585035.6.peg.742	Citrate synthase (si) (EC 2.3.3.1)
80831e5897dcdbfa195d6398bb4b3eea	fig|585055.6.peg.716	Citrate synthase (si) (EC 2.3.3.1)
80831e5897dcdbfa195d6398bb4b3eea	fig|585055.8.peg.718	Citrate synthase (si) (EC 2.3.3.1)
80831e5897dcdbfa195d6398bb4b3eea	fig|585056.7.peg.1002	Citrate synthase (si) (EC 2.3.3.1)
80831e5897dcdbfa195d6398bb4b3eea	fig|585395.4.peg.755	Citrate synthase (si) (EC 2.3.3.1)
80831e5897dcdbfa195d6398bb4b3eea	fig|585396.4.peg.768	Citrate synthase (si) (EC 2.3.3.1)
80831e5897dcdbfa195d6398bb4b3eea	fig|595495.4.peg.4356	Citrate synthase (si) (EC 2.3.3.1)
80831e5897dcdbfa195d6398bb4b3eea	fig|595496.3.peg.646	Citrate synthase (si) (EC 2.3.3.1)
80831e5897dcdbfa195d6398bb4b3eea	fig|621.8.peg.2114	Citrate synthase (si) (EC 2.3.3.1)
80831e5897dcdbfa195d6398bb4b3eea	fig|622.8.peg.2765	Citrate synthase (si) (EC 2.3.3.1)
80831e5897dcdbfa195d6398bb4b3eea	fig|623.7.peg.1696	Citrate synthase (si) (EC 2.3.3.1)
80831e5897dcdbfa195d6398bb4b3eea	fig|637912.3.peg.3	Citrate synthase (si) (EC 2.3.3.1)
80831e5897dcdbfa195d6398bb4b3eea	fig|655817.3.peg.804	Citrate synthase (si) (EC 2.3.3.1)
80831e5897dcdbfa195d6398bb4b3eea	fig|656379.3.peg.1494	Citrate synthase (si) (EC 2.3.3.1)
80831e5897dcdbfa195d6398bb4b3eea	fig|656380.3.peg.1322	Citrate synthase (si) (EC 2.3.3.1)
80831e5897dcdbfa195d6398bb4b3eea	fig|656393.3.peg.1405	Citrate synthase (si) (EC 2.3.3.1)
80831e5897dcdbfa195d6398bb4b3eea	fig|656414.3.peg.901	Citrate synthase (si) (EC 2.3.3.1)
80831e5897dcdbfa195d6398bb4b3eea	fig|656417.3.peg.843	Citrate synthase (si) (EC 2.3.3.1)
80831e5897dcdbfa195d6398bb4b3eea	fig|656437.3.peg.791	Citrate synthase (si) (EC 2.3.3.1)
80831e5897dcdbfa195d6398bb4b3eea	fig|656440.3.peg.571	Citrate synthase (si) (EC 2.3.3.1)
80831e5897dcdbfa195d6398bb4b3eea	fig|656443.3.peg.985	Citrate synthase (si) (EC 2.3.3.1)
80831e5897dcdbfa195d6398bb4b3eea	fig|656444.3.peg.1257	Citrate synthase (si) (EC 2.3.3.1)
80831e5897dcdbfa195d6398bb4b3eea	fig|6666666.5522.peg.4512	Citrate synthase (si) (EC 2.3.3.1)
80831e5897dcdbfa195d6398bb4b3eea	fig|670888.3.peg.1282	Citrate synthase (si) (EC 2.3.3.1)
80831e5897dcdbfa195d6398bb4b3eea	fig|670897.3.peg.867	Citrate synthase (si) (EC 2.3.3.1)
80831e5897dcdbfa195d6398bb4b3eea	fig|679204.3.peg.4496	Citrate synthase (si) (EC 2.3.3.1)
80831e5897dcdbfa195d6398bb4b3eea	fig|679205.4.peg.3673	Citrate synthase (si) (EC 2.3.3.1)
80831e5897dcdbfa195d6398bb4b3eea	fig|679206.4.peg.2770	Citrate synthase (si) (EC 2.3.3.1)
80831e5897dcdbfa195d6398bb4b3eea	fig|679207.4.peg.1374	Citrate synthase (si) (EC 2.3.3.1)
80831e5897dcdbfa195d6398bb4b3eea	fig|685038.3.peg.648	Citrate synthase (si) (EC 2.3.3.1)
80831e5897dcdbfa195d6398bb4b3eea	fig|714962.3.peg.731	Citrate synthase (si) (EC 2.3.3.1)
80831e5897dcdbfa195d6398bb4b3eea	fig|749527.3.peg.2584	Citrate synthase (si) (EC 2.3.3.1)
80831e5897dcdbfa195d6398bb4b3eea	fig|749528.3.peg.3924	Citrate synthase (si) (EC 2.3.3.1)
80831e5897dcdbfa195d6398bb4b3eea	fig|749531.3.peg.1845	Citrate synthase (si) (EC 2.3.3.1)
80831e5897dcdbfa195d6398bb4b3eea	fig|749533.3.peg.4668	Citrate synthase (si) (EC 2.3.3.1)
80831e5897dcdbfa195d6398bb4b3eea	fig|749537.3.peg.2060	Citrate synthase (si) (EC 2.3.3.1)
80831e5897dcdbfa195d6398bb4b3eea	fig|749538.3.peg.651	Citrate synthase (si) (EC 2.3.3.1)
80831e5897dcdbfa195d6398bb4b3eea	fig|749540.3.peg.4326	Citrate synthase (si) (EC 2.3.3.1)
80831e5897dcdbfa195d6398bb4b3eea	fig|749544.3.peg.3269	Citrate synthase (si) (EC 2.3.3.1)
80831e5897dcdbfa195d6398bb4b3eea	fig|749545.3.peg.3932	Citrate synthase (si) (EC 2.3.3.1)
80831e5897dcdbfa195d6398bb4b3eea	fig|749546.3.peg.176	Citrate synthase (si) (EC 2.3.3.1)
80831e5897dcdbfa195d6398bb4b3eea	fig|749547.3.peg.1734	Citrate synthase (si) (EC 2.3.3.1)
80831e5897dcdbfa195d6398bb4b3eea	fig|749548.3.peg.3224	Citrate synthase (si) (EC 2.3.3.1)
80831e5897dcdbfa195d6398bb4b3eea	fig|749549.3.peg.4484	Citrate synthase (si) (EC 2.3.3.1)
80831e5897dcdbfa195d6398bb4b3eea	fig|749550.3.peg.4735	Citrate synthase (si) (EC 2.3.3.1)
80831e5897dcdbfa195d6398bb4b3eea	fig|753642.3.peg.944	Citrate synthase (si) (EC 2.3.3.1)
80831e5897dcdbfa195d6398bb4b3eea	fig|83333.1.peg.712	Citrate synthase (si) (EC 2.3.3.1)
80831e5897dcdbfa195d6398bb4b3eea	fig|869729.3.peg.2993	Citrate synthase (si) (EC 2.3.3.1)
5396f3a33a06a69f91053c7c025f1ff8	fig|316385.5.peg.68	UDP-N-acetylmuramoylalanyl-D-glutamyl-2,6-diaminopimelate--D-alanyl-D-alanine ligase (EC 6.3.2.10)
5396f3a33a06a69f91053c7c025f1ff8	fig|316385.7.peg.68	UDP-N-acetylmuramoylalanyl-D-glutamyl-2,6-diaminopimelate--D-alanyl-D-alanine ligase (EC 6.3.2.10)
5396f3a33a06a69f91053c7c025f1ff8	fig|316401.4.peg.97	UDP-N-acetylmuramoylalanyl-D-glutamyl-2,6-diaminopimelate--D-alanyl-D-alanine ligase (EC 6.3.2.10)
5396f3a33a06a69f91053c7c025f1ff8	fig|316407.3.peg.85	UDP-N-acetylmuramoylalanyl-D-glutamyl-2,6-diaminopimelate--D-alanyl-D-alanine ligase (EC 6.3.2.10)
5396f3a33a06a69f91053c7c025f1ff8	fig|457401.3.peg.3589	UDP-N-acetylmuramoylalanyl-D-glutamyl-2,6-diaminopimelate--D-alanyl-D-alanine ligase (EC 6.3.2.10)
5396f3a33a06a69f91053c7c025f1ff8	fig|511145.12.peg.90	UDP-N-acetylmuramoylalanyl-D-glutamyl-2,6-diaminopimelate--D-alanyl-D-alanine ligase (EC 6.3.2.10)
5396f3a33a06a69f91053c7c025f1ff8	fig|511145.6.peg.90	UDP-N-acetylmuramoylalanyl-D-glutamyl-2,6-diaminopimelate--D-alanyl-D-alanine ligase (EC 6.3.2.10)
5396f3a33a06a69f91053c7c025f1ff8	fig|536056.3.peg.3713	UDP-N-acetylmuramoylalanyl-D-glutamyl-2,6-diaminopimelate--D-alanyl-D-alanine ligase (EC 6.3.2.10)
5396f3a33a06a69f91053c7c025f1ff8	fig|595496.3.peg.91	UDP-N-acetylmuramoylalanyl-D-glutamyl-2,6-diaminopimelate--D-alanyl-D-alanine ligase (EC 6.3.2.10)
5396f3a33a06a69f91053c7c025f1ff8	fig|656414.3.peg.209	UDP-N-acetylmuramoylalanyl-D-glutamyl-2,6-diaminopimelate--D-alanyl-D-alanine ligase (EC 6.3.2.10)
5396f3a33a06a69f91053c7c025f1ff8	fig|749540.3.peg.2865	UDP-N-acetylmuramoylalanyl-D-glutamyl-2,6-diaminopimelate--D-alanyl-D-alanine ligase (EC 6.3.2.10)
5396f3a33a06a69f91053c7c025f1ff8	fig|749548.3.peg.4257	UDP-N-acetylmuramoylalanyl-D-glutamyl-2,6-diaminopimelate--D-alanyl-D-alanine ligase (EC 6.3.2.10)
5396f3a33a06a69f91053c7c025f1ff8	fig|83333.1.peg.87	UDP-N-acetylmuramoylalanyl-D-glutamyl-2,6-diaminopimelate--D-alanyl-D-alanine ligase (EC 6.3.2.10)
342fa01374681696309c2faec0bdb333	fig|416870.7.peg.587	Cystathionine beta-lyase (EC 4.4.1.8) (EC 4.4.1.1)
342fa01374681696309c2faec0bdb333	fig|416870.9.peg.603	hypothetical protein
342fa01374681696309c2faec0bdb333	fig|746361.3.peg.607	hypothetical protein
875d66f1a7f606aa2b93a04593ffb463	fig|10116.3.peg.27278	5-oxoprolinase (EC 3.5.2.9)
875d66f1a7f606aa2b93a04593ffb463	fig|10116.3.peg.27279	5-oxoprolinase (EC 3.5.2.9)
f5f5ea86f0a727fd943f676c919fdadf	fig|224308.1.peg.1945	Superoxide dismutase [Cu-Zn] (EC 1.15.1.1)
f5f5ea86f0a727fd943f676c919fdadf	fig|224308.43.peg.2056	Superoxide dismutase [Cu-Zn] precursor (EC 1.15.1.1)
f5f5ea86f0a727fd943f676c919fdadf	fig|224308.49.peg.1991	Superoxide dismutase [Cu-Zn] precursor (EC 1.15.1.1)
f5f5ea86f0a727fd943f676c919fdadf	fig|535024.3.peg.1082	Superoxide dismutase [Cu-Zn] precursor (EC 1.15.1.1)
f5f5ea86f0a727fd943f676c919fdadf	fig|535025.4.peg.3169	Superoxide dismutase [Cu-Zn] precursor (EC 1.15.1.1)
f5f5ea86f0a727fd943f676c919fdadf	fig|535026.3.peg.2108	Superoxide dismutase [Cu-Zn] precursor (EC 1.15.1.1)
f5f5ea86f0a727fd943f676c919fdadf	fig|645657.3.peg.3027	Superoxide dismutase [Cu-Zn] precursor (EC 1.15.1.1)
0091333ddb1c6782b39e0a93ea40c8aa	fig|216597.6.peg.2550	NADH-ubiquinone oxidoreductase chain G (EC 1.6.5.3)
0091333ddb1c6782b39e0a93ea40c8aa	fig|220341.7.peg.2583	NADH-ubiquinone oxidoreductase chain G (EC 1.6.5.3)
0091333ddb1c6782b39e0a93ea40c8aa	fig|28901.42.peg.2311	NADH-ubiquinone oxidoreductase chain G (EC 1.6.5.3)
0091333ddb1c6782b39e0a93ea40c8aa	fig|439842.10.peg.2130	NADH-ubiquinone oxidoreductase chain G (EC 1.6.5.3)
0091333ddb1c6782b39e0a93ea40c8aa	fig|439842.7.peg.2138	NADH-ubiquinone oxidoreductase chain G (EC 1.6.5.3)
0091333ddb1c6782b39e0a93ea40c8aa	fig|439846.4.peg.2868	NADH-ubiquinone oxidoreductase chain G (EC 1.6.5.3)
0091333ddb1c6782b39e0a93ea40c8aa	fig|440534.5.peg.4334	NADH-ubiquinone oxidoreductase chain G (EC 1.6.5.3)
0091333ddb1c6782b39e0a93ea40c8aa	fig|454164.6.peg.2155	NADH-ubiquinone oxidoreductase chain G (EC 1.6.5.3)
0091333ddb1c6782b39e0a93ea40c8aa	fig|454168.5.peg.3054	NADH-ubiquinone oxidoreductase chain G (EC 1.6.5.3)
0091333ddb1c6782b39e0a93ea40c8aa	fig|454169.6.peg.2583	NADH-ubiquinone oxidoreductase chain G (EC 1.6.5.3)
0091333ddb1c6782b39e0a93ea40c8aa	fig|454169.8.peg.2475	NADH-ubiquinone oxidoreductase chain G (EC 1.6.5.3)
0091333ddb1c6782b39e0a93ea40c8aa	fig|454231.5.peg.336	NADH-ubiquinone oxidoreductase chain G (EC 1.6.5.3)
0091333ddb1c6782b39e0a93ea40c8aa	fig|465516.5.peg.1967	NADH-ubiquinone oxidoreductase chain G (EC 1.6.5.3)
0091333ddb1c6782b39e0a93ea40c8aa	fig|465517.10.peg.2613	NADH-ubiquinone oxidoreductase chain G (EC 1.6.5.3)
0091333ddb1c6782b39e0a93ea40c8aa	fig|496064.4.peg.4880	NADH-ubiquinone oxidoreductase chain G (EC 1.6.5.3)
0091333ddb1c6782b39e0a93ea40c8aa	fig|497974.4.peg.3336	NADH-ubiquinone oxidoreductase chain G (EC 1.6.5.3)
0091333ddb1c6782b39e0a93ea40c8aa	fig|568708.3.peg.2484	NADH-ubiquinone oxidoreductase chain G (EC 1.6.5.3)
0091333ddb1c6782b39e0a93ea40c8aa	fig|588858.6.peg.2665	NADH-ubiquinone oxidoreductase chain G (EC 1.6.5.3)
0091333ddb1c6782b39e0a93ea40c8aa	fig|99287.12.peg.2460	NADH-ubiquinone oxidoreductase chain G (EC 1.6.5.3)
0091333ddb1c6782b39e0a93ea40c8aa	fig|99287.1.peg.2246	NADH-ubiquinone oxidoreductase chain G (EC 1.6.5.3)
b12d9bbfd61664759d1858a688b319e7	fig|471472.3.peg.468	3-deoxy-manno-octulosonate cytidylyltransferase (EC 2.7.7.38)
b12d9bbfd61664759d1858a688b319e7	fig|471472.4.peg.469	3-deoxy-manno-octulosonate cytidylyltransferase (EC 2.7.7.38)
b12d9bbfd61664759d1858a688b319e7	fig|471473.3.peg.470	3-deoxy-manno-octulosonate cytidylyltransferase (EC 2.7.7.38)
b12d9bbfd61664759d1858a688b319e7	fig|471473.4.peg.470	3-deoxy-manno-octulosonate cytidylyltransferase (EC 2.7.7.38)
b12d9bbfd61664759d1858a688b319e7	fig|658599.4.peg.492	3-deoxy-manno-octulosonate cytidylyltransferase (EC 2.7.7.38)
f1c68b8222fa985c57278258b943389d	fig|39947.3.peg.3701	Glutamine synthetase type II, eukaryotic (EC 6.3.1.2)
66010fe9df6da1930304dce3e914950c	fig|4932.3.peg.1710	Phosphopantothenoylcysteine synthetase (EC 6.3.2.5)
bf6d32070e3fc733825736950b1b1d65	fig|3702.1.peg.10943	ammonium transporter 2 (AMT2) / identical to SP|Q9M6N7 Ammonium transporter 2 (AtAMT2) {Arabidopsis thaliana}; go_component: plasma membrane [goid 0005886]; go_function: ammonium transporter activity [goid 0008519]; go_function: high affinity ammonium transporter activity [goid 0015398]; go_process: ammonium transport [goid 0015696]
bf6d32070e3fc733825736950b1b1d65	fig|3702.7.peg.5247	Ammonium transporter
91a60fea7d9cd125da04d7511e08baa3	fig|216597.6.peg.2232	Cobalt-precorrin-3b C17-methyltransferase
91a60fea7d9cd125da04d7511e08baa3	fig|28901.42.peg.1994	Cobalt-precorrin-3b C17-methyltransferase
91a60fea7d9cd125da04d7511e08baa3	fig|439846.4.peg.2560	Cobalt-precorrin-3b C17-methyltransferase
91a60fea7d9cd125da04d7511e08baa3	fig|440534.5.peg.1023	Cobalt-precorrin-3b C17-methyltransferase
91a60fea7d9cd125da04d7511e08baa3	fig|568708.3.peg.2176	Cobalt-precorrin-3b C17-methyltransferase
91a60fea7d9cd125da04d7511e08baa3	fig|588858.6.peg.2352	Cobalt-precorrin-3b C17-methyltransferase
91a60fea7d9cd125da04d7511e08baa3	fig|99287.12.peg.2149	Cobalt-precorrin-3b C17-methyltransferase
91a60fea7d9cd125da04d7511e08baa3	fig|99287.1.peg.1952	Cobalt-precorrin-3b C17-methyltransferase
fb08b78e93d13a825038fa164ede74e8	fig|272630.7.peg.1718	Methylene tetrahydromethanopterin dehydrogenase (EC 1.5.99.9)
fb08b78e93d13a825038fa164ede74e8	fig|440085.6.peg.2072	Methylene tetrahydromethanopterin dehydrogenase (EC 1.5.99.9)
fb08b78e93d13a825038fa164ede74e8	fig|661410.3.peg.2312	Methylene tetrahydromethanopterin dehydrogenase (EC 1.5.99.9)
912e972907680d918eb4d90674a60f19	fig|29447.3.peg.2187	Alpha-L-fucosidase (EC 3.2.1.51)
50ec32fb2bee27756425d372456bf929	fig|3702.1.peg.13996	Phosphopantothenoylcysteine decarboxylase (EC 4.1.1.36)
50ec32fb2bee27756425d372456bf929	fig|3702.7.peg.21259	Phosphopantothenoylcysteine decarboxylase (EC 4.1.1.36) / Phosphopantothenoylcysteine synthetase (EC 6.3.2.5)
df27801418d37d5e61c386c4d958042c	fig|573059.3.peg.1303	Adenylylsulfate reductase alpha-subunit (EC 1.8.99.2)
df27801418d37d5e61c386c4d958042c	fig|573059.4.peg.839	Adenylylsulfate reductase alpha-subunit (EC 1.8.99.2)
df27801418d37d5e61c386c4d958042c	fig|882.1.peg.843	Adenylylsulfate reductase alpha-subunit (EC 1.8.99.2)
df27801418d37d5e61c386c4d958042c	fig|882.5.peg.791	Adenylylsulfate reductase alpha-subunit (EC 1.8.99.2)
0c1b3a8a92f75be0791f4df39d770cf1	fig|3702.1.peg.18852	Hydroxymethylglutaryl-CoA synthase (EC 2.3.3.10)
0c1b3a8a92f75be0791f4df39d770cf1	fig|3702.7.peg.10075	hydroxymethylglutaryl-CoA synthase / HMG-CoA synthase / 3-hydroxy-3-methylglutaryl coenzyme A synthase
9af4c76994556630134ddd74a1ed089d	fig|545693.3.peg.2652	Cobalt-precorrin-8x methylmutase (EC 5.4.1.2)
9af4c76994556630134ddd74a1ed089d	fig|592022.4.peg.2566	Cobalt-precorrin-8x methylmutase (EC 5.4.1.2)
1c90abad7cbdfb083e462451d8daa01e	fig|4932.3.peg.5340	Ribosylnicotinamide kinase, eukaryotic (EC 2.7.1.22)
1c90abad7cbdfb083e462451d8daa01e	fig|559292.3.peg.4665	Ribosylnicotinamide kinase, eukaryotic (EC 2.7.1.22)
d6474761a2d8f2787d17c08775c8d77c	fig|199310.1.peg.2794	3-oxoacyl-[acyl-carrier-protein] synthase, KASI (EC 2.3.1.41)
d6474761a2d8f2787d17c08775c8d77c	fig|199310.4.peg.2706	3-oxoacyl-[acyl-carrier-protein] synthase, KASI (EC 2.3.1.41)
d6474761a2d8f2787d17c08775c8d77c	fig|216592.1.peg.3148	3-oxoacyl-[acyl-carrier-protein] synthase, KASI (EC 2.3.1.41)
d6474761a2d8f2787d17c08775c8d77c	fig|216592.3.peg.2683	3-oxoacyl-[acyl-carrier-protein] synthase, KASI (EC 2.3.1.41)
d6474761a2d8f2787d17c08775c8d77c	fig|216593.1.peg.2408	3-oxoacyl-[acyl-carrier-protein] synthase, KASI (EC 2.3.1.41)
d6474761a2d8f2787d17c08775c8d77c	fig|300268.10.peg.2924	3-oxoacyl-[acyl-carrier-protein] synthase, KASI (EC 2.3.1.41)
d6474761a2d8f2787d17c08775c8d77c	fig|300268.11.peg.2955	3-oxoacyl-[acyl-carrier-protein] synthase, KASI (EC 2.3.1.41)
d6474761a2d8f2787d17c08775c8d77c	fig|316385.5.peg.2454	3-oxoacyl-[acyl-carrier-protein] synthase, KASI (EC 2.3.1.41)
d6474761a2d8f2787d17c08775c8d77c	fig|316385.7.peg.2511	3-oxoacyl-[acyl-carrier-protein] synthase, KASI (EC 2.3.1.41)
d6474761a2d8f2787d17c08775c8d77c	fig|316401.4.peg.2812	3-oxoacyl-[acyl-carrier-protein] synthase, KASI (EC 2.3.1.41)
d6474761a2d8f2787d17c08775c8d77c	fig|316407.3.peg.2259	3-oxoacyl-[acyl-carrier-protein] synthase, KASI (EC 2.3.1.41)
d6474761a2d8f2787d17c08775c8d77c	fig|331112.3.peg.2322	3-oxoacyl-[acyl-carrier-protein] synthase, KASI (EC 2.3.1.41)
d6474761a2d8f2787d17c08775c8d77c	fig|331112.6.peg.2426	3-oxoacyl-[acyl-carrier-protein] synthase, KASI (EC 2.3.1.41)
d6474761a2d8f2787d17c08775c8d77c	fig|340184.3.peg.597	3-oxoacyl-[acyl-carrier-protein] synthase, KASI (EC 2.3.1.41)
d6474761a2d8f2787d17c08775c8d77c	fig|340184.6.peg.629	3-oxoacyl-[acyl-carrier-protein] synthase, KASI (EC 2.3.1.41)
d6474761a2d8f2787d17c08775c8d77c	fig|340185.3.peg.751	3-oxoacyl-[acyl-carrier-protein] synthase, KASI (EC 2.3.1.41)
d6474761a2d8f2787d17c08775c8d77c	fig|340185.4.peg.793	3-oxoacyl-[acyl-carrier-protein] synthase, KASI (EC 2.3.1.41)
d6474761a2d8f2787d17c08775c8d77c	fig|340186.3.peg.2910	3-oxoacyl-[acyl-carrier-protein] synthase, KASI (EC 2.3.1.41)
d6474761a2d8f2787d17c08775c8d77c	fig|340186.5.peg.3026	3-oxoacyl-[acyl-carrier-protein] synthase, KASI (EC 2.3.1.41)
d6474761a2d8f2787d17c08775c8d77c	fig|340197.3.peg.389	3-oxoacyl-[acyl-carrier-protein] synthase, KASI (EC 2.3.1.41)
d6474761a2d8f2787d17c08775c8d77c	fig|340197.5.peg.399	3-oxoacyl-[acyl-carrier-protein] synthase, KASI (EC 2.3.1.41)
d6474761a2d8f2787d17c08775c8d77c	fig|344601.3.peg.2917	3-oxoacyl-[acyl-carrier-protein] synthase, KASI (EC 2.3.1.41)
d6474761a2d8f2787d17c08775c8d77c	fig|344601.5.peg.3040	3-oxoacyl-[acyl-carrier-protein] synthase, KASI (EC 2.3.1.41)
d6474761a2d8f2787d17c08775c8d77c	fig|344610.3.peg.286	3-oxoacyl-[acyl-carrier-protein] synthase, KASI (EC 2.3.1.41)
d6474761a2d8f2787d17c08775c8d77c	fig|344610.7.peg.3996	3-oxoacyl-[acyl-carrier-protein] synthase, KASI (EC 2.3.1.41)
d6474761a2d8f2787d17c08775c8d77c	fig|358709.5.peg.827	3-oxoacyl-[acyl-carrier-protein] synthase, KASI (EC 2.3.1.41)
d6474761a2d8f2787d17c08775c8d77c	fig|362663.8.peg.2389	3-oxoacyl-[acyl-carrier-protein] synthase, KASI (EC 2.3.1.41)
d6474761a2d8f2787d17c08775c8d77c	fig|362663.9.peg.2394	3-oxoacyl-[acyl-carrier-protein] synthase, KASI (EC 2.3.1.41)
d6474761a2d8f2787d17c08775c8d77c	fig|364106.7.peg.2634	3-oxoacyl-[acyl-carrier-protein] synthase, KASI (EC 2.3.1.41)
d6474761a2d8f2787d17c08775c8d77c	fig|364106.8.peg.2634	3-oxoacyl-[acyl-carrier-protein] synthase, KASI (EC 2.3.1.41)
d6474761a2d8f2787d17c08775c8d77c	fig|405955.13.peg.2548	3-oxoacyl-[acyl-carrier-protein] synthase, KASI (EC 2.3.1.41)
d6474761a2d8f2787d17c08775c8d77c	fig|405955.9.peg.2094	3-oxoacyl-[acyl-carrier-protein] synthase, KASI (EC 2.3.1.41)
d6474761a2d8f2787d17c08775c8d77c	fig|409438.11.peg.2795	3-oxoacyl-[acyl-carrier-protein] synthase, KASI (EC 2.3.1.41)
d6474761a2d8f2787d17c08775c8d77c	fig|413997.3.peg.2349	3-oxoacyl-[acyl-carrier-protein] synthase, KASI (EC 2.3.1.41)
d6474761a2d8f2787d17c08775c8d77c	fig|431946.3.peg.2299	3-oxoacyl-[acyl-carrier-protein] synthase, KASI (EC 2.3.1.41)
d6474761a2d8f2787d17c08775c8d77c	fig|439855.10.peg.2646	3-oxoacyl-[acyl-carrier-protein] synthase, KASI (EC 2.3.1.41)
d6474761a2d8f2787d17c08775c8d77c	fig|457401.3.peg.1551	3-oxoacyl-[acyl-carrier-protein] synthase, KASI (EC 2.3.1.41)
d6474761a2d8f2787d17c08775c8d77c	fig|469008.4.peg.1374	3-oxoacyl-[acyl-carrier-protein] synthase, KASI (EC 2.3.1.41)
d6474761a2d8f2787d17c08775c8d77c	fig|469598.5.peg.2919	3-oxoacyl-[acyl-carrier-protein] synthase, KASI (EC 2.3.1.41)
d6474761a2d8f2787d17c08775c8d77c	fig|481805.3.peg.1422	3-oxoacyl-[acyl-carrier-protein] synthase, KASI (EC 2.3.1.41)
d6474761a2d8f2787d17c08775c8d77c	fig|481805.6.peg.1420	3-oxoacyl-[acyl-carrier-protein] synthase, KASI (EC 2.3.1.41)
d6474761a2d8f2787d17c08775c8d77c	fig|511145.12.peg.2419	3-oxoacyl-[acyl-carrier-protein] synthase, KASI (EC 2.3.1.41)
d6474761a2d8f2787d17c08775c8d77c	fig|511145.6.peg.2403	3-oxoacyl-[acyl-carrier-protein] synthase, KASI (EC 2.3.1.41)
d6474761a2d8f2787d17c08775c8d77c	fig|511693.5.peg.2361	3-oxoacyl-[acyl-carrier-protein] synthase, KASI (EC 2.3.1.41)
d6474761a2d8f2787d17c08775c8d77c	fig|525281.3.peg.4132	3-oxoacyl-[acyl-carrier-protein] synthase, KASI (EC 2.3.1.41)
d6474761a2d8f2787d17c08775c8d77c	fig|536056.3.peg.1401	3-oxoacyl-[acyl-carrier-protein] synthase, KASI (EC 2.3.1.41)
d6474761a2d8f2787d17c08775c8d77c	fig|550672.3.peg.2038	3-oxoacyl-[acyl-carrier-protein] synthase, KASI (EC 2.3.1.41)
d6474761a2d8f2787d17c08775c8d77c	fig|550677.3.peg.1872	3-oxoacyl-[acyl-carrier-protein] synthase, KASI (EC 2.3.1.41)
d6474761a2d8f2787d17c08775c8d77c	fig|556266.3.peg.1075	3-oxoacyl-[acyl-carrier-protein] synthase, KASI (EC 2.3.1.41)
d6474761a2d8f2787d17c08775c8d77c	fig|562.375.peg.3315	3-oxoacyl-[acyl-carrier-protein] synthase, KASI (EC 2.3.1.41)
d6474761a2d8f2787d17c08775c8d77c	fig|566546.3.peg.1315	3-oxoacyl-[acyl-carrier-protein] synthase, KASI (EC 2.3.1.41)
d6474761a2d8f2787d17c08775c8d77c	fig|566546.4.peg.2496	3-oxoacyl-[acyl-carrier-protein] synthase, KASI (EC 2.3.1.41)
d6474761a2d8f2787d17c08775c8d77c	fig|573235.3.peg.3382	3-oxoacyl-[acyl-carrier-protein] synthase, KASI (EC 2.3.1.41)
d6474761a2d8f2787d17c08775c8d77c	fig|574521.7.peg.2529	3-oxoacyl-[acyl-carrier-protein] synthase, KASI (EC 2.3.1.41)
d6474761a2d8f2787d17c08775c8d77c	fig|585034.4.peg.2367	3-oxoacyl-[acyl-carrier-protein] synthase, KASI (EC 2.3.1.41)
d6474761a2d8f2787d17c08775c8d77c	fig|585034.5.peg.2365	3-oxoacyl-[acyl-carrier-protein] synthase, KASI (EC 2.3.1.41)
d6474761a2d8f2787d17c08775c8d77c	fig|585035.6.peg.2454	3-oxoacyl-[acyl-carrier-protein] synthase, KASI (EC 2.3.1.41)
d6474761a2d8f2787d17c08775c8d77c	fig|585055.6.peg.2604	3-oxoacyl-[acyl-carrier-protein] synthase, KASI (EC 2.3.1.41)
d6474761a2d8f2787d17c08775c8d77c	fig|585055.8.peg.2610	3-oxoacyl-[acyl-carrier-protein] synthase, KASI (EC 2.3.1.41)
d6474761a2d8f2787d17c08775c8d77c	fig|585056.7.peg.2846	3-oxoacyl-[acyl-carrier-protein] synthase, KASI (EC 2.3.1.41)
d6474761a2d8f2787d17c08775c8d77c	fig|585057.4.peg.2573	3-oxoacyl-[acyl-carrier-protein] synthase, KASI (EC 2.3.1.41)
d6474761a2d8f2787d17c08775c8d77c	fig|585057.6.peg.2576	3-oxoacyl-[acyl-carrier-protein] synthase, KASI (EC 2.3.1.41)
d6474761a2d8f2787d17c08775c8d77c	fig|585395.4.peg.2919	3-oxoacyl-[acyl-carrier-protein] synthase, KASI (EC 2.3.1.41)
d6474761a2d8f2787d17c08775c8d77c	fig|585396.4.peg.3165	3-oxoacyl-[acyl-carrier-protein] synthase, KASI (EC 2.3.1.41)
d6474761a2d8f2787d17c08775c8d77c	fig|585397.7.peg.2795	3-oxoacyl-[acyl-carrier-protein] synthase, KASI (EC 2.3.1.41)
d6474761a2d8f2787d17c08775c8d77c	fig|585397.9.peg.2792	3-oxoacyl-[acyl-carrier-protein] synthase, KASI (EC 2.3.1.41)
d6474761a2d8f2787d17c08775c8d77c	fig|595495.4.peg.116	3-oxoacyl-[acyl-carrier-protein] synthase, KASI (EC 2.3.1.41)
d6474761a2d8f2787d17c08775c8d77c	fig|595496.3.peg.2301	3-oxoacyl-[acyl-carrier-protein] synthase, KASI (EC 2.3.1.41)
d6474761a2d8f2787d17c08775c8d77c	fig|622.8.peg.1264	3-oxoacyl-[acyl-carrier-protein] synthase, KASI (EC 2.3.1.41)
d6474761a2d8f2787d17c08775c8d77c	fig|623.7.peg.3200	3-oxoacyl-[acyl-carrier-protein] synthase, KASI (EC 2.3.1.41)
d6474761a2d8f2787d17c08775c8d77c	fig|637912.3.peg.4346	3-oxoacyl-[acyl-carrier-protein] synthase, KASI (EC 2.3.1.41)
d6474761a2d8f2787d17c08775c8d77c	fig|655817.3.peg.2776	3-oxoacyl-[acyl-carrier-protein] synthase, KASI (EC 2.3.1.41)
d6474761a2d8f2787d17c08775c8d77c	fig|656379.3.peg.2865	3-oxoacyl-[acyl-carrier-protein] synthase, KASI (EC 2.3.1.41)
d6474761a2d8f2787d17c08775c8d77c	fig|656380.3.peg.2423	3-oxoacyl-[acyl-carrier-protein] synthase, KASI (EC 2.3.1.41)
d6474761a2d8f2787d17c08775c8d77c	fig|656393.3.peg.3358	3-oxoacyl-[acyl-carrier-protein] synthase, KASI (EC 2.3.1.41)
d6474761a2d8f2787d17c08775c8d77c	fig|656408.3.peg.2570	3-oxoacyl-[acyl-carrier-protein] synthase, KASI (EC 2.3.1.41)
d6474761a2d8f2787d17c08775c8d77c	fig|656414.3.peg.2711	3-oxoacyl-[acyl-carrier-protein] synthase, KASI (EC 2.3.1.41)
d6474761a2d8f2787d17c08775c8d77c	fig|656417.3.peg.3003	3-oxoacyl-[acyl-carrier-protein] synthase, KASI (EC 2.3.1.41)
d6474761a2d8f2787d17c08775c8d77c	fig|656437.3.peg.2610	3-oxoacyl-[acyl-carrier-protein] synthase, KASI (EC 2.3.1.41)
d6474761a2d8f2787d17c08775c8d77c	fig|656440.3.peg.2395	3-oxoacyl-[acyl-carrier-protein] synthase, KASI (EC 2.3.1.41)
d6474761a2d8f2787d17c08775c8d77c	fig|656443.3.peg.3033	3-oxoacyl-[acyl-carrier-protein] synthase, KASI (EC 2.3.1.41)
d6474761a2d8f2787d17c08775c8d77c	fig|656444.3.peg.3328	3-oxoacyl-[acyl-carrier-protein] synthase, KASI (EC 2.3.1.41)
d6474761a2d8f2787d17c08775c8d77c	fig|6666666.5522.peg.3613	3-oxoacyl-[acyl-carrier-protein] synthase, KASI (EC 2.3.1.41)
d6474761a2d8f2787d17c08775c8d77c	fig|670888.3.peg.3950	3-oxoacyl-[acyl-carrier-protein] synthase, KASI (EC 2.3.1.41)
d6474761a2d8f2787d17c08775c8d77c	fig|670897.3.peg.3301	3-oxoacyl-[acyl-carrier-protein] synthase, KASI (EC 2.3.1.41)
d6474761a2d8f2787d17c08775c8d77c	fig|679204.3.peg.358	3-oxoacyl-[acyl-carrier-protein] synthase, KASI (EC 2.3.1.41)
d6474761a2d8f2787d17c08775c8d77c	fig|679206.4.peg.1973	3-oxoacyl-[acyl-carrier-protein] synthase, KASI (EC 2.3.1.41)
d6474761a2d8f2787d17c08775c8d77c	fig|679207.4.peg.3036	3-oxoacyl-[acyl-carrier-protein] synthase, KASI (EC 2.3.1.41)
d6474761a2d8f2787d17c08775c8d77c	fig|685038.3.peg.2388	3-oxoacyl-[acyl-carrier-protein] synthase, KASI (EC 2.3.1.41)
d6474761a2d8f2787d17c08775c8d77c	fig|714962.3.peg.2655	3-oxoacyl-[acyl-carrier-protein] synthase, KASI (EC 2.3.1.41)
d6474761a2d8f2787d17c08775c8d77c	fig|749527.3.peg.1664	3-oxoacyl-[acyl-carrier-protein] synthase, KASI (EC 2.3.1.41)
d6474761a2d8f2787d17c08775c8d77c	fig|749528.3.peg.2671	3-oxoacyl-[acyl-carrier-protein] synthase, KASI (EC 2.3.1.41)
d6474761a2d8f2787d17c08775c8d77c	fig|749531.3.peg.3540	3-oxoacyl-[acyl-carrier-protein] synthase, KASI (EC 2.3.1.41)
d6474761a2d8f2787d17c08775c8d77c	fig|749532.3.peg.3097	3-oxoacyl-[acyl-carrier-protein] synthase, KASI (EC 2.3.1.41)
d6474761a2d8f2787d17c08775c8d77c	fig|749538.3.peg.1944	3-oxoacyl-[acyl-carrier-protein] synthase, KASI (EC 2.3.1.41)
d6474761a2d8f2787d17c08775c8d77c	fig|749540.3.peg.3916	3-oxoacyl-[acyl-carrier-protein] synthase, KASI (EC 2.3.1.41)
d6474761a2d8f2787d17c08775c8d77c	fig|749544.3.peg.2145	3-oxoacyl-[acyl-carrier-protein] synthase, KASI (EC 2.3.1.41)
d6474761a2d8f2787d17c08775c8d77c	fig|749545.3.peg.318	3-oxoacyl-[acyl-carrier-protein] synthase, KASI (EC 2.3.1.41)
d6474761a2d8f2787d17c08775c8d77c	fig|749546.3.peg.2759	3-oxoacyl-[acyl-carrier-protein] synthase, KASI (EC 2.3.1.41)
d6474761a2d8f2787d17c08775c8d77c	fig|749548.3.peg.1868	3-oxoacyl-[acyl-carrier-protein] synthase, KASI (EC 2.3.1.41)
d6474761a2d8f2787d17c08775c8d77c	fig|749549.3.peg.1234	3-oxoacyl-[acyl-carrier-protein] synthase, KASI (EC 2.3.1.41)
d6474761a2d8f2787d17c08775c8d77c	fig|749550.3.peg.2866	3-oxoacyl-[acyl-carrier-protein] synthase, KASI (EC 2.3.1.41)
d6474761a2d8f2787d17c08775c8d77c	fig|753642.3.peg.2759	3-oxoacyl-[acyl-carrier-protein] synthase, KASI (EC 2.3.1.41)
d6474761a2d8f2787d17c08775c8d77c	fig|83333.1.peg.2297	3-oxoacyl-[acyl-carrier-protein] synthase, KASI (EC 2.3.1.41)
d6474761a2d8f2787d17c08775c8d77c	fig|869729.3.peg.1102	3-oxoacyl-[acyl-carrier-protein] synthase, KASI (EC 2.3.1.41)
b9f03a52e78ffa0c5f3d4d4301c34388	fig|155864.1.peg.770	Cytochrome d ubiquinol oxidase subunit II (EC 1.10.3.-)
b9f03a52e78ffa0c5f3d4d4301c34388	fig|155864.8.peg.791	Cytochrome d ubiquinol oxidase subunit II (EC 1.10.3.-)
b9f03a52e78ffa0c5f3d4d4301c34388	fig|199310.1.peg.785	Cytochrome d ubiquinol oxidase subunit II (EC 1.10.3.-)
b9f03a52e78ffa0c5f3d4d4301c34388	fig|199310.4.peg.774	Cytochrome d ubiquinol oxidase subunit II (EC 1.10.3.-)
b9f03a52e78ffa0c5f3d4d4301c34388	fig|216592.1.peg.494	Cytochrome d ubiquinol oxidase subunit II (EC 1.10.3.-)
b9f03a52e78ffa0c5f3d4d4301c34388	fig|216592.3.peg.793	Cytochrome d ubiquinol oxidase subunit II (EC 1.10.3.-)
b9f03a52e78ffa0c5f3d4d4301c34388	fig|216593.1.peg.2891	Cytochrome d ubiquinol oxidase subunit II (EC 1.10.3.-)
b9f03a52e78ffa0c5f3d4d4301c34388	fig|300269.11.peg.758	Cytochrome d ubiquinol oxidase subunit II (EC 1.10.3.-)
b9f03a52e78ffa0c5f3d4d4301c34388	fig|300269.12.peg.786	Cytochrome d ubiquinol oxidase subunit II (EC 1.10.3.-)
b9f03a52e78ffa0c5f3d4d4301c34388	fig|316385.5.peg.796	Cytochrome d ubiquinol oxidase subunit II (EC 1.10.3.-)
b9f03a52e78ffa0c5f3d4d4301c34388	fig|316385.7.peg.810	Cytochrome d ubiquinol oxidase subunit II (EC 1.10.3.-)
b9f03a52e78ffa0c5f3d4d4301c34388	fig|316401.4.peg.867	Cytochrome d ubiquinol oxidase subunit II (EC 1.10.3.-)
b9f03a52e78ffa0c5f3d4d4301c34388	fig|316407.3.peg.707	Cytochrome d ubiquinol oxidase subunit II (EC 1.10.3.-)
b9f03a52e78ffa0c5f3d4d4301c34388	fig|331112.3.peg.729	Cytochrome d ubiquinol oxidase subunit II (EC 1.10.3.-)
b9f03a52e78ffa0c5f3d4d4301c34388	fig|331112.6.peg.762	Cytochrome d ubiquinol oxidase subunit II (EC 1.10.3.-)
b9f03a52e78ffa0c5f3d4d4301c34388	fig|340184.3.peg.2265	Cytochrome d ubiquinol oxidase subunit II (EC 1.10.3.-)
b9f03a52e78ffa0c5f3d4d4301c34388	fig|340184.6.peg.2379	Cytochrome d ubiquinol oxidase subunit II (EC 1.10.3.-)
b9f03a52e78ffa0c5f3d4d4301c34388	fig|340185.3.peg.3925	Cytochrome d ubiquinol oxidase subunit II (EC 1.10.3.-)
b9f03a52e78ffa0c5f3d4d4301c34388	fig|340185.4.peg.4135	Cytochrome d ubiquinol oxidase subunit II (EC 1.10.3.-)
b9f03a52e78ffa0c5f3d4d4301c34388	fig|340197.3.peg.4173	Cytochrome d ubiquinol oxidase subunit II (EC 1.10.3.-)
b9f03a52e78ffa0c5f3d4d4301c34388	fig|340197.5.peg.4355	Cytochrome d ubiquinol oxidase subunit II (EC 1.10.3.-)
b9f03a52e78ffa0c5f3d4d4301c34388	fig|344601.3.peg.3371	Cytochrome d ubiquinol oxidase subunit II (EC 1.10.3.-)
b9f03a52e78ffa0c5f3d4d4301c34388	fig|344601.5.peg.3527	Cytochrome d ubiquinol oxidase subunit II (EC 1.10.3.-)
b9f03a52e78ffa0c5f3d4d4301c34388	fig|344609.11.peg.749	Cytochrome d ubiquinol oxidase subunit II (EC 1.10.3.-)
b9f03a52e78ffa0c5f3d4d4301c34388	fig|344609.3.peg.4442	Cytochrome d ubiquinol oxidase subunit II (EC 1.10.3.-)
b9f03a52e78ffa0c5f3d4d4301c34388	fig|344610.3.peg.4422	Cytochrome d ubiquinol oxidase subunit II (EC 1.10.3.-)
b9f03a52e78ffa0c5f3d4d4301c34388	fig|344610.7.peg.952	Cytochrome d ubiquinol oxidase subunit II (EC 1.10.3.-)
b9f03a52e78ffa0c5f3d4d4301c34388	fig|358708.5.peg.3753	Cytochrome d ubiquinol oxidase subunit II (EC 1.10.3.-)
b9f03a52e78ffa0c5f3d4d4301c34388	fig|358709.5.peg.3949	Cytochrome d ubiquinol oxidase subunit II (EC 1.10.3.-)
b9f03a52e78ffa0c5f3d4d4301c34388	fig|362663.8.peg.757	Cytochrome d ubiquinol oxidase subunit II (EC 1.10.3.-)
b9f03a52e78ffa0c5f3d4d4301c34388	fig|362663.9.peg.757	Cytochrome d ubiquinol oxidase subunit II (EC 1.10.3.-)
b9f03a52e78ffa0c5f3d4d4301c34388	fig|364106.7.peg.826	Cytochrome d ubiquinol oxidase subunit II (EC 1.10.3.-)
b9f03a52e78ffa0c5f3d4d4301c34388	fig|364106.8.peg.825	Cytochrome d ubiquinol oxidase subunit II (EC 1.10.3.-)
b9f03a52e78ffa0c5f3d4d4301c34388	fig|386585.9.peg.887	Cytochrome d ubiquinol oxidase subunit II (EC 1.10.3.-)
b9f03a52e78ffa0c5f3d4d4301c34388	fig|405955.13.peg.697	Cytochrome d ubiquinol oxidase subunit II (EC 1.10.3.-)
b9f03a52e78ffa0c5f3d4d4301c34388	fig|405955.9.peg.580	Cytochrome d ubiquinol oxidase subunit II (EC 1.10.3.-)
b9f03a52e78ffa0c5f3d4d4301c34388	fig|409438.11.peg.920	Cytochrome d ubiquinol oxidase subunit II (EC 1.10.3.-)
b9f03a52e78ffa0c5f3d4d4301c34388	fig|413997.3.peg.737	Cytochrome d ubiquinol oxidase subunit II (EC 1.10.3.-)
b9f03a52e78ffa0c5f3d4d4301c34388	fig|431946.3.peg.698	Cytochrome d ubiquinol oxidase subunit II (EC 1.10.3.-)
b9f03a52e78ffa0c5f3d4d4301c34388	fig|439855.10.peg.917	Cytochrome d ubiquinol oxidase subunit II (EC 1.10.3.-)
b9f03a52e78ffa0c5f3d4d4301c34388	fig|444447.5.peg.3658	Cytochrome d ubiquinol oxidase subunit II (EC 1.10.3.-)
b9f03a52e78ffa0c5f3d4d4301c34388	fig|444448.5.peg.3474	Cytochrome d ubiquinol oxidase subunit II (EC 1.10.3.-)
b9f03a52e78ffa0c5f3d4d4301c34388	fig|444449.5.peg.5599	Cytochrome d ubiquinol oxidase subunit II (EC 1.10.3.-)
b9f03a52e78ffa0c5f3d4d4301c34388	fig|444450.8.peg.932	Cytochrome d ubiquinol oxidase subunit II (EC 1.10.3.-)
b9f03a52e78ffa0c5f3d4d4301c34388	fig|444451.5.peg.4996	Cytochrome d ubiquinol oxidase subunit II (EC 1.10.3.-)
b9f03a52e78ffa0c5f3d4d4301c34388	fig|444453.5.peg.817	Cytochrome d ubiquinol oxidase subunit II (EC 1.10.3.-)
b9f03a52e78ffa0c5f3d4d4301c34388	fig|444454.5.peg.5264	Cytochrome d ubiquinol oxidase subunit II (EC 1.10.3.-)
b9f03a52e78ffa0c5f3d4d4301c34388	fig|457401.3.peg.1781	Cytochrome d ubiquinol oxidase subunit II (EC 1.10.3.-)
b9f03a52e78ffa0c5f3d4d4301c34388	fig|469008.4.peg.3022	Cytochrome d ubiquinol oxidase subunit II (EC 1.10.3.-)
b9f03a52e78ffa0c5f3d4d4301c34388	fig|469598.5.peg.282	Cytochrome d ubiquinol oxidase subunit II (EC 1.10.3.-)
b9f03a52e78ffa0c5f3d4d4301c34388	fig|478005.5.peg.1316	Cytochrome d ubiquinol oxidase subunit II (EC 1.10.3.-)
b9f03a52e78ffa0c5f3d4d4301c34388	fig|478007.5.peg.3965	Cytochrome d ubiquinol oxidase subunit II (EC 1.10.3.-)
b9f03a52e78ffa0c5f3d4d4301c34388	fig|478008.5.peg.2162	Cytochrome d ubiquinol oxidase subunit II (EC 1.10.3.-)
b9f03a52e78ffa0c5f3d4d4301c34388	fig|481805.3.peg.3132	Cytochrome d ubiquinol oxidase subunit II (EC 1.10.3.-)
b9f03a52e78ffa0c5f3d4d4301c34388	fig|481805.6.peg.3118	Cytochrome d ubiquinol oxidase subunit II (EC 1.10.3.-)
b9f03a52e78ffa0c5f3d4d4301c34388	fig|502346.5.peg.420	Cytochrome d ubiquinol oxidase subunit II (EC 1.10.3.-)
b9f03a52e78ffa0c5f3d4d4301c34388	fig|511145.12.peg.765	Cytochrome d ubiquinol oxidase subunit II (EC 1.10.3.-)
b9f03a52e78ffa0c5f3d4d4301c34388	fig|511145.6.peg.756	Cytochrome d ubiquinol oxidase subunit II (EC 1.10.3.-)
b9f03a52e78ffa0c5f3d4d4301c34388	fig|511693.5.peg.734	Cytochrome d ubiquinol oxidase subunit II (EC 1.10.3.-)
b9f03a52e78ffa0c5f3d4d4301c34388	fig|525281.3.peg.2217	Cytochrome d ubiquinol oxidase subunit II (EC 1.10.3.-)
b9f03a52e78ffa0c5f3d4d4301c34388	fig|536056.3.peg.3062	Cytochrome d ubiquinol oxidase subunit II (EC 1.10.3.-)
b9f03a52e78ffa0c5f3d4d4301c34388	fig|544404.4.peg.798	Cytochrome d ubiquinol oxidase subunit II (EC 1.10.3.-)
b9f03a52e78ffa0c5f3d4d4301c34388	fig|550672.3.peg.976	Cytochrome d ubiquinol oxidase subunit II (EC 1.10.3.-)
b9f03a52e78ffa0c5f3d4d4301c34388	fig|550676.3.peg.200	Cytochrome d ubiquinol oxidase subunit II (EC 1.10.3.-)
b9f03a52e78ffa0c5f3d4d4301c34388	fig|550677.3.peg.1148	Cytochrome d ubiquinol oxidase subunit II (EC 1.10.3.-)
b9f03a52e78ffa0c5f3d4d4301c34388	fig|556266.3.peg.4037	Cytochrome d ubiquinol oxidase subunit II (EC 1.10.3.-)
b9f03a52e78ffa0c5f3d4d4301c34388	fig|562.371.peg.534	Cytochrome d ubiquinol oxidase subunit II (EC 1.10.3.-)
b9f03a52e78ffa0c5f3d4d4301c34388	fig|562.372.peg.1740	Cytochrome d ubiquinol oxidase subunit II (EC 1.10.3.-)
b9f03a52e78ffa0c5f3d4d4301c34388	fig|562.374.peg.5236	Cytochrome d ubiquinol oxidase subunit II (EC 1.10.3.-)
b9f03a52e78ffa0c5f3d4d4301c34388	fig|562.375.peg.1418	Cytochrome d ubiquinol oxidase subunit II (EC 1.10.3.-)
b9f03a52e78ffa0c5f3d4d4301c34388	fig|562.376.peg.1873	Cytochrome d ubiquinol oxidase subunit II (EC 1.10.3.-)
b9f03a52e78ffa0c5f3d4d4301c34388	fig|566546.3.peg.4562	Cytochrome d ubiquinol oxidase subunit II (EC 1.10.3.-)
b9f03a52e78ffa0c5f3d4d4301c34388	fig|566546.4.peg.789	Cytochrome d ubiquinol oxidase subunit II (EC 1.10.3.-)
b9f03a52e78ffa0c5f3d4d4301c34388	fig|570506.3.peg.396	Cytochrome d ubiquinol oxidase subunit II (EC 1.10.3.-)
b9f03a52e78ffa0c5f3d4d4301c34388	fig|573235.3.peg.810	Cytochrome d ubiquinol oxidase subunit II (EC 1.10.3.-)
b9f03a52e78ffa0c5f3d4d4301c34388	fig|574521.7.peg.660	Cytochrome d ubiquinol oxidase subunit II (EC 1.10.3.-)
b9f03a52e78ffa0c5f3d4d4301c34388	fig|585034.4.peg.704	Cytochrome d ubiquinol oxidase subunit II (EC 1.10.3.-)
b9f03a52e78ffa0c5f3d4d4301c34388	fig|585034.5.peg.704	Cytochrome d ubiquinol oxidase subunit II (EC 1.10.3.-)
b9f03a52e78ffa0c5f3d4d4301c34388	fig|585035.6.peg.756	Cytochrome d ubiquinol oxidase subunit II (EC 1.10.3.-)
b9f03a52e78ffa0c5f3d4d4301c34388	fig|585054.5.peg.2285	Cytochrome d ubiquinol oxidase subunit II (EC 1.10.3.-)
b9f03a52e78ffa0c5f3d4d4301c34388	fig|585055.6.peg.731	Cytochrome d ubiquinol oxidase subunit II (EC 1.10.3.-)
b9f03a52e78ffa0c5f3d4d4301c34388	fig|585055.8.peg.733	Cytochrome d ubiquinol oxidase subunit II (EC 1.10.3.-)
b9f03a52e78ffa0c5f3d4d4301c34388	fig|585056.7.peg.1025	Cytochrome d ubiquinol oxidase subunit II (EC 1.10.3.-)
b9f03a52e78ffa0c5f3d4d4301c34388	fig|585057.4.peg.754	Cytochrome d ubiquinol oxidase subunit II (EC 1.10.3.-)
b9f03a52e78ffa0c5f3d4d4301c34388	fig|585057.6.peg.751	Cytochrome d ubiquinol oxidase subunit II (EC 1.10.3.-)
b9f03a52e78ffa0c5f3d4d4301c34388	fig|585395.4.peg.771	Cytochrome d ubiquinol oxidase subunit II (EC 1.10.3.-)
b9f03a52e78ffa0c5f3d4d4301c34388	fig|585396.4.peg.784	Cytochrome d ubiquinol oxidase subunit II (EC 1.10.3.-)
b9f03a52e78ffa0c5f3d4d4301c34388	fig|585397.7.peg.708	Cytochrome d ubiquinol oxidase subunit II (EC 1.10.3.-)
b9f03a52e78ffa0c5f3d4d4301c34388	fig|585397.9.peg.708	Cytochrome d ubiquinol oxidase subunit II (EC 1.10.3.-)
b9f03a52e78ffa0c5f3d4d4301c34388	fig|595495.4.peg.4338	Cytochrome d ubiquinol oxidase subunit II (EC 1.10.3.-)
b9f03a52e78ffa0c5f3d4d4301c34388	fig|595496.3.peg.661	Cytochrome d ubiquinol oxidase subunit II (EC 1.10.3.-)
b9f03a52e78ffa0c5f3d4d4301c34388	fig|621.8.peg.2134	Cytochrome d ubiquinol oxidase subunit II (EC 1.10.3.-)
b9f03a52e78ffa0c5f3d4d4301c34388	fig|623.7.peg.5081	Cytochrome d ubiquinol oxidase subunit II (EC 1.10.3.-)
b9f03a52e78ffa0c5f3d4d4301c34388	fig|637388.3.peg.1521	Cytochrome d ubiquinol oxidase subunit II (EC 1.10.3.-)
b9f03a52e78ffa0c5f3d4d4301c34388	fig|655817.3.peg.820	Cytochrome d ubiquinol oxidase subunit II (EC 1.10.3.-)
b9f03a52e78ffa0c5f3d4d4301c34388	fig|656379.3.peg.1521	Cytochrome d ubiquinol oxidase subunit II (EC 1.10.3.-)
b9f03a52e78ffa0c5f3d4d4301c34388	fig|656380.3.peg.1346	Cytochrome d ubiquinol oxidase subunit II (EC 1.10.3.-)
b9f03a52e78ffa0c5f3d4d4301c34388	fig|656393.3.peg.1420	Cytochrome d ubiquinol oxidase subunit II (EC 1.10.3.-)
b9f03a52e78ffa0c5f3d4d4301c34388	fig|656408.3.peg.681	Cytochrome d ubiquinol oxidase subunit II (EC 1.10.3.-)
b9f03a52e78ffa0c5f3d4d4301c34388	fig|656414.3.peg.919	Cytochrome d ubiquinol oxidase subunit II (EC 1.10.3.-)
b9f03a52e78ffa0c5f3d4d4301c34388	fig|656417.3.peg.863	Cytochrome d ubiquinol oxidase subunit II (EC 1.10.3.-)
b9f03a52e78ffa0c5f3d4d4301c34388	fig|656419.3.peg.1019	Cytochrome d ubiquinol oxidase subunit II (EC 1.10.3.-)
b9f03a52e78ffa0c5f3d4d4301c34388	fig|656437.3.peg.819	Cytochrome d ubiquinol oxidase subunit II (EC 1.10.3.-)
b9f03a52e78ffa0c5f3d4d4301c34388	fig|656440.3.peg.586	Cytochrome d ubiquinol oxidase subunit II (EC 1.10.3.-)
b9f03a52e78ffa0c5f3d4d4301c34388	fig|656443.3.peg.1004	Cytochrome d ubiquinol oxidase subunit II (EC 1.10.3.-)
b9f03a52e78ffa0c5f3d4d4301c34388	fig|656444.3.peg.1285	Cytochrome d ubiquinol oxidase subunit II (EC 1.10.3.-)
b9f03a52e78ffa0c5f3d4d4301c34388	fig|6666666.5365.peg.5052	Cytochrome d ubiquinol oxidase subunit II (EC 1.10.3.-)
b9f03a52e78ffa0c5f3d4d4301c34388	fig|6666666.5522.peg.4526	Cytochrome d ubiquinol oxidase subunit II (EC 1.10.3.-)
b9f03a52e78ffa0c5f3d4d4301c34388	fig|670888.3.peg.1297	Cytochrome d ubiquinol oxidase subunit II (EC 1.10.3.-)
b9f03a52e78ffa0c5f3d4d4301c34388	fig|670897.3.peg.887	Cytochrome d ubiquinol oxidase subunit II (EC 1.10.3.-)
b9f03a52e78ffa0c5f3d4d4301c34388	fig|679204.3.peg.4514	Cytochrome d ubiquinol oxidase subunit II (EC 1.10.3.-)
b9f03a52e78ffa0c5f3d4d4301c34388	fig|679205.4.peg.3691	Cytochrome d ubiquinol oxidase subunit II (EC 1.10.3.-)
b9f03a52e78ffa0c5f3d4d4301c34388	fig|679206.4.peg.2752	Cytochrome d ubiquinol oxidase subunit II (EC 1.10.3.-)
b9f03a52e78ffa0c5f3d4d4301c34388	fig|679207.4.peg.1392	Cytochrome d ubiquinol oxidase subunit II (EC 1.10.3.-)
b9f03a52e78ffa0c5f3d4d4301c34388	fig|685038.3.peg.662	Cytochrome d ubiquinol oxidase subunit II (EC 1.10.3.-)
b9f03a52e78ffa0c5f3d4d4301c34388	fig|701177.3.peg.895	Cytochrome d ubiquinol oxidase subunit II (EC 1.10.3.-)
b9f03a52e78ffa0c5f3d4d4301c34388	fig|714962.3.peg.745	Cytochrome d ubiquinol oxidase subunit II (EC 1.10.3.-)
b9f03a52e78ffa0c5f3d4d4301c34388	fig|749528.3.peg.4472	Cytochrome d ubiquinol oxidase subunit II (EC 1.10.3.-)
b9f03a52e78ffa0c5f3d4d4301c34388	fig|749531.3.peg.1818	Cytochrome d ubiquinol oxidase subunit II (EC 1.10.3.-)
b9f03a52e78ffa0c5f3d4d4301c34388	fig|749532.3.peg.2610	Cytochrome d ubiquinol oxidase subunit II (EC 1.10.3.-)
b9f03a52e78ffa0c5f3d4d4301c34388	fig|749533.3.peg.4686	Cytochrome d ubiquinol oxidase subunit II (EC 1.10.3.-)
b9f03a52e78ffa0c5f3d4d4301c34388	fig|749537.3.peg.2078	Cytochrome d ubiquinol oxidase subunit II (EC 1.10.3.-)
b9f03a52e78ffa0c5f3d4d4301c34388	fig|749538.3.peg.632	Cytochrome d ubiquinol oxidase subunit II (EC 1.10.3.-)
b9f03a52e78ffa0c5f3d4d4301c34388	fig|749540.3.peg.4344	Cytochrome d ubiquinol oxidase subunit II (EC 1.10.3.-)
b9f03a52e78ffa0c5f3d4d4301c34388	fig|749544.3.peg.3287	Cytochrome d ubiquinol oxidase subunit II (EC 1.10.3.-)
b9f03a52e78ffa0c5f3d4d4301c34388	fig|749545.3.peg.3950	Cytochrome d ubiquinol oxidase subunit II (EC 1.10.3.-)
b9f03a52e78ffa0c5f3d4d4301c34388	fig|749546.3.peg.4774	Cytochrome d ubiquinol oxidase subunit II (EC 1.10.3.-)
b9f03a52e78ffa0c5f3d4d4301c34388	fig|749547.3.peg.1715	Cytochrome d ubiquinol oxidase subunit II (EC 1.10.3.-)
b9f03a52e78ffa0c5f3d4d4301c34388	fig|749548.3.peg.3205	Cytochrome d ubiquinol oxidase subunit II (EC 1.10.3.-)
b9f03a52e78ffa0c5f3d4d4301c34388	fig|749549.3.peg.4511	Cytochrome d ubiquinol oxidase subunit II (EC 1.10.3.-)
b9f03a52e78ffa0c5f3d4d4301c34388	fig|749550.3.peg.3835	Cytochrome d ubiquinol oxidase subunit II (EC 1.10.3.-)
b9f03a52e78ffa0c5f3d4d4301c34388	fig|753642.3.peg.930	Cytochrome d ubiquinol oxidase subunit II (EC 1.10.3.-)
b9f03a52e78ffa0c5f3d4d4301c34388	fig|83333.1.peg.726	Cytochrome d ubiquinol oxidase subunit II (EC 1.10.3.-)
b9f03a52e78ffa0c5f3d4d4301c34388	fig|83334.1.peg.839	Cytochrome d ubiquinol oxidase subunit II (EC 1.10.3.-)
b9f03a52e78ffa0c5f3d4d4301c34388	fig|869729.3.peg.2978	Cytochrome d ubiquinol oxidase subunit II (EC 1.10.3.-)
20d083c4bac3ccf7cfb27d2ae66e613f	fig|4932.3.peg.1373	L-asparaginase (EC 3.5.1.1)
20d083c4bac3ccf7cfb27d2ae66e613f	fig|559292.3.peg.1206	L-asparaginase (EC 3.5.1.1)
7aefa8b335a998c5135c5aad7cc9855d	fig|198215.1.peg.1085	Spermidine Putrescine ABC transporter permease component PotB (TC 3.A.1.11.1)
7aefa8b335a998c5135c5aad7cc9855d	fig|216592.3.peg.1242	Spermidine Putrescine ABC transporter permease component PotB (TC 3.A.1.11.1)
7aefa8b335a998c5135c5aad7cc9855d	fig|316385.7.peg.1223	Spermidine Putrescine ABC transporter permease component PotB (TC 3.A.1.11.1)
7aefa8b335a998c5135c5aad7cc9855d	fig|316407.3.peg.1087	Spermidine Putrescine ABC transporter permease component PotB (TC 3.A.1.11.1)
7aefa8b335a998c5135c5aad7cc9855d	fig|331112.6.peg.1213	Spermidine Putrescine ABC transporter permease component PotB (TC 3.A.1.11.1)
7aefa8b335a998c5135c5aad7cc9855d	fig|340197.5.peg.3335	Spermidine Putrescine ABC transporter permease component PotB (TC 3.A.1.11.1)
7aefa8b335a998c5135c5aad7cc9855d	fig|362663.8.peg.1141	Spermidine Putrescine ABC transporter permease component PotB (TC 3.A.1.11.1)
7aefa8b335a998c5135c5aad7cc9855d	fig|362663.9.peg.1141	Spermidine Putrescine ABC transporter permease component PotB (TC 3.A.1.11.1)
7aefa8b335a998c5135c5aad7cc9855d	fig|364106.7.peg.1329	Spermidine Putrescine ABC transporter permease component PotB (TC 3.A.1.11.1)
7aefa8b335a998c5135c5aad7cc9855d	fig|364106.8.peg.1328	Spermidine Putrescine ABC transporter permease component PotB (TC 3.A.1.11.1)
7aefa8b335a998c5135c5aad7cc9855d	fig|405955.13.peg.1129	Spermidine Putrescine ABC transporter permease component PotB (TC 3.A.1.11.1)
7aefa8b335a998c5135c5aad7cc9855d	fig|413997.3.peg.1184	Spermidine Putrescine ABC transporter permease component PotB (TC 3.A.1.11.1)
7aefa8b335a998c5135c5aad7cc9855d	fig|431946.3.peg.1074	Spermidine Putrescine ABC transporter permease component PotB (TC 3.A.1.11.1)
7aefa8b335a998c5135c5aad7cc9855d	fig|457401.3.peg.1065	Spermidine Putrescine ABC transporter permease component PotB (TC 3.A.1.11.1)
7aefa8b335a998c5135c5aad7cc9855d	fig|469598.5.peg.813	Spermidine Putrescine ABC transporter permease component PotB (TC 3.A.1.11.1)
7aefa8b335a998c5135c5aad7cc9855d	fig|481805.6.peg.2649	Spermidine Putrescine ABC transporter permease component PotB (TC 3.A.1.11.1)
7aefa8b335a998c5135c5aad7cc9855d	fig|511693.5.peg.1213	Spermidine Putrescine ABC transporter permease component PotB (TC 3.A.1.11.1)
7aefa8b335a998c5135c5aad7cc9855d	fig|556266.3.peg.680	Spermidine Putrescine ABC transporter permease component PotB (TC 3.A.1.11.1)
7aefa8b335a998c5135c5aad7cc9855d	fig|562.375.peg.5076	Spermidine Putrescine ABC transporter permease component PotB (TC 3.A.1.11.1)
7aefa8b335a998c5135c5aad7cc9855d	fig|585055.6.peg.1270	Spermidine Putrescine ABC transporter permease component PotB (TC 3.A.1.11.1)
7aefa8b335a998c5135c5aad7cc9855d	fig|585055.8.peg.1269	Spermidine Putrescine ABC transporter permease component PotB (TC 3.A.1.11.1)
7aefa8b335a998c5135c5aad7cc9855d	fig|585057.4.peg.2116	Spermidine Putrescine ABC transporter permease component PotB (TC 3.A.1.11.1)
7aefa8b335a998c5135c5aad7cc9855d	fig|585057.6.peg.2115	Spermidine Putrescine ABC transporter permease component PotB (TC 3.A.1.11.1)
7aefa8b335a998c5135c5aad7cc9855d	fig|595496.3.peg.1071	Spermidine Putrescine ABC transporter permease component PotB (TC 3.A.1.11.1)
7aefa8b335a998c5135c5aad7cc9855d	fig|656414.3.peg.1371	Spermidine Putrescine ABC transporter permease component PotB (TC 3.A.1.11.1)
7aefa8b335a998c5135c5aad7cc9855d	fig|656437.3.peg.1271	Spermidine Putrescine ABC transporter permease component PotB (TC 3.A.1.11.1)
7aefa8b335a998c5135c5aad7cc9855d	fig|656444.3.peg.1774	Spermidine Putrescine ABC transporter permease component PotB (TC 3.A.1.11.1)
7aefa8b335a998c5135c5aad7cc9855d	fig|6666666.5365.peg.5913	Spermidine Putrescine ABC transporter permease component PotB (TC 3.A.1.11.1)
7aefa8b335a998c5135c5aad7cc9855d	fig|6666666.5522.peg.2449	Spermidine Putrescine ABC transporter permease component PotB (TC 3.A.1.11.1)
7aefa8b335a998c5135c5aad7cc9855d	fig|670888.3.peg.1752	Spermidine Putrescine ABC transporter permease component PotB (TC 3.A.1.11.1)
7aefa8b335a998c5135c5aad7cc9855d	fig|679206.4.peg.864	Spermidine Putrescine ABC transporter permease component PotB (TC 3.A.1.11.1)
7aefa8b335a998c5135c5aad7cc9855d	fig|679207.4.peg.1842	Spermidine Putrescine ABC transporter permease component PotB (TC 3.A.1.11.1)
7aefa8b335a998c5135c5aad7cc9855d	fig|701177.3.peg.1376	Spermidine Putrescine ABC transporter permease component PotB (TC 3.A.1.11.1)
7aefa8b335a998c5135c5aad7cc9855d	fig|714962.3.peg.1256	Spermidine Putrescine ABC transporter permease component PotB (TC 3.A.1.11.1)
7aefa8b335a998c5135c5aad7cc9855d	fig|749527.3.peg.3373	Spermidine Putrescine ABC transporter permease component PotB (TC 3.A.1.11.1)
7aefa8b335a998c5135c5aad7cc9855d	fig|749532.3.peg.3250	Spermidine Putrescine ABC transporter permease component PotB (TC 3.A.1.11.1)
7aefa8b335a998c5135c5aad7cc9855d	fig|749537.3.peg.1624	Spermidine Putrescine ABC transporter permease component PotB (TC 3.A.1.11.1)
7aefa8b335a998c5135c5aad7cc9855d	fig|749545.3.peg.845	Spermidine Putrescine ABC transporter permease component PotB (TC 3.A.1.11.1)
7aefa8b335a998c5135c5aad7cc9855d	fig|749547.3.peg.660	Spermidine Putrescine ABC transporter permease component PotB (TC 3.A.1.11.1)
7aefa8b335a998c5135c5aad7cc9855d	fig|749550.3.peg.2039	Spermidine Putrescine ABC transporter permease component PotB (TC 3.A.1.11.1)
7aefa8b335a998c5135c5aad7cc9855d	fig|753642.3.peg.2186	Spermidine Putrescine ABC transporter permease component PotB (TC 3.A.1.11.1)
7aefa8b335a998c5135c5aad7cc9855d	fig|83333.1.peg.1110	Spermidine Putrescine ABC transporter permease component PotB (TC 3.A.1.11.1)
7aefa8b335a998c5135c5aad7cc9855d	fig|869729.3.peg.2495	Spermidine Putrescine ABC transporter permease component PotB (TC 3.A.1.11.1)
90353d691d465d58cd65c42d31e405ae	fig|224308.43.peg.2450	Phosphopentomutase (EC 5.4.2.7)
90353d691d465d58cd65c42d31e405ae	fig|224308.49.peg.2361	Phosphopentomutase (EC 5.4.2.7)
90353d691d465d58cd65c42d31e405ae	fig|535024.3.peg.1481	Phosphopentomutase (EC 5.4.2.7)
90353d691d465d58cd65c42d31e405ae	fig|535025.4.peg.3568	Phosphopentomutase (EC 5.4.2.7)
90353d691d465d58cd65c42d31e405ae	fig|535026.3.peg.2507	Phosphopentomutase (EC 5.4.2.7)
9b6c79347099bb5f161d3ea00a812b4f	fig|3702.1.peg.15952	Asparagine synthetase [glutamine-hydrolyzing] (EC 6.3.5.4)
9b6c79347099bb5f161d3ea00a812b4f	fig|3702.7.peg.29385	Asparagine synthetase [glutamine-hydrolyzing] (EC 6.3.5.4)
224c9b5cddbe7cce94d4b0d9efb673f8	fig|316407.3.peg.3375	Ribose ABC transport system, high affinity permease RbsD (TC 3.A.1.2.1)
224c9b5cddbe7cce94d4b0d9efb673f8	fig|386585.9.peg.4896	Ribose ABC transport system, high affinity permease RbsD (TC 3.A.1.2.1)
224c9b5cddbe7cce94d4b0d9efb673f8	fig|444447.5.peg.2153	Ribose ABC transport system, high affinity permease RbsD (TC 3.A.1.2.1)
224c9b5cddbe7cce94d4b0d9efb673f8	fig|444448.5.peg.1989	Ribose ABC transport system, high affinity permease RbsD (TC 3.A.1.2.1)
224c9b5cddbe7cce94d4b0d9efb673f8	fig|444451.5.peg.5307	Ribose ABC transport system, high affinity permease RbsD (TC 3.A.1.2.1)
224c9b5cddbe7cce94d4b0d9efb673f8	fig|444453.5.peg.2130	Ribose ABC transport system, high affinity permease RbsD (TC 3.A.1.2.1)
224c9b5cddbe7cce94d4b0d9efb673f8	fig|478005.5.peg.2058	Ribose ABC transport system, high affinity permease RbsD (TC 3.A.1.2.1)
224c9b5cddbe7cce94d4b0d9efb673f8	fig|562.371.peg.2972	Ribose ABC transport system, high affinity permease RbsD (TC 3.A.1.2.1)
224c9b5cddbe7cce94d4b0d9efb673f8	fig|562.372.peg.3856	Ribose ABC transport system, high affinity permease RbsD (TC 3.A.1.2.1)
224c9b5cddbe7cce94d4b0d9efb673f8	fig|570506.3.peg.4803	Ribose ABC transport system, high affinity permease RbsD (TC 3.A.1.2.1)
224c9b5cddbe7cce94d4b0d9efb673f8	fig|701177.3.peg.4565	Ribose ABC transport system, high affinity permease RbsD (TC 3.A.1.2.1)
224c9b5cddbe7cce94d4b0d9efb673f8	fig|83334.1.peg.4661	Ribose ABC transport system, high affinity permease RbsD (TC 3.A.1.2.1)
1448673b9a3465ab1c7d40e7786b0648	fig|300268.10.peg.2355	Phosphoenolpyruvate-dihydroxyacetone phosphotransferase (EC 2.7.1.121), ADP-binding subunit DhaL
1448673b9a3465ab1c7d40e7786b0648	fig|300268.11.peg.2382	Phosphoenolpyruvate-dihydroxyacetone phosphotransferase (EC 2.7.1.121), ADP-binding subunit DhaL
1448673b9a3465ab1c7d40e7786b0648	fig|316385.7.peg.1279	Phosphoenolpyruvate-dihydroxyacetone phosphotransferase (EC 2.7.1.121), ADP-binding subunit DhaL
1448673b9a3465ab1c7d40e7786b0648	fig|316401.4.peg.1507	Phosphoenolpyruvate-dihydroxyacetone phosphotransferase (EC 2.7.1.121), ADP-binding subunit DhaL
1448673b9a3465ab1c7d40e7786b0648	fig|316407.3.peg.1160	Phosphoenolpyruvate-dihydroxyacetone phosphotransferase (EC 2.7.1.121), ADP-binding subunit DhaL
1448673b9a3465ab1c7d40e7786b0648	fig|344609.11.peg.1494	Phosphoenolpyruvate-dihydroxyacetone phosphotransferase (EC 2.7.1.121), ADP-binding subunit DhaL
1448673b9a3465ab1c7d40e7786b0648	fig|344609.3.peg.1093	Phosphoenolpyruvate-dihydroxyacetone phosphotransferase (EC 2.7.1.121), ADP-binding subunit DhaL
1448673b9a3465ab1c7d40e7786b0648	fig|358708.5.peg.4843	Phosphoenolpyruvate-dihydroxyacetone phosphotransferase (EC 2.7.1.121), ADP-binding subunit DhaL
1448673b9a3465ab1c7d40e7786b0648	fig|358709.5.peg.1527	Phosphoenolpyruvate-dihydroxyacetone phosphotransferase (EC 2.7.1.121), ADP-binding subunit DhaL
1448673b9a3465ab1c7d40e7786b0648	fig|413997.3.peg.1238	Phosphoenolpyruvate-dihydroxyacetone phosphotransferase (EC 2.7.1.121), ADP-binding subunit DhaL
1448673b9a3465ab1c7d40e7786b0648	fig|457401.3.peg.1010	Phosphoenolpyruvate-dihydroxyacetone phosphotransferase (EC 2.7.1.121), ADP-binding subunit DhaL
1448673b9a3465ab1c7d40e7786b0648	fig|469008.4.peg.2497	Phosphoenolpyruvate-dihydroxyacetone phosphotransferase (EC 2.7.1.121), ADP-binding subunit DhaL
1448673b9a3465ab1c7d40e7786b0648	fig|481805.3.peg.2604	Phosphoenolpyruvate-dihydroxyacetone phosphotransferase (EC 2.7.1.121), ADP-binding subunit DhaL
1448673b9a3465ab1c7d40e7786b0648	fig|481805.6.peg.2591	Phosphoenolpyruvate-dihydroxyacetone phosphotransferase (EC 2.7.1.121), ADP-binding subunit DhaL
1448673b9a3465ab1c7d40e7786b0648	fig|511145.12.peg.1246	Phosphoenolpyruvate-dihydroxyacetone phosphotransferase (EC 2.7.1.121), ADP-binding subunit DhaL
1448673b9a3465ab1c7d40e7786b0648	fig|511145.6.peg.1235	Phosphoenolpyruvate-dihydroxyacetone phosphotransferase (EC 2.7.1.121), ADP-binding subunit DhaL
1448673b9a3465ab1c7d40e7786b0648	fig|511693.5.peg.1269	Phosphoenolpyruvate-dihydroxyacetone phosphotransferase (EC 2.7.1.121), ADP-binding subunit DhaL
1448673b9a3465ab1c7d40e7786b0648	fig|536056.3.peg.2580	Phosphoenolpyruvate-dihydroxyacetone phosphotransferase (EC 2.7.1.121), ADP-binding subunit DhaL
1448673b9a3465ab1c7d40e7786b0648	fig|595496.3.peg.1127	Phosphoenolpyruvate-dihydroxyacetone phosphotransferase (EC 2.7.1.121), ADP-binding subunit DhaL
1448673b9a3465ab1c7d40e7786b0648	fig|621.8.peg.4604	Phosphoenolpyruvate-dihydroxyacetone phosphotransferase (EC 2.7.1.121), ADP-binding subunit DhaL
1448673b9a3465ab1c7d40e7786b0648	fig|622.8.peg.3642	Phosphoenolpyruvate-dihydroxyacetone phosphotransferase (EC 2.7.1.121), ADP-binding subunit DhaL
1448673b9a3465ab1c7d40e7786b0648	fig|623.7.peg.301	Phosphoenolpyruvate-dihydroxyacetone phosphotransferase (EC 2.7.1.121), ADP-binding subunit DhaL
1448673b9a3465ab1c7d40e7786b0648	fig|637912.3.peg.2498	Phosphoenolpyruvate-dihydroxyacetone phosphotransferase (EC 2.7.1.121), ADP-binding subunit DhaL
1448673b9a3465ab1c7d40e7786b0648	fig|656414.3.peg.1430	Phosphoenolpyruvate-dihydroxyacetone phosphotransferase (EC 2.7.1.121), ADP-binding subunit DhaL
1448673b9a3465ab1c7d40e7786b0648	fig|670888.3.peg.1879	Phosphoenolpyruvate-dihydroxyacetone phosphotransferase (EC 2.7.1.121), ADP-binding subunit DhaL
1448673b9a3465ab1c7d40e7786b0648	fig|749537.3.peg.3849	Phosphoenolpyruvate-dihydroxyacetone phosphotransferase (EC 2.7.1.121), ADP-binding subunit DhaL
1448673b9a3465ab1c7d40e7786b0648	fig|749538.3.peg.4715	Phosphoenolpyruvate-dihydroxyacetone phosphotransferase (EC 2.7.1.121), ADP-binding subunit DhaL
1448673b9a3465ab1c7d40e7786b0648	fig|749540.3.peg.4439	Phosphoenolpyruvate-dihydroxyacetone phosphotransferase (EC 2.7.1.121), ADP-binding subunit DhaL
1448673b9a3465ab1c7d40e7786b0648	fig|749544.3.peg.1398	Phosphoenolpyruvate-dihydroxyacetone phosphotransferase (EC 2.7.1.121), ADP-binding subunit DhaL
1448673b9a3465ab1c7d40e7786b0648	fig|749547.3.peg.599	Phosphoenolpyruvate-dihydroxyacetone phosphotransferase (EC 2.7.1.121), ADP-binding subunit DhaL
1448673b9a3465ab1c7d40e7786b0648	fig|749548.3.peg.4964	Phosphoenolpyruvate-dihydroxyacetone phosphotransferase (EC 2.7.1.121), ADP-binding subunit DhaL
1448673b9a3465ab1c7d40e7786b0648	fig|83333.1.peg.1185	Phosphoenolpyruvate-dihydroxyacetone phosphotransferase (EC 2.7.1.121), ADP-binding subunit DhaL
35557116fa2ab8e024b5c1e11c8a237e	fig|4932.3.peg.1680	Subunit Vb of cytochrome c oxidase, which is the terminal member of the mitochondrial inner membrane electron transport chain; predominantly expressed during anaerobic growth while its isoform Va (Cox5Ap) is expressed during aerobic growth
35557116fa2ab8e024b5c1e11c8a237e	fig|559292.3.peg.2687	Subunit Vb of cytochrome c oxidase, which is the terminal member of the mitochondrial inner membrane electron transport chain; predominantly expressed during anaerobic growth while its isoform Va (Cox5Ap) is expressed during aerobic growth
26b325269d71f8e676d573888d32811c	fig|233413.1.peg.2441	NAD synthetase (EC 6.3.1.5) / Glutamine amidotransferase chain of NAD synthetase
1f9a8356d1f4271503b6a7b636a7aec2	fig|216592.1.peg.3729	Transporter, LysE family
1f9a8356d1f4271503b6a7b636a7aec2	fig|316407.3.peg.2817	Transporter, LysE family
1f9a8356d1f4271503b6a7b636a7aec2	fig|331112.3.peg.2877	Transporter, LysE family
1f9a8356d1f4271503b6a7b636a7aec2	fig|481805.3.peg.838	Transporter, LysE family
1f9a8356d1f4271503b6a7b636a7aec2	fig|83333.1.peg.2874	Transporter, LysE family
f1c9fe6d8c99edae65b1aafd2931c961	fig|379731.4.peg.4152	Cytochrome c oxidase polypeptide I (EC 1.9.3.1)
f1c9fe6d8c99edae65b1aafd2931c961	fig|379731.5.peg.4153	Cytochrome c oxidase polypeptide I (EC 1.9.3.1)
36871dcfe1571efa43dd6a24b6f8caf0	fig|155864.1.peg.4131	Biotin carboxyl carrier protein of acetyl-CoA carboxylase
36871dcfe1571efa43dd6a24b6f8caf0	fig|155864.8.peg.4066	Biotin carboxyl carrier protein of acetyl-CoA carboxylase
36871dcfe1571efa43dd6a24b6f8caf0	fig|198214.1.peg.3090	Biotin carboxyl carrier protein of acetyl-CoA carboxylase
36871dcfe1571efa43dd6a24b6f8caf0	fig|198214.7.peg.3901	Biotin carboxyl carrier protein of acetyl-CoA carboxylase
36871dcfe1571efa43dd6a24b6f8caf0	fig|198215.1.peg.3015	Biotin carboxyl carrier protein of acetyl-CoA carboxylase
36871dcfe1571efa43dd6a24b6f8caf0	fig|198215.6.peg.3840	Biotin carboxyl carrier protein of acetyl-CoA carboxylase
36871dcfe1571efa43dd6a24b6f8caf0	fig|199310.1.peg.3920	Biotin carboxyl carrier protein of acetyl-CoA carboxylase
36871dcfe1571efa43dd6a24b6f8caf0	fig|199310.4.peg.3776	Biotin carboxyl carrier protein of acetyl-CoA carboxylase
36871dcfe1571efa43dd6a24b6f8caf0	fig|216592.1.peg.4184	Biotin carboxyl carrier protein of acetyl-CoA carboxylase
36871dcfe1571efa43dd6a24b6f8caf0	fig|216592.3.peg.3678	Biotin carboxyl carrier protein of acetyl-CoA carboxylase
36871dcfe1571efa43dd6a24b6f8caf0	fig|216593.1.peg.4888	Biotin carboxyl carrier protein of acetyl-CoA carboxylase
36871dcfe1571efa43dd6a24b6f8caf0	fig|216599.1.peg.4275	Biotin carboxyl carrier protein of acetyl-CoA carboxylase
36871dcfe1571efa43dd6a24b6f8caf0	fig|300268.10.peg.3779	Biotin carboxyl carrier protein of acetyl-CoA carboxylase
36871dcfe1571efa43dd6a24b6f8caf0	fig|300268.11.peg.3817	Biotin carboxyl carrier protein of acetyl-CoA carboxylase
36871dcfe1571efa43dd6a24b6f8caf0	fig|300269.11.peg.3903	Biotin carboxyl carrier protein of acetyl-CoA carboxylase
36871dcfe1571efa43dd6a24b6f8caf0	fig|300269.12.peg.4029	Biotin carboxyl carrier protein of acetyl-CoA carboxylase
36871dcfe1571efa43dd6a24b6f8caf0	fig|316385.5.peg.3383	Biotin carboxyl carrier protein of acetyl-CoA carboxylase
36871dcfe1571efa43dd6a24b6f8caf0	fig|316385.7.peg.3457	Biotin carboxyl carrier protein of acetyl-CoA carboxylase
36871dcfe1571efa43dd6a24b6f8caf0	fig|316401.4.peg.3989	Biotin carboxyl carrier protein of acetyl-CoA carboxylase
36871dcfe1571efa43dd6a24b6f8caf0	fig|316407.3.peg.3131	Biotin carboxyl carrier protein of acetyl-CoA carboxylase
36871dcfe1571efa43dd6a24b6f8caf0	fig|331111.12.peg.3993	Biotin carboxyl carrier protein of acetyl-CoA carboxylase
36871dcfe1571efa43dd6a24b6f8caf0	fig|331111.3.peg.1406	Biotin carboxyl carrier protein of acetyl-CoA carboxylase
36871dcfe1571efa43dd6a24b6f8caf0	fig|331112.3.peg.3226	Biotin carboxyl carrier protein of acetyl-CoA carboxylase
36871dcfe1571efa43dd6a24b6f8caf0	fig|331112.6.peg.3363	Biotin carboxyl carrier protein of acetyl-CoA carboxylase
36871dcfe1571efa43dd6a24b6f8caf0	fig|340185.3.peg.5196	Biotin carboxyl carrier protein of acetyl-CoA carboxylase
36871dcfe1571efa43dd6a24b6f8caf0	fig|340185.4.peg.5511	Biotin carboxyl carrier protein of acetyl-CoA carboxylase
36871dcfe1571efa43dd6a24b6f8caf0	fig|340186.3.peg.5096	Biotin carboxyl carrier protein of acetyl-CoA carboxylase
36871dcfe1571efa43dd6a24b6f8caf0	fig|340186.5.peg.5394	Biotin carboxyl carrier protein of acetyl-CoA carboxylase
36871dcfe1571efa43dd6a24b6f8caf0	fig|340197.3.peg.3961	Biotin carboxyl carrier protein of acetyl-CoA carboxylase
36871dcfe1571efa43dd6a24b6f8caf0	fig|340197.5.peg.4137	Biotin carboxyl carrier protein of acetyl-CoA carboxylase
36871dcfe1571efa43dd6a24b6f8caf0	fig|344609.11.peg.3620	Biotin carboxyl carrier protein of acetyl-CoA carboxylase
36871dcfe1571efa43dd6a24b6f8caf0	fig|344609.3.peg.1266	Biotin carboxyl carrier protein of acetyl-CoA carboxylase
36871dcfe1571efa43dd6a24b6f8caf0	fig|344610.3.peg.4770	Biotin carboxyl carrier protein of acetyl-CoA carboxylase
36871dcfe1571efa43dd6a24b6f8caf0	fig|344610.7.peg.2981	Biotin carboxyl carrier protein of acetyl-CoA carboxylase
36871dcfe1571efa43dd6a24b6f8caf0	fig|358709.5.peg.4984	Biotin carboxyl carrier protein of acetyl-CoA carboxylase
36871dcfe1571efa43dd6a24b6f8caf0	fig|362663.8.peg.3369	Biotin carboxyl carrier protein of acetyl-CoA carboxylase
36871dcfe1571efa43dd6a24b6f8caf0	fig|362663.9.peg.3380	Biotin carboxyl carrier protein of acetyl-CoA carboxylase
36871dcfe1571efa43dd6a24b6f8caf0	fig|364106.7.peg.3639	Biotin carboxyl carrier protein of acetyl-CoA carboxylase
36871dcfe1571efa43dd6a24b6f8caf0	fig|364106.8.peg.3640	Biotin carboxyl carrier protein of acetyl-CoA carboxylase
36871dcfe1571efa43dd6a24b6f8caf0	fig|373384.10.peg.3711	Biotin carboxyl carrier protein of acetyl-CoA carboxylase
36871dcfe1571efa43dd6a24b6f8caf0	fig|373384.11.peg.3751	Biotin carboxyl carrier protein of acetyl-CoA carboxylase
36871dcfe1571efa43dd6a24b6f8caf0	fig|386585.9.peg.4310	Biotin carboxyl carrier protein of acetyl-CoA carboxylase
36871dcfe1571efa43dd6a24b6f8caf0	fig|405955.13.peg.3666	Biotin carboxyl carrier protein of acetyl-CoA carboxylase
36871dcfe1571efa43dd6a24b6f8caf0	fig|405955.9.peg.3060	Biotin carboxyl carrier protein of acetyl-CoA carboxylase
36871dcfe1571efa43dd6a24b6f8caf0	fig|409438.11.peg.3704	Biotin carboxyl carrier protein of acetyl-CoA carboxylase
36871dcfe1571efa43dd6a24b6f8caf0	fig|413997.3.peg.3262	Biotin carboxyl carrier protein of acetyl-CoA carboxylase
36871dcfe1571efa43dd6a24b6f8caf0	fig|431946.3.peg.3214	Biotin carboxyl carrier protein of acetyl-CoA carboxylase
36871dcfe1571efa43dd6a24b6f8caf0	fig|439855.10.peg.3706	Biotin carboxyl carrier protein of acetyl-CoA carboxylase
36871dcfe1571efa43dd6a24b6f8caf0	fig|444447.5.peg.1558	Biotin carboxyl carrier protein of acetyl-CoA carboxylase
36871dcfe1571efa43dd6a24b6f8caf0	fig|444448.5.peg.1398	Biotin carboxyl carrier protein of acetyl-CoA carboxylase
36871dcfe1571efa43dd6a24b6f8caf0	fig|444449.5.peg.2645	Biotin carboxyl carrier protein of acetyl-CoA carboxylase
36871dcfe1571efa43dd6a24b6f8caf0	fig|444450.8.peg.4481	Biotin carboxyl carrier protein of acetyl-CoA carboxylase
36871dcfe1571efa43dd6a24b6f8caf0	fig|444451.5.peg.5535	Biotin carboxyl carrier protein of acetyl-CoA carboxylase
36871dcfe1571efa43dd6a24b6f8caf0	fig|444452.5.peg.5390	Biotin carboxyl carrier protein of acetyl-CoA carboxylase
36871dcfe1571efa43dd6a24b6f8caf0	fig|457400.3.peg.1194	Biotin carboxyl carrier protein of acetyl-CoA carboxylase
36871dcfe1571efa43dd6a24b6f8caf0	fig|457401.3.peg.2533	Biotin carboxyl carrier protein of acetyl-CoA carboxylase
36871dcfe1571efa43dd6a24b6f8caf0	fig|469008.4.peg.503	Biotin carboxyl carrier protein of acetyl-CoA carboxylase
36871dcfe1571efa43dd6a24b6f8caf0	fig|469598.5.peg.969	Biotin carboxyl carrier protein of acetyl-CoA carboxylase
36871dcfe1571efa43dd6a24b6f8caf0	fig|478004.5.peg.5457	Biotin carboxyl carrier protein of acetyl-CoA carboxylase
36871dcfe1571efa43dd6a24b6f8caf0	fig|478005.5.peg.3081	Biotin carboxyl carrier protein of acetyl-CoA carboxylase
36871dcfe1571efa43dd6a24b6f8caf0	fig|478008.5.peg.1455	Biotin carboxyl carrier protein of acetyl-CoA carboxylase
36871dcfe1571efa43dd6a24b6f8caf0	fig|481805.3.peg.477	Biotin carboxyl carrier protein of acetyl-CoA carboxylase
36871dcfe1571efa43dd6a24b6f8caf0	fig|481805.6.peg.479	Biotin carboxyl carrier protein of acetyl-CoA carboxylase
36871dcfe1571efa43dd6a24b6f8caf0	fig|502346.5.peg.3119	Biotin carboxyl carrier protein of acetyl-CoA carboxylase
36871dcfe1571efa43dd6a24b6f8caf0	fig|511145.12.peg.3354	Biotin carboxyl carrier protein of acetyl-CoA carboxylase
36871dcfe1571efa43dd6a24b6f8caf0	fig|511145.6.peg.3338	Biotin carboxyl carrier protein of acetyl-CoA carboxylase
36871dcfe1571efa43dd6a24b6f8caf0	fig|511693.5.peg.3274	Biotin carboxyl carrier protein of acetyl-CoA carboxylase
36871dcfe1571efa43dd6a24b6f8caf0	fig|525281.3.peg.3771	Biotin carboxyl carrier protein of acetyl-CoA carboxylase
36871dcfe1571efa43dd6a24b6f8caf0	fig|536056.3.peg.471	Biotin carboxyl carrier protein of acetyl-CoA carboxylase
36871dcfe1571efa43dd6a24b6f8caf0	fig|544404.4.peg.4291	Biotin carboxyl carrier protein of acetyl-CoA carboxylase
36871dcfe1571efa43dd6a24b6f8caf0	fig|550672.3.peg.2843	Biotin carboxyl carrier protein of acetyl-CoA carboxylase
36871dcfe1571efa43dd6a24b6f8caf0	fig|550676.3.peg.3405	Biotin carboxyl carrier protein of acetyl-CoA carboxylase
36871dcfe1571efa43dd6a24b6f8caf0	fig|550677.3.peg.3810	Biotin carboxyl carrier protein of acetyl-CoA carboxylase
36871dcfe1571efa43dd6a24b6f8caf0	fig|556266.3.peg.1607	Biotin carboxyl carrier protein of acetyl-CoA carboxylase
36871dcfe1571efa43dd6a24b6f8caf0	fig|562.371.peg.2565	Biotin carboxyl carrier protein of acetyl-CoA carboxylase
36871dcfe1571efa43dd6a24b6f8caf0	fig|562.372.peg.2760	Biotin carboxyl carrier protein of acetyl-CoA carboxylase
36871dcfe1571efa43dd6a24b6f8caf0	fig|562.373.peg.2038	Biotin carboxyl carrier protein of acetyl-CoA carboxylase
36871dcfe1571efa43dd6a24b6f8caf0	fig|562.374.peg.4837	Biotin carboxyl carrier protein of acetyl-CoA carboxylase
36871dcfe1571efa43dd6a24b6f8caf0	fig|562.375.peg.911	Biotin carboxyl carrier protein of acetyl-CoA carboxylase
36871dcfe1571efa43dd6a24b6f8caf0	fig|562.376.peg.1638	Biotin carboxyl carrier protein of acetyl-CoA carboxylase
36871dcfe1571efa43dd6a24b6f8caf0	fig|566546.3.peg.4720	Biotin carboxyl carrier protein of acetyl-CoA carboxylase
36871dcfe1571efa43dd6a24b6f8caf0	fig|566546.4.peg.3484	Biotin carboxyl carrier protein of acetyl-CoA carboxylase
36871dcfe1571efa43dd6a24b6f8caf0	fig|570506.3.peg.949	Biotin carboxyl carrier protein of acetyl-CoA carboxylase
36871dcfe1571efa43dd6a24b6f8caf0	fig|574521.7.peg.3604	Biotin carboxyl carrier protein of acetyl-CoA carboxylase
36871dcfe1571efa43dd6a24b6f8caf0	fig|585034.4.peg.3336	Biotin carboxyl carrier protein of acetyl-CoA carboxylase
36871dcfe1571efa43dd6a24b6f8caf0	fig|585034.5.peg.3334	Biotin carboxyl carrier protein of acetyl-CoA carboxylase
36871dcfe1571efa43dd6a24b6f8caf0	fig|585035.6.peg.3566	Biotin carboxyl carrier protein of acetyl-CoA carboxylase
36871dcfe1571efa43dd6a24b6f8caf0	fig|585054.5.peg.3129	Biotin carboxyl carrier protein of acetyl-CoA carboxylase
36871dcfe1571efa43dd6a24b6f8caf0	fig|585055.6.peg.3707	Biotin carboxyl carrier protein of acetyl-CoA carboxylase
36871dcfe1571efa43dd6a24b6f8caf0	fig|585055.8.peg.3710	Biotin carboxyl carrier protein of acetyl-CoA carboxylase
36871dcfe1571efa43dd6a24b6f8caf0	fig|585056.7.peg.3911	Biotin carboxyl carrier protein of acetyl-CoA carboxylase
36871dcfe1571efa43dd6a24b6f8caf0	fig|585057.4.peg.3874	Biotin carboxyl carrier protein of acetyl-CoA carboxylase
36871dcfe1571efa43dd6a24b6f8caf0	fig|585057.6.peg.3883	Biotin carboxyl carrier protein of acetyl-CoA carboxylase
36871dcfe1571efa43dd6a24b6f8caf0	fig|585395.4.peg.4178	Biotin carboxyl carrier protein of acetyl-CoA carboxylase
36871dcfe1571efa43dd6a24b6f8caf0	fig|585397.7.peg.3909	Biotin carboxyl carrier protein of acetyl-CoA carboxylase
36871dcfe1571efa43dd6a24b6f8caf0	fig|585397.9.peg.3906	Biotin carboxyl carrier protein of acetyl-CoA carboxylase
36871dcfe1571efa43dd6a24b6f8caf0	fig|591020.3.peg.3883	Biotin carboxyl carrier protein of acetyl-CoA carboxylase
36871dcfe1571efa43dd6a24b6f8caf0	fig|595495.4.peg.662	Biotin carboxyl carrier protein of acetyl-CoA carboxylase
36871dcfe1571efa43dd6a24b6f8caf0	fig|595496.3.peg.3237	Biotin carboxyl carrier protein of acetyl-CoA carboxylase
36871dcfe1571efa43dd6a24b6f8caf0	fig|621.8.peg.63	Biotin carboxyl carrier protein of acetyl-CoA carboxylase
36871dcfe1571efa43dd6a24b6f8caf0	fig|622.8.peg.5123	Biotin carboxyl carrier protein of acetyl-CoA carboxylase
36871dcfe1571efa43dd6a24b6f8caf0	fig|623.7.peg.1656	Biotin carboxyl carrier protein of acetyl-CoA carboxylase
36871dcfe1571efa43dd6a24b6f8caf0	fig|637388.3.peg.5344	Biotin carboxyl carrier protein of acetyl-CoA carboxylase
36871dcfe1571efa43dd6a24b6f8caf0	fig|637912.3.peg.2880	Biotin carboxyl carrier protein of acetyl-CoA carboxylase
36871dcfe1571efa43dd6a24b6f8caf0	fig|655817.3.peg.3827	Biotin carboxyl carrier protein of acetyl-CoA carboxylase
36871dcfe1571efa43dd6a24b6f8caf0	fig|656379.3.peg.3994	Biotin carboxyl carrier protein of acetyl-CoA carboxylase
36871dcfe1571efa43dd6a24b6f8caf0	fig|656380.3.peg.3913	Biotin carboxyl carrier protein of acetyl-CoA carboxylase
36871dcfe1571efa43dd6a24b6f8caf0	fig|656393.3.peg.4302	Biotin carboxyl carrier protein of acetyl-CoA carboxylase
36871dcfe1571efa43dd6a24b6f8caf0	fig|656408.3.peg.3678	Biotin carboxyl carrier protein of acetyl-CoA carboxylase
36871dcfe1571efa43dd6a24b6f8caf0	fig|656414.3.peg.3735	Biotin carboxyl carrier protein of acetyl-CoA carboxylase
36871dcfe1571efa43dd6a24b6f8caf0	fig|656417.3.peg.4086	Biotin carboxyl carrier protein of acetyl-CoA carboxylase
36871dcfe1571efa43dd6a24b6f8caf0	fig|656419.3.peg.4236	Biotin carboxyl carrier protein of acetyl-CoA carboxylase
36871dcfe1571efa43dd6a24b6f8caf0	fig|656437.3.peg.3679	Biotin carboxyl carrier protein of acetyl-CoA carboxylase
36871dcfe1571efa43dd6a24b6f8caf0	fig|656440.3.peg.3484	Biotin carboxyl carrier protein of acetyl-CoA carboxylase
36871dcfe1571efa43dd6a24b6f8caf0	fig|656443.3.peg.4153	Biotin carboxyl carrier protein of acetyl-CoA carboxylase
36871dcfe1571efa43dd6a24b6f8caf0	fig|656444.3.peg.4517	Biotin carboxyl carrier protein of acetyl-CoA carboxylase
36871dcfe1571efa43dd6a24b6f8caf0	fig|6666666.5365.peg.1589	Biotin carboxyl carrier protein of acetyl-CoA carboxylase
36871dcfe1571efa43dd6a24b6f8caf0	fig|6666666.5522.peg.1853	Biotin carboxyl carrier protein of acetyl-CoA carboxylase
36871dcfe1571efa43dd6a24b6f8caf0	fig|670888.3.peg.2993	Biotin carboxyl carrier protein of acetyl-CoA carboxylase
36871dcfe1571efa43dd6a24b6f8caf0	fig|670897.3.peg.4079	Biotin carboxyl carrier protein of acetyl-CoA carboxylase
36871dcfe1571efa43dd6a24b6f8caf0	fig|679204.3.peg.989	Biotin carboxyl carrier protein of acetyl-CoA carboxylase
36871dcfe1571efa43dd6a24b6f8caf0	fig|679205.4.peg.2770	Biotin carboxyl carrier protein of acetyl-CoA carboxylase
36871dcfe1571efa43dd6a24b6f8caf0	fig|679206.4.peg.4687	Biotin carboxyl carrier protein of acetyl-CoA carboxylase
36871dcfe1571efa43dd6a24b6f8caf0	fig|679207.4.peg.2482	Biotin carboxyl carrier protein of acetyl-CoA carboxylase
36871dcfe1571efa43dd6a24b6f8caf0	fig|685038.3.peg.3287	Biotin carboxyl carrier protein of acetyl-CoA carboxylase
36871dcfe1571efa43dd6a24b6f8caf0	fig|714962.3.peg.3704	Biotin carboxyl carrier protein of acetyl-CoA carboxylase
36871dcfe1571efa43dd6a24b6f8caf0	fig|749527.3.peg.2171	Biotin carboxyl carrier protein of acetyl-CoA carboxylase
36871dcfe1571efa43dd6a24b6f8caf0	fig|749528.3.peg.4210	Biotin carboxyl carrier protein of acetyl-CoA carboxylase
36871dcfe1571efa43dd6a24b6f8caf0	fig|749531.3.peg.2457	Biotin carboxyl carrier protein of acetyl-CoA carboxylase
36871dcfe1571efa43dd6a24b6f8caf0	fig|749532.3.peg.1238	Biotin carboxyl carrier protein of acetyl-CoA carboxylase
36871dcfe1571efa43dd6a24b6f8caf0	fig|749533.3.peg.2395	Biotin carboxyl carrier protein of acetyl-CoA carboxylase
36871dcfe1571efa43dd6a24b6f8caf0	fig|749537.3.peg.1192	Biotin carboxyl carrier protein of acetyl-CoA carboxylase
36871dcfe1571efa43dd6a24b6f8caf0	fig|749538.3.peg.1767	Biotin carboxyl carrier protein of acetyl-CoA carboxylase
36871dcfe1571efa43dd6a24b6f8caf0	fig|749540.3.peg.3414	Biotin carboxyl carrier protein of acetyl-CoA carboxylase
36871dcfe1571efa43dd6a24b6f8caf0	fig|749545.3.peg.3825	Biotin carboxyl carrier protein of acetyl-CoA carboxylase
36871dcfe1571efa43dd6a24b6f8caf0	fig|749546.3.peg.1837	Biotin carboxyl carrier protein of acetyl-CoA carboxylase
36871dcfe1571efa43dd6a24b6f8caf0	fig|749547.3.peg.1032	Biotin carboxyl carrier protein of acetyl-CoA carboxylase
36871dcfe1571efa43dd6a24b6f8caf0	fig|749548.3.peg.3961	Biotin carboxyl carrier protein of acetyl-CoA carboxylase
36871dcfe1571efa43dd6a24b6f8caf0	fig|749549.3.peg.2006	Biotin carboxyl carrier protein of acetyl-CoA carboxylase
36871dcfe1571efa43dd6a24b6f8caf0	fig|749550.3.peg.1319	Biotin carboxyl carrier protein of acetyl-CoA carboxylase
36871dcfe1571efa43dd6a24b6f8caf0	fig|753642.3.peg.4715	Biotin carboxyl carrier protein of acetyl-CoA carboxylase
36871dcfe1571efa43dd6a24b6f8caf0	fig|83333.1.peg.3197	Biotin carboxyl carrier protein of acetyl-CoA carboxylase
36871dcfe1571efa43dd6a24b6f8caf0	fig|83334.1.peg.4100	Biotin carboxyl carrier protein of acetyl-CoA carboxylase
36871dcfe1571efa43dd6a24b6f8caf0	fig|869729.3.peg.28	Biotin carboxyl carrier protein of acetyl-CoA carboxylase
a238cd9d1174930eb3db4bce4c988d0a	fig|262724.1.peg.560	Phosphopantetheine adenylyltransferase (EC 2.7.7.3)
a238cd9d1174930eb3db4bce4c988d0a	fig|262724.6.peg.559	Phosphopantetheine adenylyltransferase (EC 2.7.7.3)
a238cd9d1174930eb3db4bce4c988d0a	fig|300852.3.peg.1013	Phosphopantetheine adenylyltransferase (EC 2.7.7.3)
a238cd9d1174930eb3db4bce4c988d0a	fig|300852.9.peg.912	Phosphopantetheine adenylyltransferase (EC 2.7.7.3)
c666367d127b4ef9fb8355b60616afaa	fig|224308.1.peg.2667	Cobalt-zinc-cadmium resistance protein CzcD
c666367d127b4ef9fb8355b60616afaa	fig|224308.43.peg.2776	Cobalt-zinc-cadmium resistance protein CzcD
c666367d127b4ef9fb8355b60616afaa	fig|224308.49.peg.2675	Cobalt-zinc-cadmium resistance protein CzcD
c666367d127b4ef9fb8355b60616afaa	fig|535024.3.peg.1809	Cobalt-zinc-cadmium resistance protein CzcD
c666367d127b4ef9fb8355b60616afaa	fig|535025.4.peg.3897	Cobalt-zinc-cadmium resistance protein CzcD
c666367d127b4ef9fb8355b60616afaa	fig|535026.3.peg.2838	Cobalt-zinc-cadmium resistance protein CzcD
62951e464659f15414bb3cbb7df27595	fig|99287.1.peg.115	Acetolactate synthase small subunit (EC 2.2.1.6)
32f3803c6c8bcec272568f71495b48e8	fig|216597.6.peg.2580	Histidine ABC transporter, ATP-binding protein HisP (TC 3.A.1.3.1)
32f3803c6c8bcec272568f71495b48e8	fig|272994.5.peg.526	Histidine ABC transporter, ATP-binding protein HisP (TC 3.A.1.3.1)
32f3803c6c8bcec272568f71495b48e8	fig|272994.6.peg.515	Histidine ABC transporter, ATP-binding protein HisP (TC 3.A.1.3.1)
32f3803c6c8bcec272568f71495b48e8	fig|28901.42.peg.2343	Histidine ABC transporter, ATP-binding protein HisP (TC 3.A.1.3.1)
32f3803c6c8bcec272568f71495b48e8	fig|439842.10.peg.2160	Histidine ABC transporter, ATP-binding protein HisP (TC 3.A.1.3.1)
32f3803c6c8bcec272568f71495b48e8	fig|439842.7.peg.2168	Histidine ABC transporter, ATP-binding protein HisP (TC 3.A.1.3.1)
32f3803c6c8bcec272568f71495b48e8	fig|439843.6.peg.2625	Histidine ABC transporter, ATP-binding protein HisP (TC 3.A.1.3.1)
32f3803c6c8bcec272568f71495b48e8	fig|439843.8.peg.2637	Histidine ABC transporter, ATP-binding protein HisP (TC 3.A.1.3.1)
32f3803c6c8bcec272568f71495b48e8	fig|439846.4.peg.2897	Histidine ABC transporter, ATP-binding protein HisP (TC 3.A.1.3.1)
32f3803c6c8bcec272568f71495b48e8	fig|439851.5.peg.2722	Histidine ABC transporter, ATP-binding protein HisP (TC 3.A.1.3.1)
32f3803c6c8bcec272568f71495b48e8	fig|439851.8.peg.2698	Histidine ABC transporter, ATP-binding protein HisP (TC 3.A.1.3.1)
32f3803c6c8bcec272568f71495b48e8	fig|440534.5.peg.2869	Histidine ABC transporter, ATP-binding protein HisP (TC 3.A.1.3.1)
32f3803c6c8bcec272568f71495b48e8	fig|454166.6.peg.2439	Histidine ABC transporter, ATP-binding protein HisP (TC 3.A.1.3.1)
32f3803c6c8bcec272568f71495b48e8	fig|454166.8.peg.2439	Histidine ABC transporter, ATP-binding protein HisP (TC 3.A.1.3.1)
32f3803c6c8bcec272568f71495b48e8	fig|454167.5.peg.2681	Histidine ABC transporter, ATP-binding protein HisP (TC 3.A.1.3.1)
32f3803c6c8bcec272568f71495b48e8	fig|454168.5.peg.3024	Histidine ABC transporter, ATP-binding protein HisP (TC 3.A.1.3.1)
32f3803c6c8bcec272568f71495b48e8	fig|454169.8.peg.2503	Histidine ABC transporter, ATP-binding protein HisP (TC 3.A.1.3.1)
32f3803c6c8bcec272568f71495b48e8	fig|454231.5.peg.366	Histidine ABC transporter, ATP-binding protein HisP (TC 3.A.1.3.1)
32f3803c6c8bcec272568f71495b48e8	fig|465516.5.peg.1937	Histidine ABC transporter, ATP-binding protein HisP (TC 3.A.1.3.1)
32f3803c6c8bcec272568f71495b48e8	fig|465517.10.peg.2583	Histidine ABC transporter, ATP-binding protein HisP (TC 3.A.1.3.1)
32f3803c6c8bcec272568f71495b48e8	fig|465518.5.peg.162	Histidine ABC transporter, ATP-binding protein HisP (TC 3.A.1.3.1)
32f3803c6c8bcec272568f71495b48e8	fig|550537.5.peg.2370	Histidine ABC transporter, ATP-binding protein HisP (TC 3.A.1.3.1)
32f3803c6c8bcec272568f71495b48e8	fig|550538.3.peg.2529	Histidine ABC transporter, ATP-binding protein HisP (TC 3.A.1.3.1)
32f3803c6c8bcec272568f71495b48e8	fig|550538.5.peg.2522	Histidine ABC transporter, ATP-binding protein HisP (TC 3.A.1.3.1)
32f3803c6c8bcec272568f71495b48e8	fig|568708.3.peg.2512	Histidine ABC transporter, ATP-binding protein HisP (TC 3.A.1.3.1)
32f3803c6c8bcec272568f71495b48e8	fig|573395.3.peg.3015	Histidine ABC transporter, ATP-binding protein HisP (TC 3.A.1.3.1)
32f3803c6c8bcec272568f71495b48e8	fig|588858.6.peg.2693	Histidine ABC transporter, ATP-binding protein HisP (TC 3.A.1.3.1)
32f3803c6c8bcec272568f71495b48e8	fig|99287.1.peg.2274	Histidine ABC transporter, ATP-binding protein HisP (TC 3.A.1.3.1)
38b4db6538bce110ff050407026631c8	fig|216599.1.peg.3666	L-carnitine/gamma-butyrobetaine antiporter
38b4db6538bce110ff050407026631c8	fig|300269.11.peg.55	L-carnitine/gamma-butyrobetaine antiporter
38b4db6538bce110ff050407026631c8	fig|300269.12.peg.60	L-carnitine/gamma-butyrobetaine antiporter
38b4db6538bce110ff050407026631c8	fig|316385.7.peg.40	L-carnitine/gamma-butyrobetaine antiporter
38b4db6538bce110ff050407026631c8	fig|316401.4.peg.45	L-carnitine/gamma-butyrobetaine antiporter
38b4db6538bce110ff050407026631c8	fig|316407.3.peg.40	L-carnitine/gamma-butyrobetaine antiporter
38b4db6538bce110ff050407026631c8	fig|331111.12.peg.371	L-carnitine/gamma-butyrobetaine antiporter
38b4db6538bce110ff050407026631c8	fig|331111.3.peg.2613	L-carnitine/gamma-butyrobetaine antiporter
38b4db6538bce110ff050407026631c8	fig|331112.3.peg.40	L-carnitine/gamma-butyrobetaine antiporter
38b4db6538bce110ff050407026631c8	fig|331112.6.peg.43	L-carnitine/gamma-butyrobetaine antiporter
38b4db6538bce110ff050407026631c8	fig|340185.3.peg.4595	L-carnitine/gamma-butyrobetaine antiporter
38b4db6538bce110ff050407026631c8	fig|340185.4.peg.4843	L-carnitine/gamma-butyrobetaine antiporter
38b4db6538bce110ff050407026631c8	fig|340186.3.peg.4199	L-carnitine/gamma-butyrobetaine antiporter
38b4db6538bce110ff050407026631c8	fig|340186.5.peg.4414	L-carnitine/gamma-butyrobetaine antiporter
38b4db6538bce110ff050407026631c8	fig|344610.3.peg.789	L-carnitine/gamma-butyrobetaine antiporter
38b4db6538bce110ff050407026631c8	fig|344610.7.peg.1702	L-carnitine/gamma-butyrobetaine antiporter
38b4db6538bce110ff050407026631c8	fig|364106.7.peg.175	L-carnitine/gamma-butyrobetaine antiporter
38b4db6538bce110ff050407026631c8	fig|364106.8.peg.174	L-carnitine/gamma-butyrobetaine antiporter
38b4db6538bce110ff050407026631c8	fig|405955.13.peg.43	L-carnitine/gamma-butyrobetaine antiporter
38b4db6538bce110ff050407026631c8	fig|405955.9.peg.34	L-carnitine/gamma-butyrobetaine antiporter
38b4db6538bce110ff050407026631c8	fig|469598.5.peg.3821	L-carnitine/gamma-butyrobetaine antiporter
38b4db6538bce110ff050407026631c8	fig|511145.12.peg.39	L-carnitine/gamma-butyrobetaine antiporter
38b4db6538bce110ff050407026631c8	fig|511145.6.peg.40	L-carnitine/gamma-butyrobetaine antiporter
38b4db6538bce110ff050407026631c8	fig|536056.3.peg.3765	L-carnitine/gamma-butyrobetaine antiporter
38b4db6538bce110ff050407026631c8	fig|550676.3.peg.4704	L-carnitine/gamma-butyrobetaine antiporter
38b4db6538bce110ff050407026631c8	fig|566546.3.peg.1694	L-carnitine/gamma-butyrobetaine antiporter
38b4db6538bce110ff050407026631c8	fig|566546.4.peg.39	L-carnitine/gamma-butyrobetaine antiporter
38b4db6538bce110ff050407026631c8	fig|573235.3.peg.40	L-carnitine/gamma-butyrobetaine antiporter
38b4db6538bce110ff050407026631c8	fig|585035.6.peg.43	L-carnitine/gamma-butyrobetaine antiporter
38b4db6538bce110ff050407026631c8	fig|585055.6.peg.41	L-carnitine/gamma-butyrobetaine antiporter
38b4db6538bce110ff050407026631c8	fig|585055.8.peg.41	L-carnitine/gamma-butyrobetaine antiporter
38b4db6538bce110ff050407026631c8	fig|585056.7.peg.227	L-carnitine/gamma-butyrobetaine antiporter
38b4db6538bce110ff050407026631c8	fig|585396.4.peg.39	L-carnitine/gamma-butyrobetaine antiporter
38b4db6538bce110ff050407026631c8	fig|595495.4.peg.1687	L-carnitine/gamma-butyrobetaine antiporter
38b4db6538bce110ff050407026631c8	fig|595496.3.peg.40	L-carnitine/gamma-butyrobetaine antiporter
38b4db6538bce110ff050407026631c8	fig|656379.3.peg.278	L-carnitine/gamma-butyrobetaine antiporter
38b4db6538bce110ff050407026631c8	fig|656380.3.peg.215	L-carnitine/gamma-butyrobetaine antiporter
38b4db6538bce110ff050407026631c8	fig|656393.3.peg.650	L-carnitine/gamma-butyrobetaine antiporter
38b4db6538bce110ff050407026631c8	fig|656419.3.peg.194	L-carnitine/gamma-butyrobetaine antiporter
38b4db6538bce110ff050407026631c8	fig|656437.3.peg.94	L-carnitine/gamma-butyrobetaine antiporter
38b4db6538bce110ff050407026631c8	fig|656444.3.peg.367	L-carnitine/gamma-butyrobetaine antiporter
38b4db6538bce110ff050407026631c8	fig|6666666.5522.peg.4113	L-carnitine/gamma-butyrobetaine antiporter
38b4db6538bce110ff050407026631c8	fig|670888.3.peg.619	L-carnitine/gamma-butyrobetaine antiporter
38b4db6538bce110ff050407026631c8	fig|679204.3.peg.3148	L-carnitine/gamma-butyrobetaine antiporter
38b4db6538bce110ff050407026631c8	fig|679205.4.peg.3548	L-carnitine/gamma-butyrobetaine antiporter
38b4db6538bce110ff050407026631c8	fig|714962.3.peg.41	L-carnitine/gamma-butyrobetaine antiporter
38b4db6538bce110ff050407026631c8	fig|749531.3.peg.1238	L-carnitine/gamma-butyrobetaine antiporter
38b4db6538bce110ff050407026631c8	fig|749532.3.peg.4229	L-carnitine/gamma-butyrobetaine antiporter
38b4db6538bce110ff050407026631c8	fig|749537.3.peg.3130	L-carnitine/gamma-butyrobetaine antiporter
38b4db6538bce110ff050407026631c8	fig|749538.3.peg.2046	L-carnitine/gamma-butyrobetaine antiporter
38b4db6538bce110ff050407026631c8	fig|749544.3.peg.2656	L-carnitine/gamma-butyrobetaine antiporter
38b4db6538bce110ff050407026631c8	fig|749545.3.peg.2070	L-carnitine/gamma-butyrobetaine antiporter
38b4db6538bce110ff050407026631c8	fig|749548.3.peg.2387	L-carnitine/gamma-butyrobetaine antiporter
38b4db6538bce110ff050407026631c8	fig|749549.3.peg.4615	L-carnitine/gamma-butyrobetaine antiporter
38b4db6538bce110ff050407026631c8	fig|753642.3.peg.3598	L-carnitine/gamma-butyrobetaine antiporter
38b4db6538bce110ff050407026631c8	fig|83333.1.peg.41	L-carnitine/gamma-butyrobetaine antiporter
38b4db6538bce110ff050407026631c8	fig|869729.3.peg.4941	L-carnitine/gamma-butyrobetaine antiporter
a0bf80f126384bc8680e7a03b139d49c	fig|3702.1.peg.28183	Pyrophosphate--fructose 6-phosphate 1-phosphotransferase, alpha subunit (EC 2.7.1.90)
a0bf80f126384bc8680e7a03b139d49c	fig|3702.7.peg.27505	Pyrophosphate--fructose 6-phosphate 1-phosphotransferase, alpha subunit (EC 2.7.1.90)
55f5a9e101748ad0f2cc1f1f6558e4ee	fig|471223.3.peg.2599	Proton/glutamate symport protein @ Sodium/glutamate symport protein
f17c8c71b27ae8c4a82de40baa5d4ddd	fig|216597.6.peg.4561	Thiazole biosynthesis protein ThiH
f17c8c71b27ae8c4a82de40baa5d4ddd	fig|28901.42.peg.5116	Thiazole biosynthesis protein ThiH
f17c8c71b27ae8c4a82de40baa5d4ddd	fig|423368.6.peg.4491	Thiazole biosynthesis protein ThiH
f17c8c71b27ae8c4a82de40baa5d4ddd	fig|423368.8.peg.4472	Thiazole biosynthesis protein ThiH
f17c8c71b27ae8c4a82de40baa5d4ddd	fig|440534.5.peg.4591	Thiazole biosynthesis protein ThiH
f17c8c71b27ae8c4a82de40baa5d4ddd	fig|568708.3.peg.4389	Thiazole biosynthesis protein ThiH
f17c8c71b27ae8c4a82de40baa5d4ddd	fig|588858.6.peg.4529	Thiazole biosynthesis protein ThiH
f17c8c71b27ae8c4a82de40baa5d4ddd	fig|99287.12.peg.4373	Thiazole biosynthesis protein ThiH
f17c8c71b27ae8c4a82de40baa5d4ddd	fig|99287.1.peg.4001	Thiazole biosynthesis protein ThiH
0c7424555e1316758e203742149c7df2	fig|210007.7.peg.1063	PTS system, mannitol-specific IIB component (EC 2.7.1.69) / PTS system, mannitol-specific IIC component (EC 2.7.1.69)
83f848873c0472f6e4404e2eb3ade331	fig|178306.1.peg.2294	Acetyl-CoA synthetase (ADP-forming) alpha chain (EC 6.2.1.13)
42d497419a55e8f85fbbf0897fb40734	fig|316385.5.peg.183	Methylglyoxal reductase, acetol producing (EC 1.1.1.-) / 2,5-diketo-D-gluconic acid reductase B (EC 1.1.1.274)
42d497419a55e8f85fbbf0897fb40734	fig|316385.7.peg.186	Methylglyoxal reductase, acetol producing (EC 1.1.1.-) / 2,5-diketo-D-gluconic acid reductase B (EC 1.1.1.274)
42d497419a55e8f85fbbf0897fb40734	fig|316407.3.peg.198	Methylglyoxal reductase, acetol producing (EC 1.1.1.-) / 2,5-diketo-D-gluconic acid reductase B (EC 1.1.1.274)
42d497419a55e8f85fbbf0897fb40734	fig|344610.3.peg.2603	Methylglyoxal reductase, acetol producing (EC 1.1.1.-) / 2,5-diketo-D-gluconic acid reductase B (EC 1.1.1.274)
42d497419a55e8f85fbbf0897fb40734	fig|344610.7.peg.1476	Methylglyoxal reductase, acetol producing (EC 1.1.1.-) / 2,5-diketo-D-gluconic acid reductase B (EC 1.1.1.274)
42d497419a55e8f85fbbf0897fb40734	fig|409438.11.peg.325	Methylglyoxal reductase, acetol producing (EC 1.1.1.-) / 2,5-diketo-D-gluconic acid reductase B (EC 1.1.1.274)
42d497419a55e8f85fbbf0897fb40734	fig|457401.3.peg.2461	Methylglyoxal reductase, acetol producing (EC 1.1.1.-) / 2,5-diketo-D-gluconic acid reductase B (EC 1.1.1.274)
42d497419a55e8f85fbbf0897fb40734	fig|511145.12.peg.209	Methylglyoxal reductase, acetol producing (EC 1.1.1.-) / 2,5-diketo-D-gluconic acid reductase B (EC 1.1.1.274)
42d497419a55e8f85fbbf0897fb40734	fig|511145.6.peg.208	Methylglyoxal reductase, acetol producing (EC 1.1.1.-) / 2,5-diketo-D-gluconic acid reductase B (EC 1.1.1.274)
42d497419a55e8f85fbbf0897fb40734	fig|536056.3.peg.3594	Methylglyoxal reductase, acetol producing (EC 1.1.1.-) / 2,5-diketo-D-gluconic acid reductase B (EC 1.1.1.274)
42d497419a55e8f85fbbf0897fb40734	fig|556266.3.peg.2988	Methylglyoxal reductase, acetol producing (EC 1.1.1.-) / 2,5-diketo-D-gluconic acid reductase B (EC 1.1.1.274)
42d497419a55e8f85fbbf0897fb40734	fig|585034.4.peg.208	Methylglyoxal reductase, acetol producing (EC 1.1.1.-) / 2,5-diketo-D-gluconic acid reductase B (EC 1.1.1.274)
42d497419a55e8f85fbbf0897fb40734	fig|585034.5.peg.208	Methylglyoxal reductase, acetol producing (EC 1.1.1.-) / 2,5-diketo-D-gluconic acid reductase B (EC 1.1.1.274)
42d497419a55e8f85fbbf0897fb40734	fig|595496.3.peg.209	Methylglyoxal reductase, acetol producing (EC 1.1.1.-) / 2,5-diketo-D-gluconic acid reductase B (EC 1.1.1.274)
42d497419a55e8f85fbbf0897fb40734	fig|656408.3.peg.131	Methylglyoxal reductase, acetol producing (EC 1.1.1.-) / 2,5-diketo-D-gluconic acid reductase B (EC 1.1.1.274)
42d497419a55e8f85fbbf0897fb40734	fig|656414.3.peg.333	Methylglyoxal reductase, acetol producing (EC 1.1.1.-) / 2,5-diketo-D-gluconic acid reductase B (EC 1.1.1.274)
42d497419a55e8f85fbbf0897fb40734	fig|656443.3.peg.355	Methylglyoxal reductase, acetol producing (EC 1.1.1.-) / 2,5-diketo-D-gluconic acid reductase B (EC 1.1.1.274)
42d497419a55e8f85fbbf0897fb40734	fig|679205.4.peg.4222	Methylglyoxal reductase, acetol producing (EC 1.1.1.-) / 2,5-diketo-D-gluconic acid reductase B (EC 1.1.1.274)
42d497419a55e8f85fbbf0897fb40734	fig|679206.4.peg.3168	Methylglyoxal reductase, acetol producing (EC 1.1.1.-) / 2,5-diketo-D-gluconic acid reductase B (EC 1.1.1.274)
42d497419a55e8f85fbbf0897fb40734	fig|749533.3.peg.3450	Methylglyoxal reductase, acetol producing (EC 1.1.1.-) / 2,5-diketo-D-gluconic acid reductase B (EC 1.1.1.274)
42d497419a55e8f85fbbf0897fb40734	fig|749538.3.peg.2654	Methylglyoxal reductase, acetol producing (EC 1.1.1.-) / 2,5-diketo-D-gluconic acid reductase B (EC 1.1.1.274)
42d497419a55e8f85fbbf0897fb40734	fig|749540.3.peg.2008	Methylglyoxal reductase, acetol producing (EC 1.1.1.-) / 2,5-diketo-D-gluconic acid reductase B (EC 1.1.1.274)
42d497419a55e8f85fbbf0897fb40734	fig|749548.3.peg.4566	Methylglyoxal reductase, acetol producing (EC 1.1.1.-) / 2,5-diketo-D-gluconic acid reductase B (EC 1.1.1.274)
42d497419a55e8f85fbbf0897fb40734	fig|83333.1.peg.202	Methylglyoxal reductase, acetol producing (EC 1.1.1.-) / 2,5-diketo-D-gluconic acid reductase B (EC 1.1.1.274)
03fa755ed4c3cb2fbab103e0feddbfc8	fig|281310.6.peg.1882	Enoyl-[acyl-carrier-protein] reductase [NADH] (EC 1.3.1.9)
03fa755ed4c3cb2fbab103e0feddbfc8	fig|374927.4.peg.1439	Enoyl-[acyl-carrier-protein] reductase [NADH] (EC 1.3.1.9)
03fa755ed4c3cb2fbab103e0feddbfc8	fig|374930.8.peg.669	Enoyl-[acyl-carrier-protein] reductase [NADH] (EC 1.3.1.9)
03fa755ed4c3cb2fbab103e0feddbfc8	fig|374930.9.peg.681	Enoyl-[acyl-carrier-protein] reductase [NADH] (EC 1.3.1.9)
03fa755ed4c3cb2fbab103e0feddbfc8	fig|521005.3.peg.1596	Enoyl-[acyl-carrier-protein] reductase [NADH] (EC 1.3.1.9)
03fa755ed4c3cb2fbab103e0feddbfc8	fig|656912.3.peg.1288	Enoyl-[acyl-carrier-protein] reductase [NADH] (EC 1.3.1.9)
03fa755ed4c3cb2fbab103e0feddbfc8	fig|656913.3.peg.1287	Enoyl-[acyl-carrier-protein] reductase [NADH] (EC 1.3.1.9)
03fa755ed4c3cb2fbab103e0feddbfc8	fig|71421.1.peg.1646	Enoyl-[acyl-carrier-protein] reductase [NADH] (EC 1.3.1.9)
03fa755ed4c3cb2fbab103e0feddbfc8	fig|71421.8.peg.1815	Enoyl-[acyl-carrier-protein] reductase [NADH] (EC 1.3.1.9)
03fa755ed4c3cb2fbab103e0feddbfc8	fig|866630.3.peg.404	Enoyl-[acyl-carrier-protein] reductase [NADH] (EC 1.3.1.9)
d2e7d0b083eaf00e77aaa538711b503e	fig|316385.5.peg.2673	Probable 3-phenylpropionic acid transporter ## PnuD
d2e7d0b083eaf00e77aaa538711b503e	fig|316385.7.peg.2731	Probable 3-phenylpropionic acid transporter
d2e7d0b083eaf00e77aaa538711b503e	fig|316407.3.peg.2458	Probable 3-phenylpropionic acid transporter ## PnuD
d2e7d0b083eaf00e77aaa538711b503e	fig|331111.12.peg.3099	Probable 3-phenylpropionic acid transporter
d2e7d0b083eaf00e77aaa538711b503e	fig|331111.3.peg.549	Probable 3-phenylpropionic acid transporter ## PnuD
d2e7d0b083eaf00e77aaa538711b503e	fig|340185.3.peg.2394	Probable 3-phenylpropionic acid transporter ## PnuD
d2e7d0b083eaf00e77aaa538711b503e	fig|340185.4.peg.2534	Probable 3-phenylpropionic acid transporter
d2e7d0b083eaf00e77aaa538711b503e	fig|344601.3.peg.1656	Probable 3-phenylpropionic acid transporter ## PnuD
d2e7d0b083eaf00e77aaa538711b503e	fig|344601.5.peg.1730	Probable 3-phenylpropionic acid transporter
d2e7d0b083eaf00e77aaa538711b503e	fig|457401.3.peg.858	Probable 3-phenylpropionic acid transporter
d2e7d0b083eaf00e77aaa538711b503e	fig|481805.3.peg.1220	Probable 3-phenylpropionic acid transporter ## PnuD
d2e7d0b083eaf00e77aaa538711b503e	fig|481805.6.peg.1218	Probable 3-phenylpropionic acid transporter
d2e7d0b083eaf00e77aaa538711b503e	fig|511145.12.peg.2637	Probable 3-phenylpropionic acid transporter
d2e7d0b083eaf00e77aaa538711b503e	fig|511145.6.peg.2623	Probable 3-phenylpropionic acid transporter ## PnuD
d2e7d0b083eaf00e77aaa538711b503e	fig|536056.3.peg.1190	Probable 3-phenylpropionic acid transporter
d2e7d0b083eaf00e77aaa538711b503e	fig|550672.3.peg.2267	Probable 3-phenylpropionic acid transporter
d2e7d0b083eaf00e77aaa538711b503e	fig|550677.3.peg.2420	Probable 3-phenylpropionic acid transporter
d2e7d0b083eaf00e77aaa538711b503e	fig|556266.3.peg.643	Probable 3-phenylpropionic acid transporter
d2e7d0b083eaf00e77aaa538711b503e	fig|562.375.peg.3523	Probable 3-phenylpropionic acid transporter
d2e7d0b083eaf00e77aaa538711b503e	fig|566546.3.peg.3873	Probable 3-phenylpropionic acid transporter
d2e7d0b083eaf00e77aaa538711b503e	fig|566546.4.peg.2735	Probable 3-phenylpropionic acid transporter ## PnuD
d2e7d0b083eaf00e77aaa538711b503e	fig|573235.3.peg.3653	Probable 3-phenylpropionic acid transporter
d2e7d0b083eaf00e77aaa538711b503e	fig|585055.6.peg.2858	Probable 3-phenylpropionic acid transporter
d2e7d0b083eaf00e77aaa538711b503e	fig|585055.8.peg.2862	Probable 3-phenylpropionic acid transporter
d2e7d0b083eaf00e77aaa538711b503e	fig|585056.7.peg.3043	Probable 3-phenylpropionic acid transporter
d2e7d0b083eaf00e77aaa538711b503e	fig|585395.4.peg.3198	Probable 3-phenylpropionic acid transporter
d2e7d0b083eaf00e77aaa538711b503e	fig|585396.4.peg.3365	Probable 3-phenylpropionic acid transporter
d2e7d0b083eaf00e77aaa538711b503e	fig|595495.4.peg.3332	Probable 3-phenylpropionic acid transporter
d2e7d0b083eaf00e77aaa538711b503e	fig|595496.3.peg.2519	Probable 3-phenylpropionic acid transporter
d2e7d0b083eaf00e77aaa538711b503e	fig|656379.3.peg.3170	Probable 3-phenylpropionic acid transporter
d2e7d0b083eaf00e77aaa538711b503e	fig|656380.3.peg.2620	Probable 3-phenylpropionic acid transporter
d2e7d0b083eaf00e77aaa538711b503e	fig|656408.3.peg.2829	Probable 3-phenylpropionic acid transporter
d2e7d0b083eaf00e77aaa538711b503e	fig|656437.3.peg.2867	Probable 3-phenylpropionic acid transporter
d2e7d0b083eaf00e77aaa538711b503e	fig|656443.3.peg.3297	Probable 3-phenylpropionic acid transporter
d2e7d0b083eaf00e77aaa538711b503e	fig|6666666.5522.peg.686	Probable 3-phenylpropionic acid transporter ## PnuD
d2e7d0b083eaf00e77aaa538711b503e	fig|679205.4.peg.1420	Probable 3-phenylpropionic acid transporter
d2e7d0b083eaf00e77aaa538711b503e	fig|679206.4.peg.572	Probable 3-phenylpropionic acid transporter
d2e7d0b083eaf00e77aaa538711b503e	fig|679207.4.peg.2669	Probable 3-phenylpropionic acid transporter
d2e7d0b083eaf00e77aaa538711b503e	fig|749531.3.peg.3824	Probable 3-phenylpropionic acid transporter
d2e7d0b083eaf00e77aaa538711b503e	fig|749532.3.peg.4461	Probable 3-phenylpropionic acid transporter
d2e7d0b083eaf00e77aaa538711b503e	fig|749533.3.peg.2245	Probable 3-phenylpropionic acid transporter
d2e7d0b083eaf00e77aaa538711b503e	fig|749537.3.peg.4117	Probable 3-phenylpropionic acid transporter
d2e7d0b083eaf00e77aaa538711b503e	fig|749538.3.peg.4862	Probable 3-phenylpropionic acid transporter
d2e7d0b083eaf00e77aaa538711b503e	fig|749540.3.peg.4573	Probable 3-phenylpropionic acid transporter
d2e7d0b083eaf00e77aaa538711b503e	fig|749545.3.peg.4822	Probable 3-phenylpropionic acid transporter
d2e7d0b083eaf00e77aaa538711b503e	fig|749548.3.peg.1324	Probable 3-phenylpropionic acid transporter
d2e7d0b083eaf00e77aaa538711b503e	fig|749549.3.peg.2375	Probable 3-phenylpropionic acid transporter
d2e7d0b083eaf00e77aaa538711b503e	fig|83333.1.peg.2506	Probable 3-phenylpropionic acid transporter ## PnuD
05afc641074561bb7c3cff9689e350ff	fig|155864.1.peg.4508	2-amino-3-ketobutyrate coenzyme A ligase (EC 2.3.1.29)
05afc641074561bb7c3cff9689e350ff	fig|155864.8.peg.4470	2-amino-3-ketobutyrate coenzyme A ligase (EC 2.3.1.29)
05afc641074561bb7c3cff9689e350ff	fig|198214.1.peg.3437	2-amino-3-ketobutyrate coenzyme A ligase (EC 2.3.1.29)
05afc641074561bb7c3cff9689e350ff	fig|198214.7.peg.4319	2-amino-3-ketobutyrate coenzyme A ligase (EC 2.3.1.29)
05afc641074561bb7c3cff9689e350ff	fig|198215.1.peg.3534	2-amino-3-ketobutyrate coenzyme A ligase (EC 2.3.1.29)
05afc641074561bb7c3cff9689e350ff	fig|198215.6.peg.4474	2-amino-3-ketobutyrate coenzyme A ligase (EC 2.3.1.29)
05afc641074561bb7c3cff9689e350ff	fig|216593.1.peg.4360	2-amino-3-ketobutyrate coenzyme A ligase (EC 2.3.1.29)
05afc641074561bb7c3cff9689e350ff	fig|316385.5.peg.3751	2-amino-3-ketobutyrate coenzyme A ligase (EC 2.3.1.29)
05afc641074561bb7c3cff9689e350ff	fig|316385.7.peg.3837	2-amino-3-ketobutyrate coenzyme A ligase (EC 2.3.1.29)
05afc641074561bb7c3cff9689e350ff	fig|316401.4.peg.4389	2-amino-3-ketobutyrate coenzyme A ligase (EC 2.3.1.29)
05afc641074561bb7c3cff9689e350ff	fig|316407.3.peg.3510	2-amino-3-ketobutyrate coenzyme A ligase (EC 2.3.1.29)
05afc641074561bb7c3cff9689e350ff	fig|331111.12.peg.4362	2-amino-3-ketobutyrate coenzyme A ligase (EC 2.3.1.29)
05afc641074561bb7c3cff9689e350ff	fig|331111.3.peg.1763	2-amino-3-ketobutyrate coenzyme A ligase (EC 2.3.1.29)
05afc641074561bb7c3cff9689e350ff	fig|340184.3.peg.3257	2-amino-3-ketobutyrate coenzyme A ligase (EC 2.3.1.29)
05afc641074561bb7c3cff9689e350ff	fig|340184.6.peg.3400	2-amino-3-ketobutyrate coenzyme A ligase (EC 2.3.1.29)
05afc641074561bb7c3cff9689e350ff	fig|340185.3.peg.4490	2-amino-3-ketobutyrate coenzyme A ligase (EC 2.3.1.29)
05afc641074561bb7c3cff9689e350ff	fig|340185.4.peg.4736	2-amino-3-ketobutyrate coenzyme A ligase (EC 2.3.1.29)
05afc641074561bb7c3cff9689e350ff	fig|344601.3.peg.1820	2-amino-3-ketobutyrate coenzyme A ligase (EC 2.3.1.29)
05afc641074561bb7c3cff9689e350ff	fig|344601.5.peg.1900	2-amino-3-ketobutyrate coenzyme A ligase (EC 2.3.1.29)
05afc641074561bb7c3cff9689e350ff	fig|358708.5.peg.127	2-amino-3-ketobutyrate coenzyme A ligase (EC 2.3.1.29)
05afc641074561bb7c3cff9689e350ff	fig|386585.9.peg.4711	2-amino-3-ketobutyrate coenzyme A ligase (EC 2.3.1.29)
05afc641074561bb7c3cff9689e350ff	fig|409438.11.peg.4073	2-amino-3-ketobutyrate coenzyme A ligase (EC 2.3.1.29)
05afc641074561bb7c3cff9689e350ff	fig|444447.5.peg.1967	2-amino-3-ketobutyrate coenzyme A ligase (EC 2.3.1.29)
05afc641074561bb7c3cff9689e350ff	fig|444448.5.peg.1805	2-amino-3-ketobutyrate coenzyme A ligase (EC 2.3.1.29)
05afc641074561bb7c3cff9689e350ff	fig|444449.5.peg.3050	2-amino-3-ketobutyrate coenzyme A ligase (EC 2.3.1.29)
05afc641074561bb7c3cff9689e350ff	fig|444450.8.peg.4886	2-amino-3-ketobutyrate coenzyme A ligase (EC 2.3.1.29)
05afc641074561bb7c3cff9689e350ff	fig|444451.5.peg.180	2-amino-3-ketobutyrate coenzyme A ligase (EC 2.3.1.29)
05afc641074561bb7c3cff9689e350ff	fig|444452.5.peg.6	2-amino-3-ketobutyrate coenzyme A ligase (EC 2.3.1.29)
05afc641074561bb7c3cff9689e350ff	fig|444453.5.peg.1334	2-amino-3-ketobutyrate coenzyme A ligase (EC 2.3.1.29)
05afc641074561bb7c3cff9689e350ff	fig|444454.5.peg.3596	2-amino-3-ketobutyrate coenzyme A ligase (EC 2.3.1.29)
05afc641074561bb7c3cff9689e350ff	fig|478004.5.peg.361	2-amino-3-ketobutyrate coenzyme A ligase (EC 2.3.1.29)
05afc641074561bb7c3cff9689e350ff	fig|478005.5.peg.314	2-amino-3-ketobutyrate coenzyme A ligase (EC 2.3.1.29)
05afc641074561bb7c3cff9689e350ff	fig|478006.5.peg.328	2-amino-3-ketobutyrate coenzyme A ligase (EC 2.3.1.29)
05afc641074561bb7c3cff9689e350ff	fig|478007.5.peg.334	2-amino-3-ketobutyrate coenzyme A ligase (EC 2.3.1.29)
05afc641074561bb7c3cff9689e350ff	fig|478008.5.peg.856	2-amino-3-ketobutyrate coenzyme A ligase (EC 2.3.1.29)
05afc641074561bb7c3cff9689e350ff	fig|502346.5.peg.2132	2-amino-3-ketobutyrate coenzyme A ligase (EC 2.3.1.29)
05afc641074561bb7c3cff9689e350ff	fig|511145.12.peg.3737	2-amino-3-ketobutyrate coenzyme A ligase (EC 2.3.1.29)
05afc641074561bb7c3cff9689e350ff	fig|511145.6.peg.3719	2-amino-3-ketobutyrate coenzyme A ligase (EC 2.3.1.29)
05afc641074561bb7c3cff9689e350ff	fig|536056.3.peg.90	2-amino-3-ketobutyrate coenzyme A ligase (EC 2.3.1.29)
05afc641074561bb7c3cff9689e350ff	fig|544404.4.peg.4697	2-amino-3-ketobutyrate coenzyme A ligase (EC 2.3.1.29)
05afc641074561bb7c3cff9689e350ff	fig|550676.3.peg.3814	2-amino-3-ketobutyrate coenzyme A ligase (EC 2.3.1.29)
05afc641074561bb7c3cff9689e350ff	fig|550677.3.peg.4194	2-amino-3-ketobutyrate coenzyme A ligase (EC 2.3.1.29)
05afc641074561bb7c3cff9689e350ff	fig|562.371.peg.3175	2-amino-3-ketobutyrate coenzyme A ligase (EC 2.3.1.29)
05afc641074561bb7c3cff9689e350ff	fig|562.372.peg.4058	2-amino-3-ketobutyrate coenzyme A ligase (EC 2.3.1.29)
05afc641074561bb7c3cff9689e350ff	fig|562.373.peg.1590	2-amino-3-ketobutyrate coenzyme A ligase (EC 2.3.1.29)
05afc641074561bb7c3cff9689e350ff	fig|562.374.peg.1182	2-amino-3-ketobutyrate coenzyme A ligase (EC 2.3.1.29)
05afc641074561bb7c3cff9689e350ff	fig|570506.3.peg.637	2-amino-3-ketobutyrate coenzyme A ligase (EC 2.3.1.29)
05afc641074561bb7c3cff9689e350ff	fig|573235.3.peg.5130	2-amino-3-ketobutyrate coenzyme A ligase (EC 2.3.1.29)
05afc641074561bb7c3cff9689e350ff	fig|574521.7.peg.3959	2-amino-3-ketobutyrate coenzyme A ligase (EC 2.3.1.29)
05afc641074561bb7c3cff9689e350ff	fig|585034.4.peg.3708	2-amino-3-ketobutyrate coenzyme A ligase (EC 2.3.1.29)
05afc641074561bb7c3cff9689e350ff	fig|585034.5.peg.3705	2-amino-3-ketobutyrate coenzyme A ligase (EC 2.3.1.29)
05afc641074561bb7c3cff9689e350ff	fig|585056.7.peg.4309	2-amino-3-ketobutyrate coenzyme A ligase (EC 2.3.1.29)
05afc641074561bb7c3cff9689e350ff	fig|585395.4.peg.4775	2-amino-3-ketobutyrate coenzyme A ligase (EC 2.3.1.29)
05afc641074561bb7c3cff9689e350ff	fig|591020.3.peg.4283	2-amino-3-ketobutyrate coenzyme A ligase (EC 2.3.1.29)
05afc641074561bb7c3cff9689e350ff	fig|595496.3.peg.3619	2-amino-3-ketobutyrate coenzyme A ligase (EC 2.3.1.29)
05afc641074561bb7c3cff9689e350ff	fig|621.8.peg.2877	2-amino-3-ketobutyrate coenzyme A ligase (EC 2.3.1.29)
05afc641074561bb7c3cff9689e350ff	fig|622.8.peg.4707	2-amino-3-ketobutyrate coenzyme A ligase (EC 2.3.1.29)
05afc641074561bb7c3cff9689e350ff	fig|637388.3.peg.1271	2-amino-3-ketobutyrate coenzyme A ligase (EC 2.3.1.29)
05afc641074561bb7c3cff9689e350ff	fig|656379.3.peg.4955	2-amino-3-ketobutyrate coenzyme A ligase (EC 2.3.1.29)
05afc641074561bb7c3cff9689e350ff	fig|656380.3.peg.4743	2-amino-3-ketobutyrate coenzyme A ligase (EC 2.3.1.29)
05afc641074561bb7c3cff9689e350ff	fig|656393.3.peg.4713	2-amino-3-ketobutyrate coenzyme A ligase (EC 2.3.1.29)
05afc641074561bb7c3cff9689e350ff	fig|656408.3.peg.4071	2-amino-3-ketobutyrate coenzyme A ligase (EC 2.3.1.29)
05afc641074561bb7c3cff9689e350ff	fig|656437.3.peg.4075	2-amino-3-ketobutyrate coenzyme A ligase (EC 2.3.1.29)
05afc641074561bb7c3cff9689e350ff	fig|656443.3.peg.4550	2-amino-3-ketobutyrate coenzyme A ligase (EC 2.3.1.29)
05afc641074561bb7c3cff9689e350ff	fig|656444.3.peg.4935	2-amino-3-ketobutyrate coenzyme A ligase (EC 2.3.1.29)
05afc641074561bb7c3cff9689e350ff	fig|670897.3.peg.3715	2-amino-3-ketobutyrate coenzyme A ligase (EC 2.3.1.29)
05afc641074561bb7c3cff9689e350ff	fig|679204.3.peg.4555	2-amino-3-ketobutyrate coenzyme A ligase (EC 2.3.1.29)
05afc641074561bb7c3cff9689e350ff	fig|679205.4.peg.2366	2-amino-3-ketobutyrate coenzyme A ligase (EC 2.3.1.29)
05afc641074561bb7c3cff9689e350ff	fig|679206.4.peg.2164	2-amino-3-ketobutyrate coenzyme A ligase (EC 2.3.1.29)
05afc641074561bb7c3cff9689e350ff	fig|701177.3.peg.4383	2-amino-3-ketobutyrate coenzyme A ligase (EC 2.3.1.29)
05afc641074561bb7c3cff9689e350ff	fig|749531.3.peg.881	2-amino-3-ketobutyrate coenzyme A ligase (EC 2.3.1.29)
05afc641074561bb7c3cff9689e350ff	fig|749532.3.peg.373	2-amino-3-ketobutyrate coenzyme A ligase (EC 2.3.1.29)
05afc641074561bb7c3cff9689e350ff	fig|749533.3.peg.5288	2-amino-3-ketobutyrate coenzyme A ligase (EC 2.3.1.29)
05afc641074561bb7c3cff9689e350ff	fig|749537.3.peg.3337	2-amino-3-ketobutyrate coenzyme A ligase (EC 2.3.1.29)
05afc641074561bb7c3cff9689e350ff	fig|749545.3.peg.1522	2-amino-3-ketobutyrate coenzyme A ligase (EC 2.3.1.29)
05afc641074561bb7c3cff9689e350ff	fig|749549.3.peg.2557	2-amino-3-ketobutyrate coenzyme A ligase (EC 2.3.1.29)
05afc641074561bb7c3cff9689e350ff	fig|83333.1.peg.3553	2-amino-3-ketobutyrate coenzyme A ligase (EC 2.3.1.29)
05afc641074561bb7c3cff9689e350ff	fig|83334.1.peg.4466	2-amino-3-ketobutyrate coenzyme A ligase (EC 2.3.1.29)
163cfc8fd2865d5964911f73a6501378	fig|9606.3.peg.31666	Fructose-bisphosphate aldolase class I (EC 4.1.2.13)
8f36bde342970478d921f5c12a47e86d	fig|272623.1.peg.1887	Oligopeptide transport system permease protein OppB (TC 3.A.1.5.1)
8f36bde342970478d921f5c12a47e86d	fig|272623.7.peg.1972	Oligopeptide transport system permease protein oppB (TC 3.A.1.5.1)
6bf499b0ed2a55a20573294c362eec36	fig|316385.5.peg.181	Methionine ABC transporter ATP-binding protein
6bf499b0ed2a55a20573294c362eec36	fig|316385.7.peg.184	Methionine ABC transporter ATP-binding protein
6bf499b0ed2a55a20573294c362eec36	fig|316407.3.peg.196	Methionine ABC transporter ATP-binding protein
6bf499b0ed2a55a20573294c362eec36	fig|457401.3.peg.3705	Methionine ABC transporter ATP-binding protein
6bf499b0ed2a55a20573294c362eec36	fig|511145.12.peg.207	Methionine ABC transporter ATP-binding protein
6bf499b0ed2a55a20573294c362eec36	fig|511145.6.peg.206	Methionine ABC transporter ATP-binding protein
6bf499b0ed2a55a20573294c362eec36	fig|536056.3.peg.3596	Methionine ABC transporter ATP-binding protein
6bf499b0ed2a55a20573294c362eec36	fig|595496.3.peg.207	Methionine ABC transporter ATP-binding protein
6bf499b0ed2a55a20573294c362eec36	fig|656414.3.peg.330	Methionine ABC transporter ATP-binding protein
6bf499b0ed2a55a20573294c362eec36	fig|749538.3.peg.3000	Methionine ABC transporter ATP-binding protein
6bf499b0ed2a55a20573294c362eec36	fig|749540.3.peg.2984	Methionine ABC transporter ATP-binding protein
6bf499b0ed2a55a20573294c362eec36	fig|749544.3.peg.1854	Methionine ABC transporter ATP-binding protein
6bf499b0ed2a55a20573294c362eec36	fig|83333.1.peg.200	Methionine ABC transporter ATP-binding protein
31df0e8e11596a1a6e342188bf7bd6d7	fig|9598.2.peg.28461	Glycine cleavage system H protein
31df0e8e11596a1a6e342188bf7bd6d7	fig|9606.3.peg.12625	Glycine cleavage system H protein
31df0e8e11596a1a6e342188bf7bd6d7	fig|9606.3.peg.2571	Glycine cleavage system H protein
4410d3143bf3511fba0668dd8bfdb985	fig|379731.4.peg.445	Glycolate dehydrogenase (EC 1.1.99.14), FAD-binding subunit GlcE
4410d3143bf3511fba0668dd8bfdb985	fig|379731.5.peg.445	Glycolate dehydrogenase (EC 1.1.99.14), FAD-binding subunit GlcE
f2e879184c94b31e8bdf11268071aef5	fig|3702.1.peg.6044	Glutamine synthetase (EC 6.3.1.2), cytosolic
f2e879184c94b31e8bdf11268071aef5	fig|3702.7.peg.23995	Glutamine synthetase type II, eukaryotic (EC 6.3.1.2)
7b292310ebc60b2e2b11b9604111e416	fig|381666.6.peg.6134	Aldehyde dehydrogenase (EC 1.2.1.3)
7c5d418afa423a2305adf8e6a8e8e84e	fig|272942.6.peg.3176	Enoyl-CoA hydratase (EC 4.2.1.17)
4e97c2068758d0076cc2d47bb6638ecd	fig|316385.5.peg.3028	Glycine dehydrogenase [decarboxylating] (glycine cleavage system P protein) (EC 1.4.4.2)
4e97c2068758d0076cc2d47bb6638ecd	fig|316385.7.peg.3096	Glycine dehydrogenase [decarboxylating] (glycine cleavage system P protein) (EC 1.4.4.2)
4e97c2068758d0076cc2d47bb6638ecd	fig|316401.4.peg.3502	Glycine dehydrogenase [decarboxylating] (glycine cleavage system P protein) (EC 1.4.4.2)
4e97c2068758d0076cc2d47bb6638ecd	fig|316407.3.peg.2798	Glycine dehydrogenase [decarboxylating] (glycine cleavage system P protein) (EC 1.4.4.2)
4e97c2068758d0076cc2d47bb6638ecd	fig|358709.5.peg.1413	Glycine dehydrogenase [decarboxylating] (glycine cleavage system P protein) (EC 1.4.4.2)
4e97c2068758d0076cc2d47bb6638ecd	fig|457401.3.peg.2635	Glycine dehydrogenase [decarboxylating] (glycine cleavage system P protein) (EC 1.4.4.2)
4e97c2068758d0076cc2d47bb6638ecd	fig|511145.12.peg.2998	Glycine dehydrogenase [decarboxylating] (glycine cleavage system P protein) (EC 1.4.4.2)
4e97c2068758d0076cc2d47bb6638ecd	fig|511145.6.peg.2983	Glycine dehydrogenase [decarboxylating] (glycine cleavage system P protein) (EC 1.4.4.2)
4e97c2068758d0076cc2d47bb6638ecd	fig|536056.3.peg.828	Glycine dehydrogenase [decarboxylating] (glycine cleavage system P protein) (EC 1.4.4.2)
4e97c2068758d0076cc2d47bb6638ecd	fig|595496.3.peg.2881	Glycine dehydrogenase [decarboxylating] (glycine cleavage system P protein) (EC 1.4.4.2)
4e97c2068758d0076cc2d47bb6638ecd	fig|749537.3.peg.4619	Glycine dehydrogenase [decarboxylating] (glycine cleavage system P protein) (EC 1.4.4.2)
4e97c2068758d0076cc2d47bb6638ecd	fig|749538.3.peg.1159	Glycine dehydrogenase [decarboxylating] (glycine cleavage system P protein) (EC 1.4.4.2)
4e97c2068758d0076cc2d47bb6638ecd	fig|749540.3.peg.410	Glycine dehydrogenase [decarboxylating] (glycine cleavage system P protein) (EC 1.4.4.2)
4e97c2068758d0076cc2d47bb6638ecd	fig|749544.3.peg.190	Glycine dehydrogenase [decarboxylating] (glycine cleavage system P protein) (EC 1.4.4.2)
4e97c2068758d0076cc2d47bb6638ecd	fig|749548.3.peg.3530	Glycine dehydrogenase [decarboxylating] (glycine cleavage system P protein) (EC 1.4.4.2)
4e97c2068758d0076cc2d47bb6638ecd	fig|83333.1.peg.2855	Glycine dehydrogenase [decarboxylating] (glycine cleavage system P protein) (EC 1.4.4.2)
a467324b834006f7d43f56f890259284	fig|196627.13.peg.1159	Diaminopimelate decarboxylase (EC 4.1.1.20)
a467324b834006f7d43f56f890259284	fig|196627.14.peg.1163	Diaminopimelate decarboxylase (EC 4.1.1.20)
a467324b834006f7d43f56f890259284	fig|196627.4.peg.1134	Diaminopimelate decarboxylase (EC 4.1.1.20)
a2f986db349495ab8e137c64e44a0981	fig|224308.43.peg.1043	Substrate-specific component TrpP of tryptophan ECF transporter
a2f986db349495ab8e137c64e44a0981	fig|535024.3.peg.4277	Substrate-specific component TrpP of tryptophan ECF transporter
a2f986db349495ab8e137c64e44a0981	fig|535025.4.peg.2139	Substrate-specific component TrpP of tryptophan ECF transporter
a2f986db349495ab8e137c64e44a0981	fig|645657.3.peg.1966	Substrate-specific component TrpP of tryptophan ECF transporter
e747c1678f9adbfd0debf7483969847c	fig|665029.3.peg.12	Thiazole biosynthesis protein ThiG
e747c1678f9adbfd0debf7483969847c	fig|716540.3.peg.3537	Thiazole biosynthesis protein ThiG
492d0e2674ccb8be52474c262f1d3c65	fig|316385.5.peg.953	Transport ATP-binding protein CydC
492d0e2674ccb8be52474c262f1d3c65	fig|316385.7.peg.966	Transport ATP-binding protein CydC
492d0e2674ccb8be52474c262f1d3c65	fig|316407.3.peg.852	Transport ATP-binding protein CydC
492d0e2674ccb8be52474c262f1d3c65	fig|511145.12.peg.915	Transport ATP-binding protein CydC
492d0e2674ccb8be52474c262f1d3c65	fig|511145.6.peg.907	Transport ATP-binding protein CydC
492d0e2674ccb8be52474c262f1d3c65	fig|536056.3.peg.2911	Transport ATP-binding protein CydC
492d0e2674ccb8be52474c262f1d3c65	fig|595496.3.peg.813	Transport ATP-binding protein CydC
492d0e2674ccb8be52474c262f1d3c65	fig|83333.1.peg.871	Transport ATP-binding protein CydC
7f003f7603a3e17b62644ae5647f1eda	fig|266834.11.peg.3793	L-threonine 3-dehydrogenase (EC 1.1.1.103)
7f003f7603a3e17b62644ae5647f1eda	fig|266834.1.peg.3609	L-threonine 3-dehydrogenase (EC 1.1.1.103)
965129acfd775e91e078def44ea670b2	fig|216597.6.peg.4100	Lipopolysaccharide core biosynthesis protein RfaY
965129acfd775e91e078def44ea670b2	fig|28901.42.peg.4738	Lipopolysaccharide core biosynthesis protein RfaY
965129acfd775e91e078def44ea670b2	fig|295319.15.peg.3792	Lipopolysaccharide core biosynthesis protein RfaY
965129acfd775e91e078def44ea670b2	fig|295319.3.peg.2699	Lipopolysaccharide core biosynthesis protein RfaY
965129acfd775e91e078def44ea670b2	fig|439846.4.peg.4218	Lipopolysaccharide core biosynthesis protein RfaY
965129acfd775e91e078def44ea670b2	fig|440534.5.peg.4028	Lipopolysaccharide core biosynthesis protein RfaY
965129acfd775e91e078def44ea670b2	fig|454164.6.peg.1686	Lipopolysaccharide core biosynthesis protein RfaY
965129acfd775e91e078def44ea670b2	fig|454166.6.peg.3826	Lipopolysaccharide core biosynthesis protein RfaY
965129acfd775e91e078def44ea670b2	fig|454166.8.peg.3824	Lipopolysaccharide core biosynthesis protein RfaY
965129acfd775e91e078def44ea670b2	fig|454169.6.peg.3993	Lipopolysaccharide core biosynthesis protein RfaY
965129acfd775e91e078def44ea670b2	fig|454169.8.peg.3839	Lipopolysaccharide core biosynthesis protein RfaY
965129acfd775e91e078def44ea670b2	fig|554290.7.peg.3764	Lipopolysaccharide core biosynthesis protein RfaY
965129acfd775e91e078def44ea670b2	fig|554290.9.peg.3802	Lipopolysaccharide core biosynthesis protein RfaY
965129acfd775e91e078def44ea670b2	fig|568708.3.peg.3945	Lipopolysaccharide core biosynthesis protein RfaY
965129acfd775e91e078def44ea670b2	fig|573395.3.peg.791	Lipopolysaccharide core biosynthesis protein RfaY
965129acfd775e91e078def44ea670b2	fig|588858.6.peg.4085	Lipopolysaccharide core biosynthesis protein RfaY
965129acfd775e91e078def44ea670b2	fig|99287.12.peg.3930	Lipopolysaccharide core biosynthesis protein RfaY
965129acfd775e91e078def44ea670b2	fig|99287.1.peg.3592	Lipopolysaccharide core biosynthesis protein RfaY
180f88f6b6278de93b871d44559ff23c	fig|4932.3.peg.654	Citrate synthase (si) (EC 2.3.3.1)
180f88f6b6278de93b871d44559ff23c	fig|559292.3.peg.568	Citrate synthase (si) (EC 2.3.3.1)
37e66445f45b5bcdd9d5ae34e61223d8	fig|3702.1.peg.25264	Glutamine synthetase (EC 6.3.1.2), plastidic
37e66445f45b5bcdd9d5ae34e61223d8	fig|3702.7.peg.14712	Glutamine synthetase type II, eukaryotic (EC 6.3.1.2)
37e66445f45b5bcdd9d5ae34e61223d8	fig|3702.7.peg.14713	Glutamine synthetase type II, eukaryotic (EC 6.3.1.2)
37e66445f45b5bcdd9d5ae34e61223d8	fig|3702.7.peg.14714	Glutamine synthetase type II, eukaryotic (EC 6.3.1.2)
7a839c82814c9e723c9a5ca6d6674715	fig|316385.5.peg.1581	Respiratory nitrate reductase alpha chain (EC 1.7.99.4)
7a839c82814c9e723c9a5ca6d6674715	fig|316385.7.peg.1621	Respiratory nitrate reductase alpha chain (EC 1.7.99.4)
7a839c82814c9e723c9a5ca6d6674715	fig|316407.3.peg.1427	Respiratory nitrate reductase alpha chain (EC 1.7.99.4)
7a839c82814c9e723c9a5ca6d6674715	fig|358709.5.peg.959	Respiratory nitrate reductase alpha chain (EC 1.7.99.4)
7a839c82814c9e723c9a5ca6d6674715	fig|413997.3.peg.1500	Respiratory nitrate reductase alpha chain (EC 1.7.99.4)
7a839c82814c9e723c9a5ca6d6674715	fig|457401.3.peg.572	Respiratory nitrate reductase alpha chain (EC 1.7.99.4)
7a839c82814c9e723c9a5ca6d6674715	fig|469008.4.peg.2235	Respiratory nitrate reductase alpha chain (EC 1.7.99.4)
7a839c82814c9e723c9a5ca6d6674715	fig|481805.3.peg.2350	Respiratory nitrate reductase alpha chain (EC 1.7.99.4)
7a839c82814c9e723c9a5ca6d6674715	fig|481805.6.peg.2341	Respiratory nitrate reductase alpha chain (EC 1.7.99.4)
7a839c82814c9e723c9a5ca6d6674715	fig|511145.12.peg.1534	Respiratory nitrate reductase alpha chain (EC 1.7.99.4)
7a839c82814c9e723c9a5ca6d6674715	fig|511145.6.peg.1520	Respiratory nitrate reductase alpha chain (EC 1.7.99.4)
7a839c82814c9e723c9a5ca6d6674715	fig|511693.5.peg.1539	Respiratory nitrate reductase alpha chain (EC 1.7.99.4)
7a839c82814c9e723c9a5ca6d6674715	fig|536056.3.peg.2289	Respiratory nitrate reductase alpha chain (EC 1.7.99.4)
7a839c82814c9e723c9a5ca6d6674715	fig|595496.3.peg.1423	Respiratory nitrate reductase alpha chain (EC 1.7.99.4)
7a839c82814c9e723c9a5ca6d6674715	fig|656414.3.peg.1756	Respiratory nitrate reductase alpha chain (EC 1.7.99.4)
7a839c82814c9e723c9a5ca6d6674715	fig|670888.3.peg.2136	Respiratory nitrate reductase alpha chain (EC 1.7.99.4)
7a839c82814c9e723c9a5ca6d6674715	fig|749533.3.peg.5054	Respiratory nitrate reductase alpha chain (EC 1.7.99.4)
7a839c82814c9e723c9a5ca6d6674715	fig|749540.3.peg.707	Respiratory nitrate reductase alpha chain (EC 1.7.99.4)
7a839c82814c9e723c9a5ca6d6674715	fig|749547.3.peg.1800	Respiratory nitrate reductase alpha chain (EC 1.7.99.4)
7a839c82814c9e723c9a5ca6d6674715	fig|83333.1.peg.1454	Respiratory nitrate reductase alpha chain (EC 1.7.99.4)
3918ef25a3f8c72a8f7500b7e2a042c6	fig|3702.1.peg.25780	Glucose-6-phosphate 1-dehydrogenase (EC 1.1.1.49)
3918ef25a3f8c72a8f7500b7e2a042c6	fig|3702.7.peg.15310	Glucose-6-phosphate 1-dehydrogenase (EC 1.1.1.49)
a0b61118b58840ae71976faed35280be	fig|2433.3.peg.2482	Glycolate dehydrogenase (EC 1.1.99.14), iron-sulfur subunit GlcF
a0b61118b58840ae71976faed35280be	fig|391589.3.peg.2662	Glycolate dehydrogenase (EC 1.1.99.14), iron-sulfur subunit GlcF
3c778838db426eafafa86324b8c1dd40	fig|316385.7.peg.1300	Calcium/proton antiporter
3c778838db426eafafa86324b8c1dd40	fig|316407.3.peg.1180	Calcium/proton antiporter
3c778838db426eafafa86324b8c1dd40	fig|344610.3.peg.4263	Calcium/proton antiporter
3c778838db426eafafa86324b8c1dd40	fig|344610.7.peg.4891	Calcium/proton antiporter
3c778838db426eafafa86324b8c1dd40	fig|457401.3.peg.830	Calcium/proton antiporter
3c778838db426eafafa86324b8c1dd40	fig|511145.12.peg.1267	Calcium/proton antiporter
3c778838db426eafafa86324b8c1dd40	fig|511145.6.peg.1256	Calcium/proton antiporter
3c778838db426eafafa86324b8c1dd40	fig|536056.3.peg.2559	Calcium/proton antiporter
3c778838db426eafafa86324b8c1dd40	fig|550676.3.peg.700	Calcium/proton antiporter
3c778838db426eafafa86324b8c1dd40	fig|595496.3.peg.1148	Calcium/proton antiporter
3c778838db426eafafa86324b8c1dd40	fig|749538.3.peg.4991	Calcium/proton antiporter
3c778838db426eafafa86324b8c1dd40	fig|749544.3.peg.1696	Calcium/proton antiporter
3c778838db426eafafa86324b8c1dd40	fig|749548.3.peg.3857	Calcium/proton antiporter
3c778838db426eafafa86324b8c1dd40	fig|83333.1.peg.1205	Calcium/proton antiporter
300c74155a86c6cce681c47f5229d764	fig|216597.6.peg.3257	Sulfite reductase [NADPH] flavoprotein alpha-component (EC 1.8.1.2)
300c74155a86c6cce681c47f5229d764	fig|28901.42.peg.3769	Sulfite reductase [NADPH] flavoprotein alpha-component (EC 1.8.1.2)
300c74155a86c6cce681c47f5229d764	fig|440534.5.peg.3014	Sulfite reductase [NADPH] flavoprotein alpha-component (EC 1.8.1.2)
300c74155a86c6cce681c47f5229d764	fig|568708.3.peg.3094	Sulfite reductase [NADPH] flavoprotein alpha-component (EC 1.8.1.2)
300c74155a86c6cce681c47f5229d764	fig|588858.6.peg.3274	Sulfite reductase [NADPH] flavoprotein alpha-component (EC 1.8.1.2)
300c74155a86c6cce681c47f5229d764	fig|99287.12.peg.3109	Sulfite reductase [NADPH] flavoprotein alpha-component (EC 1.8.1.2)
300c74155a86c6cce681c47f5229d764	fig|99287.1.peg.2844	Sulfite reductase [NADPH] flavoprotein alpha-component (EC 1.8.1.2)
46cb054f679a0d65fafe09a8fcea5404	fig|316385.5.peg.2823	PTS system, glucitol/sorbitol-specific IIB component and second of two IIC components (EC 2.7.1.69)
46cb054f679a0d65fafe09a8fcea5404	fig|316385.7.peg.2889	PTS system, glucitol/sorbitol-specific IIB component and second of two IIC components (EC 2.7.1.69)
46cb054f679a0d65fafe09a8fcea5404	fig|316401.4.peg.3274	PTS system, glucitol/sorbitol-specific IIB component and second of two IIC components (EC 2.7.1.69)
46cb054f679a0d65fafe09a8fcea5404	fig|316407.3.peg.2606	PTS system, glucitol/sorbitol-specific IIB component and second of two IIC components (EC 2.7.1.69)
46cb054f679a0d65fafe09a8fcea5404	fig|457400.3.peg.682	PTS system, glucitol/sorbitol-specific IIB component and second of two IIC components (EC 2.7.1.69)
46cb054f679a0d65fafe09a8fcea5404	fig|457401.3.peg.2842	PTS system, glucitol/sorbitol-specific IIB component and second of two IIC components (EC 2.7.1.69)
46cb054f679a0d65fafe09a8fcea5404	fig|511145.12.peg.2794	PTS system, glucitol/sorbitol-specific IIB component and second of two IIC components (EC 2.7.1.69)
46cb054f679a0d65fafe09a8fcea5404	fig|511145.6.peg.2778	PTS system, glucitol/sorbitol-specific IIB component and second of two IIC components (EC 2.7.1.69)
46cb054f679a0d65fafe09a8fcea5404	fig|536056.3.peg.1033	PTS system, glucitol/sorbitol-specific IIB component and second of two IIC components (EC 2.7.1.69)
46cb054f679a0d65fafe09a8fcea5404	fig|595496.3.peg.2675	PTS system, glucitol/sorbitol-specific IIB component and second of two IIC components (EC 2.7.1.69)
46cb054f679a0d65fafe09a8fcea5404	fig|656414.3.peg.3135	PTS system, glucitol/sorbitol-specific IIB component and second of two IIC components (EC 2.7.1.69)
46cb054f679a0d65fafe09a8fcea5404	fig|749538.3.peg.599	PTS system, glucitol/sorbitol-specific IIB component and second of two IIC components (EC 2.7.1.69)
46cb054f679a0d65fafe09a8fcea5404	fig|749540.3.peg.1888	PTS system, glucitol/sorbitol-specific IIB component and second of two IIC components (EC 2.7.1.69)
46cb054f679a0d65fafe09a8fcea5404	fig|749544.3.peg.818	PTS system, glucitol/sorbitol-specific IIB component and second of two IIC components (EC 2.7.1.69)
57dbed8f2b25e21562fa68d2b351141f	fig|4932.3.peg.6603	Ammonium transporter
57dbed8f2b25e21562fa68d2b351141f	fig|559292.3.peg.5797	Ammonium transporter
d002bc182970de90bbfc20eaf52c80bb	fig|3702.1.peg.27241	similar to SP|Q03460 Glutamate synthase [NADH], chloroplast precursor (EC 1.4.1.14) (NADH- GOGAT) {Medicago sativa}; go_component: plastid [goid 0009536]; go_function: glutamate synthase (NADH) activity [goid 0016040]; go_process: glutamate biosynthesis, using glutamate synthase (NADPH) [goid 0019269]; go_process: nitrate assimilation [goid 0042128] / glutamate synthase [NADH], chloroplast, putative
d002bc182970de90bbfc20eaf52c80bb	fig|3702.7.peg.26371	Glutamate synthase [NADPH] large chain (EC 1.4.1.13)
d002bc182970de90bbfc20eaf52c80bb	fig|3702.7.peg.26372	Glutamate synthase [NADPH] large chain (EC 1.4.1.13)
d002bc182970de90bbfc20eaf52c80bb	fig|3702.7.peg.26373	Glutamate synthase [NADPH] large chain (EC 1.4.1.13)
aacd74b04c4dbb77af22176b5a672776	fig|1148.1.peg.1339	Manganese ABC transporter, ATP-binding protein SitB
aacd74b04c4dbb77af22176b5a672776	fig|1148.35.peg.1470	Manganese ABC transporter, ATP-binding protein SitB
578c0aab604f3ac23388ee0a1659d606	fig|9606.3.peg.18728	Kynureninase (EC 3.7.1.3)
21f16f854e3ba61149163dd69681337a	fig|316407.3.peg.404	Phosphatidylglycerophosphatase A (EC 3.1.3.27)
21f16f854e3ba61149163dd69681337a	fig|331111.3.peg.2989	Phosphatidylglycerophosphatase A (EC 3.1.3.27)
21f16f854e3ba61149163dd69681337a	fig|331112.3.peg.454	Phosphatidylglycerophosphatase A (EC 3.1.3.27)
21f16f854e3ba61149163dd69681337a	fig|340184.3.peg.1818	Phosphatidylglycerophosphatase A (EC 3.1.3.27)
21f16f854e3ba61149163dd69681337a	fig|340185.3.peg.3598	Phosphatidylglycerophosphatase A (EC 3.1.3.27)
21f16f854e3ba61149163dd69681337a	fig|340186.3.peg.1781	Phosphatidylglycerophosphatase A (EC 3.1.3.27)
21f16f854e3ba61149163dd69681337a	fig|344601.3.peg.3170	Phosphatidylglycerophosphatase A (EC 3.1.3.27)
21f16f854e3ba61149163dd69681337a	fig|344609.3.peg.3493	Phosphatidylglycerophosphatase A (EC 3.1.3.27)
21f16f854e3ba61149163dd69681337a	fig|344610.3.peg.3653	Phosphatidylglycerophosphatase A (EC 3.1.3.27)
21f16f854e3ba61149163dd69681337a	fig|6666666.5365.peg.5024	Phosphatidylglycerophosphatase A (EC 3.1.3.27)
21f16f854e3ba61149163dd69681337a	fig|83333.1.peg.415	Phosphatidylglycerophosphatase A (EC 3.1.3.27)
fb55e46a74c6b75082b16321a53f5076	fig|316407.3.peg.337	2-hydroxy-6-ketonona-2,4-dienedioic acid hydrolase (EC 3.7.1.-)
fb55e46a74c6b75082b16321a53f5076	fig|83333.1.peg.346	2-hydroxy-6-ketonona-2,4-dienedioic acid hydrolase (EC 3.7.1.-)
cf2de654bb4c18b3844bf12d98645565	fig|290402.34.peg.335	PTS system, glucitol/sorbitol-specific IIB component and second of two IIC components (EC 2.7.1.69)
cf2de654bb4c18b3844bf12d98645565	fig|290402.41.peg.347	PTS system, glucitol/sorbitol-specific IIB component and second of two IIC components (EC 2.7.1.69)
6c6eb03ed77aa04ee79fcbe795e7d332	fig|224308.1.peg.2683	Glutamate racemase (EC 5.1.1.3)
6c6eb03ed77aa04ee79fcbe795e7d332	fig|224308.43.peg.2794	Glutamate racemase (EC 5.1.1.3)
6c6eb03ed77aa04ee79fcbe795e7d332	fig|224308.49.peg.2687	Glutamate racemase (EC 5.1.1.3)
6c6eb03ed77aa04ee79fcbe795e7d332	fig|535024.3.peg.1826	Glutamate racemase (EC 5.1.1.3)
6c6eb03ed77aa04ee79fcbe795e7d332	fig|535025.4.peg.3914	Glutamate racemase (EC 5.1.1.3)
6c6eb03ed77aa04ee79fcbe795e7d332	fig|535026.3.peg.2854	Glutamate racemase (EC 5.1.1.3)
307e4dafc622b419abd71db1a90ceebe	fig|155864.1.peg.1656	Nudix-like NDP and NTP phosphohydrolase YmfB
307e4dafc622b419abd71db1a90ceebe	fig|155864.8.peg.1449	Nudix-like NDP and NTP phosphohydrolase YmfB
307e4dafc622b419abd71db1a90ceebe	fig|198214.1.peg.1103	Nudix-like NDP and NTP phosphohydrolase YmfB
307e4dafc622b419abd71db1a90ceebe	fig|198214.7.peg.1354	Nudix-like NDP and NTP phosphohydrolase YmfB
307e4dafc622b419abd71db1a90ceebe	fig|198215.1.peg.1110	Nudix-like NDP and NTP phosphohydrolase YmfB
307e4dafc622b419abd71db1a90ceebe	fig|198215.6.peg.1351	Nudix-like NDP and NTP phosphohydrolase YmfB
307e4dafc622b419abd71db1a90ceebe	fig|199310.1.peg.1465	Nudix-like NDP and NTP phosphohydrolase YmfB
307e4dafc622b419abd71db1a90ceebe	fig|199310.4.peg.1414	Nudix-like NDP and NTP phosphohydrolase YmfB
307e4dafc622b419abd71db1a90ceebe	fig|216592.1.peg.1559	Nudix-like NDP and NTP phosphohydrolase YmfB
307e4dafc622b419abd71db1a90ceebe	fig|216592.3.peg.1251	Nudix-like NDP and NTP phosphohydrolase YmfB
307e4dafc622b419abd71db1a90ceebe	fig|216593.1.peg.1706	Nudix-like NDP and NTP phosphohydrolase YmfB
307e4dafc622b419abd71db1a90ceebe	fig|216598.1.peg.176	Nudix-like NDP and NTP phosphohydrolase YmfB
307e4dafc622b419abd71db1a90ceebe	fig|300267.13.peg.2431	Nudix-like NDP and NTP phosphohydrolase YmfB
307e4dafc622b419abd71db1a90ceebe	fig|316385.5.peg.1212	Nudix-like NDP and NTP phosphohydrolase YmfB
307e4dafc622b419abd71db1a90ceebe	fig|316385.7.peg.1232	Nudix-like NDP and NTP phosphohydrolase YmfB
307e4dafc622b419abd71db1a90ceebe	fig|316401.4.peg.1453	Nudix-like NDP and NTP phosphohydrolase YmfB
307e4dafc622b419abd71db1a90ceebe	fig|316407.3.peg.1096	Nudix-like NDP and NTP phosphohydrolase YmfB
307e4dafc622b419abd71db1a90ceebe	fig|331111.12.peg.1589	Nudix-like NDP and NTP phosphohydrolase YmfB
307e4dafc622b419abd71db1a90ceebe	fig|331111.3.peg.3765	Nudix-like NDP and NTP phosphohydrolase YmfB
307e4dafc622b419abd71db1a90ceebe	fig|331112.3.peg.1172	Nudix-like NDP and NTP phosphohydrolase YmfB
307e4dafc622b419abd71db1a90ceebe	fig|331112.6.peg.1223	Nudix-like NDP and NTP phosphohydrolase YmfB
307e4dafc622b419abd71db1a90ceebe	fig|340184.3.peg.3420	Nudix-like NDP and NTP phosphohydrolase YmfB
307e4dafc622b419abd71db1a90ceebe	fig|340184.6.peg.3571	Nudix-like NDP and NTP phosphohydrolase YmfB
307e4dafc622b419abd71db1a90ceebe	fig|340186.3.peg.4960	Nudix-like NDP and NTP phosphohydrolase YmfB
307e4dafc622b419abd71db1a90ceebe	fig|340186.5.peg.5221	Nudix-like NDP and NTP phosphohydrolase YmfB
307e4dafc622b419abd71db1a90ceebe	fig|340197.3.peg.3199	Nudix-like NDP and NTP phosphohydrolase YmfB
307e4dafc622b419abd71db1a90ceebe	fig|340197.5.peg.3344	Nudix-like NDP and NTP phosphohydrolase YmfB
307e4dafc622b419abd71db1a90ceebe	fig|344610.3.peg.4372	Nudix-like NDP and NTP phosphohydrolase YmfB
307e4dafc622b419abd71db1a90ceebe	fig|344610.7.peg.4818	Nudix-like NDP and NTP phosphohydrolase YmfB
307e4dafc622b419abd71db1a90ceebe	fig|358708.5.peg.3344	Nudix-like NDP and NTP phosphohydrolase YmfB
307e4dafc622b419abd71db1a90ceebe	fig|362663.8.peg.1150	Nudix-like NDP and NTP phosphohydrolase YmfB
307e4dafc622b419abd71db1a90ceebe	fig|362663.9.peg.1150	Nudix-like NDP and NTP phosphohydrolase YmfB
307e4dafc622b419abd71db1a90ceebe	fig|364106.7.peg.1338	Nudix-like NDP and NTP phosphohydrolase YmfB
307e4dafc622b419abd71db1a90ceebe	fig|364106.8.peg.1337	Nudix-like NDP and NTP phosphohydrolase YmfB
307e4dafc622b419abd71db1a90ceebe	fig|373384.10.peg.1329	Nudix-like NDP and NTP phosphohydrolase YmfB
307e4dafc622b419abd71db1a90ceebe	fig|373384.11.peg.1344	Nudix-like NDP and NTP phosphohydrolase YmfB
307e4dafc622b419abd71db1a90ceebe	fig|386585.9.peg.1706	Nudix-like NDP and NTP phosphohydrolase YmfB
307e4dafc622b419abd71db1a90ceebe	fig|405955.13.peg.1138	Nudix-like NDP and NTP phosphohydrolase YmfB
307e4dafc622b419abd71db1a90ceebe	fig|405955.9.peg.959	Nudix-like NDP and NTP phosphohydrolase YmfB
307e4dafc622b419abd71db1a90ceebe	fig|409438.11.peg.1338	Nudix-like NDP and NTP phosphohydrolase YmfB
307e4dafc622b419abd71db1a90ceebe	fig|431946.3.peg.1083	Nudix-like NDP and NTP phosphohydrolase YmfB
307e4dafc622b419abd71db1a90ceebe	fig|439855.10.peg.2149	Nudix-like NDP and NTP phosphohydrolase YmfB
307e4dafc622b419abd71db1a90ceebe	fig|444447.5.peg.4440	Nudix-like NDP and NTP phosphohydrolase YmfB
307e4dafc622b419abd71db1a90ceebe	fig|444448.5.peg.4203	Nudix-like NDP and NTP phosphohydrolase YmfB
307e4dafc622b419abd71db1a90ceebe	fig|444449.5.peg.4939	Nudix-like NDP and NTP phosphohydrolase YmfB
307e4dafc622b419abd71db1a90ceebe	fig|444450.8.peg.1663	Nudix-like NDP and NTP phosphohydrolase YmfB
307e4dafc622b419abd71db1a90ceebe	fig|444451.5.peg.3589	Nudix-like NDP and NTP phosphohydrolase YmfB
307e4dafc622b419abd71db1a90ceebe	fig|444452.5.peg.2724	Nudix-like NDP and NTP phosphohydrolase YmfB
307e4dafc622b419abd71db1a90ceebe	fig|444453.5.peg.3817	Nudix-like NDP and NTP phosphohydrolase YmfB
307e4dafc622b419abd71db1a90ceebe	fig|444454.5.peg.6082	Nudix-like NDP and NTP phosphohydrolase YmfB
307e4dafc622b419abd71db1a90ceebe	fig|457401.3.peg.1056	Nudix-like NDP and NTP phosphohydrolase YmfB
307e4dafc622b419abd71db1a90ceebe	fig|469598.5.peg.804	Nudix-like NDP and NTP phosphohydrolase YmfB
307e4dafc622b419abd71db1a90ceebe	fig|478004.5.peg.3192	Nudix-like NDP and NTP phosphohydrolase YmfB
307e4dafc622b419abd71db1a90ceebe	fig|478005.5.peg.4678	Nudix-like NDP and NTP phosphohydrolase YmfB
307e4dafc622b419abd71db1a90ceebe	fig|478006.5.peg.2989	Nudix-like NDP and NTP phosphohydrolase YmfB
307e4dafc622b419abd71db1a90ceebe	fig|478007.5.peg.3134	Nudix-like NDP and NTP phosphohydrolase YmfB
307e4dafc622b419abd71db1a90ceebe	fig|478008.5.peg.2830	Nudix-like NDP and NTP phosphohydrolase YmfB
307e4dafc622b419abd71db1a90ceebe	fig|481805.3.peg.2652	Nudix-like NDP and NTP phosphohydrolase YmfB
307e4dafc622b419abd71db1a90ceebe	fig|481805.6.peg.2640	Nudix-like NDP and NTP phosphohydrolase YmfB
307e4dafc622b419abd71db1a90ceebe	fig|502346.5.peg.2687	Nudix-like NDP and NTP phosphohydrolase YmfB
307e4dafc622b419abd71db1a90ceebe	fig|511145.12.peg.1181	Nudix-like NDP and NTP phosphohydrolase YmfB
307e4dafc622b419abd71db1a90ceebe	fig|511145.6.peg.1172	Nudix-like NDP and NTP phosphohydrolase YmfB
307e4dafc622b419abd71db1a90ceebe	fig|525281.3.peg.341	Nudix-like NDP and NTP phosphohydrolase YmfB
307e4dafc622b419abd71db1a90ceebe	fig|536056.3.peg.2645	Nudix-like NDP and NTP phosphohydrolase YmfB
307e4dafc622b419abd71db1a90ceebe	fig|544404.4.peg.1526	Nudix-like NDP and NTP phosphohydrolase YmfB
307e4dafc622b419abd71db1a90ceebe	fig|550676.3.peg.631	Nudix-like NDP and NTP phosphohydrolase YmfB
307e4dafc622b419abd71db1a90ceebe	fig|550677.3.peg.2579	Nudix-like NDP and NTP phosphohydrolase YmfB
307e4dafc622b419abd71db1a90ceebe	fig|556266.3.peg.671	Nudix-like NDP and NTP phosphohydrolase YmfB
307e4dafc622b419abd71db1a90ceebe	fig|562.371.peg.502	Nudix-like NDP and NTP phosphohydrolase YmfB
307e4dafc622b419abd71db1a90ceebe	fig|562.372.peg.5330	Nudix-like NDP and NTP phosphohydrolase YmfB
307e4dafc622b419abd71db1a90ceebe	fig|562.373.peg.278	Nudix-like NDP and NTP phosphohydrolase YmfB
307e4dafc622b419abd71db1a90ceebe	fig|562.374.peg.574	Nudix-like NDP and NTP phosphohydrolase YmfB
307e4dafc622b419abd71db1a90ceebe	fig|562.376.peg.3349	Nudix-like NDP and NTP phosphohydrolase YmfB
307e4dafc622b419abd71db1a90ceebe	fig|566546.3.peg.359	Nudix-like NDP and NTP phosphohydrolase YmfB
307e4dafc622b419abd71db1a90ceebe	fig|566546.4.peg.1238	Nudix-like NDP and NTP phosphohydrolase YmfB
307e4dafc622b419abd71db1a90ceebe	fig|570506.3.peg.4623	Nudix-like NDP and NTP phosphohydrolase YmfB
307e4dafc622b419abd71db1a90ceebe	fig|573235.3.peg.1692	Nudix-like NDP and NTP phosphohydrolase YmfB
307e4dafc622b419abd71db1a90ceebe	fig|574521.7.peg.1307	Nudix-like NDP and NTP phosphohydrolase YmfB
307e4dafc622b419abd71db1a90ceebe	fig|585034.4.peg.1165	Nudix-like NDP and NTP phosphohydrolase YmfB
307e4dafc622b419abd71db1a90ceebe	fig|585034.5.peg.1162	Nudix-like NDP and NTP phosphohydrolase YmfB
307e4dafc622b419abd71db1a90ceebe	fig|585035.6.peg.1155	Nudix-like NDP and NTP phosphohydrolase YmfB
307e4dafc622b419abd71db1a90ceebe	fig|585054.5.peg.1739	Nudix-like NDP and NTP phosphohydrolase YmfB
307e4dafc622b419abd71db1a90ceebe	fig|585057.4.peg.2082	Nudix-like NDP and NTP phosphohydrolase YmfB
307e4dafc622b419abd71db1a90ceebe	fig|585057.6.peg.2081	Nudix-like NDP and NTP phosphohydrolase YmfB
307e4dafc622b419abd71db1a90ceebe	fig|585396.4.peg.1540	Nudix-like NDP and NTP phosphohydrolase YmfB
307e4dafc622b419abd71db1a90ceebe	fig|591020.3.peg.1410	Nudix-like NDP and NTP phosphohydrolase YmfB
307e4dafc622b419abd71db1a90ceebe	fig|595495.4.peg.1797	Nudix-like NDP and NTP phosphohydrolase YmfB
307e4dafc622b419abd71db1a90ceebe	fig|595496.3.peg.1081	Nudix-like NDP and NTP phosphohydrolase YmfB
307e4dafc622b419abd71db1a90ceebe	fig|621.8.peg.3244	Nudix-like NDP and NTP phosphohydrolase YmfB
307e4dafc622b419abd71db1a90ceebe	fig|637388.3.peg.4322	Nudix-like NDP and NTP phosphohydrolase YmfB
307e4dafc622b419abd71db1a90ceebe	fig|655817.3.peg.1422	Nudix-like NDP and NTP phosphohydrolase YmfB
307e4dafc622b419abd71db1a90ceebe	fig|656393.3.peg.1910	Nudix-like NDP and NTP phosphohydrolase YmfB
307e4dafc622b419abd71db1a90ceebe	fig|656408.3.peg.1216	Nudix-like NDP and NTP phosphohydrolase YmfB
307e4dafc622b419abd71db1a90ceebe	fig|656414.3.peg.1381	Nudix-like NDP and NTP phosphohydrolase YmfB
307e4dafc622b419abd71db1a90ceebe	fig|656417.3.peg.1375	Nudix-like NDP and NTP phosphohydrolase YmfB
307e4dafc622b419abd71db1a90ceebe	fig|656443.3.peg.1470	Nudix-like NDP and NTP phosphohydrolase YmfB
307e4dafc622b419abd71db1a90ceebe	fig|670888.3.peg.1762	Nudix-like NDP and NTP phosphohydrolase YmfB
307e4dafc622b419abd71db1a90ceebe	fig|670897.3.peg.2075	Nudix-like NDP and NTP phosphohydrolase YmfB
307e4dafc622b419abd71db1a90ceebe	fig|679204.3.peg.3606	Nudix-like NDP and NTP phosphohydrolase YmfB
307e4dafc622b419abd71db1a90ceebe	fig|679205.4.peg.4527	Nudix-like NDP and NTP phosphohydrolase YmfB
307e4dafc622b419abd71db1a90ceebe	fig|679206.4.peg.853	Nudix-like NDP and NTP phosphohydrolase YmfB
307e4dafc622b419abd71db1a90ceebe	fig|685038.3.peg.1103	Nudix-like NDP and NTP phosphohydrolase YmfB
307e4dafc622b419abd71db1a90ceebe	fig|701177.3.peg.1404	Nudix-like NDP and NTP phosphohydrolase YmfB
307e4dafc622b419abd71db1a90ceebe	fig|714962.3.peg.1265	Nudix-like NDP and NTP phosphohydrolase YmfB
307e4dafc622b419abd71db1a90ceebe	fig|749528.3.peg.1010	Nudix-like NDP and NTP phosphohydrolase YmfB
307e4dafc622b419abd71db1a90ceebe	fig|749531.3.peg.3719	Nudix-like NDP and NTP phosphohydrolase YmfB
307e4dafc622b419abd71db1a90ceebe	fig|749532.3.peg.3241	Nudix-like NDP and NTP phosphohydrolase YmfB
307e4dafc622b419abd71db1a90ceebe	fig|749533.3.peg.4642	Nudix-like NDP and NTP phosphohydrolase YmfB
307e4dafc622b419abd71db1a90ceebe	fig|749537.3.peg.1633	Nudix-like NDP and NTP phosphohydrolase YmfB
307e4dafc622b419abd71db1a90ceebe	fig|749538.3.peg.2750	Nudix-like NDP and NTP phosphohydrolase YmfB
307e4dafc622b419abd71db1a90ceebe	fig|749540.3.peg.1163	Nudix-like NDP and NTP phosphohydrolase YmfB
307e4dafc622b419abd71db1a90ceebe	fig|749544.3.peg.1344	Nudix-like NDP and NTP phosphohydrolase YmfB
307e4dafc622b419abd71db1a90ceebe	fig|749545.3.peg.855	Nudix-like NDP and NTP phosphohydrolase YmfB
307e4dafc622b419abd71db1a90ceebe	fig|749546.3.peg.4716	Nudix-like NDP and NTP phosphohydrolase YmfB
307e4dafc622b419abd71db1a90ceebe	fig|749548.3.peg.5014	Nudix-like NDP and NTP phosphohydrolase YmfB
307e4dafc622b419abd71db1a90ceebe	fig|749550.3.peg.2030	Nudix-like NDP and NTP phosphohydrolase YmfB
307e4dafc622b419abd71db1a90ceebe	fig|753642.3.peg.2176	Nudix-like NDP and NTP phosphohydrolase YmfB
307e4dafc622b419abd71db1a90ceebe	fig|754093.3.peg.5077	Nudix-like NDP and NTP phosphohydrolase YmfB
307e4dafc622b419abd71db1a90ceebe	fig|83333.1.peg.1119	Nudix-like NDP and NTP phosphohydrolase YmfB
307e4dafc622b419abd71db1a90ceebe	fig|83334.1.peg.1651	Nudix-like NDP and NTP phosphohydrolase YmfB
307e4dafc622b419abd71db1a90ceebe	fig|869729.3.peg.2484	Nudix-like NDP and NTP phosphohydrolase YmfB
17ed096ce7c7a41e1f5bcb85422c8623	fig|1085.1.peg.2974	Nitrogenase (iron-iron) delta chain (EC 1.18.6.1)
b4f989738550d6b545772d44b8e0b3b1	fig|10090.3.peg.26084	Nicotinamide phosphoribosyltransferase (EC 2.4.2.12)
ce98f7e599e257eb280a44ef34bae5c9	fig|224308.1.peg.2708	PTS system, mannose-specific IIC component (EC 2.7.1.69) / PTS system, fructose-specific IIC component (EC 2.7.1.69)
ce98f7e599e257eb280a44ef34bae5c9	fig|224308.43.peg.2821	PTS system, mannose-specific IIC component (EC 2.7.1.69) / PTS system, fructose-specific IIC component (EC 2.7.1.69)
ce98f7e599e257eb280a44ef34bae5c9	fig|535024.3.peg.1854	PTS system, mannose-specific IIC component (EC 2.7.1.69) / PTS system, fructose-specific IIC component (EC 2.7.1.69)
ce98f7e599e257eb280a44ef34bae5c9	fig|535025.4.peg.3941	PTS system, mannose-specific IIC component (EC 2.7.1.69) / PTS system, fructose-specific IIC component (EC 2.7.1.69)
ce98f7e599e257eb280a44ef34bae5c9	fig|535026.3.peg.2882	PTS system, mannose-specific IIC component (EC 2.7.1.69) / PTS system, fructose-specific IIC component (EC 2.7.1.69)
56b226c52290070252bd42a2bf820b69	fig|272942.6.peg.3203	Malyl-CoA lyase (EC 4.1.3.24)
dd1b930a6597075e715769d1e3bc1701	fig|9606.3.peg.21048	Pyridoxal kinase (EC 2.7.1.35)
7566525905a5785a27a8cd3373cc5f84	fig|224308.1.peg.3600	Ribose ABC transport system, high affinity permease RbsD (TC 3.A.1.2.1)
7566525905a5785a27a8cd3373cc5f84	fig|224308.43.peg.3765	Ribose ABC transport system, high affinity permease RbsD (TC 3.A.1.2.1)
7566525905a5785a27a8cd3373cc5f84	fig|224308.49.peg.3606	Ribose ABC transport system, high affinity permease RbsD (TC 3.A.1.2.1)
7566525905a5785a27a8cd3373cc5f84	fig|535024.3.peg.2806	Ribose ABC transport system, high affinity permease RbsD (TC 3.A.1.2.1)
7566525905a5785a27a8cd3373cc5f84	fig|535025.4.peg.690	Ribose ABC transport system, high affinity permease RbsD (TC 3.A.1.2.1)
7566525905a5785a27a8cd3373cc5f84	fig|535026.3.peg.3834	Ribose ABC transport system, high affinity permease RbsD (TC 3.A.1.2.1)
66ae0b1304ebaa72502caea0b4321b60	fig|316385.7.peg.2555	D-serine dehydratase (EC 4.3.1.18)
66ae0b1304ebaa72502caea0b4321b60	fig|316407.3.peg.2302	D-serine dehydratase (EC 4.3.1.18)
66ae0b1304ebaa72502caea0b4321b60	fig|344610.3.peg.313	D-serine dehydratase (EC 4.3.1.18)
66ae0b1304ebaa72502caea0b4321b60	fig|481805.3.peg.1395	D-serine dehydratase (EC 4.3.1.18)
66ae0b1304ebaa72502caea0b4321b60	fig|595495.4.peg.31	D-serine dehydratase (EC 4.3.1.18)
66ae0b1304ebaa72502caea0b4321b60	fig|749533.3.peg.1165	D-serine dehydratase (EC 4.3.1.18)
66ae0b1304ebaa72502caea0b4321b60	fig|83333.1.peg.2339	D-serine dehydratase (EC 4.3.1.18)
9ddd5d41eeabe7187d91a5e2b40ce0ce	fig|379731.4.peg.446	Glycolate dehydrogenase (EC 1.1.99.14), iron-sulfur subunit GlcF
9ddd5d41eeabe7187d91a5e2b40ce0ce	fig|379731.5.peg.446	Glycolate dehydrogenase (EC 1.1.99.14), iron-sulfur subunit GlcF
7d33545ebd455a17d869f6cff29ce550	fig|196627.13.peg.1940	Malate:quinone oxidoreductase (EC 1.1.99.16)
7d33545ebd455a17d869f6cff29ce550	fig|196627.14.peg.1902	Malate:quinone oxidoreductase (EC 1.1.99.16)
7d33545ebd455a17d869f6cff29ce550	fig|196627.4.peg.1487	Malate:quinone oxidoreductase (EC 1.1.99.16)
7d33545ebd455a17d869f6cff29ce550	fig|340322.5.peg.1884	Malate:quinone oxidoreductase (EC 1.1.99.16)
030701c93ad15f22879ea7aff09b3693	fig|272942.6.peg.595	Nitrogenase (iron-iron) reductase and maturation protein AnfH
6316bd6741973809c0ab33699fd7855e	fig|4932.3.peg.5522	Diphosphomevalonate decarboxylase (EC 4.1.1.33)
6316bd6741973809c0ab33699fd7855e	fig|559292.3.peg.4829	Diphosphomevalonate decarboxylase (EC 4.1.1.33)
c907cf8fadfa1c36047887a02c27fd25	fig|316385.5.peg.3891	Ribose ABC transport system, periplasmic ribose-binding protein RbsB (TC 3.A.1.2.1)
c907cf8fadfa1c36047887a02c27fd25	fig|316385.7.peg.3975	Ribose ABC transport system, periplasmic ribose-binding protein RbsB (TC 3.A.1.2.1)
c907cf8fadfa1c36047887a02c27fd25	fig|316407.3.peg.3372	Ribose ABC transport system, periplasmic ribose-binding protein RbsB (TC 3.A.1.2.1)
c907cf8fadfa1c36047887a02c27fd25	fig|511145.12.peg.3876	Ribose ABC transport system, periplasmic ribose-binding protein RbsB (TC 3.A.1.2.1)
c907cf8fadfa1c36047887a02c27fd25	fig|511145.6.peg.3858	Ribose ABC transport system, periplasmic ribose-binding protein RbsB (TC 3.A.1.2.1)
c907cf8fadfa1c36047887a02c27fd25	fig|536056.3.peg.4486	Ribose ABC transport system, periplasmic ribose-binding protein RbsB (TC 3.A.1.2.1)
c907cf8fadfa1c36047887a02c27fd25	fig|595496.3.peg.3758	Ribose ABC transport system, periplasmic ribose-binding protein RbsB (TC 3.A.1.2.1)
c907cf8fadfa1c36047887a02c27fd25	fig|83333.1.peg.3689	Ribose ABC transport system, periplasmic ribose-binding protein RbsB (TC 3.A.1.2.1)
2ca0c67d3fc8b1e9e095215ce43dd124	fig|267377.1.peg.945	Glyceraldehyde-3-phosphate: ferredoxin oxidoreductase (EC 1.2.7.6)
5ae057d2fc4b9be156dae464fc875ca8	fig|224308.1.peg.968	Na+/H+ antiporter NhaC
5ae057d2fc4b9be156dae464fc875ca8	fig|224308.43.peg.1010	Na+/H+ antiporter NhaC
5ae057d2fc4b9be156dae464fc875ca8	fig|224308.49.peg.994	Na+/H+ antiporter NhaC
5ae057d2fc4b9be156dae464fc875ca8	fig|535024.3.peg.4244	Na+/H+ antiporter NhaC
5ae057d2fc4b9be156dae464fc875ca8	fig|535025.4.peg.2105	Na+/H+ antiporter NhaC
5ae057d2fc4b9be156dae464fc875ca8	fig|535026.3.peg.1039	Na+/H+ antiporter NhaC
c34223158d641f5eb7e22df2c5194204	fig|340197.3.peg.836	RTX toxin transporter, ATP-binding protein # Hemolysin translocation protein HlyB
c34223158d641f5eb7e22df2c5194204	fig|340197.5.peg.874	Methionine ABC transporter ATP-binding protein
c34223158d641f5eb7e22df2c5194204	fig|364106.7.peg.4794	alpha-hemolysin translocation ATP-binding protein HlyB
c34223158d641f5eb7e22df2c5194204	fig|364106.8.peg.4793	Methionine ABC transporter ATP-binding protein
c34223158d641f5eb7e22df2c5194204	fig|749550.3.peg.458	Methionine ABC transporter ATP-binding protein
c34223158d641f5eb7e22df2c5194204	fig|869729.3.peg.4690	Methionine ABC transporter ATP-binding protein
a9f67a7b71d1debd4fa3d6551bd8d009	fig|155864.1.peg.4006	D-galactarate permease
a9f67a7b71d1debd4fa3d6551bd8d009	fig|155864.8.peg.3934	D-galactarate permease
a9f67a7b71d1debd4fa3d6551bd8d009	fig|316385.5.peg.3254	D-galactarate permease
a9f67a7b71d1debd4fa3d6551bd8d009	fig|316385.7.peg.3324	D-galactarate permease
a9f67a7b71d1debd4fa3d6551bd8d009	fig|316401.4.peg.3858	D-galactarate permease
a9f67a7b71d1debd4fa3d6551bd8d009	fig|316407.3.peg.3009	D-galactarate permease
a9f67a7b71d1debd4fa3d6551bd8d009	fig|331111.12.peg.3867	D-galactarate permease
a9f67a7b71d1debd4fa3d6551bd8d009	fig|331111.3.peg.1281	D-galactarate permease
a9f67a7b71d1debd4fa3d6551bd8d009	fig|331112.3.peg.3097	D-galactarate permease
a9f67a7b71d1debd4fa3d6551bd8d009	fig|331112.6.peg.3235	D-galactarate permease
a9f67a7b71d1debd4fa3d6551bd8d009	fig|340184.3.peg.4847	D-galactarate permease
a9f67a7b71d1debd4fa3d6551bd8d009	fig|340184.6.peg.5065	D-galactarate permease
a9f67a7b71d1debd4fa3d6551bd8d009	fig|340186.3.peg.375	D-galactarate permease
a9f67a7b71d1debd4fa3d6551bd8d009	fig|340186.5.peg.396	D-galactarate permease
a9f67a7b71d1debd4fa3d6551bd8d009	fig|344601.3.peg.2051	D-galactarate permease
a9f67a7b71d1debd4fa3d6551bd8d009	fig|344601.5.peg.2143	D-galactarate permease
a9f67a7b71d1debd4fa3d6551bd8d009	fig|344610.3.peg.2801	D-galactarate permease
a9f67a7b71d1debd4fa3d6551bd8d009	fig|344610.7.peg.3114	D-galactarate permease
a9f67a7b71d1debd4fa3d6551bd8d009	fig|358708.5.peg.3425	D-galactarate permease
a9f67a7b71d1debd4fa3d6551bd8d009	fig|386585.9.peg.4179	D-galactarate permease
a9f67a7b71d1debd4fa3d6551bd8d009	fig|409438.11.peg.3579	D-galactarate permease
a9f67a7b71d1debd4fa3d6551bd8d009	fig|413997.3.peg.3134	D-galactarate permease
a9f67a7b71d1debd4fa3d6551bd8d009	fig|444447.5.peg.1427	D-galactarate permease
a9f67a7b71d1debd4fa3d6551bd8d009	fig|444448.5.peg.1270	D-galactarate permease
a9f67a7b71d1debd4fa3d6551bd8d009	fig|444449.5.peg.2517	D-galactarate permease
a9f67a7b71d1debd4fa3d6551bd8d009	fig|444450.8.peg.4353	D-galactarate permease
a9f67a7b71d1debd4fa3d6551bd8d009	fig|444451.5.peg.925	D-galactarate permease
a9f67a7b71d1debd4fa3d6551bd8d009	fig|444452.5.peg.2298	D-galactarate permease
a9f67a7b71d1debd4fa3d6551bd8d009	fig|444453.5.peg.2693	D-galactarate permease
a9f67a7b71d1debd4fa3d6551bd8d009	fig|444454.5.peg.3058	D-galactarate permease
a9f67a7b71d1debd4fa3d6551bd8d009	fig|457400.3.peg.1082	D-galactarate permease
a9f67a7b71d1debd4fa3d6551bd8d009	fig|457401.3.peg.3063	D-galactarate permease
a9f67a7b71d1debd4fa3d6551bd8d009	fig|469008.4.peg.631	D-galactarate permease
a9f67a7b71d1debd4fa3d6551bd8d009	fig|478004.5.peg.954	D-galactarate permease
a9f67a7b71d1debd4fa3d6551bd8d009	fig|478005.5.peg.3445	D-galactarate permease
a9f67a7b71d1debd4fa3d6551bd8d009	fig|478006.5.peg.2545	D-galactarate permease
a9f67a7b71d1debd4fa3d6551bd8d009	fig|478007.5.peg.575	D-galactarate permease
a9f67a7b71d1debd4fa3d6551bd8d009	fig|478008.5.peg.1324	D-galactarate permease
a9f67a7b71d1debd4fa3d6551bd8d009	fig|481805.3.peg.609	D-galactarate permease
a9f67a7b71d1debd4fa3d6551bd8d009	fig|481805.6.peg.605	D-galactarate permease
a9f67a7b71d1debd4fa3d6551bd8d009	fig|502346.5.peg.3251	D-galactarate permease
a9f67a7b71d1debd4fa3d6551bd8d009	fig|511145.12.peg.3220	D-galactarate permease
a9f67a7b71d1debd4fa3d6551bd8d009	fig|511145.6.peg.3205	D-galactarate permease
a9f67a7b71d1debd4fa3d6551bd8d009	fig|511693.5.peg.3145	D-galactarate permease
a9f67a7b71d1debd4fa3d6551bd8d009	fig|536056.3.peg.604	D-galactarate permease
a9f67a7b71d1debd4fa3d6551bd8d009	fig|544404.4.peg.4163	D-galactarate permease
a9f67a7b71d1debd4fa3d6551bd8d009	fig|550672.3.peg.2718	D-galactarate permease
a9f67a7b71d1debd4fa3d6551bd8d009	fig|550676.3.peg.3281	D-galactarate permease
a9f67a7b71d1debd4fa3d6551bd8d009	fig|556266.3.peg.2117	D-galactarate permease
a9f67a7b71d1debd4fa3d6551bd8d009	fig|562.371.peg.2432	D-galactarate permease
a9f67a7b71d1debd4fa3d6551bd8d009	fig|562.372.peg.2627	D-galactarate permease
a9f67a7b71d1debd4fa3d6551bd8d009	fig|562.373.peg.1906	D-galactarate permease
a9f67a7b71d1debd4fa3d6551bd8d009	fig|562.374.peg.4703	D-galactarate permease
a9f67a7b71d1debd4fa3d6551bd8d009	fig|566546.3.peg.3229	D-galactarate permease
a9f67a7b71d1debd4fa3d6551bd8d009	fig|566546.4.peg.3359	D-galactarate permease
a9f67a7b71d1debd4fa3d6551bd8d009	fig|570506.3.peg.821	D-galactarate permease
a9f67a7b71d1debd4fa3d6551bd8d009	fig|573235.3.peg.4331	D-galactarate permease
a9f67a7b71d1debd4fa3d6551bd8d009	fig|585034.4.peg.3211	D-galactarate permease
a9f67a7b71d1debd4fa3d6551bd8d009	fig|585034.5.peg.3209	D-galactarate permease
a9f67a7b71d1debd4fa3d6551bd8d009	fig|585055.6.peg.3579	D-galactarate permease
a9f67a7b71d1debd4fa3d6551bd8d009	fig|585055.8.peg.3582	D-galactarate permease
a9f67a7b71d1debd4fa3d6551bd8d009	fig|585396.4.peg.4087	D-galactarate permease
a9f67a7b71d1debd4fa3d6551bd8d009	fig|595495.4.peg.794	D-galactarate permease
a9f67a7b71d1debd4fa3d6551bd8d009	fig|595496.3.peg.3104	D-galactarate permease
a9f67a7b71d1debd4fa3d6551bd8d009	fig|637388.3.peg.5216	D-galactarate permease
a9f67a7b71d1debd4fa3d6551bd8d009	fig|656408.3.peg.3547	D-galactarate permease
a9f67a7b71d1debd4fa3d6551bd8d009	fig|656414.3.peg.3597	D-galactarate permease
a9f67a7b71d1debd4fa3d6551bd8d009	fig|656419.3.peg.4107	D-galactarate permease
a9f67a7b71d1debd4fa3d6551bd8d009	fig|656443.3.peg.3898	D-galactarate permease
a9f67a7b71d1debd4fa3d6551bd8d009	fig|6666666.5522.peg.1728	D-galactarate permease
a9f67a7b71d1debd4fa3d6551bd8d009	fig|670888.3.peg.3125	D-galactarate permease
a9f67a7b71d1debd4fa3d6551bd8d009	fig|679204.3.peg.859	D-galactarate permease
a9f67a7b71d1debd4fa3d6551bd8d009	fig|679206.4.peg.1503	D-galactarate permease
a9f67a7b71d1debd4fa3d6551bd8d009	fig|679207.4.peg.2611	D-galactarate permease
a9f67a7b71d1debd4fa3d6551bd8d009	fig|701177.3.peg.3864	D-galactarate permease
a9f67a7b71d1debd4fa3d6551bd8d009	fig|749532.3.peg.1618	D-galactarate permease
a9f67a7b71d1debd4fa3d6551bd8d009	fig|749537.3.peg.504	D-galactarate permease
a9f67a7b71d1debd4fa3d6551bd8d009	fig|749540.3.peg.1410	D-galactarate permease
a9f67a7b71d1debd4fa3d6551bd8d009	fig|749544.3.peg.750	D-galactarate permease
a9f67a7b71d1debd4fa3d6551bd8d009	fig|749545.3.peg.3359	D-galactarate permease
a9f67a7b71d1debd4fa3d6551bd8d009	fig|749548.3.peg.1136	D-galactarate permease
a9f67a7b71d1debd4fa3d6551bd8d009	fig|83333.1.peg.3072	D-galactarate permease
a9f67a7b71d1debd4fa3d6551bd8d009	fig|83334.1.peg.3979	D-galactarate permease
acf777a26e95df8b501b85f33468ed58	fig|3702.1.peg.22124	Inositol-1-phosphate synthase (EC 5.5.1.4)
acf777a26e95df8b501b85f33468ed58	fig|3702.7.peg.3141	myo-inositol-1-phosphate synthase 1
8a590deeaf2ad2df16c9939b039ad036	fig|3702.1.peg.16033	Aldehyde dehydrogenase 2B4, mitochondrial precursor (EC 1.2.1.3) (ALDH2a)
8a590deeaf2ad2df16c9939b039ad036	fig|3702.7.peg.29474	Aldehyde dehydrogenase (EC 1.2.1.3)
ae69fa9032f318c5dbc87adb51b9f425	fig|3702.1.peg.6984	Isochorismate synthase (EC 5.4.4.2) @ Menaquinone-specific isochorismate synthase (EC 5.4.4.2)
ae69fa9032f318c5dbc87adb51b9f425	fig|3702.7.peg.25145	ADC synthase superfamily protein
941ca9aeac07a0f01c7a8d98afa84f8f	fig|316385.5.peg.3191	predicted tartrate:succinate antiporter
941ca9aeac07a0f01c7a8d98afa84f8f	fig|316385.7.peg.3260	Citrate Succinate antiporter (TC 2.A.47.3.2)
941ca9aeac07a0f01c7a8d98afa84f8f	fig|316401.4.peg.3784	Citrate Succinate antiporter (TC 2.A.47.3.2)
941ca9aeac07a0f01c7a8d98afa84f8f	fig|316407.3.peg.2949	Putative tartrate carrier (Tartrate transporter) (Tartrate/succinate antiporter)
941ca9aeac07a0f01c7a8d98afa84f8f	fig|358709.5.peg.1177	Citrate Succinate antiporter (TC 2.A.47.3.2)
941ca9aeac07a0f01c7a8d98afa84f8f	fig|457400.3.peg.1019	Citrate Succinate antiporter (TC 2.A.47.3.2)
941ca9aeac07a0f01c7a8d98afa84f8f	fig|457401.3.peg.3127	Citrate Succinate antiporter (TC 2.A.47.3.2)
941ca9aeac07a0f01c7a8d98afa84f8f	fig|481805.3.peg.677	Putative tartrate carrier (Tartrate transporter) (Tartrate/succinate antiporter)
941ca9aeac07a0f01c7a8d98afa84f8f	fig|481805.6.peg.675	Citrate Succinate antiporter (TC 2.A.47.3.2)
941ca9aeac07a0f01c7a8d98afa84f8f	fig|511145.12.peg.3157	Citrate Succinate antiporter (TC 2.A.47.3.2)
941ca9aeac07a0f01c7a8d98afa84f8f	fig|511145.6.peg.3142	Citrate Succinate antiporter (TC 2.A.47.3.2)
941ca9aeac07a0f01c7a8d98afa84f8f	fig|536056.3.peg.667	Citrate Succinate antiporter (TC 2.A.47.3.2)
941ca9aeac07a0f01c7a8d98afa84f8f	fig|585034.4.peg.3145	Citrate Succinate antiporter (TC 2.A.47.3.2)
941ca9aeac07a0f01c7a8d98afa84f8f	fig|585034.5.peg.3143	Citrate Succinate antiporter (TC 2.A.47.3.2)
941ca9aeac07a0f01c7a8d98afa84f8f	fig|595496.3.peg.3041	Citrate Succinate antiporter (TC 2.A.47.3.2)
941ca9aeac07a0f01c7a8d98afa84f8f	fig|656414.3.peg.3527	Citrate Succinate antiporter (TC 2.A.47.3.2)
941ca9aeac07a0f01c7a8d98afa84f8f	fig|679205.4.peg.438	Citrate Succinate antiporter (TC 2.A.47.3.2)
941ca9aeac07a0f01c7a8d98afa84f8f	fig|749533.3.peg.3569	Citrate Succinate antiporter (TC 2.A.47.3.2)
941ca9aeac07a0f01c7a8d98afa84f8f	fig|749538.3.peg.3844	Citrate Succinate antiporter (TC 2.A.47.3.2)
941ca9aeac07a0f01c7a8d98afa84f8f	fig|749548.3.peg.1085	Citrate Succinate antiporter (TC 2.A.47.3.2)
941ca9aeac07a0f01c7a8d98afa84f8f	fig|83333.1.peg.3011	Putative tartrate carrier (Tartrate transporter) (Tartrate/succinate antiporter)
7ec583216ab1bd0c74bace080956defb	fig|262724.6.peg.1542	Homoaconitase large subunit (EC 4.2.1.36)
c1c3ca6e8efcc3b63d57c196c9112a71	fig|224308.1.peg.2363	L-asparaginase I, cytoplasmic (EC 3.5.1.1)
c1c3ca6e8efcc3b63d57c196c9112a71	fig|224308.43.peg.2459	L-asparaginase (EC 3.5.1.1)
c1c3ca6e8efcc3b63d57c196c9112a71	fig|224308.49.peg.2370	L-asparaginase (EC 3.5.1.1)
c1c3ca6e8efcc3b63d57c196c9112a71	fig|535024.3.peg.1490	L-asparaginase (EC 3.5.1.1)
c1c3ca6e8efcc3b63d57c196c9112a71	fig|535025.4.peg.3577	L-asparaginase (EC 3.5.1.1)
c1c3ca6e8efcc3b63d57c196c9112a71	fig|535026.3.peg.2516	L-asparaginase (EC 3.5.1.1)
c1c3ca6e8efcc3b63d57c196c9112a71	fig|645657.3.peg.3283	L-asparaginase (EC 3.5.1.1)
7586174d65a34ebdeb7c204b5595913c	fig|224308.1.peg.1973	Lysine 2,3-aminomutase (EC 5.4.3.2)
7586174d65a34ebdeb7c204b5595913c	fig|224308.43.peg.2089	Lysine 2,3-aminomutase (EC 5.4.3.2)
7586174d65a34ebdeb7c204b5595913c	fig|224308.49.peg.2025	Lysine 2,3-aminomutase (EC 5.4.3.2)
7586174d65a34ebdeb7c204b5595913c	fig|535024.3.peg.1117	Lysine 2,3-aminomutase (EC 5.4.3.2)
7586174d65a34ebdeb7c204b5595913c	fig|535025.4.peg.3204	Lysine 2,3-aminomutase (EC 5.4.3.2)
7586174d65a34ebdeb7c204b5595913c	fig|535026.3.peg.2143	Lysine 2,3-aminomutase (EC 5.4.3.2)
cb8f93ae4aeada694a752b8673a04a68	fig|272947.1.peg.796	Citrate synthase (si) (EC 2.3.3.1)
cb8f93ae4aeada694a752b8673a04a68	fig|272947.5.peg.882	Citrate synthase (si) (EC 2.3.3.1)
cb8f93ae4aeada694a752b8673a04a68	fig|449216.3.peg.869	Citrate synthase (si) (EC 2.3.3.1)
e820b618e4694d2638135f467b6dc493	fig|243365.1.peg.3180	Phenylalanine-4-hydroxylase (EC 1.14.16.1)
e820b618e4694d2638135f467b6dc493	fig|243365.4.peg.3099	Phenylalanine-4-hydroxylase (EC 1.14.16.1)
098aaf9159b81ee45a44df28f568f53c	fig|4932.3.peg.3533	3-hydroxyanthranilate 3,4-dioxygenase (EC 1.13.11.6)
098aaf9159b81ee45a44df28f568f53c	fig|559292.3.peg.3059	3-hydroxyanthranilate 3,4-dioxygenase (EC 1.13.11.6)
56465745261a446fbb9d76b7721f9c17	fig|300852.3.peg.75	NA+/H+ antiporter (napA), putative
56465745261a446fbb9d76b7721f9c17	fig|300852.9.peg.42	trk system potassium uptake protein trkA
1c4560196177b2a7f72341bb43df947d	fig|224308.43.peg.1616	UDP-N-acetylglucosamine--N-acetylmuramyl-(pentapeptide) pyrophosphoryl-undecaprenol N-acetylglucosamine transferase (EC 2.4.1.227)
1c4560196177b2a7f72341bb43df947d	fig|224308.49.peg.1563	UDP-N-acetylglucosamine--N-acetylmuramyl-(pentapeptide) pyrophosphoryl-undecaprenol N-acetylglucosamine transferase (EC 2.4.1.227)
1c4560196177b2a7f72341bb43df947d	fig|535024.3.peg.631	UDP-N-acetylglucosamine--N-acetylmuramyl-(pentapeptide) pyrophosphoryl-undecaprenol N-acetylglucosamine transferase (EC 2.4.1.227)
1c4560196177b2a7f72341bb43df947d	fig|535025.4.peg.2719	UDP-N-acetylglucosamine--N-acetylmuramyl-(pentapeptide) pyrophosphoryl-undecaprenol N-acetylglucosamine transferase (EC 2.4.1.227)
1c4560196177b2a7f72341bb43df947d	fig|535026.3.peg.1653	UDP-N-acetylglucosamine--N-acetylmuramyl-(pentapeptide) pyrophosphoryl-undecaprenol N-acetylglucosamine transferase (EC 2.4.1.227)
411405b610696c93d210c34800289bf8	fig|316385.7.peg.2607	Cysteine synthase B (EC 2.5.1.47)
411405b610696c93d210c34800289bf8	fig|316401.4.peg.2896	Cysteine synthase B (EC 2.5.1.47)
411405b610696c93d210c34800289bf8	fig|316407.3.peg.2351	Cysteine synthase B (EC 2.5.1.47)
411405b610696c93d210c34800289bf8	fig|511145.12.peg.2516	Cysteine synthase B (EC 2.5.1.47)
411405b610696c93d210c34800289bf8	fig|511145.6.peg.2501	Cysteine synthase B (EC 2.5.1.47)
411405b610696c93d210c34800289bf8	fig|536056.3.peg.1304	Cysteine synthase B (EC 2.5.1.47)
411405b610696c93d210c34800289bf8	fig|595496.3.peg.2397	Cysteine synthase B (EC 2.5.1.47)
411405b610696c93d210c34800289bf8	fig|749538.3.peg.4803	Cysteine synthase B (EC 2.5.1.47)
411405b610696c93d210c34800289bf8	fig|83333.1.peg.2389	Cysteine synthase B (EC 2.5.1.47)
3d4d07c6d0201932400ae28cc925d661	fig|316385.5.peg.4122	Sulfur carrier protein ThiS
3d4d07c6d0201932400ae28cc925d661	fig|316385.7.peg.4208	Sulfur carrier protein ThiS
3d4d07c6d0201932400ae28cc925d661	fig|316407.3.peg.3163	Sulfur carrier protein ThiS
3d4d07c6d0201932400ae28cc925d661	fig|344610.3.peg.1255	Sulfur carrier protein ThiS
3d4d07c6d0201932400ae28cc925d661	fig|344610.7.peg.2156	Sulfur carrier protein ThiS
3d4d07c6d0201932400ae28cc925d661	fig|457400.3.peg.2091	Sulfur carrier protein ThiS
3d4d07c6d0201932400ae28cc925d661	fig|457401.3.peg.4734	Sulfur carrier protein ThiS
3d4d07c6d0201932400ae28cc925d661	fig|481805.3.peg.4340	Sulfur carrier protein ThiS
3d4d07c6d0201932400ae28cc925d661	fig|481805.6.peg.4322	Sulfur carrier protein ThiS
3d4d07c6d0201932400ae28cc925d661	fig|511145.12.peg.4105	Sulfur carrier protein ThiS
3d4d07c6d0201932400ae28cc925d661	fig|511145.6.peg.4087	Sulfur carrier protein ThiS
3d4d07c6d0201932400ae28cc925d661	fig|536056.3.peg.4255	Sulfur carrier protein ThiS
3d4d07c6d0201932400ae28cc925d661	fig|595496.3.peg.3989	Sulfur carrier protein ThiS
3d4d07c6d0201932400ae28cc925d661	fig|656414.3.peg.4530	Sulfur carrier protein ThiS
3d4d07c6d0201932400ae28cc925d661	fig|670888.3.peg.4763	Sulfur carrier protein ThiS
3d4d07c6d0201932400ae28cc925d661	fig|749537.3.peg.2886	Sulfur carrier protein ThiS
3d4d07c6d0201932400ae28cc925d661	fig|749538.3.peg.955	Sulfur carrier protein ThiS
3d4d07c6d0201932400ae28cc925d661	fig|749540.3.peg.2261	Sulfur carrier protein ThiS
3d4d07c6d0201932400ae28cc925d661	fig|749544.3.peg.689	Sulfur carrier protein ThiS
3d4d07c6d0201932400ae28cc925d661	fig|749548.3.peg.1708	Sulfur carrier protein ThiS
3d4d07c6d0201932400ae28cc925d661	fig|83333.1.peg.3904	Sulfur carrier protein ThiS
3465e4be87e4d9cd1e38fc893602e36c	fig|209261.1.peg.3679	High-affinity branched-chain amino acid transport system permease protein LivH (TC 3.A.1.4.1)
3465e4be87e4d9cd1e38fc893602e36c	fig|209261.6.peg.4289	High-affinity branched-chain amino acid transport system permease protein LivH (TC 3.A.1.4.1)
3465e4be87e4d9cd1e38fc893602e36c	fig|216597.6.peg.3928	High-affinity branched-chain amino acid transport system permease protein LivH (TC 3.A.1.4.1)
3465e4be87e4d9cd1e38fc893602e36c	fig|220341.1.peg.3747	High-affinity branched-chain amino acid transport system permease protein LivH (TC 3.A.1.4.1)
3465e4be87e4d9cd1e38fc893602e36c	fig|220341.7.peg.4339	High-affinity branched-chain amino acid transport system permease protein LivH (TC 3.A.1.4.1)
3465e4be87e4d9cd1e38fc893602e36c	fig|272994.5.peg.3686	High-affinity branched-chain amino acid transport system permease protein LivH (TC 3.A.1.4.1)
3465e4be87e4d9cd1e38fc893602e36c	fig|272994.6.peg.3580	High-affinity branched-chain amino acid transport system permease protein LivH (TC 3.A.1.4.1)
3465e4be87e4d9cd1e38fc893602e36c	fig|295319.15.peg.3620	High-affinity branched-chain amino acid transport system permease protein LivH (TC 3.A.1.4.1)
3465e4be87e4d9cd1e38fc893602e36c	fig|295319.3.peg.3191	High-affinity branched-chain amino acid transport system permease protein LivH (TC 3.A.1.4.1)
3465e4be87e4d9cd1e38fc893602e36c	fig|423368.6.peg.3872	High-affinity branched-chain amino acid transport system permease protein LivH (TC 3.A.1.4.1)
3465e4be87e4d9cd1e38fc893602e36c	fig|423368.8.peg.3853	High-affinity branched-chain amino acid transport system permease protein LivH (TC 3.A.1.4.1)
3465e4be87e4d9cd1e38fc893602e36c	fig|439843.6.peg.3735	High-affinity branched-chain amino acid transport system permease protein LivH (TC 3.A.1.4.1)
3465e4be87e4d9cd1e38fc893602e36c	fig|439843.8.peg.3751	High-affinity branched-chain amino acid transport system permease protein LivH (TC 3.A.1.4.1)
3465e4be87e4d9cd1e38fc893602e36c	fig|439846.4.peg.4054	High-affinity branched-chain amino acid transport system permease protein LivH (TC 3.A.1.4.1)
3465e4be87e4d9cd1e38fc893602e36c	fig|439851.5.peg.3897	High-affinity branched-chain amino acid transport system permease protein LivH (TC 3.A.1.4.1)
3465e4be87e4d9cd1e38fc893602e36c	fig|439851.8.peg.3873	High-affinity branched-chain amino acid transport system permease protein LivH (TC 3.A.1.4.1)
3465e4be87e4d9cd1e38fc893602e36c	fig|440534.5.peg.877	High-affinity branched-chain amino acid transport system permease protein LivH (TC 3.A.1.4.1)
3465e4be87e4d9cd1e38fc893602e36c	fig|454164.6.peg.1521	High-affinity branched-chain amino acid transport system permease protein LivH (TC 3.A.1.4.1)
3465e4be87e4d9cd1e38fc893602e36c	fig|454165.5.peg.560	High-affinity branched-chain amino acid transport system permease protein LivH (TC 3.A.1.4.1)
3465e4be87e4d9cd1e38fc893602e36c	fig|454167.5.peg.2222	High-affinity branched-chain amino acid transport system permease protein LivH (TC 3.A.1.4.1)
3465e4be87e4d9cd1e38fc893602e36c	fig|454168.5.peg.2343	High-affinity branched-chain amino acid transport system permease protein LivH (TC 3.A.1.4.1)
3465e4be87e4d9cd1e38fc893602e36c	fig|454169.6.peg.3827	High-affinity branched-chain amino acid transport system permease protein LivH (TC 3.A.1.4.1)
3465e4be87e4d9cd1e38fc893602e36c	fig|454169.8.peg.3674	High-affinity branched-chain amino acid transport system permease protein LivH (TC 3.A.1.4.1)
3465e4be87e4d9cd1e38fc893602e36c	fig|465516.5.peg.1109	High-affinity branched-chain amino acid transport system permease protein LivH (TC 3.A.1.4.1)
3465e4be87e4d9cd1e38fc893602e36c	fig|465517.10.peg.1375	High-affinity branched-chain amino acid transport system permease protein LivH (TC 3.A.1.4.1)
3465e4be87e4d9cd1e38fc893602e36c	fig|465518.5.peg.774	High-affinity branched-chain amino acid transport system permease protein LivH (TC 3.A.1.4.1)
3465e4be87e4d9cd1e38fc893602e36c	fig|496064.4.peg.4666	High-affinity branched-chain amino acid transport system permease protein LivH (TC 3.A.1.4.1)
3465e4be87e4d9cd1e38fc893602e36c	fig|497974.4.peg.3179	High-affinity branched-chain amino acid transport system permease protein LivH (TC 3.A.1.4.1)
3465e4be87e4d9cd1e38fc893602e36c	fig|550537.3.peg.3602	High-affinity branched-chain amino acid transport system permease protein LivH (TC 3.A.1.4.1)
3465e4be87e4d9cd1e38fc893602e36c	fig|550537.5.peg.3454	High-affinity branched-chain amino acid transport system permease protein LivH (TC 3.A.1.4.1)
3465e4be87e4d9cd1e38fc893602e36c	fig|550538.3.peg.4119	High-affinity branched-chain amino acid transport system permease protein LivH (TC 3.A.1.4.1)
3465e4be87e4d9cd1e38fc893602e36c	fig|550538.5.peg.4116	High-affinity branched-chain amino acid transport system permease protein LivH (TC 3.A.1.4.1)
3465e4be87e4d9cd1e38fc893602e36c	fig|554290.7.peg.3595	High-affinity branched-chain amino acid transport system permease protein LivH (TC 3.A.1.4.1)
3465e4be87e4d9cd1e38fc893602e36c	fig|554290.9.peg.3631	High-affinity branched-chain amino acid transport system permease protein LivH (TC 3.A.1.4.1)
3465e4be87e4d9cd1e38fc893602e36c	fig|568708.3.peg.3779	High-affinity branched-chain amino acid transport system permease protein LivH (TC 3.A.1.4.1)
3465e4be87e4d9cd1e38fc893602e36c	fig|588858.6.peg.3919	High-affinity branched-chain amino acid transport system permease protein LivH (TC 3.A.1.4.1)
3465e4be87e4d9cd1e38fc893602e36c	fig|99287.12.peg.3766	High-affinity branched-chain amino acid transport system permease protein LivH (TC 3.A.1.4.1)
3465e4be87e4d9cd1e38fc893602e36c	fig|99287.1.peg.3440	High-affinity branched-chain amino acid transport system permease protein LivH (TC 3.A.1.4.1)
cd1a2ca319cce2c24ca029bbb833d154	fig|4932.3.peg.2364	Hexokinase (EC 2.7.1.1)
cd1a2ca319cce2c24ca029bbb833d154	fig|559292.3.peg.1830	Hexokinase (EC 2.7.1.1)
17b50542c4464887d71f953258713dfb	fig|224308.1.peg.623	Major myo-inositol transporter IolT
17b50542c4464887d71f953258713dfb	fig|224308.43.peg.656	Major myo-inositol transporter IolT
17b50542c4464887d71f953258713dfb	fig|224308.49.peg.638	Major myo-inositol transporter IolT
17b50542c4464887d71f953258713dfb	fig|535024.3.peg.3875	Major myo-inositol transporter IolT
17b50542c4464887d71f953258713dfb	fig|535025.4.peg.1744	Major myo-inositol transporter IolT
17b50542c4464887d71f953258713dfb	fig|535026.3.peg.675	Major myo-inositol transporter IolT
2165c50ecee9a08eb47b64b4e20b4bac	fig|4932.3.peg.6340	Isopentenyl-diphosphate delta-isomerase (EC 5.3.3.2)
2165c50ecee9a08eb47b64b4e20b4bac	fig|559292.3.peg.5562	Isopentenyl-diphosphate delta-isomerase (EC 5.3.3.2)
17011cc7980aeb8ad3bd03beb1d94ed0	fig|164513.4.peg.1733	Ferredoxin--sulfite reductase, actinobacterial type (EC 1.8.7.1)
17011cc7980aeb8ad3bd03beb1d94ed0	fig|233413.5.peg.2651	Ferredoxin--sulfite reductase, actinobacterial type (EC 1.8.7.1)
17011cc7980aeb8ad3bd03beb1d94ed0	fig|336982.7.peg.2689	Ferredoxin--sulfite reductase, actinobacterial type (EC 1.8.7.1)
17011cc7980aeb8ad3bd03beb1d94ed0	fig|348776.4.peg.2625	Ferredoxin--sulfite reductase, actinobacterial type (EC 1.8.7.1)
17011cc7980aeb8ad3bd03beb1d94ed0	fig|395095.3.peg.2124	Ferredoxin--sulfite reductase, actinobacterial type (EC 1.8.7.1)
17011cc7980aeb8ad3bd03beb1d94ed0	fig|410289.15.peg.2626	Ferredoxin--sulfite reductase, actinobacterial type (EC 1.8.7.1)
17011cc7980aeb8ad3bd03beb1d94ed0	fig|419947.8.peg.2713	Ferredoxin--sulfite reductase, actinobacterial type (EC 1.8.7.1)
17011cc7980aeb8ad3bd03beb1d94ed0	fig|419947.9.peg.2313	Ferredoxin--sulfite reductase, actinobacterial type (EC 1.8.7.1)
17011cc7980aeb8ad3bd03beb1d94ed0	fig|478433.3.peg.2719	Ferredoxin--sulfite reductase, actinobacterial type (EC 1.8.7.1)
17011cc7980aeb8ad3bd03beb1d94ed0	fig|478434.4.peg.1752	Ferredoxin--sulfite reductase, actinobacterial type (EC 1.8.7.1)
17011cc7980aeb8ad3bd03beb1d94ed0	fig|478435.3.peg.2762	Ferredoxin--sulfite reductase, actinobacterial type (EC 1.8.7.1)
17011cc7980aeb8ad3bd03beb1d94ed0	fig|515615.3.peg.2201	Ferredoxin--sulfite reductase, actinobacterial type (EC 1.8.7.1)
17011cc7980aeb8ad3bd03beb1d94ed0	fig|515616.3.peg.2433	Ferredoxin--sulfite reductase, actinobacterial type (EC 1.8.7.1)
17011cc7980aeb8ad3bd03beb1d94ed0	fig|520140.3.peg.2357	Ferredoxin--sulfite reductase, actinobacterial type (EC 1.8.7.1)
17011cc7980aeb8ad3bd03beb1d94ed0	fig|520141.3.peg.2364	Ferredoxin--sulfite reductase, actinobacterial type (EC 1.8.7.1)
17011cc7980aeb8ad3bd03beb1d94ed0	fig|537209.3.peg.2502	Ferredoxin--sulfite reductase, actinobacterial type (EC 1.8.7.1)
17011cc7980aeb8ad3bd03beb1d94ed0	fig|537210.3.peg.2894	Ferredoxin--sulfite reductase, actinobacterial type (EC 1.8.7.1)
17011cc7980aeb8ad3bd03beb1d94ed0	fig|555461.3.peg.2503	Ferredoxin--sulfite reductase, actinobacterial type (EC 1.8.7.1)
17011cc7980aeb8ad3bd03beb1d94ed0	fig|561275.4.peg.2625	Ferredoxin--sulfite reductase, actinobacterial type (EC 1.8.7.1)
17011cc7980aeb8ad3bd03beb1d94ed0	fig|611302.3.peg.3277	Ferredoxin--sulfite reductase, actinobacterial type (EC 1.8.7.1)
17011cc7980aeb8ad3bd03beb1d94ed0	fig|611303.3.peg.3227	Ferredoxin--sulfite reductase, actinobacterial type (EC 1.8.7.1)
17011cc7980aeb8ad3bd03beb1d94ed0	fig|611304.3.peg.1083	Ferredoxin--sulfite reductase, actinobacterial type (EC 1.8.7.1)
17011cc7980aeb8ad3bd03beb1d94ed0	fig|663886.3.peg.2719	Ferredoxin--sulfite reductase, actinobacterial type (EC 1.8.7.1)
17011cc7980aeb8ad3bd03beb1d94ed0	fig|663887.3.peg.2715	Ferredoxin--sulfite reductase, actinobacterial type (EC 1.8.7.1)
17011cc7980aeb8ad3bd03beb1d94ed0	fig|675512.3.peg.2744	Ferredoxin--sulfite reductase, actinobacterial type (EC 1.8.7.1)
17011cc7980aeb8ad3bd03beb1d94ed0	fig|675513.3.peg.2269	Ferredoxin--sulfite reductase, actinobacterial type (EC 1.8.7.1)
17011cc7980aeb8ad3bd03beb1d94ed0	fig|675514.3.peg.2787	Ferredoxin--sulfite reductase, actinobacterial type (EC 1.8.7.1)
17011cc7980aeb8ad3bd03beb1d94ed0	fig|675515.3.peg.2932	Ferredoxin--sulfite reductase, actinobacterial type (EC 1.8.7.1)
17011cc7980aeb8ad3bd03beb1d94ed0	fig|675516.3.peg.3037	Ferredoxin--sulfite reductase, actinobacterial type (EC 1.8.7.1)
17011cc7980aeb8ad3bd03beb1d94ed0	fig|675517.3.peg.3266	Ferredoxin--sulfite reductase, actinobacterial type (EC 1.8.7.1)
17011cc7980aeb8ad3bd03beb1d94ed0	fig|675518.3.peg.3693	Ferredoxin--sulfite reductase, actinobacterial type (EC 1.8.7.1)
17011cc7980aeb8ad3bd03beb1d94ed0	fig|675519.3.peg.3267	Ferredoxin--sulfite reductase, actinobacterial type (EC 1.8.7.1)
17011cc7980aeb8ad3bd03beb1d94ed0	fig|675520.3.peg.2464	Ferredoxin--sulfite reductase, actinobacterial type (EC 1.8.7.1)
17011cc7980aeb8ad3bd03beb1d94ed0	fig|675521.3.peg.2522	Ferredoxin--sulfite reductase, actinobacterial type (EC 1.8.7.1)
17011cc7980aeb8ad3bd03beb1d94ed0	fig|675522.3.peg.2549	Ferredoxin--sulfite reductase, actinobacterial type (EC 1.8.7.1)
17011cc7980aeb8ad3bd03beb1d94ed0	fig|675523.3.peg.2869	Ferredoxin--sulfite reductase, actinobacterial type (EC 1.8.7.1)
17011cc7980aeb8ad3bd03beb1d94ed0	fig|83331.1.peg.2533	Ferredoxin--sulfite reductase, actinobacterial type (EC 1.8.7.1)
17011cc7980aeb8ad3bd03beb1d94ed0	fig|83331.22.peg.2689	Ferredoxin--sulfite reductase, actinobacterial type (EC 1.8.7.1)
17011cc7980aeb8ad3bd03beb1d94ed0	fig|83332.12.peg.2674	Ferredoxin--sulfite reductase, actinobacterial type (EC 1.8.7.1)
8bc7415b7c68067c31a53fc817d207d1	fig|196620.1.peg.2054	Pantothenate kinase type II, eukaryotic (EC 2.7.1.33)
8bc7415b7c68067c31a53fc817d207d1	fig|196620.5.peg.2129	Pantothenate kinase type II, eukaryotic (EC 2.7.1.33)
8bc7415b7c68067c31a53fc817d207d1	fig|282459.1.peg.2010	Pantothenate kinase type II, eukaryotic (EC 2.7.1.33)
8bc7415b7c68067c31a53fc817d207d1	fig|282459.5.peg.2115	Pantothenate kinase type II, eukaryotic (EC 2.7.1.33)
8bc7415b7c68067c31a53fc817d207d1	fig|367830.3.peg.581	Pantothenate kinase type II, eukaryotic (EC 2.7.1.33)
8bc7415b7c68067c31a53fc817d207d1	fig|426430.6.peg.1978	Pantothenate kinase type II, eukaryotic (EC 2.7.1.33)
8bc7415b7c68067c31a53fc817d207d1	fig|426430.8.peg.2220	Pantothenate kinase type II, eukaryotic (EC 2.7.1.33)
8bc7415b7c68067c31a53fc817d207d1	fig|451515.3.peg.2224	Pantothenate kinase type II, eukaryotic (EC 2.7.1.33)
8bc7415b7c68067c31a53fc817d207d1	fig|451516.9.peg.2213	Pantothenate kinase type II, eukaryotic (EC 2.7.1.33)
8bc7415b7c68067c31a53fc817d207d1	fig|452948.4.peg.1826	Pantothenate kinase type II, eukaryotic (EC 2.7.1.33)
8bc7415b7c68067c31a53fc817d207d1	fig|455227.3.peg.1249	Pantothenate kinase type II, eukaryotic (EC 2.7.1.33)
8bc7415b7c68067c31a53fc817d207d1	fig|546342.3.peg.8	Pantothenate kinase type II, eukaryotic (EC 2.7.1.33)
8bc7415b7c68067c31a53fc817d207d1	fig|546342.4.peg.2277	Pantothenate kinase type II, eukaryotic (EC 2.7.1.33)
8bc7415b7c68067c31a53fc817d207d1	fig|546343.3.peg.1689	Pantothenate kinase type II, eukaryotic (EC 2.7.1.33)
8bc7415b7c68067c31a53fc817d207d1	fig|548474.3.peg.1899	Pantothenate kinase type II, eukaryotic (EC 2.7.1.33)
8bc7415b7c68067c31a53fc817d207d1	fig|548475.3.peg.2582	Pantothenate kinase type II, eukaryotic (EC 2.7.1.33)
8bc7415b7c68067c31a53fc817d207d1	fig|553567.3.peg.2667	Pantothenate kinase type II, eukaryotic (EC 2.7.1.33)
8bc7415b7c68067c31a53fc817d207d1	fig|553590.4.peg.2158	Pantothenate kinase type II, eukaryotic (EC 2.7.1.33)
8bc7415b7c68067c31a53fc817d207d1	fig|553594.3.peg.1484	Pantothenate kinase type II, eukaryotic (EC 2.7.1.33)
8bc7415b7c68067c31a53fc817d207d1	fig|644279.4.peg.2272	Pantothenate kinase type II, eukaryotic (EC 2.7.1.33)
8bc7415b7c68067c31a53fc817d207d1	fig|663951.4.peg.2404	Pantothenate kinase type II, eukaryotic (EC 2.7.1.33)
8bc7415b7c68067c31a53fc817d207d1	fig|762962.3.peg.2377	Pantothenate kinase type II, eukaryotic (EC 2.7.1.33)
8bc7415b7c68067c31a53fc817d207d1	fig|862516.3.peg.125	Pantothenate kinase type II, eukaryotic (EC 2.7.1.33)
8bc7415b7c68067c31a53fc817d207d1	fig|93061.3.peg.2240	Pantothenate kinase type II, eukaryotic (EC 2.7.1.33)
8bc7415b7c68067c31a53fc817d207d1	fig|93061.5.peg.2148	Pantothenate kinase type II, eukaryotic (EC 2.7.1.33)
8bc7415b7c68067c31a53fc817d207d1	fig|93062.19.peg.2067	Pantothenate kinase type II, eukaryotic (EC 2.7.1.33)
8bc7415b7c68067c31a53fc817d207d1	fig|93062.4.peg.2128	Pantothenate kinase type II, eukaryotic (EC 2.7.1.33)
36013ebcd78237ebe4a2d0204c80604b	fig|316385.5.peg.1175	DNA polymerase III delta prime subunit (EC 2.7.7.7)
36013ebcd78237ebe4a2d0204c80604b	fig|316385.7.peg.1195	DNA polymerase III delta prime subunit (EC 2.7.7.7)
36013ebcd78237ebe4a2d0204c80604b	fig|316401.4.peg.1343	DNA polymerase III delta prime subunit (EC 2.7.7.7)
36013ebcd78237ebe4a2d0204c80604b	fig|316407.3.peg.1061	DNA polymerase III delta prime subunit (EC 2.7.7.7)
36013ebcd78237ebe4a2d0204c80604b	fig|457401.3.peg.1094	DNA polymerase III delta prime subunit (EC 2.7.7.7)
36013ebcd78237ebe4a2d0204c80604b	fig|511145.12.peg.1142	DNA polymerase III delta prime subunit (EC 2.7.7.7)
36013ebcd78237ebe4a2d0204c80604b	fig|511145.6.peg.1134	DNA polymerase III delta prime subunit (EC 2.7.7.7)
36013ebcd78237ebe4a2d0204c80604b	fig|536056.3.peg.2683	DNA polymerase III delta prime subunit (EC 2.7.7.7)
36013ebcd78237ebe4a2d0204c80604b	fig|595496.3.peg.1043	DNA polymerase III delta prime subunit (EC 2.7.7.7)
36013ebcd78237ebe4a2d0204c80604b	fig|656414.3.peg.1341	DNA polymerase III delta prime subunit (EC 2.7.7.7)
36013ebcd78237ebe4a2d0204c80604b	fig|749538.3.peg.2089	DNA polymerase III delta prime subunit (EC 2.7.7.7)
36013ebcd78237ebe4a2d0204c80604b	fig|749540.3.peg.1124	DNA polymerase III delta prime subunit (EC 2.7.7.7)
36013ebcd78237ebe4a2d0204c80604b	fig|749544.3.peg.1995	DNA polymerase III delta prime subunit (EC 2.7.7.7)
36013ebcd78237ebe4a2d0204c80604b	fig|749548.3.peg.5053	DNA polymerase III delta prime subunit (EC 2.7.7.7)
36013ebcd78237ebe4a2d0204c80604b	fig|83333.1.peg.1084	DNA polymerase III delta prime subunit (EC 2.7.7.7)
998df999495b31ba77ad4776b9729c9b	fig|224308.1.peg.3349	Sulfite reductase [NADPH] hemoprotein beta-component (EC 1.8.1.2)
998df999495b31ba77ad4776b9729c9b	fig|224308.43.peg.3506	Sulfite reductase [NADPH] hemoprotein beta-component (EC 1.8.1.2)
998df999495b31ba77ad4776b9729c9b	fig|224308.49.peg.3359	Sulfite reductase [NADPH] hemoprotein beta-component (EC 1.8.1.2)
998df999495b31ba77ad4776b9729c9b	fig|535024.3.peg.2547	Sulfite reductase [NADPH] hemoprotein beta-component (EC 1.8.1.2)
998df999495b31ba77ad4776b9729c9b	fig|535025.4.peg.430	Sulfite reductase [NADPH] hemoprotein beta-component (EC 1.8.1.2)
998df999495b31ba77ad4776b9729c9b	fig|535026.3.peg.3573	Sulfite reductase [NADPH] hemoprotein beta-component (EC 1.8.1.2)
998df999495b31ba77ad4776b9729c9b	fig|645657.3.peg.4318	Sulfite reductase [NADPH] hemoprotein beta-component (EC 1.8.1.2)
55adc234ce102909a3a204887fe6fa3b	fig|316385.5.peg.3732	Mannitol-1-phosphate 5-dehydrogenase (EC 1.1.1.17)
55adc234ce102909a3a204887fe6fa3b	fig|316385.7.peg.3818	Mannitol-1-phosphate 5-dehydrogenase (EC 1.1.1.17)
55adc234ce102909a3a204887fe6fa3b	fig|316407.3.peg.3528	Mannitol-1-phosphate 5-dehydrogenase (EC 1.1.1.17)
55adc234ce102909a3a204887fe6fa3b	fig|511145.12.peg.3718	Mannitol-1-phosphate 5-dehydrogenase (EC 1.1.1.17)
55adc234ce102909a3a204887fe6fa3b	fig|511145.6.peg.3700	Mannitol-1-phosphate 5-dehydrogenase (EC 1.1.1.17)
55adc234ce102909a3a204887fe6fa3b	fig|536056.3.peg.109	Mannitol-1-phosphate 5-dehydrogenase (EC 1.1.1.17)
55adc234ce102909a3a204887fe6fa3b	fig|595496.3.peg.3600	Mannitol-1-phosphate 5-dehydrogenase (EC 1.1.1.17)
55adc234ce102909a3a204887fe6fa3b	fig|83333.1.peg.3535	Mannitol-1-phosphate 5-dehydrogenase (EC 1.1.1.17)
8e1971e3a4b5e90c16262507a38f99e8	fig|155864.1.peg.4522	Phosphopantetheine adenylyltransferase (EC 2.7.7.3)
8e1971e3a4b5e90c16262507a38f99e8	fig|155864.8.peg.4484	Phosphopantetheine adenylyltransferase (EC 2.7.7.3)
8e1971e3a4b5e90c16262507a38f99e8	fig|198214.1.peg.3453	Phosphopantetheine adenylyltransferase (EC 2.7.7.3)
8e1971e3a4b5e90c16262507a38f99e8	fig|198214.7.peg.4336	Phosphopantetheine adenylyltransferase (EC 2.7.7.3)
8e1971e3a4b5e90c16262507a38f99e8	fig|198215.1.peg.3519	Phosphopantetheine adenylyltransferase (EC 2.7.7.3)
8e1971e3a4b5e90c16262507a38f99e8	fig|198215.6.peg.4458	Phosphopantetheine adenylyltransferase (EC 2.7.7.3)
8e1971e3a4b5e90c16262507a38f99e8	fig|216592.1.peg.917	Phosphopantetheine adenylyltransferase (EC 2.7.7.3)
8e1971e3a4b5e90c16262507a38f99e8	fig|216592.3.peg.4093	Phosphopantetheine adenylyltransferase (EC 2.7.7.3)
8e1971e3a4b5e90c16262507a38f99e8	fig|300268.10.peg.4338	Phosphopantetheine adenylyltransferase (EC 2.7.7.3)
8e1971e3a4b5e90c16262507a38f99e8	fig|300268.11.peg.4379	Phosphopantetheine adenylyltransferase (EC 2.7.7.3)
8e1971e3a4b5e90c16262507a38f99e8	fig|316385.5.peg.3768	Phosphopantetheine adenylyltransferase (EC 2.7.7.3)
8e1971e3a4b5e90c16262507a38f99e8	fig|316385.7.peg.3854	Phosphopantetheine adenylyltransferase (EC 2.7.7.3)
8e1971e3a4b5e90c16262507a38f99e8	fig|316407.3.peg.3493	Phosphopantetheine adenylyltransferase (EC 2.7.7.3)
8e1971e3a4b5e90c16262507a38f99e8	fig|331111.12.peg.4376	Phosphopantetheine adenylyltransferase (EC 2.7.7.3)
8e1971e3a4b5e90c16262507a38f99e8	fig|331111.3.peg.1777	Phosphopantetheine adenylyltransferase (EC 2.7.7.3)
8e1971e3a4b5e90c16262507a38f99e8	fig|331112.3.peg.3599	Phosphopantetheine adenylyltransferase (EC 2.7.7.3)
8e1971e3a4b5e90c16262507a38f99e8	fig|331112.6.peg.3755	Phosphopantetheine adenylyltransferase (EC 2.7.7.3)
8e1971e3a4b5e90c16262507a38f99e8	fig|340184.3.peg.2882	Phosphopantetheine adenylyltransferase (EC 2.7.7.3)
8e1971e3a4b5e90c16262507a38f99e8	fig|340184.6.peg.3017	Phosphopantetheine adenylyltransferase (EC 2.7.7.3)
8e1971e3a4b5e90c16262507a38f99e8	fig|340185.3.peg.2104	Phosphopantetheine adenylyltransferase (EC 2.7.7.3)
8e1971e3a4b5e90c16262507a38f99e8	fig|340185.4.peg.2234	Phosphopantetheine adenylyltransferase (EC 2.7.7.3)
8e1971e3a4b5e90c16262507a38f99e8	fig|340186.3.peg.3557	Phosphopantetheine adenylyltransferase (EC 2.7.7.3)
8e1971e3a4b5e90c16262507a38f99e8	fig|340186.5.peg.3711	Phosphopantetheine adenylyltransferase (EC 2.7.7.3)
8e1971e3a4b5e90c16262507a38f99e8	fig|344601.3.peg.4938	Phosphopantetheine adenylyltransferase (EC 2.7.7.3)
8e1971e3a4b5e90c16262507a38f99e8	fig|344601.5.peg.5252	Phosphopantetheine adenylyltransferase (EC 2.7.7.3)
8e1971e3a4b5e90c16262507a38f99e8	fig|344609.11.peg.4450	Phosphopantetheine adenylyltransferase (EC 2.7.7.3)
8e1971e3a4b5e90c16262507a38f99e8	fig|344609.3.peg.62	Phosphopantetheine adenylyltransferase (EC 2.7.7.3)
8e1971e3a4b5e90c16262507a38f99e8	fig|344610.3.peg.1938	Phosphopantetheine adenylyltransferase (EC 2.7.7.3)
8e1971e3a4b5e90c16262507a38f99e8	fig|344610.7.peg.2573	Phosphopantetheine adenylyltransferase (EC 2.7.7.3)
8e1971e3a4b5e90c16262507a38f99e8	fig|358709.5.peg.156	Phosphopantetheine adenylyltransferase (EC 2.7.7.3)
8e1971e3a4b5e90c16262507a38f99e8	fig|373384.10.peg.4379	Phosphopantetheine adenylyltransferase (EC 2.7.7.3)
8e1971e3a4b5e90c16262507a38f99e8	fig|373384.11.peg.4426	Phosphopantetheine adenylyltransferase (EC 2.7.7.3)
8e1971e3a4b5e90c16262507a38f99e8	fig|386585.9.peg.4725	Phosphopantetheine adenylyltransferase (EC 2.7.7.3)
8e1971e3a4b5e90c16262507a38f99e8	fig|409438.11.peg.4087	Phosphopantetheine adenylyltransferase (EC 2.7.7.3)
8e1971e3a4b5e90c16262507a38f99e8	fig|413997.3.peg.3667	Phosphopantetheine adenylyltransferase (EC 2.7.7.3)
8e1971e3a4b5e90c16262507a38f99e8	fig|439855.10.peg.4106	Phosphopantetheine adenylyltransferase (EC 2.7.7.3)
8e1971e3a4b5e90c16262507a38f99e8	fig|444447.5.peg.1983	Phosphopantetheine adenylyltransferase (EC 2.7.7.3)
8e1971e3a4b5e90c16262507a38f99e8	fig|444448.5.peg.1819	Phosphopantetheine adenylyltransferase (EC 2.7.7.3)
8e1971e3a4b5e90c16262507a38f99e8	fig|444450.8.peg.4900	Phosphopantetheine adenylyltransferase (EC 2.7.7.3)
8e1971e3a4b5e90c16262507a38f99e8	fig|444451.5.peg.166	Phosphopantetheine adenylyltransferase (EC 2.7.7.3)
8e1971e3a4b5e90c16262507a38f99e8	fig|444452.5.peg.1750	Phosphopantetheine adenylyltransferase (EC 2.7.7.3)
8e1971e3a4b5e90c16262507a38f99e8	fig|444453.5.peg.1349	Phosphopantetheine adenylyltransferase (EC 2.7.7.3)
8e1971e3a4b5e90c16262507a38f99e8	fig|444454.5.peg.3610	Phosphopantetheine adenylyltransferase (EC 2.7.7.3)
8e1971e3a4b5e90c16262507a38f99e8	fig|469008.4.peg.96	Phosphopantetheine adenylyltransferase (EC 2.7.7.3)
8e1971e3a4b5e90c16262507a38f99e8	fig|478004.5.peg.375	Phosphopantetheine adenylyltransferase (EC 2.7.7.3)
8e1971e3a4b5e90c16262507a38f99e8	fig|478005.5.peg.328	Phosphopantetheine adenylyltransferase (EC 2.7.7.3)
8e1971e3a4b5e90c16262507a38f99e8	fig|478006.5.peg.342	Phosphopantetheine adenylyltransferase (EC 2.7.7.3)
8e1971e3a4b5e90c16262507a38f99e8	fig|478007.5.peg.348	Phosphopantetheine adenylyltransferase (EC 2.7.7.3)
8e1971e3a4b5e90c16262507a38f99e8	fig|478008.5.peg.871	Phosphopantetheine adenylyltransferase (EC 2.7.7.3)
8e1971e3a4b5e90c16262507a38f99e8	fig|481805.3.peg.81	Phosphopantetheine adenylyltransferase (EC 2.7.7.3)
8e1971e3a4b5e90c16262507a38f99e8	fig|481805.6.peg.83	Phosphopantetheine adenylyltransferase (EC 2.7.7.3)
8e1971e3a4b5e90c16262507a38f99e8	fig|502346.5.peg.2118	Phosphopantetheine adenylyltransferase (EC 2.7.7.3)
8e1971e3a4b5e90c16262507a38f99e8	fig|511145.12.peg.3754	Phosphopantetheine adenylyltransferase (EC 2.7.7.3)
8e1971e3a4b5e90c16262507a38f99e8	fig|511145.6.peg.3736	Phosphopantetheine adenylyltransferase (EC 2.7.7.3)
8e1971e3a4b5e90c16262507a38f99e8	fig|511693.5.peg.3684	Phosphopantetheine adenylyltransferase (EC 2.7.7.3)
8e1971e3a4b5e90c16262507a38f99e8	fig|536056.3.peg.73	Phosphopantetheine adenylyltransferase (EC 2.7.7.3)
8e1971e3a4b5e90c16262507a38f99e8	fig|544404.4.peg.4711	Phosphopantetheine adenylyltransferase (EC 2.7.7.3)
8e1971e3a4b5e90c16262507a38f99e8	fig|550672.3.peg.3701	Phosphopantetheine adenylyltransferase (EC 2.7.7.3)
8e1971e3a4b5e90c16262507a38f99e8	fig|556266.3.peg.4212	Phosphopantetheine adenylyltransferase (EC 2.7.7.3)
8e1971e3a4b5e90c16262507a38f99e8	fig|562.371.peg.3161	Phosphopantetheine adenylyltransferase (EC 2.7.7.3)
8e1971e3a4b5e90c16262507a38f99e8	fig|562.372.peg.4044	Phosphopantetheine adenylyltransferase (EC 2.7.7.3)
8e1971e3a4b5e90c16262507a38f99e8	fig|562.373.peg.1576	Phosphopantetheine adenylyltransferase (EC 2.7.7.3)
8e1971e3a4b5e90c16262507a38f99e8	fig|562.374.peg.1168	Phosphopantetheine adenylyltransferase (EC 2.7.7.3)
8e1971e3a4b5e90c16262507a38f99e8	fig|566546.3.peg.4154	Phosphopantetheine adenylyltransferase (EC 2.7.7.3)
8e1971e3a4b5e90c16262507a38f99e8	fig|566546.4.peg.3871	Phosphopantetheine adenylyltransferase (EC 2.7.7.3)
8e1971e3a4b5e90c16262507a38f99e8	fig|570506.3.peg.623	Phosphopantetheine adenylyltransferase (EC 2.7.7.3)
8e1971e3a4b5e90c16262507a38f99e8	fig|573235.3.peg.5116	Phosphopantetheine adenylyltransferase (EC 2.7.7.3)
8e1971e3a4b5e90c16262507a38f99e8	fig|585034.4.peg.3722	Phosphopantetheine adenylyltransferase (EC 2.7.7.3)
8e1971e3a4b5e90c16262507a38f99e8	fig|585034.5.peg.3719	Phosphopantetheine adenylyltransferase (EC 2.7.7.3)
8e1971e3a4b5e90c16262507a38f99e8	fig|585055.6.peg.4131	Phosphopantetheine adenylyltransferase (EC 2.7.7.3)
8e1971e3a4b5e90c16262507a38f99e8	fig|585055.8.peg.4134	Phosphopantetheine adenylyltransferase (EC 2.7.7.3)
8e1971e3a4b5e90c16262507a38f99e8	fig|585395.4.peg.4761	Phosphopantetheine adenylyltransferase (EC 2.7.7.3)
8e1971e3a4b5e90c16262507a38f99e8	fig|585396.4.peg.4613	Phosphopantetheine adenylyltransferase (EC 2.7.7.3)
8e1971e3a4b5e90c16262507a38f99e8	fig|591020.3.peg.4299	Phosphopantetheine adenylyltransferase (EC 2.7.7.3)
8e1971e3a4b5e90c16262507a38f99e8	fig|595495.4.peg.3603	Phosphopantetheine adenylyltransferase (EC 2.7.7.3)
8e1971e3a4b5e90c16262507a38f99e8	fig|595496.3.peg.3636	Phosphopantetheine adenylyltransferase (EC 2.7.7.3)
8e1971e3a4b5e90c16262507a38f99e8	fig|622.8.peg.4693	Phosphopantetheine adenylyltransferase (EC 2.7.7.3)
8e1971e3a4b5e90c16262507a38f99e8	fig|623.7.peg.3562	Phosphopantetheine adenylyltransferase (EC 2.7.7.3)
8e1971e3a4b5e90c16262507a38f99e8	fig|637388.3.peg.1257	Phosphopantetheine adenylyltransferase (EC 2.7.7.3)
8e1971e3a4b5e90c16262507a38f99e8	fig|637912.3.peg.2273	Phosphopantetheine adenylyltransferase (EC 2.7.7.3)
8e1971e3a4b5e90c16262507a38f99e8	fig|656393.3.peg.4727	Phosphopantetheine adenylyltransferase (EC 2.7.7.3)
8e1971e3a4b5e90c16262507a38f99e8	fig|656408.3.peg.4085	Phosphopantetheine adenylyltransferase (EC 2.7.7.3)
8e1971e3a4b5e90c16262507a38f99e8	fig|656443.3.peg.4565	Phosphopantetheine adenylyltransferase (EC 2.7.7.3)
8e1971e3a4b5e90c16262507a38f99e8	fig|656444.3.peg.4950	Phosphopantetheine adenylyltransferase (EC 2.7.7.3)
8e1971e3a4b5e90c16262507a38f99e8	fig|6666666.5365.peg.3145	Phosphopantetheine adenylyltransferase (EC 2.7.7.3)
8e1971e3a4b5e90c16262507a38f99e8	fig|6666666.5522.peg.2725	Phosphopantetheine adenylyltransferase (EC 2.7.7.3)
8e1971e3a4b5e90c16262507a38f99e8	fig|679204.3.peg.4689	Phosphopantetheine adenylyltransferase (EC 2.7.7.3)
8e1971e3a4b5e90c16262507a38f99e8	fig|679205.4.peg.2381	Phosphopantetheine adenylyltransferase (EC 2.7.7.3)
8e1971e3a4b5e90c16262507a38f99e8	fig|679206.4.peg.2178	Phosphopantetheine adenylyltransferase (EC 2.7.7.3)
8e1971e3a4b5e90c16262507a38f99e8	fig|679207.4.peg.142	Phosphopantetheine adenylyltransferase (EC 2.7.7.3)
8e1971e3a4b5e90c16262507a38f99e8	fig|701177.3.peg.4397	Phosphopantetheine adenylyltransferase (EC 2.7.7.3)
8e1971e3a4b5e90c16262507a38f99e8	fig|749527.3.peg.3846	Phosphopantetheine adenylyltransferase (EC 2.7.7.3)
8e1971e3a4b5e90c16262507a38f99e8	fig|749532.3.peg.387	Phosphopantetheine adenylyltransferase (EC 2.7.7.3)
8e1971e3a4b5e90c16262507a38f99e8	fig|749533.3.peg.5302	Phosphopantetheine adenylyltransferase (EC 2.7.7.3)
8e1971e3a4b5e90c16262507a38f99e8	fig|749537.3.peg.3323	Phosphopantetheine adenylyltransferase (EC 2.7.7.3)
8e1971e3a4b5e90c16262507a38f99e8	fig|749545.3.peg.1536	Phosphopantetheine adenylyltransferase (EC 2.7.7.3)
8e1971e3a4b5e90c16262507a38f99e8	fig|749547.3.peg.304	Phosphopantetheine adenylyltransferase (EC 2.7.7.3)
8e1971e3a4b5e90c16262507a38f99e8	fig|83333.1.peg.3570	Phosphopantetheine adenylyltransferase (EC 2.7.7.3)
8e1971e3a4b5e90c16262507a38f99e8	fig|83334.1.peg.4480	Phosphopantetheine adenylyltransferase (EC 2.7.7.3)
b31318ac87074f58f0928983ebdd6ff2	fig|3702.1.peg.20830	similar to SP|O04161 Ammonium transporter 1, member 2 (LeAMT1;2) {Lycopersicon esculentum}; contains Pfam profile PF00909: Ammonium Transporter Family; go_component: membrane [goid 0016020]; go_function: ammonium transporter activity [goid 0008519]; go_process: transport [goid 0006810] / ammonium transporter, putative
b31318ac87074f58f0928983ebdd6ff2	fig|3702.7.peg.1548	Ammonium transporter
49d65fbaf902d4f5cc3df7b6920285a2	fig|216597.6.peg.2246	Propanediol utilization: dehydratase, medium subunit
49d65fbaf902d4f5cc3df7b6920285a2	fig|272994.5.peg.909	Propanediol dehydratase medium subunit (EC 4.2.1.28) @ Glycerol dehydratase medium subunit (EC 4.2.1.30)
49d65fbaf902d4f5cc3df7b6920285a2	fig|272994.6.peg.894	Propanediol dehydratase medium subunit (EC 4.2.1.28)
49d65fbaf902d4f5cc3df7b6920285a2	fig|28901.42.peg.2008	Propanediol utilization: dehydratase, medium subunit
49d65fbaf902d4f5cc3df7b6920285a2	fig|439843.6.peg.2261	Propanediol dehydratase medium subunit (EC 4.2.1.28) @ Glycerol dehydratase medium subunit (EC 4.2.1.30)
49d65fbaf902d4f5cc3df7b6920285a2	fig|439843.8.peg.2269	Propanediol dehydratase medium subunit (EC 4.2.1.28)
49d65fbaf902d4f5cc3df7b6920285a2	fig|439846.4.peg.2574	Propanediol dehydratase medium subunit (EC 4.2.1.28)
49d65fbaf902d4f5cc3df7b6920285a2	fig|440534.5.peg.1037	Propanediol dehydratase medium subunit (EC 4.2.1.28)
49d65fbaf902d4f5cc3df7b6920285a2	fig|454165.5.peg.3819	Propanediol dehydratase medium subunit (EC 4.2.1.28)
49d65fbaf902d4f5cc3df7b6920285a2	fig|454166.6.peg.2112	Propanediol dehydratase medium subunit (EC 4.2.1.28) @ Glycerol dehydratase medium subunit (EC 4.2.1.30)
49d65fbaf902d4f5cc3df7b6920285a2	fig|454166.8.peg.2113	Propanediol dehydratase medium subunit (EC 4.2.1.28)
49d65fbaf902d4f5cc3df7b6920285a2	fig|454167.5.peg.2368	Propanediol dehydratase medium subunit (EC 4.2.1.28)
49d65fbaf902d4f5cc3df7b6920285a2	fig|465518.5.peg.463	Propanediol dehydratase medium subunit (EC 4.2.1.28)
49d65fbaf902d4f5cc3df7b6920285a2	fig|568708.3.peg.2190	Propanediol dehydratase medium subunit (EC 4.2.1.28)
49d65fbaf902d4f5cc3df7b6920285a2	fig|573395.3.peg.4190	Propanediol dehydratase medium subunit (EC 4.2.1.28)
49d65fbaf902d4f5cc3df7b6920285a2	fig|588858.6.peg.2366	Propanediol dehydratase medium subunit (EC 4.2.1.28)
49d65fbaf902d4f5cc3df7b6920285a2	fig|99287.12.peg.2163	Propanediol dehydratase medium subunit (EC 4.2.1.28)
49d65fbaf902d4f5cc3df7b6920285a2	fig|99287.1.peg.1966	Propanediol dehydratase medium subunit (EC 4.2.1.28)
26c877dbd5f98d65b52210dbaef2fc5e	fig|224308.1.peg.3733	Respiratory nitrate reductase delta chain (EC 1.7.99.4)
26c877dbd5f98d65b52210dbaef2fc5e	fig|224308.43.peg.3907	Respiratory nitrate reductase delta chain (EC 1.7.99.4)
26c877dbd5f98d65b52210dbaef2fc5e	fig|224308.49.peg.3738	Respiratory nitrate reductase delta chain (EC 1.7.99.4)
26c877dbd5f98d65b52210dbaef2fc5e	fig|535024.3.peg.2949	Respiratory nitrate reductase delta chain (EC 1.7.99.4)
26c877dbd5f98d65b52210dbaef2fc5e	fig|535025.4.peg.832	Respiratory nitrate reductase delta chain (EC 1.7.99.4)
26c877dbd5f98d65b52210dbaef2fc5e	fig|535026.3.peg.3976	Respiratory nitrate reductase delta chain (EC 1.7.99.4)
4958103bc71341f7156bffe93334285c	fig|316385.7.peg.1000	3-deoxy-manno-octulosonate cytidylyltransferase (EC 2.7.7.38)
4958103bc71341f7156bffe93334285c	fig|316401.4.peg.1135	3-deoxy-manno-octulosonate cytidylyltransferase (EC 2.7.7.38)
4958103bc71341f7156bffe93334285c	fig|316407.3.peg.884	3-deoxy-manno-octulosonate cytidylyltransferase (EC 2.7.7.38)
4958103bc71341f7156bffe93334285c	fig|331112.3.peg.954	3-deoxy-manno-octulosonate cytidylyltransferase (EC 2.7.7.38)
4958103bc71341f7156bffe93334285c	fig|331112.6.peg.993	3-deoxy-manno-octulosonate cytidylyltransferase (EC 2.7.7.38)
4958103bc71341f7156bffe93334285c	fig|344610.3.peg.3970	3-deoxy-manno-octulosonate cytidylyltransferase (EC 2.7.7.38)
4958103bc71341f7156bffe93334285c	fig|344610.7.peg.741	3-deoxy-manno-octulosonate cytidylyltransferase (EC 2.7.7.38)
4958103bc71341f7156bffe93334285c	fig|358709.5.peg.3434	3-deoxy-manno-octulosonate cytidylyltransferase (EC 2.7.7.38)
4958103bc71341f7156bffe93334285c	fig|413997.3.peg.966	3-deoxy-manno-octulosonate cytidylyltransferase (EC 2.7.7.38)
4958103bc71341f7156bffe93334285c	fig|457401.3.peg.1279	3-deoxy-manno-octulosonate cytidylyltransferase (EC 2.7.7.38)
4958103bc71341f7156bffe93334285c	fig|469008.4.peg.2771	3-deoxy-manno-octulosonate cytidylyltransferase (EC 2.7.7.38)
4958103bc71341f7156bffe93334285c	fig|481805.3.peg.2878	3-deoxy-manno-octulosonate cytidylyltransferase (EC 2.7.7.38)
4958103bc71341f7156bffe93334285c	fig|481805.6.peg.2868	3-deoxy-manno-octulosonate cytidylyltransferase (EC 2.7.7.38)
4958103bc71341f7156bffe93334285c	fig|511145.12.peg.949	3-deoxy-manno-octulosonate cytidylyltransferase (EC 2.7.7.38)
4958103bc71341f7156bffe93334285c	fig|511145.6.peg.942	3-deoxy-manno-octulosonate cytidylyltransferase (EC 2.7.7.38)
4958103bc71341f7156bffe93334285c	fig|511693.5.peg.991	3-deoxy-manno-octulosonate cytidylyltransferase (EC 2.7.7.38)
4958103bc71341f7156bffe93334285c	fig|536056.3.peg.2877	3-deoxy-manno-octulosonate cytidylyltransferase (EC 2.7.7.38)
4958103bc71341f7156bffe93334285c	fig|595496.3.peg.848	3-deoxy-manno-octulosonate cytidylyltransferase (EC 2.7.7.38)
4958103bc71341f7156bffe93334285c	fig|621.8.peg.5644	3-deoxy-manno-octulosonate cytidylyltransferase (EC 2.7.7.38)
4958103bc71341f7156bffe93334285c	fig|637912.3.peg.312	3-deoxy-manno-octulosonate cytidylyltransferase (EC 2.7.7.38)
4958103bc71341f7156bffe93334285c	fig|656414.3.peg.1134	3-deoxy-manno-octulosonate cytidylyltransferase (EC 2.7.7.38)
4958103bc71341f7156bffe93334285c	fig|670888.3.peg.1534	3-deoxy-manno-octulosonate cytidylyltransferase (EC 2.7.7.38)
4958103bc71341f7156bffe93334285c	fig|749540.3.peg.108	3-deoxy-manno-octulosonate cytidylyltransferase (EC 2.7.7.38)
4958103bc71341f7156bffe93334285c	fig|749547.3.peg.1869	3-deoxy-manno-octulosonate cytidylyltransferase (EC 2.7.7.38)
4958103bc71341f7156bffe93334285c	fig|749548.3.peg.1422	3-deoxy-manno-octulosonate cytidylyltransferase (EC 2.7.7.38)
4958103bc71341f7156bffe93334285c	fig|83333.1.peg.903	3-deoxy-manno-octulosonate cytidylyltransferase (EC 2.7.7.38)
7608c49a59cb9ae665ee49e1f0b75e99	fig|264203.3.peg.405	Glucose-6-phosphate isomerase (EC 5.3.1.9)
7608c49a59cb9ae665ee49e1f0b75e99	fig|264203.5.peg.1148	Glucose-6-phosphate isomerase (EC 5.3.1.9)
7608c49a59cb9ae665ee49e1f0b75e99	fig|622759.3.peg.142	Glucose-6-phosphate isomerase (EC 5.3.1.9)
81ceddaac98c865928b8e863f3bba063	fig|316385.5.peg.1579	Respiratory nitrate reductase delta chain (EC 1.7.99.4)
81ceddaac98c865928b8e863f3bba063	fig|316385.7.peg.1619	Respiratory nitrate reductase delta chain (EC 1.7.99.4)
81ceddaac98c865928b8e863f3bba063	fig|316401.4.peg.1764	Respiratory nitrate reductase delta chain (EC 1.7.99.4)
81ceddaac98c865928b8e863f3bba063	fig|316407.3.peg.1425	Respiratory nitrate reductase delta chain (EC 1.7.99.4)
81ceddaac98c865928b8e863f3bba063	fig|331111.12.peg.1930	Respiratory nitrate reductase delta chain (EC 1.7.99.4)
81ceddaac98c865928b8e863f3bba063	fig|331111.3.peg.4090	Respiratory nitrate reductase delta chain (EC 1.7.99.4)
81ceddaac98c865928b8e863f3bba063	fig|331112.3.peg.1453	Respiratory nitrate reductase delta chain (EC 1.7.99.4)
81ceddaac98c865928b8e863f3bba063	fig|331112.6.peg.1513	Respiratory nitrate reductase delta chain (EC 1.7.99.4)
81ceddaac98c865928b8e863f3bba063	fig|340184.3.peg.99	Respiratory nitrate reductase delta chain (EC 1.7.99.4)
81ceddaac98c865928b8e863f3bba063	fig|340184.6.peg.102	Respiratory nitrate reductase delta chain (EC 1.7.99.4)
81ceddaac98c865928b8e863f3bba063	fig|340186.3.peg.176	Respiratory nitrate reductase delta chain (EC 1.7.99.4)
81ceddaac98c865928b8e863f3bba063	fig|340186.5.peg.188	Respiratory nitrate reductase delta chain (EC 1.7.99.4)
81ceddaac98c865928b8e863f3bba063	fig|358709.5.peg.957	Respiratory nitrate reductase delta chain (EC 1.7.99.4)
81ceddaac98c865928b8e863f3bba063	fig|413997.3.peg.1498	Respiratory nitrate reductase delta chain (EC 1.7.99.4)
81ceddaac98c865928b8e863f3bba063	fig|457401.3.peg.574	Respiratory nitrate reductase delta chain (EC 1.7.99.4)
81ceddaac98c865928b8e863f3bba063	fig|469008.4.peg.2237	Respiratory nitrate reductase delta chain (EC 1.7.99.4)
81ceddaac98c865928b8e863f3bba063	fig|481805.3.peg.2352	Respiratory nitrate reductase delta chain (EC 1.7.99.4)
81ceddaac98c865928b8e863f3bba063	fig|481805.6.peg.2343	Respiratory nitrate reductase delta chain (EC 1.7.99.4)
81ceddaac98c865928b8e863f3bba063	fig|511145.12.peg.1532	Respiratory nitrate reductase delta chain (EC 1.7.99.4)
81ceddaac98c865928b8e863f3bba063	fig|511145.6.peg.1518	Respiratory nitrate reductase delta chain (EC 1.7.99.4)
81ceddaac98c865928b8e863f3bba063	fig|511693.5.peg.1537	Respiratory nitrate reductase delta chain (EC 1.7.99.4)
81ceddaac98c865928b8e863f3bba063	fig|536056.3.peg.2291	Respiratory nitrate reductase delta chain (EC 1.7.99.4)
81ceddaac98c865928b8e863f3bba063	fig|562.375.peg.3794	Respiratory nitrate reductase delta chain (EC 1.7.99.4)
81ceddaac98c865928b8e863f3bba063	fig|595496.3.peg.1421	Respiratory nitrate reductase delta chain (EC 1.7.99.4)
81ceddaac98c865928b8e863f3bba063	fig|656414.3.peg.1754	Respiratory nitrate reductase delta chain (EC 1.7.99.4)
81ceddaac98c865928b8e863f3bba063	fig|670888.3.peg.2134	Respiratory nitrate reductase delta chain (EC 1.7.99.4)
81ceddaac98c865928b8e863f3bba063	fig|679205.4.peg.5542	Respiratory nitrate reductase delta chain (EC 1.7.99.4)
81ceddaac98c865928b8e863f3bba063	fig|749532.3.peg.205	Respiratory nitrate reductase delta chain (EC 1.7.99.4)
81ceddaac98c865928b8e863f3bba063	fig|749533.3.peg.5056	Respiratory nitrate reductase delta chain (EC 1.7.99.4)
81ceddaac98c865928b8e863f3bba063	fig|749537.3.peg.2511	Respiratory nitrate reductase delta chain (EC 1.7.99.4)
81ceddaac98c865928b8e863f3bba063	fig|749538.3.peg.1583	Respiratory nitrate reductase delta chain (EC 1.7.99.4)
81ceddaac98c865928b8e863f3bba063	fig|749540.3.peg.705	Respiratory nitrate reductase delta chain (EC 1.7.99.4)
81ceddaac98c865928b8e863f3bba063	fig|749544.3.peg.1892	Respiratory nitrate reductase delta chain (EC 1.7.99.4)
81ceddaac98c865928b8e863f3bba063	fig|749545.3.peg.3738	Respiratory nitrate reductase delta chain (EC 1.7.99.4)
81ceddaac98c865928b8e863f3bba063	fig|749547.3.peg.1798	Respiratory nitrate reductase delta chain (EC 1.7.99.4)
81ceddaac98c865928b8e863f3bba063	fig|83333.1.peg.1452	Respiratory nitrate reductase delta chain (EC 1.7.99.4)
4c58b24867ec0db7420aab926dff6efb	fig|272620.3.peg.3527	Glycerol dehydratase reactivation factor large subunit
4c58b24867ec0db7420aab926dff6efb	fig|272620.9.peg.3546	Glycerol dehydratase reactivation factor large subunit
4c58b24867ec0db7420aab926dff6efb	fig|484021.4.peg.4189	Glycerol dehydratase reactivation factor large subunit
68a7b6ffd74481e53076568ba8c61d1f	fig|155864.1.peg.356	High-affinity choline uptake protein BetT
68a7b6ffd74481e53076568ba8c61d1f	fig|155864.8.peg.351	High-affinity choline uptake protein BetT
68a7b6ffd74481e53076568ba8c61d1f	fig|216592.1.peg.4914	High-affinity choline uptake protein BetT
68a7b6ffd74481e53076568ba8c61d1f	fig|216592.3.peg.369	High-affinity choline uptake protein BetT
68a7b6ffd74481e53076568ba8c61d1f	fig|316385.7.peg.300	High-affinity choline uptake protein BetT
68a7b6ffd74481e53076568ba8c61d1f	fig|316407.3.peg.303	High-affinity choline uptake protein BetT
68a7b6ffd74481e53076568ba8c61d1f	fig|331111.3.peg.2882	High-affinity choline uptake protein BetT
68a7b6ffd74481e53076568ba8c61d1f	fig|331112.3.peg.348	High-affinity choline uptake protein BetT
68a7b6ffd74481e53076568ba8c61d1f	fig|331112.6.peg.367	High-affinity choline uptake protein BetT
68a7b6ffd74481e53076568ba8c61d1f	fig|340185.3.peg.2710	High-affinity choline uptake protein BetT
68a7b6ffd74481e53076568ba8c61d1f	fig|340185.4.peg.2854	High-affinity choline uptake protein BetT
68a7b6ffd74481e53076568ba8c61d1f	fig|340186.3.peg.1869	High-affinity choline uptake protein BetT
68a7b6ffd74481e53076568ba8c61d1f	fig|340186.5.peg.1934	High-affinity choline uptake protein BetT
68a7b6ffd74481e53076568ba8c61d1f	fig|344601.3.peg.1868	High-affinity choline uptake protein BetT
68a7b6ffd74481e53076568ba8c61d1f	fig|344601.5.peg.1946	High-affinity choline uptake protein BetT
68a7b6ffd74481e53076568ba8c61d1f	fig|358709.5.peg.2906	High-affinity choline uptake protein BetT
68a7b6ffd74481e53076568ba8c61d1f	fig|386585.9.peg.452	High-affinity choline uptake protein BetT
68a7b6ffd74481e53076568ba8c61d1f	fig|409438.11.peg.457	High-affinity choline uptake protein BetT
68a7b6ffd74481e53076568ba8c61d1f	fig|444447.5.peg.3208	High-affinity choline uptake protein BetT
68a7b6ffd74481e53076568ba8c61d1f	fig|444448.5.peg.3038	High-affinity choline uptake protein BetT
68a7b6ffd74481e53076568ba8c61d1f	fig|444449.5.peg.5162	High-affinity choline uptake protein BetT
68a7b6ffd74481e53076568ba8c61d1f	fig|444450.8.peg.500	High-affinity choline uptake protein BetT
68a7b6ffd74481e53076568ba8c61d1f	fig|444451.5.peg.2964	High-affinity choline uptake protein BetT
68a7b6ffd74481e53076568ba8c61d1f	fig|444452.5.peg.2203	High-affinity choline uptake protein BetT
68a7b6ffd74481e53076568ba8c61d1f	fig|444453.5.peg.4009	High-affinity choline uptake protein BetT
68a7b6ffd74481e53076568ba8c61d1f	fig|444454.5.peg.4821	High-affinity choline uptake protein BetT
68a7b6ffd74481e53076568ba8c61d1f	fig|457401.3.peg.2353	High-affinity choline uptake protein BetT
68a7b6ffd74481e53076568ba8c61d1f	fig|478004.5.peg.2636	High-affinity choline uptake protein BetT
68a7b6ffd74481e53076568ba8c61d1f	fig|478006.5.peg.2444	High-affinity choline uptake protein BetT
68a7b6ffd74481e53076568ba8c61d1f	fig|478007.5.peg.2473	High-affinity choline uptake protein BetT
68a7b6ffd74481e53076568ba8c61d1f	fig|478008.5.peg.3778	High-affinity choline uptake protein BetT
68a7b6ffd74481e53076568ba8c61d1f	fig|481805.3.peg.3553	High-affinity choline uptake protein BetT
68a7b6ffd74481e53076568ba8c61d1f	fig|502346.5.peg.910	High-affinity choline uptake protein BetT
68a7b6ffd74481e53076568ba8c61d1f	fig|511145.12.peg.321	High-affinity choline uptake protein BetT
68a7b6ffd74481e53076568ba8c61d1f	fig|511145.6.peg.319	High-affinity choline uptake protein BetT
68a7b6ffd74481e53076568ba8c61d1f	fig|511693.5.peg.290	High-affinity choline uptake protein BetT
68a7b6ffd74481e53076568ba8c61d1f	fig|536056.3.peg.3482	High-affinity choline uptake protein BetT
68a7b6ffd74481e53076568ba8c61d1f	fig|544404.4.peg.365	High-affinity choline uptake protein BetT
68a7b6ffd74481e53076568ba8c61d1f	fig|550676.3.peg.1102	High-affinity choline uptake protein BetT
68a7b6ffd74481e53076568ba8c61d1f	fig|550677.3.peg.742	High-affinity choline uptake protein BetT
68a7b6ffd74481e53076568ba8c61d1f	fig|556266.3.peg.3105	High-affinity choline uptake protein BetT
68a7b6ffd74481e53076568ba8c61d1f	fig|562.371.peg.1225	High-affinity choline uptake protein BetT
68a7b6ffd74481e53076568ba8c61d1f	fig|562.372.peg.677	High-affinity choline uptake protein BetT
68a7b6ffd74481e53076568ba8c61d1f	fig|562.373.peg.500	High-affinity choline uptake protein BetT
68a7b6ffd74481e53076568ba8c61d1f	fig|562.374.peg.1259	High-affinity choline uptake protein BetT
68a7b6ffd74481e53076568ba8c61d1f	fig|566546.3.peg.4965	High-affinity choline uptake protein BetT
68a7b6ffd74481e53076568ba8c61d1f	fig|566546.4.peg.385	High-affinity choline uptake protein BetT
68a7b6ffd74481e53076568ba8c61d1f	fig|570506.3.peg.1847	High-affinity choline uptake protein BetT
68a7b6ffd74481e53076568ba8c61d1f	fig|573235.3.peg.344	High-affinity choline uptake protein BetT
68a7b6ffd74481e53076568ba8c61d1f	fig|585034.4.peg.304	High-affinity choline uptake protein BetT
68a7b6ffd74481e53076568ba8c61d1f	fig|585034.5.peg.303	High-affinity choline uptake protein BetT
68a7b6ffd74481e53076568ba8c61d1f	fig|585055.8.peg.323	High-affinity choline uptake protein BetT
68a7b6ffd74481e53076568ba8c61d1f	fig|585395.4.peg.303	High-affinity choline uptake protein BetT
68a7b6ffd74481e53076568ba8c61d1f	fig|585396.4.peg.353	High-affinity choline uptake protein BetT
68a7b6ffd74481e53076568ba8c61d1f	fig|595495.4.peg.568	High-affinity choline uptake protein BetT
68a7b6ffd74481e53076568ba8c61d1f	fig|637388.3.peg.839	High-affinity choline uptake protein BetT
68a7b6ffd74481e53076568ba8c61d1f	fig|656379.3.peg.1182	High-affinity choline uptake protein BetT
68a7b6ffd74481e53076568ba8c61d1f	fig|656380.3.peg.939	High-affinity choline uptake protein BetT
68a7b6ffd74481e53076568ba8c61d1f	fig|656408.3.peg.239	High-affinity choline uptake protein BetT
68a7b6ffd74481e53076568ba8c61d1f	fig|656414.3.peg.501	High-affinity choline uptake protein BetT
68a7b6ffd74481e53076568ba8c61d1f	fig|656419.3.peg.499	High-affinity choline uptake protein BetT
68a7b6ffd74481e53076568ba8c61d1f	fig|656443.3.peg.518	High-affinity choline uptake protein BetT
68a7b6ffd74481e53076568ba8c61d1f	fig|656444.3.peg.770	High-affinity choline uptake protein BetT
68a7b6ffd74481e53076568ba8c61d1f	fig|6666666.5522.peg.1004	High-affinity choline uptake protein BetT
68a7b6ffd74481e53076568ba8c61d1f	fig|679205.4.peg.15	High-affinity choline uptake protein BetT
68a7b6ffd74481e53076568ba8c61d1f	fig|679206.4.peg.246	High-affinity choline uptake protein BetT
68a7b6ffd74481e53076568ba8c61d1f	fig|679207.4.peg.2166	High-affinity choline uptake protein BetT
68a7b6ffd74481e53076568ba8c61d1f	fig|701177.3.peg.406	High-affinity choline uptake protein BetT
68a7b6ffd74481e53076568ba8c61d1f	fig|749531.3.peg.1348	High-affinity choline uptake protein BetT
68a7b6ffd74481e53076568ba8c61d1f	fig|749532.3.peg.2766	High-affinity choline uptake protein BetT
68a7b6ffd74481e53076568ba8c61d1f	fig|749533.3.peg.1487	High-affinity choline uptake protein BetT
68a7b6ffd74481e53076568ba8c61d1f	fig|749538.3.peg.91	High-affinity choline uptake protein BetT
68a7b6ffd74481e53076568ba8c61d1f	fig|749544.3.peg.1295	High-affinity choline uptake protein BetT
68a7b6ffd74481e53076568ba8c61d1f	fig|749545.3.peg.1887	High-affinity choline uptake protein BetT
68a7b6ffd74481e53076568ba8c61d1f	fig|749547.3.peg.18	High-affinity choline uptake protein BetT
68a7b6ffd74481e53076568ba8c61d1f	fig|749548.3.peg.81	High-affinity choline uptake protein BetT
68a7b6ffd74481e53076568ba8c61d1f	fig|749549.3.peg.610	High-affinity choline uptake protein BetT
68a7b6ffd74481e53076568ba8c61d1f	fig|83333.1.peg.311	High-affinity choline uptake protein BetT
68a7b6ffd74481e53076568ba8c61d1f	fig|83334.1.peg.440	High-affinity choline uptake protein BetT
afca347a3905655ea3d7136083764ddf	fig|4932.3.peg.2199	5-formyltetrahydrofolate cyclo-ligase (EC 6.3.3.2)
afca347a3905655ea3d7136083764ddf	fig|559292.3.peg.1684	5-formyltetrahydrofolate cyclo-ligase (EC 6.3.3.2)
7e64acd267c62a7da9ed35041789577a	fig|4932.3.peg.3714	Ornithine decarboxylase (EC 4.1.1.17)
7e64acd267c62a7da9ed35041789577a	fig|559292.3.peg.3227	Ornithine decarboxylase (EC 4.1.1.17)
2bbf2e847e82d59300e9b6b74fad999d	fig|155864.1.peg.2488	Arginine N-succinyltransferase (EC 2.3.1.109)
2bbf2e847e82d59300e9b6b74fad999d	fig|155864.8.peg.2336	Arginine N-succinyltransferase (EC 2.3.1.109)
2bbf2e847e82d59300e9b6b74fad999d	fig|216592.1.peg.1289	Arginine N-succinyltransferase (EC 2.3.1.109)
2bbf2e847e82d59300e9b6b74fad999d	fig|216592.3.peg.1987	Arginine N-succinyltransferase (EC 2.3.1.109)
2bbf2e847e82d59300e9b6b74fad999d	fig|316385.7.peg.1907	Arginine N-succinyltransferase (EC 2.3.1.109)
2bbf2e847e82d59300e9b6b74fad999d	fig|316401.4.peg.2040	Arginine N-succinyltransferase (EC 2.3.1.109)
2bbf2e847e82d59300e9b6b74fad999d	fig|316407.3.peg.1702	Arginine N-succinyltransferase (EC 2.3.1.109)
2bbf2e847e82d59300e9b6b74fad999d	fig|331112.3.peg.1724	Arginine N-succinyltransferase (EC 2.3.1.109)
2bbf2e847e82d59300e9b6b74fad999d	fig|331112.6.peg.1790	Arginine N-succinyltransferase (EC 2.3.1.109)
2bbf2e847e82d59300e9b6b74fad999d	fig|344610.3.peg.2395	Arginine N-succinyltransferase (EC 2.3.1.109)
2bbf2e847e82d59300e9b6b74fad999d	fig|344610.7.peg.5402	Arginine N-succinyltransferase (EC 2.3.1.109)
2bbf2e847e82d59300e9b6b74fad999d	fig|358709.5.peg.3248	Arginine N-succinyltransferase (EC 2.3.1.109)
2bbf2e847e82d59300e9b6b74fad999d	fig|386585.9.peg.2567	Arginine N-succinyltransferase (EC 2.3.1.109)
2bbf2e847e82d59300e9b6b74fad999d	fig|413997.3.peg.1795	Arginine N-succinyltransferase (EC 2.3.1.109)
2bbf2e847e82d59300e9b6b74fad999d	fig|439855.10.peg.1603	Arginine N-succinyltransferase (EC 2.3.1.109)
2bbf2e847e82d59300e9b6b74fad999d	fig|444447.5.peg.4996	Arginine N-succinyltransferase (EC 2.3.1.109)
2bbf2e847e82d59300e9b6b74fad999d	fig|444448.5.peg.4992	Arginine N-succinyltransferase (EC 2.3.1.109)
2bbf2e847e82d59300e9b6b74fad999d	fig|444449.5.peg.688	Arginine N-succinyltransferase (EC 2.3.1.109)
2bbf2e847e82d59300e9b6b74fad999d	fig|444450.8.peg.2503	Arginine N-succinyltransferase (EC 2.3.1.109)
2bbf2e847e82d59300e9b6b74fad999d	fig|444451.5.peg.2579	Arginine N-succinyltransferase (EC 2.3.1.109)
2bbf2e847e82d59300e9b6b74fad999d	fig|444452.5.peg.2441	Arginine N-succinyltransferase (EC 2.3.1.109)
2bbf2e847e82d59300e9b6b74fad999d	fig|444453.5.peg.3546	Arginine N-succinyltransferase (EC 2.3.1.109)
2bbf2e847e82d59300e9b6b74fad999d	fig|444454.5.peg.1200	Arginine N-succinyltransferase (EC 2.3.1.109)
2bbf2e847e82d59300e9b6b74fad999d	fig|457401.3.peg.311	Arginine N-succinyltransferase (EC 2.3.1.109)
2bbf2e847e82d59300e9b6b74fad999d	fig|469008.4.peg.1955	Arginine N-succinyltransferase (EC 2.3.1.109)
2bbf2e847e82d59300e9b6b74fad999d	fig|478004.5.peg.3451	Arginine N-succinyltransferase (EC 2.3.1.109)
2bbf2e847e82d59300e9b6b74fad999d	fig|478005.5.peg.4305	Arginine N-succinyltransferase (EC 2.3.1.109)
2bbf2e847e82d59300e9b6b74fad999d	fig|478006.5.peg.2643	Arginine N-succinyltransferase (EC 2.3.1.109)
2bbf2e847e82d59300e9b6b74fad999d	fig|478007.5.peg.2764	Arginine N-succinyltransferase (EC 2.3.1.109)
2bbf2e847e82d59300e9b6b74fad999d	fig|481805.3.peg.2032	Arginine N-succinyltransferase (EC 2.3.1.109)
2bbf2e847e82d59300e9b6b74fad999d	fig|481805.6.peg.2028	Arginine N-succinyltransferase (EC 2.3.1.109)
2bbf2e847e82d59300e9b6b74fad999d	fig|502346.5.peg.4945	Arginine N-succinyltransferase (EC 2.3.1.109)
2bbf2e847e82d59300e9b6b74fad999d	fig|511145.12.peg.1819	Arginine N-succinyltransferase (EC 2.3.1.109)
2bbf2e847e82d59300e9b6b74fad999d	fig|511145.6.peg.1804	Arginine N-succinyltransferase (EC 2.3.1.109)
2bbf2e847e82d59300e9b6b74fad999d	fig|511693.5.peg.1823	Arginine N-succinyltransferase (EC 2.3.1.109)
2bbf2e847e82d59300e9b6b74fad999d	fig|536056.3.peg.2005	Arginine N-succinyltransferase (EC 2.3.1.109)
2bbf2e847e82d59300e9b6b74fad999d	fig|544404.4.peg.2365	Arginine N-succinyltransferase (EC 2.3.1.109)
2bbf2e847e82d59300e9b6b74fad999d	fig|562.371.peg.4603	Arginine N-succinyltransferase (EC 2.3.1.109)
2bbf2e847e82d59300e9b6b74fad999d	fig|562.372.peg.4835	Arginine N-succinyltransferase (EC 2.3.1.109)
2bbf2e847e82d59300e9b6b74fad999d	fig|562.373.peg.5505	Arginine N-succinyltransferase (EC 2.3.1.109)
2bbf2e847e82d59300e9b6b74fad999d	fig|562.374.peg.4277	Arginine N-succinyltransferase (EC 2.3.1.109)
2bbf2e847e82d59300e9b6b74fad999d	fig|570506.3.peg.3960	Arginine N-succinyltransferase (EC 2.3.1.109)
2bbf2e847e82d59300e9b6b74fad999d	fig|585057.4.peg.1369	Arginine N-succinyltransferase (EC 2.3.1.109)
2bbf2e847e82d59300e9b6b74fad999d	fig|585057.6.peg.1369	Arginine N-succinyltransferase (EC 2.3.1.109)
2bbf2e847e82d59300e9b6b74fad999d	fig|595496.3.peg.1709	Arginine N-succinyltransferase (EC 2.3.1.109)
2bbf2e847e82d59300e9b6b74fad999d	fig|637388.3.peg.4845	Arginine N-succinyltransferase (EC 2.3.1.109)
2bbf2e847e82d59300e9b6b74fad999d	fig|656414.3.peg.2069	Arginine N-succinyltransferase (EC 2.3.1.109)
2bbf2e847e82d59300e9b6b74fad999d	fig|656419.3.peg.2462	Arginine N-succinyltransferase (EC 2.3.1.109)
2bbf2e847e82d59300e9b6b74fad999d	fig|656437.3.peg.1974	Arginine N-succinyltransferase (EC 2.3.1.109)
2bbf2e847e82d59300e9b6b74fad999d	fig|670888.3.peg.251	Arginine N-succinyltransferase (EC 2.3.1.109)
2bbf2e847e82d59300e9b6b74fad999d	fig|701177.3.peg.2195	Arginine N-succinyltransferase (EC 2.3.1.109)
2bbf2e847e82d59300e9b6b74fad999d	fig|749538.3.peg.4352	Arginine N-succinyltransferase (EC 2.3.1.109)
2bbf2e847e82d59300e9b6b74fad999d	fig|749547.3.peg.2933	Arginine N-succinyltransferase (EC 2.3.1.109)
2bbf2e847e82d59300e9b6b74fad999d	fig|749548.3.peg.994	Arginine N-succinyltransferase (EC 2.3.1.109)
2bbf2e847e82d59300e9b6b74fad999d	fig|83333.1.peg.1730	Arginine N-succinyltransferase (EC 2.3.1.109)
2bbf2e847e82d59300e9b6b74fad999d	fig|83334.1.peg.2479	Arginine N-succinyltransferase (EC 2.3.1.109)
7ef6d967d72c0e3ac384724878a61652	fig|196620.1.peg.2120	Galactose-6-phosphate isomerase, LacB subunit (EC 5.3.1.26)
7ef6d967d72c0e3ac384724878a61652	fig|196620.5.peg.2192	Galactose-6-phosphate isomerase, LacB subunit (EC 5.3.1.26)
7ef6d967d72c0e3ac384724878a61652	fig|282458.1.peg.2155	Galactose-6-phosphate isomerase, LacB subunit (EC 5.3.1.26)
7ef6d967d72c0e3ac384724878a61652	fig|282458.4.peg.2295	Galactose-6-phosphate isomerase, LacB subunit (EC 5.3.1.26)
7ef6d967d72c0e3ac384724878a61652	fig|282459.1.peg.2070	Galactose-6-phosphate isomerase, LacB subunit (EC 5.3.1.26)
7ef6d967d72c0e3ac384724878a61652	fig|282459.5.peg.2177	Galactose-6-phosphate isomerase, LacB subunit (EC 5.3.1.26)
7ef6d967d72c0e3ac384724878a61652	fig|367830.3.peg.1225	Galactose-6-phosphate isomerase, LacB subunit (EC 5.3.1.26)
7ef6d967d72c0e3ac384724878a61652	fig|426430.6.peg.2039	Galactose-6-phosphate isomerase, LacB subunit (EC 5.3.1.26)
7ef6d967d72c0e3ac384724878a61652	fig|426430.8.peg.2284	Galactose-6-phosphate isomerase, LacB subunit (EC 5.3.1.26)
7ef6d967d72c0e3ac384724878a61652	fig|450394.6.peg.1405	Galactose-6-phosphate isomerase, LacB subunit (EC 5.3.1.26)
7ef6d967d72c0e3ac384724878a61652	fig|451515.3.peg.2290	Galactose-6-phosphate isomerase, LacB subunit (EC 5.3.1.26)
7ef6d967d72c0e3ac384724878a61652	fig|451516.9.peg.2279	Galactose-6-phosphate isomerase, LacB subunit (EC 5.3.1.26)
7ef6d967d72c0e3ac384724878a61652	fig|452948.4.peg.1567	Galactose-6-phosphate isomerase, LacB subunit (EC 5.3.1.26)
7ef6d967d72c0e3ac384724878a61652	fig|455227.3.peg.414	Galactose-6-phosphate isomerase, LacB subunit (EC 5.3.1.26)
7ef6d967d72c0e3ac384724878a61652	fig|546342.3.peg.2712	Galactose-6-phosphate isomerase, LacB subunit (EC 5.3.1.26)
7ef6d967d72c0e3ac384724878a61652	fig|546342.4.peg.2348	Galactose-6-phosphate isomerase, LacB subunit (EC 5.3.1.26)
7ef6d967d72c0e3ac384724878a61652	fig|546343.3.peg.539	Galactose-6-phosphate isomerase, LacB subunit (EC 5.3.1.26)
7ef6d967d72c0e3ac384724878a61652	fig|548470.3.peg.1153	Galactose-6-phosphate isomerase, LacB subunit (EC 5.3.1.26)
7ef6d967d72c0e3ac384724878a61652	fig|548473.3.peg.399	Galactose-6-phosphate isomerase, LacB subunit (EC 5.3.1.26)
7ef6d967d72c0e3ac384724878a61652	fig|548474.3.peg.49	Galactose-6-phosphate isomerase, LacB subunit (EC 5.3.1.26)
7ef6d967d72c0e3ac384724878a61652	fig|548475.3.peg.1970	Galactose-6-phosphate isomerase, LacB subunit (EC 5.3.1.26)
7ef6d967d72c0e3ac384724878a61652	fig|553567.3.peg.317	Galactose-6-phosphate isomerase, LacB subunit (EC 5.3.1.26)
7ef6d967d72c0e3ac384724878a61652	fig|553583.3.peg.161	Galactose-6-phosphate isomerase, LacB subunit (EC 5.3.1.26)
7ef6d967d72c0e3ac384724878a61652	fig|553590.4.peg.793	Galactose-6-phosphate isomerase, LacB subunit (EC 5.3.1.26)
7ef6d967d72c0e3ac384724878a61652	fig|553594.3.peg.2469	Galactose-6-phosphate isomerase, LacB subunit (EC 5.3.1.26)
7ef6d967d72c0e3ac384724878a61652	fig|585143.3.peg.339	Galactose-6-phosphate isomerase, LacB subunit (EC 5.3.1.26)
7ef6d967d72c0e3ac384724878a61652	fig|585144.3.peg.649	Galactose-6-phosphate isomerase, LacB subunit (EC 5.3.1.26)
7ef6d967d72c0e3ac384724878a61652	fig|585145.3.peg.459	Galactose-6-phosphate isomerase, LacB subunit (EC 5.3.1.26)
7ef6d967d72c0e3ac384724878a61652	fig|585146.3.peg.29	Galactose-6-phosphate isomerase, LacB subunit (EC 5.3.1.26)
7ef6d967d72c0e3ac384724878a61652	fig|585147.3.peg.1750	Galactose-6-phosphate isomerase, LacB subunit (EC 5.3.1.26)
7ef6d967d72c0e3ac384724878a61652	fig|585148.3.peg.1741	Galactose-6-phosphate isomerase, LacB subunit (EC 5.3.1.26)
7ef6d967d72c0e3ac384724878a61652	fig|585149.6.peg.2368	Galactose-6-phosphate isomerase, LacB subunit (EC 5.3.1.26)
7ef6d967d72c0e3ac384724878a61652	fig|585150.3.peg.581	Galactose-6-phosphate isomerase, LacB subunit (EC 5.3.1.26)
7ef6d967d72c0e3ac384724878a61652	fig|585151.3.peg.1909	Galactose-6-phosphate isomerase, LacB subunit (EC 5.3.1.26)
7ef6d967d72c0e3ac384724878a61652	fig|585152.3.peg.2166	Galactose-6-phosphate isomerase, LacB subunit (EC 5.3.1.26)
7ef6d967d72c0e3ac384724878a61652	fig|585153.3.peg.462	Galactose-6-phosphate isomerase, LacB subunit (EC 5.3.1.26)
7ef6d967d72c0e3ac384724878a61652	fig|585154.3.peg.2254	Galactose-6-phosphate isomerase, LacB subunit (EC 5.3.1.26)
7ef6d967d72c0e3ac384724878a61652	fig|585155.3.peg.733	Galactose-6-phosphate isomerase, LacB subunit (EC 5.3.1.26)
7ef6d967d72c0e3ac384724878a61652	fig|585156.3.peg.1746	Galactose-6-phosphate isomerase, LacB subunit (EC 5.3.1.26)
7ef6d967d72c0e3ac384724878a61652	fig|585157.3.peg.1864	Galactose-6-phosphate isomerase, LacB subunit (EC 5.3.1.26)
7ef6d967d72c0e3ac384724878a61652	fig|585158.3.peg.240	Galactose-6-phosphate isomerase, LacB subunit (EC 5.3.1.26)
7ef6d967d72c0e3ac384724878a61652	fig|585159.3.peg.2297	Galactose-6-phosphate isomerase, LacB subunit (EC 5.3.1.26)
7ef6d967d72c0e3ac384724878a61652	fig|585160.3.peg.2004	Galactose-6-phosphate isomerase, LacB subunit (EC 5.3.1.26)
7ef6d967d72c0e3ac384724878a61652	fig|585161.3.peg.2026	Galactose-6-phosphate isomerase, LacB subunit (EC 5.3.1.26)
7ef6d967d72c0e3ac384724878a61652	fig|644279.4.peg.2332	Galactose-6-phosphate isomerase, LacB subunit (EC 5.3.1.26)
7ef6d967d72c0e3ac384724878a61652	fig|663951.4.peg.2471	Galactose-6-phosphate isomerase, LacB subunit (EC 5.3.1.26)
7ef6d967d72c0e3ac384724878a61652	fig|685039.3.peg.2293	Galactose-6-phosphate isomerase, LacB subunit (EC 5.3.1.26)
7ef6d967d72c0e3ac384724878a61652	fig|762962.3.peg.2316	Galactose-6-phosphate isomerase, LacB subunit (EC 5.3.1.26)
7ef6d967d72c0e3ac384724878a61652	fig|862516.3.peg.188	Galactose-6-phosphate isomerase, LacB subunit (EC 5.3.1.26)
7ef6d967d72c0e3ac384724878a61652	fig|869816.3.peg.2205	Galactose-6-phosphate isomerase, LacB subunit (EC 5.3.1.26)
7ef6d967d72c0e3ac384724878a61652	fig|93061.3.peg.2313	Galactose-6-phosphate isomerase, LacB subunit (EC 5.3.1.26)
7ef6d967d72c0e3ac384724878a61652	fig|93061.5.peg.2213	Galactose-6-phosphate isomerase, LacB subunit (EC 5.3.1.26)
7ef6d967d72c0e3ac384724878a61652	fig|93062.19.peg.2130	Galactose-6-phosphate isomerase, LacB subunit (EC 5.3.1.26)
7ef6d967d72c0e3ac384724878a61652	fig|93062.4.peg.2213	Galactose-6-phosphate isomerase, LacB subunit (EC 5.3.1.26)
84acf7128e6971f3662dea6c30744adb	fig|656912.3.peg.1374	Ribosomal small subunit pseudouridine synthase A (EC 4.2.1.70)
84acf7128e6971f3662dea6c30744adb	fig|71421.1.peg.1189	Ribosomal small subunit pseudouridine synthase A (EC 4.2.1.70) ## SSU Psi516
84acf7128e6971f3662dea6c30744adb	fig|71421.8.peg.1295	Ribosomal small subunit pseudouridine synthase A (EC 4.2.1.70)
fe09e7789c4aee7f584c901aeefbf14c	fig|158878.14.peg.2248	Heme ABC type transporter HtsABC, permease protein HtsC
fe09e7789c4aee7f584c901aeefbf14c	fig|158878.1.peg.2175	Heme ABC type transporter HtsABC, permease protein HtsC
fe09e7789c4aee7f584c901aeefbf14c	fig|158879.11.peg.2138	Heme ABC type transporter HtsABC, permease protein HtsC
fe09e7789c4aee7f584c901aeefbf14c	fig|158879.1.peg.2050	Heme ABC type transporter HtsABC, permease protein HtsC
fe09e7789c4aee7f584c901aeefbf14c	fig|282458.1.peg.2136	Heme ABC type transporter HtsABC, permease protein HtsC
fe09e7789c4aee7f584c901aeefbf14c	fig|359786.13.peg.2326	Heme ABC type transporter HtsABC, permease protein HtsC
fe09e7789c4aee7f584c901aeefbf14c	fig|359786.3.peg.1463	Heme ABC type transporter HtsABC, permease protein HtsC
fe09e7789c4aee7f584c901aeefbf14c	fig|359787.11.peg.2295	Heme ABC type transporter HtsABC, permease protein HtsC
fe09e7789c4aee7f584c901aeefbf14c	fig|359787.3.peg.5	Heme ABC type transporter HtsABC, permease protein HtsC
fe09e7789c4aee7f584c901aeefbf14c	fig|418127.4.peg.2001	Heme ABC type transporter HtsABC, permease protein HtsC
fe09e7789c4aee7f584c901aeefbf14c	fig|418127.6.peg.2217	Heme ABC type transporter HtsABC, permease protein HtsC
fe09e7789c4aee7f584c901aeefbf14c	fig|548470.3.peg.1173	Heme ABC type transporter HtsABC, permease protein HtsC
fe09e7789c4aee7f584c901aeefbf14c	fig|553565.3.peg.2237	Heme ABC type transporter HtsABC, permease protein HtsC
fe09e7789c4aee7f584c901aeefbf14c	fig|553568.3.peg.1534	Heme ABC type transporter HtsABC, permease protein HtsC
fe09e7789c4aee7f584c901aeefbf14c	fig|553571.3.peg.1761	Heme ABC type transporter HtsABC, permease protein HtsC
fe09e7789c4aee7f584c901aeefbf14c	fig|553573.3.peg.479	Heme ABC type transporter HtsABC, permease protein HtsC
fe09e7789c4aee7f584c901aeefbf14c	fig|553574.3.peg.2531	Heme ABC type transporter HtsABC, permease protein HtsC
fe09e7789c4aee7f584c901aeefbf14c	fig|553577.3.peg.2659	Heme ABC type transporter HtsABC, permease protein HtsC
fe09e7789c4aee7f584c901aeefbf14c	fig|553580.3.peg.2771	Heme ABC type transporter HtsABC, permease protein HtsC
fe09e7789c4aee7f584c901aeefbf14c	fig|553581.3.peg.2331	Heme ABC type transporter HtsABC, permease protein HtsC
fe09e7789c4aee7f584c901aeefbf14c	fig|553583.3.peg.2319	Heme ABC type transporter HtsABC, permease protein HtsC
fe09e7789c4aee7f584c901aeefbf14c	fig|553588.3.peg.259	Heme ABC type transporter HtsABC, permease protein HtsC
fe09e7789c4aee7f584c901aeefbf14c	fig|553592.3.peg.121	Heme ABC type transporter HtsABC, permease protein HtsC
fe09e7789c4aee7f584c901aeefbf14c	fig|553596.3.peg.236	Heme ABC type transporter HtsABC, permease protein HtsC
fe09e7789c4aee7f584c901aeefbf14c	fig|553601.3.peg.2683	Heme ABC type transporter HtsABC, permease protein HtsC
fe09e7789c4aee7f584c901aeefbf14c	fig|585143.3.peg.320	Heme ABC type transporter HtsABC, permease protein HtsC
fe09e7789c4aee7f584c901aeefbf14c	fig|585144.3.peg.630	Heme ABC type transporter HtsABC, permease protein HtsC
fe09e7789c4aee7f584c901aeefbf14c	fig|585145.3.peg.440	Heme ABC type transporter HtsABC, permease protein HtsC
fe09e7789c4aee7f584c901aeefbf14c	fig|585146.3.peg.10	Heme ABC type transporter HtsABC, permease protein HtsC
fe09e7789c4aee7f584c901aeefbf14c	fig|585147.3.peg.1731	Heme ABC type transporter HtsABC, permease protein HtsC
fe09e7789c4aee7f584c901aeefbf14c	fig|585148.3.peg.1722	Heme ABC type transporter HtsABC, permease protein HtsC
fe09e7789c4aee7f584c901aeefbf14c	fig|585149.6.peg.2349	Heme ABC type transporter HtsABC, permease protein HtsC
fe09e7789c4aee7f584c901aeefbf14c	fig|585150.3.peg.13	Heme ABC type transporter HtsABC, permease protein HtsC
fe09e7789c4aee7f584c901aeefbf14c	fig|585153.3.peg.443	Heme ABC type transporter HtsABC, permease protein HtsC
fe09e7789c4aee7f584c901aeefbf14c	fig|585154.3.peg.2234	Heme ABC type transporter HtsABC, permease protein HtsC
fe09e7789c4aee7f584c901aeefbf14c	fig|585157.3.peg.1845	Heme ABC type transporter HtsABC, permease protein HtsC
fe09e7789c4aee7f584c901aeefbf14c	fig|585158.3.peg.221	Heme ABC type transporter HtsABC, permease protein HtsC
fe09e7789c4aee7f584c901aeefbf14c	fig|585159.3.peg.2278	Heme ABC type transporter HtsABC, permease protein HtsC
fe09e7789c4aee7f584c901aeefbf14c	fig|585160.3.peg.1662	Heme ABC type transporter HtsABC, permease protein HtsC
fe09e7789c4aee7f584c901aeefbf14c	fig|585161.3.peg.2007	Heme ABC type transporter HtsABC, permease protein HtsC
fe09e7789c4aee7f584c901aeefbf14c	fig|585891.3.peg.2244	Heme ABC type transporter HtsABC, permease protein HtsC
fe09e7789c4aee7f584c901aeefbf14c	fig|680649.3.peg.1185	Heme ABC type transporter HtsABC, permease protein HtsC
fe09e7789c4aee7f584c901aeefbf14c	fig|681288.4.peg.2192	Heme ABC type transporter HtsABC, permease protein HtsC
fe09e7789c4aee7f584c901aeefbf14c	fig|703339.3.peg.2154	Heme ABC type transporter HtsABC, permease protein HtsC
d0ea3523508bac76fc5039e04dcb7ae3	fig|224308.1.peg.3086	Menaquinone-specific isochorismate synthase (EC 5.4.4.2)
39c69eb6638c09727606ecbe7b8676ee	fig|242507.1.peg.4613	Saccharopine dehydrogenase [NADP , L-glutamate-forming] (EC 1.5.1.10)
8cc05077228007da2d3a7bf7635361b4	fig|216592.1.peg.4205	Methionine ABC transporter permease protein
8cc05077228007da2d3a7bf7635361b4	fig|216592.3.peg.210	Methionine ABC transporter permease protein
8cc05077228007da2d3a7bf7635361b4	fig|216599.1.peg.995	Methionine ABC transporter permease protein
8cc05077228007da2d3a7bf7635361b4	fig|300268.10.peg.397	Methionine ABC transporter permease protein
8cc05077228007da2d3a7bf7635361b4	fig|300268.11.peg.401	Methionine ABC transporter permease protein
8cc05077228007da2d3a7bf7635361b4	fig|300269.11.peg.236	Methionine ABC transporter permease protein
8cc05077228007da2d3a7bf7635361b4	fig|300269.12.peg.248	Methionine ABC transporter permease protein
8cc05077228007da2d3a7bf7635361b4	fig|316385.5.peg.180	Methionine ABC transporter permease protein
8cc05077228007da2d3a7bf7635361b4	fig|316385.7.peg.183	Methionine ABC transporter permease protein
8cc05077228007da2d3a7bf7635361b4	fig|316407.3.peg.195	Methionine ABC transporter permease protein
8cc05077228007da2d3a7bf7635361b4	fig|331111.12.peg.537	Methionine ABC transporter permease protein
8cc05077228007da2d3a7bf7635361b4	fig|331111.3.peg.2773	Methionine ABC transporter permease protein
8cc05077228007da2d3a7bf7635361b4	fig|331112.3.peg.199	Methionine ABC transporter permease protein
8cc05077228007da2d3a7bf7635361b4	fig|331112.6.peg.206	Methionine ABC transporter permease protein
8cc05077228007da2d3a7bf7635361b4	fig|340184.3.peg.2118	Methionine ABC transporter permease protein
8cc05077228007da2d3a7bf7635361b4	fig|340184.6.peg.2223	Methionine ABC transporter permease protein
8cc05077228007da2d3a7bf7635361b4	fig|340185.3.peg.1552	Methionine ABC transporter permease protein
8cc05077228007da2d3a7bf7635361b4	fig|340185.4.peg.1640	Methionine ABC transporter permease protein
8cc05077228007da2d3a7bf7635361b4	fig|340186.3.peg.719	Methionine ABC transporter permease protein
8cc05077228007da2d3a7bf7635361b4	fig|340186.5.peg.746	Methionine ABC transporter permease protein
8cc05077228007da2d3a7bf7635361b4	fig|344610.3.peg.3933	Methionine ABC transporter permease protein
8cc05077228007da2d3a7bf7635361b4	fig|344610.7.peg.1479	Methionine ABC transporter permease protein
8cc05077228007da2d3a7bf7635361b4	fig|358709.5.peg.4695	Methionine ABC transporter permease protein
8cc05077228007da2d3a7bf7635361b4	fig|409438.11.peg.322	Methionine ABC transporter permease protein
8cc05077228007da2d3a7bf7635361b4	fig|413997.3.peg.209	Methionine ABC transporter permease protein
8cc05077228007da2d3a7bf7635361b4	fig|439855.10.peg.386	Methionine ABC transporter permease protein
8cc05077228007da2d3a7bf7635361b4	fig|457400.3.peg.282	Methionine ABC transporter permease protein
8cc05077228007da2d3a7bf7635361b4	fig|457401.3.peg.3704	Methionine ABC transporter permease protein
8cc05077228007da2d3a7bf7635361b4	fig|469008.4.peg.3533	Methionine ABC transporter permease protein
8cc05077228007da2d3a7bf7635361b4	fig|481805.3.peg.3722	Methionine ABC transporter permease protein
8cc05077228007da2d3a7bf7635361b4	fig|481805.6.peg.3699	Methionine ABC transporter permease protein
8cc05077228007da2d3a7bf7635361b4	fig|511145.12.peg.206	Methionine ABC transporter permease protein
8cc05077228007da2d3a7bf7635361b4	fig|511145.6.peg.205	Methionine ABC transporter permease protein
8cc05077228007da2d3a7bf7635361b4	fig|511693.5.peg.210	Methionine ABC transporter permease protein
8cc05077228007da2d3a7bf7635361b4	fig|536056.3.peg.3597	Methionine ABC transporter permease protein
8cc05077228007da2d3a7bf7635361b4	fig|550672.3.peg.4473	Methionine ABC transporter permease protein
8cc05077228007da2d3a7bf7635361b4	fig|550676.3.peg.4884	Methionine ABC transporter permease protein
8cc05077228007da2d3a7bf7635361b4	fig|550677.3.peg.589	Methionine ABC transporter permease protein
8cc05077228007da2d3a7bf7635361b4	fig|556266.3.peg.4487	Methionine ABC transporter permease protein
8cc05077228007da2d3a7bf7635361b4	fig|562.375.peg.2439	Methionine ABC transporter permease protein
8cc05077228007da2d3a7bf7635361b4	fig|562.376.peg.156	Methionine ABC transporter permease protein
8cc05077228007da2d3a7bf7635361b4	fig|566546.3.peg.1522	Methionine ABC transporter permease protein
8cc05077228007da2d3a7bf7635361b4	fig|566546.4.peg.200	Methionine ABC transporter permease protein
8cc05077228007da2d3a7bf7635361b4	fig|573235.3.peg.208	Methionine ABC transporter permease protein
8cc05077228007da2d3a7bf7635361b4	fig|585034.4.peg.205	Methionine ABC transporter permease protein
8cc05077228007da2d3a7bf7635361b4	fig|585034.5.peg.205	Methionine ABC transporter permease protein
8cc05077228007da2d3a7bf7635361b4	fig|585054.5.peg.228	Methionine ABC transporter permease protein
8cc05077228007da2d3a7bf7635361b4	fig|585055.6.peg.205	Methionine ABC transporter permease protein
8cc05077228007da2d3a7bf7635361b4	fig|585055.8.peg.205	Methionine ABC transporter permease protein
8cc05077228007da2d3a7bf7635361b4	fig|585056.7.peg.390	Methionine ABC transporter permease protein
8cc05077228007da2d3a7bf7635361b4	fig|585057.4.peg.475	Methionine ABC transporter permease protein
8cc05077228007da2d3a7bf7635361b4	fig|585057.6.peg.474	Methionine ABC transporter permease protein
8cc05077228007da2d3a7bf7635361b4	fig|585395.4.peg.205	Methionine ABC transporter permease protein
8cc05077228007da2d3a7bf7635361b4	fig|585396.4.peg.208	Methionine ABC transporter permease protein
8cc05077228007da2d3a7bf7635361b4	fig|595495.4.peg.3922	Methionine ABC transporter permease protein
8cc05077228007da2d3a7bf7635361b4	fig|595496.3.peg.206	Methionine ABC transporter permease protein
8cc05077228007da2d3a7bf7635361b4	fig|621.8.peg.4870	Methionine ABC transporter permease protein
8cc05077228007da2d3a7bf7635361b4	fig|622.8.peg.4559	Methionine ABC transporter permease protein
8cc05077228007da2d3a7bf7635361b4	fig|623.7.peg.3719	Methionine ABC transporter permease protein
8cc05077228007da2d3a7bf7635361b4	fig|656379.3.peg.443	Methionine ABC transporter permease protein
8cc05077228007da2d3a7bf7635361b4	fig|656380.3.peg.379	Methionine ABC transporter permease protein
8cc05077228007da2d3a7bf7635361b4	fig|656408.3.peg.5168	Methionine ABC transporter permease protein
8cc05077228007da2d3a7bf7635361b4	fig|656414.3.peg.329	Methionine ABC transporter permease protein
8cc05077228007da2d3a7bf7635361b4	fig|656417.3.peg.304	Methionine ABC transporter permease protein
8cc05077228007da2d3a7bf7635361b4	fig|656419.3.peg.367	Methionine ABC transporter permease protein
8cc05077228007da2d3a7bf7635361b4	fig|656437.3.peg.268	Methionine ABC transporter permease protein
8cc05077228007da2d3a7bf7635361b4	fig|656443.3.peg.351	Methionine ABC transporter permease protein
8cc05077228007da2d3a7bf7635361b4	fig|656444.3.peg.541	Methionine ABC transporter permease protein
8cc05077228007da2d3a7bf7635361b4	fig|6666666.5522.peg.2879	Methionine ABC transporter permease protein
8cc05077228007da2d3a7bf7635361b4	fig|670888.3.peg.786	Methionine ABC transporter permease protein
8cc05077228007da2d3a7bf7635361b4	fig|679204.3.peg.3502	Methionine ABC transporter permease protein
8cc05077228007da2d3a7bf7635361b4	fig|679205.4.peg.3812	Methionine ABC transporter permease protein
8cc05077228007da2d3a7bf7635361b4	fig|679206.4.peg.5064	Methionine ABC transporter permease protein
8cc05077228007da2d3a7bf7635361b4	fig|679207.4.peg.5007	Methionine ABC transporter permease protein
8cc05077228007da2d3a7bf7635361b4	fig|685038.3.peg.204	Methionine ABC transporter permease protein
8cc05077228007da2d3a7bf7635361b4	fig|749527.3.peg.3171	Methionine ABC transporter permease protein
8cc05077228007da2d3a7bf7635361b4	fig|749531.3.peg.4410	Methionine ABC transporter permease protein
8cc05077228007da2d3a7bf7635361b4	fig|749532.3.peg.264	Methionine ABC transporter permease protein
8cc05077228007da2d3a7bf7635361b4	fig|749533.3.peg.221	Methionine ABC transporter permease protein
8cc05077228007da2d3a7bf7635361b4	fig|749537.3.peg.3600	Methionine ABC transporter permease protein
8cc05077228007da2d3a7bf7635361b4	fig|749538.3.peg.3001	Methionine ABC transporter permease protein
8cc05077228007da2d3a7bf7635361b4	fig|749540.3.peg.2983	Methionine ABC transporter permease protein
8cc05077228007da2d3a7bf7635361b4	fig|749544.3.peg.1853	Methionine ABC transporter permease protein
8cc05077228007da2d3a7bf7635361b4	fig|749545.3.peg.90	Methionine ABC transporter permease protein
8cc05077228007da2d3a7bf7635361b4	fig|749547.3.peg.4196	Methionine ABC transporter permease protein
8cc05077228007da2d3a7bf7635361b4	fig|749548.3.peg.4235	Methionine ABC transporter permease protein
8cc05077228007da2d3a7bf7635361b4	fig|749549.3.peg.4526	Methionine ABC transporter permease protein
8cc05077228007da2d3a7bf7635361b4	fig|753642.3.peg.3421	Methionine ABC transporter permease protein
8cc05077228007da2d3a7bf7635361b4	fig|83333.1.peg.199	Methionine ABC transporter permease protein
a736fefbc2a2915675dce171e94facc1	fig|3702.1.peg.15676	Arogenate dehydratase (EC 4.2.1.91) # Transit peptide, ACT domain
a736fefbc2a2915675dce171e94facc1	fig|3702.7.peg.29058	Prephenate dehydratase (EC 4.2.1.51)
32d155fcb823a5709ecc2361c7e4ae1f	fig|224308.1.peg.2259	Menaquinone-cytochrome c reductase, cytochrome B subunit
32d155fcb823a5709ecc2361c7e4ae1f	fig|224308.43.peg.2350	Menaquinone-cytochrome c reductase, cytochrome B subunit
32d155fcb823a5709ecc2361c7e4ae1f	fig|224308.49.peg.2262	Menaquinone-cytochrome c reductase, cytochrome B subunit
32d155fcb823a5709ecc2361c7e4ae1f	fig|535024.3.peg.1379	Menaquinone-cytochrome c reductase, cytochrome B subunit
32d155fcb823a5709ecc2361c7e4ae1f	fig|535025.4.peg.3467	Menaquinone-cytochrome c reductase, cytochrome B subunit
32d155fcb823a5709ecc2361c7e4ae1f	fig|535026.3.peg.2406	Menaquinone-cytochrome c reductase, cytochrome B subunit
32d155fcb823a5709ecc2361c7e4ae1f	fig|645657.3.peg.3171	Menaquinone-cytochrome c reductase, cytochrome B subunit
32d155fcb823a5709ecc2361c7e4ae1f	fig|655816.3.peg.2252	Menaquinone-cytochrome c reductase, cytochrome B subunit
32d155fcb823a5709ecc2361c7e4ae1f	fig|703612.3.peg.3571	Menaquinone-cytochrome c reductase, cytochrome B subunit
32d155fcb823a5709ecc2361c7e4ae1f	fig|720555.4.peg.1851	Menaquinone-cytochrome c reductase, cytochrome B subunit
e7f584aa97ca6eadf929cdc555a144ac	fig|3702.1.peg.22410	Isocitrate dehydrogenase 3 subunit alpha, mitochondrial precursor (EC 1.1.1.41)
e7f584aa97ca6eadf929cdc555a144ac	fig|3702.7.peg.11300	Isocitrate dehydrogenase [NAD] (EC 1.1.1.41)
f440b09c72dedc188a2020155b374b71	fig|4896.1.peg.762	Ammonium transporter
8f905c79e71d690f5e33955bd51f6e87	fig|195102.1.peg.247	N-acetylmannosamine-6-phosphate 2-epimerase (EC 5.1.3.9)
8f905c79e71d690f5e33955bd51f6e87	fig|195102.6.peg.241	N-acetylmannosamine-6-phosphate 2-epimerase (EC 5.1.3.9)
8f905c79e71d690f5e33955bd51f6e87	fig|195103.10.peg.164	N-acetylmannosamine-6-phosphate 2-epimerase (EC 5.1.3.9)
8f905c79e71d690f5e33955bd51f6e87	fig|195103.9.peg.164	N-acetylmannosamine-6-phosphate 2-epimerase (EC 5.1.3.9)
8f905c79e71d690f5e33955bd51f6e87	fig|445334.5.peg.2262	N-acetylmannosamine-6-phosphate 2-epimerase (EC 5.1.3.9)
8f905c79e71d690f5e33955bd51f6e87	fig|451754.5.peg.1602	N-acetylmannosamine-6-phosphate 2-epimerase (EC 5.1.3.9)
8f905c79e71d690f5e33955bd51f6e87	fig|451755.5.peg.1331	N-acetylmannosamine-6-phosphate 2-epimerase (EC 5.1.3.9)
8f905c79e71d690f5e33955bd51f6e87	fig|451756.6.peg.1114	N-acetylmannosamine-6-phosphate 2-epimerase (EC 5.1.3.9)
d24434e5c6104328796d1ddb556c82d8	fig|9606.3.peg.32025	Glutamine-dependent 2-keto-4-methylthiobutyrate transaminase
2bf47a89f365ec8efeb4f9e086890573	fig|573066.3.peg.977	Diaminopimelate decarboxylase (EC 4.1.1.20)
d2d393683162293809d77fc6f0eaf673	fig|10116.3.peg.15387	Hydroxymethylglutaryl-CoA reductase (EC 1.1.1.34)
c5137872cffdb5e30d8ad69220f56624	fig|208964.12.peg.2173	Kynurenine formamidase, bacterial (EC 3.5.1.9)
c5137872cffdb5e30d8ad69220f56624	fig|208964.1.peg.2081	Kynurenine formamidase, bacterial (EC 3.5.1.9)
c5137872cffdb5e30d8ad69220f56624	fig|350704.7.peg.1822	Kynurenine formamidase, bacterial (EC 3.5.1.9)
1cc2b33a587a076b516310fb2c6221c5	fig|316385.7.peg.4371	Cytochrome c-type biogenesis protein DsbD, protein-disulfide reductase (EC 1.8.1.8)
1cc2b33a587a076b516310fb2c6221c5	fig|316407.3.peg.3973	Cytochrome c-type biogenesis protein DsbD, protein-disulfide reductase (EC 1.8.1.8)
1cc2b33a587a076b516310fb2c6221c5	fig|344610.3.peg.1415	Cytochrome c-type biogenesis protein DsbD, protein-disulfide reductase (EC 1.8.1.8)
1cc2b33a587a076b516310fb2c6221c5	fig|344610.7.peg.1989	Cytochrome c-type biogenesis protein DsbD, protein-disulfide reductase (EC 1.8.1.8)
1cc2b33a587a076b516310fb2c6221c5	fig|457401.3.peg.3786	Cytochrome c-type biogenesis protein DsbD, protein-disulfide reductase (EC 1.8.1.8)
1cc2b33a587a076b516310fb2c6221c5	fig|481805.3.peg.4160	Cytochrome c-type biogenesis protein DsbD, protein-disulfide reductase (EC 1.8.1.8)
1cc2b33a587a076b516310fb2c6221c5	fig|481805.6.peg.4146	Cytochrome c-type biogenesis protein DsbD, protein-disulfide reductase (EC 1.8.1.8)
1cc2b33a587a076b516310fb2c6221c5	fig|511145.12.peg.4267	Cytochrome c-type biogenesis protein DsbD, protein-disulfide reductase (EC 1.8.1.8)
1cc2b33a587a076b516310fb2c6221c5	fig|511145.6.peg.4246	Cytochrome c-type biogenesis protein DsbD, protein-disulfide reductase (EC 1.8.1.8)
1cc2b33a587a076b516310fb2c6221c5	fig|595495.4.peg.4100	Cytochrome c-type biogenesis protein DsbD, protein-disulfide reductase (EC 1.8.1.8)
1cc2b33a587a076b516310fb2c6221c5	fig|595496.3.peg.4211	Cytochrome c-type biogenesis protein DsbD, protein-disulfide reductase (EC 1.8.1.8)
1cc2b33a587a076b516310fb2c6221c5	fig|656414.3.peg.4698	Cytochrome c-type biogenesis protein DsbD, protein-disulfide reductase (EC 1.8.1.8)
1cc2b33a587a076b516310fb2c6221c5	fig|749538.3.peg.2442	Cytochrome c-type biogenesis protein DsbD, protein-disulfide reductase (EC 1.8.1.8)
1cc2b33a587a076b516310fb2c6221c5	fig|749540.3.peg.3027	Cytochrome c-type biogenesis protein DsbD, protein-disulfide reductase (EC 1.8.1.8)
1cc2b33a587a076b516310fb2c6221c5	fig|749548.3.peg.3681	Cytochrome c-type biogenesis protein DsbD, protein-disulfide reductase (EC 1.8.1.8)
1cc2b33a587a076b516310fb2c6221c5	fig|83333.1.peg.4047	Cytochrome c-type biogenesis protein DsbD, protein-disulfide reductase (EC 1.8.1.8)
cba7be7ea812de7bc82b54505be02258	fig|344610.3.peg.692	2,4-dihydroxyhept-2-ene-1,7-dioic acid aldolase (EC 4.1.2.-)
cba7be7ea812de7bc82b54505be02258	fig|344610.7.peg.1802	2,4-dihydroxyhept-2-ene-1,7-dioic acid aldolase (EC 4.1.2.-)
cba7be7ea812de7bc82b54505be02258	fig|409438.11.peg.4812	2,4-dihydroxyhept-2-ene-1,7-dioic acid aldolase (EC 4.1.2.-)
cba7be7ea812de7bc82b54505be02258	fig|481805.3.peg.3984	2,4-dihydroxyhept-2-ene-1,7-dioic acid aldolase (EC 4.1.2.-)
cba7be7ea812de7bc82b54505be02258	fig|481805.6.peg.3964	2,4-dihydroxyhept-2-ene-1,7-dioic acid aldolase (EC 4.1.2.-)
cba7be7ea812de7bc82b54505be02258	fig|566546.3.peg.3723	2,4-dihydroxyhept-2-ene-1,7-dioic acid aldolase (EC 4.1.2.-)
cba7be7ea812de7bc82b54505be02258	fig|566546.4.peg.4651	2,4-dihydroxyhept-2-ene-1,7-dioic acid aldolase (EC 4.1.2.-)
cba7be7ea812de7bc82b54505be02258	fig|585396.4.peg.5438	2,4-dihydroxyhept-2-ene-1,7-dioic acid aldolase (EC 4.1.2.-)
cba7be7ea812de7bc82b54505be02258	fig|595495.4.peg.2075	2,4-dihydroxyhept-2-ene-1,7-dioic acid aldolase (EC 4.1.2.-)
cba7be7ea812de7bc82b54505be02258	fig|623.7.peg.2352	2,4-dihydroxyhept-2-ene-1,7-dioic acid aldolase (EC 4.1.2.-)
cba7be7ea812de7bc82b54505be02258	fig|679204.3.peg.2753	2,4-dihydroxyhept-2-ene-1,7-dioic acid aldolase (EC 4.1.2.-)
cba7be7ea812de7bc82b54505be02258	fig|749545.3.peg.4213	2,4-dihydroxyhept-2-ene-1,7-dioic acid aldolase (EC 4.1.2.-)
518ab68b367a8bcbaf0e60eda73a4c12	fig|528348.4.peg.633	Cytochrome c oxidase subunit CcoP (EC 1.9.3.1)
518ab68b367a8bcbaf0e60eda73a4c12	fig|528351.3.peg.1084	Cytochrome c oxidase subunit CcoP (EC 1.9.3.1)
518ab68b367a8bcbaf0e60eda73a4c12	fig|528352.5.peg.1762	Cytochrome c oxidase subunit CcoP (EC 1.9.3.1)
518ab68b367a8bcbaf0e60eda73a4c12	fig|528356.5.peg.1805	Cytochrome c oxidase subunit CcoP (EC 1.9.3.1)
518ab68b367a8bcbaf0e60eda73a4c12	fig|528358.6.peg.1983	Cytochrome c oxidase subunit CcoP (EC 1.9.3.1)
e1563eb054395fe82e7cdc23ac5ae889	fig|190485.1.peg.2209	N-acetylornithine carbamoyltransferase (EC 2.1.3.9)
e1563eb054395fe82e7cdc23ac5ae889	fig|190485.4.peg.2399	N-acetylornithine carbamoyltransferase (EC 2.1.3.9)
e1563eb054395fe82e7cdc23ac5ae889	fig|314565.3.peg.1598	N-acetylornithine carbamoyltransferase (EC 2.1.3.9)
e1563eb054395fe82e7cdc23ac5ae889	fig|314565.5.peg.2008	N-acetylornithine carbamoyltransferase (EC 2.1.3.9)
e1563eb054395fe82e7cdc23ac5ae889	fig|509169.3.peg.1959	N-acetylornithine carbamoyltransferase (EC 2.1.3.9)
e1563eb054395fe82e7cdc23ac5ae889	fig|509169.4.peg.1968	N-acetylornithine carbamoyltransferase (EC 2.1.3.9)
36df79898bb26f749bc6c630cc7e5f9e	fig|216597.6.peg.2672	Cysteine synthase B (EC 2.5.1.47)
36df79898bb26f749bc6c630cc7e5f9e	fig|440534.5.peg.1743	Cysteine synthase B (EC 2.5.1.47)
36df79898bb26f749bc6c630cc7e5f9e	fig|454164.6.peg.2838	Cysteine synthase B (EC 2.5.1.47)
36df79898bb26f749bc6c630cc7e5f9e	fig|454168.5.peg.3784	Cysteine synthase B (EC 2.5.1.47)
36df79898bb26f749bc6c630cc7e5f9e	fig|454169.6.peg.2712	Cysteine synthase B (EC 2.5.1.47)
36df79898bb26f749bc6c630cc7e5f9e	fig|454169.8.peg.2603	Cysteine synthase B (EC 2.5.1.47)
36df79898bb26f749bc6c630cc7e5f9e	fig|550537.3.peg.2573	Cysteine synthase B (EC 2.5.1.47)
36df79898bb26f749bc6c630cc7e5f9e	fig|550537.5.peg.2459	Cysteine synthase B (EC 2.5.1.47)
36df79898bb26f749bc6c630cc7e5f9e	fig|550538.3.peg.2625	Cysteine synthase B (EC 2.5.1.47)
36df79898bb26f749bc6c630cc7e5f9e	fig|550538.5.peg.2619	Cysteine synthase B (EC 2.5.1.47)
36df79898bb26f749bc6c630cc7e5f9e	fig|568708.3.peg.2602	Cysteine synthase B (EC 2.5.1.47)
36df79898bb26f749bc6c630cc7e5f9e	fig|588858.6.peg.2784	Cysteine synthase B (EC 2.5.1.47)
36df79898bb26f749bc6c630cc7e5f9e	fig|99287.12.peg.2578	Cysteine synthase B (EC 2.5.1.47)
36df79898bb26f749bc6c630cc7e5f9e	fig|99287.1.peg.2356	Cysteine synthase B (EC 2.5.1.47)
38dc09edee3601b6fd4cdff8a45d8cfa	fig|208963.12.peg.3153	Hydroxymethylglutaryl-CoA lyase (EC 4.1.3.4)
38dc09edee3601b6fd4cdff8a45d8cfa	fig|208963.3.peg.3615	Hydroxymethylglutaryl-CoA lyase (EC 4.1.3.4)
38dc09edee3601b6fd4cdff8a45d8cfa	fig|208964.12.peg.2095	Hydroxymethylglutaryl-CoA lyase (EC 4.1.3.4)
38dc09edee3601b6fd4cdff8a45d8cfa	fig|208964.1.peg.2011	Hydroxymethylglutaryl-CoA lyase (EC 4.1.3.4)
38dc09edee3601b6fd4cdff8a45d8cfa	fig|509633.3.peg.2975	Hydroxymethylglutaryl-CoA lyase (EC 4.1.3.4)
38dc09edee3601b6fd4cdff8a45d8cfa	fig|798130.4.peg.203	Hydroxymethylglutaryl-CoA lyase (EC 4.1.3.4)
155a53f477a13c454410f51d10965644	fig|1148.1.peg.2942	Sulfate transport system permease protein CysT
155a53f477a13c454410f51d10965644	fig|1148.35.peg.3265	Sulfate transport system permease protein CysT
b94eded7df4fae3a18d70c1fde3a2d4f	fig|216597.6.peg.2256	CoA-acylating propionaldehyde dehydrogenase
b94eded7df4fae3a18d70c1fde3a2d4f	fig|272994.5.peg.899	CoA-acylating propionaldehyde dehydrogenase
b94eded7df4fae3a18d70c1fde3a2d4f	fig|272994.6.peg.884	CoA-acylating propionaldehyde dehydrogenase
b94eded7df4fae3a18d70c1fde3a2d4f	fig|28901.42.peg.2018	CoA-acylating propionaldehyde dehydrogenase
b94eded7df4fae3a18d70c1fde3a2d4f	fig|423368.6.peg.2338	CoA-acylating propionaldehyde dehydrogenase
b94eded7df4fae3a18d70c1fde3a2d4f	fig|423368.8.peg.2318	CoA-acylating propionaldehyde dehydrogenase
b94eded7df4fae3a18d70c1fde3a2d4f	fig|439846.4.peg.2584	CoA-acylating propionaldehyde dehydrogenase
b94eded7df4fae3a18d70c1fde3a2d4f	fig|440534.5.peg.1047	CoA-acylating propionaldehyde dehydrogenase
b94eded7df4fae3a18d70c1fde3a2d4f	fig|454164.6.peg.1871	CoA-acylating propionaldehyde dehydrogenase
b94eded7df4fae3a18d70c1fde3a2d4f	fig|454169.6.peg.2298	CoA-acylating propionaldehyde dehydrogenase
b94eded7df4fae3a18d70c1fde3a2d4f	fig|454169.8.peg.2194	CoA-acylating propionaldehyde dehydrogenase
b94eded7df4fae3a18d70c1fde3a2d4f	fig|568708.3.peg.2200	CoA-acylating propionaldehyde dehydrogenase
b94eded7df4fae3a18d70c1fde3a2d4f	fig|588858.6.peg.2376	CoA-acylating propionaldehyde dehydrogenase
b94eded7df4fae3a18d70c1fde3a2d4f	fig|99287.12.peg.2173	CoA-acylating propionaldehyde dehydrogenase
b94eded7df4fae3a18d70c1fde3a2d4f	fig|99287.1.peg.1976	CoA-acylating propionaldehyde dehydrogenase # propanediol utilization cluster
55e819878c41ad9ded96016d9ef2c09d	fig|224324.1.peg.90	Ammonium transporter
55e819878c41ad9ded96016d9ef2c09d	fig|224324.8.peg.96	Ammonium transporter
68a6590dd6787ab04719aec0346d0caa	fig|316401.4.peg.3399	Cysteine desulfurase CsdA-CsdE (EC 2.8.1.7), main protein CsdA
68a6590dd6787ab04719aec0346d0caa	fig|316407.3.peg.2712	Cysteine desulfurase CsdA-CsdE (EC 2.8.1.7), main protein CsdA
68a6590dd6787ab04719aec0346d0caa	fig|344610.3.peg.4093	Cysteine desulfurase CsdA-CsdE (EC 2.8.1.7), main protein CsdA
68a6590dd6787ab04719aec0346d0caa	fig|457400.3.peg.802	Cysteine desulfurase CsdA-CsdE (EC 2.8.1.7), main protein CsdA
68a6590dd6787ab04719aec0346d0caa	fig|481805.3.peg.954	Cysteine desulfurase CsdA-CsdE (EC 2.8.1.7), main protein CsdA
68a6590dd6787ab04719aec0346d0caa	fig|511145.12.peg.2910	Cysteine desulfurase CsdA-CsdE (EC 2.8.1.7), main protein CsdA
68a6590dd6787ab04719aec0346d0caa	fig|511693.5.peg.2806	Cysteine desulfurase CsdA-CsdE (EC 2.8.1.7), main protein CsdA
68a6590dd6787ab04719aec0346d0caa	fig|536056.3.peg.917	Cysteine desulfurase CsdA-CsdE (EC 2.8.1.7), main protein CsdA
68a6590dd6787ab04719aec0346d0caa	fig|585055.8.peg.3133	Cysteine desulfurase CsdA-CsdE (EC 2.8.1.7), main protein CsdA
68a6590dd6787ab04719aec0346d0caa	fig|656408.3.peg.3103	Cysteine desulfurase CsdA-CsdE (EC 2.8.1.7), main protein CsdA
68a6590dd6787ab04719aec0346d0caa	fig|656414.3.peg.3245	Cysteine desulfurase CsdA-CsdE (EC 2.8.1.7), main protein CsdA
68a6590dd6787ab04719aec0346d0caa	fig|656443.3.peg.3576	Cysteine desulfurase CsdA-CsdE (EC 2.8.1.7), main protein CsdA
68a6590dd6787ab04719aec0346d0caa	fig|6666666.5522.peg.4402	Cysteine desulfurase CsdA-CsdE (EC 2.8.1.7), main protein CsdA
68a6590dd6787ab04719aec0346d0caa	fig|679206.4.peg.390	Cysteine desulfurase CsdA-CsdE (EC 2.8.1.7), main protein CsdA
68a6590dd6787ab04719aec0346d0caa	fig|749538.3.peg.3716	Cysteine desulfurase CsdA-CsdE (EC 2.8.1.7), main protein CsdA
68a6590dd6787ab04719aec0346d0caa	fig|749540.3.peg.1794	Cysteine desulfurase CsdA-CsdE (EC 2.8.1.7), main protein CsdA
68a6590dd6787ab04719aec0346d0caa	fig|749547.3.peg.3386	Cysteine desulfurase CsdA-CsdE (EC 2.8.1.7), main protein CsdA
68a6590dd6787ab04719aec0346d0caa	fig|83333.1.peg.2766	Cysteine desulfurase CsdA-CsdE (EC 2.8.1.7), main protein CsdA
40408cbc7a35cea3dc3c34911ccf3b0b	fig|10116.3.peg.3204	Enoyl-CoA hydratase (EC 4.2.1.17)
3ddd1cf74df4beb6797caa74ead8a1b6	fig|4932.3.peg.6521	Chorismate mutase III (EC 5.4.99.5)
3ddd1cf74df4beb6797caa74ead8a1b6	fig|559292.3.peg.5726	Chorismate mutase III (EC 5.4.99.5)
48e601c721cd6e4115fb371bc5c924f9	fig|3702.1.peg.7173	Hydroxymethylglutaryl-CoA reductase (EC 1.1.1.34)
09495c743d43bad76dfffe6581c798de	fig|3702.1.peg.27604	Inositol oxygenase (EC 1.13.99.1)
09495c743d43bad76dfffe6581c798de	fig|3702.7.peg.26804	Inositol oxygenase (EC 1.13.99.1)
b8b142d70b052a91557b1fedeb81967a	fig|3702.1.peg.1014	Nitrate/nitrite transporter
f26d5d282df7f8f52826e8a91d695d9a	fig|316385.5.peg.315	Alpha-ketoglutarate-dependent taurine dioxygenase (EC 1.14.11.17)
f26d5d282df7f8f52826e8a91d695d9a	fig|316385.7.peg.322	Alpha-ketoglutarate-dependent taurine dioxygenase (EC 1.14.11.17)
f26d5d282df7f8f52826e8a91d695d9a	fig|316407.3.peg.355	Alpha-ketoglutarate-dependent taurine dioxygenase (EC 1.14.11.17)
f26d5d282df7f8f52826e8a91d695d9a	fig|344610.3.peg.3599	Alpha-ketoglutarate-dependent taurine dioxygenase (EC 1.14.11.17)
f26d5d282df7f8f52826e8a91d695d9a	fig|344610.7.peg.1311	Alpha-ketoglutarate-dependent taurine dioxygenase (EC 1.14.11.17)
f26d5d282df7f8f52826e8a91d695d9a	fig|439855.10.peg.569	Alpha-ketoglutarate-dependent taurine dioxygenase (EC 1.14.11.17)
f26d5d282df7f8f52826e8a91d695d9a	fig|457401.3.peg.2301	Alpha-ketoglutarate-dependent taurine dioxygenase (EC 1.14.11.17)
f26d5d282df7f8f52826e8a91d695d9a	fig|511145.12.peg.379	Alpha-ketoglutarate-dependent taurine dioxygenase (EC 1.14.11.17)
f26d5d282df7f8f52826e8a91d695d9a	fig|511145.6.peg.373	Alpha-ketoglutarate-dependent taurine dioxygenase (EC 1.14.11.17)
f26d5d282df7f8f52826e8a91d695d9a	fig|536056.3.peg.3428	Alpha-ketoglutarate-dependent taurine dioxygenase (EC 1.14.11.17)
f26d5d282df7f8f52826e8a91d695d9a	fig|550677.3.peg.798	Alpha-ketoglutarate-dependent taurine dioxygenase (EC 1.14.11.17)
f26d5d282df7f8f52826e8a91d695d9a	fig|595496.3.peg.281	Alpha-ketoglutarate-dependent taurine dioxygenase (EC 1.14.11.17)
f26d5d282df7f8f52826e8a91d695d9a	fig|656408.3.peg.298	Alpha-ketoglutarate-dependent taurine dioxygenase (EC 1.14.11.17)
f26d5d282df7f8f52826e8a91d695d9a	fig|656414.3.peg.554	Alpha-ketoglutarate-dependent taurine dioxygenase (EC 1.14.11.17)
f26d5d282df7f8f52826e8a91d695d9a	fig|679206.4.peg.187	Alpha-ketoglutarate-dependent taurine dioxygenase (EC 1.14.11.17)
f26d5d282df7f8f52826e8a91d695d9a	fig|749531.3.peg.4207	Alpha-ketoglutarate-dependent taurine dioxygenase (EC 1.14.11.17)
f26d5d282df7f8f52826e8a91d695d9a	fig|749537.3.peg.51	Alpha-ketoglutarate-dependent taurine dioxygenase (EC 1.14.11.17)
f26d5d282df7f8f52826e8a91d695d9a	fig|749538.3.peg.1330	Alpha-ketoglutarate-dependent taurine dioxygenase (EC 1.14.11.17)
f26d5d282df7f8f52826e8a91d695d9a	fig|749540.3.peg.3682	Alpha-ketoglutarate-dependent taurine dioxygenase (EC 1.14.11.17)
f26d5d282df7f8f52826e8a91d695d9a	fig|749548.3.peg.2692	Alpha-ketoglutarate-dependent taurine dioxygenase (EC 1.14.11.17)
f26d5d282df7f8f52826e8a91d695d9a	fig|83333.1.peg.365	Alpha-ketoglutarate-dependent taurine dioxygenase (EC 1.14.11.17)
ca244aa075b5a2d75d9f1d793d5364e7	fig|216597.6.peg.2676	Sulfate and thiosulfate binding protein CysP
ca244aa075b5a2d75d9f1d793d5364e7	fig|28901.42.peg.2440	Sulfate and thiosulfate binding protein CysP
ca244aa075b5a2d75d9f1d793d5364e7	fig|440534.5.peg.1747	Sulfate and thiosulfate binding protein CysP
ca244aa075b5a2d75d9f1d793d5364e7	fig|568708.3.peg.2606	Sulfate and thiosulfate binding protein CysP
ca244aa075b5a2d75d9f1d793d5364e7	fig|588858.6.peg.2788	Sulfate and thiosulfate binding protein CysP
ca244aa075b5a2d75d9f1d793d5364e7	fig|99287.12.peg.2582	Sulfate and thiosulfate binding protein CysP
ca244aa075b5a2d75d9f1d793d5364e7	fig|99287.1.peg.2360	Sulfate and thiosulfate binding protein CysP
a9da13e8323531f803eb2b62313589de	fig|216597.6.peg.1174	Glucose-1-phosphatase (EC 3.1.3.10)
a9da13e8323531f803eb2b62313589de	fig|272994.5.peg.2030	Glucose-1-phosphatase (EC 3.1.3.10)
a9da13e8323531f803eb2b62313589de	fig|28901.42.peg.978	Glucose-1-phosphatase (EC 3.1.3.10)
a9da13e8323531f803eb2b62313589de	fig|321314.4.peg.658	Glucose-1-phosphatase (EC 3.1.3.10)
a9da13e8323531f803eb2b62313589de	fig|321314.9.peg.1424	Glucose-1-phosphatase (EC 3.1.3.10)
a9da13e8323531f803eb2b62313589de	fig|423368.6.peg.1339	Glucose-1-phosphatase (EC 3.1.3.10)
a9da13e8323531f803eb2b62313589de	fig|423368.8.peg.1327	Glucose-1-phosphatase (EC 3.1.3.10)
a9da13e8323531f803eb2b62313589de	fig|439843.6.peg.1257	Glucose-1-phosphatase (EC 3.1.3.10)
a9da13e8323531f803eb2b62313589de	fig|439843.8.peg.1263	Glucose-1-phosphatase (EC 3.1.3.10)
a9da13e8323531f803eb2b62313589de	fig|439846.4.peg.1533	Glucose-1-phosphatase (EC 3.1.3.10)
a9da13e8323531f803eb2b62313589de	fig|440534.5.peg.3478	Glucose-1-phosphatase (EC 3.1.3.10)
a9da13e8323531f803eb2b62313589de	fig|454165.5.peg.2360	Glucose-1-phosphatase (EC 3.1.3.10)
a9da13e8323531f803eb2b62313589de	fig|454166.6.peg.1065	Glucose-1-phosphatase (EC 3.1.3.10)
a9da13e8323531f803eb2b62313589de	fig|454166.8.peg.1067	Glucose-1-phosphatase (EC 3.1.3.10)
a9da13e8323531f803eb2b62313589de	fig|465518.5.peg.3504	Glucose-1-phosphatase (EC 3.1.3.10)
a9da13e8323531f803eb2b62313589de	fig|568708.3.peg.1171	Glucose-1-phosphatase (EC 3.1.3.10)
a9da13e8323531f803eb2b62313589de	fig|573395.3.peg.1657	Glucose-1-phosphatase (EC 3.1.3.10)
a9da13e8323531f803eb2b62313589de	fig|588858.6.peg.1260	Glucose-1-phosphatase (EC 3.1.3.10)
a9da13e8323531f803eb2b62313589de	fig|99287.12.peg.1182	Glucose-1-phosphatase (EC 3.1.3.10)
a9da13e8323531f803eb2b62313589de	fig|99287.1.peg.1083	Glucose-1-phosphatase (EC 3.1.3.10)
c4eebd38ff0275c24e9e48ccad3b1737	fig|158878.14.peg.747	Ribonucleotide reduction protein NrdI
c4eebd38ff0275c24e9e48ccad3b1737	fig|158878.1.peg.730	Ribonucleotide reduction protein NrdI
c4eebd38ff0275c24e9e48ccad3b1737	fig|158879.11.peg.724	Ribonucleotide reduction protein NrdI
c4eebd38ff0275c24e9e48ccad3b1737	fig|158879.1.peg.701	Ribonucleotide reduction protein NrdI
c4eebd38ff0275c24e9e48ccad3b1737	fig|196620.1.peg.692	Ribonucleotide reduction protein NrdI
c4eebd38ff0275c24e9e48ccad3b1737	fig|196620.5.peg.729	Ribonucleotide reduction protein NrdI
c4eebd38ff0275c24e9e48ccad3b1737	fig|273036.3.peg.761	Ribonucleotide reduction protein NrdI
c4eebd38ff0275c24e9e48ccad3b1737	fig|273036.6.peg.730	Ribonucleotide reduction protein NrdI
c4eebd38ff0275c24e9e48ccad3b1737	fig|282458.1.peg.730	Ribonucleotide reduction protein NrdI
c4eebd38ff0275c24e9e48ccad3b1737	fig|282458.4.peg.782	Ribonucleotide reduction protein NrdI
c4eebd38ff0275c24e9e48ccad3b1737	fig|282459.1.peg.690	Ribonucleotide reduction protein NrdI
c4eebd38ff0275c24e9e48ccad3b1737	fig|282459.5.peg.725	Ribonucleotide reduction protein NrdI
c4eebd38ff0275c24e9e48ccad3b1737	fig|359786.13.peg.816	Ribonucleotide reduction protein NrdI
c4eebd38ff0275c24e9e48ccad3b1737	fig|359786.3.peg.2549	Ribonucleotide reduction protein NrdI
c4eebd38ff0275c24e9e48ccad3b1737	fig|359787.3.peg.2351	Ribonucleotide reduction protein NrdI
c4eebd38ff0275c24e9e48ccad3b1737	fig|367830.3.peg.1681	Ribonucleotide reduction protein NrdI
c4eebd38ff0275c24e9e48ccad3b1737	fig|418127.4.peg.683	Ribonucleotide reduction protein NrdI
c4eebd38ff0275c24e9e48ccad3b1737	fig|426430.6.peg.686	Ribonucleotide reduction protein NrdI
c4eebd38ff0275c24e9e48ccad3b1737	fig|426430.8.peg.755	Ribonucleotide reduction protein NrdI
c4eebd38ff0275c24e9e48ccad3b1737	fig|450394.6.peg.1293	Ribonucleotide reduction protein NrdI
c4eebd38ff0275c24e9e48ccad3b1737	fig|451515.3.peg.792	Ribonucleotide reduction protein NrdI
c4eebd38ff0275c24e9e48ccad3b1737	fig|451516.9.peg.796	Ribonucleotide reduction protein NrdI
c4eebd38ff0275c24e9e48ccad3b1737	fig|452948.4.peg.1489	Ribonucleotide reduction protein NrdI
c4eebd38ff0275c24e9e48ccad3b1737	fig|455227.3.peg.1550	Ribonucleotide reduction protein NrdI
c4eebd38ff0275c24e9e48ccad3b1737	fig|505321.3.peg.1347	Ribonucleotide reduction protein NrdI
c4eebd38ff0275c24e9e48ccad3b1737	fig|546342.3.peg.2156	Ribonucleotide reduction protein NrdI
c4eebd38ff0275c24e9e48ccad3b1737	fig|546342.4.peg.783	Ribonucleotide reduction protein NrdI
c4eebd38ff0275c24e9e48ccad3b1737	fig|546343.3.peg.1172	Ribonucleotide reduction protein NrdI
c4eebd38ff0275c24e9e48ccad3b1737	fig|548470.3.peg.1754	Ribonucleotide reduction protein NrdI
c4eebd38ff0275c24e9e48ccad3b1737	fig|548474.3.peg.1492	Ribonucleotide reduction protein NrdI
c4eebd38ff0275c24e9e48ccad3b1737	fig|548475.3.peg.1033	Ribonucleotide reduction protein NrdI
c4eebd38ff0275c24e9e48ccad3b1737	fig|553565.3.peg.749	Ribonucleotide reduction protein NrdI
c4eebd38ff0275c24e9e48ccad3b1737	fig|553567.3.peg.2440	Ribonucleotide reduction protein NrdI
c4eebd38ff0275c24e9e48ccad3b1737	fig|553571.3.peg.840	Ribonucleotide reduction protein NrdI
c4eebd38ff0275c24e9e48ccad3b1737	fig|553573.3.peg.436	Ribonucleotide reduction protein NrdI
c4eebd38ff0275c24e9e48ccad3b1737	fig|553577.3.peg.1251	Ribonucleotide reduction protein NrdI
c4eebd38ff0275c24e9e48ccad3b1737	fig|553580.3.peg.1596	Ribonucleotide reduction protein NrdI
c4eebd38ff0275c24e9e48ccad3b1737	fig|553581.3.peg.488	Ribonucleotide reduction protein NrdI
c4eebd38ff0275c24e9e48ccad3b1737	fig|553588.3.peg.1888	Ribonucleotide reduction protein NrdI
c4eebd38ff0275c24e9e48ccad3b1737	fig|553590.4.peg.1470	Ribonucleotide reduction protein NrdI
c4eebd38ff0275c24e9e48ccad3b1737	fig|553594.3.peg.1036	Ribonucleotide reduction protein NrdI
c4eebd38ff0275c24e9e48ccad3b1737	fig|553601.3.peg.317	Ribonucleotide reduction protein NrdI
c4eebd38ff0275c24e9e48ccad3b1737	fig|585143.3.peg.1250	Ribonucleotide reduction protein NrdI
c4eebd38ff0275c24e9e48ccad3b1737	fig|585144.3.peg.1608	Ribonucleotide reduction protein NrdI
c4eebd38ff0275c24e9e48ccad3b1737	fig|585145.3.peg.1238	Ribonucleotide reduction protein NrdI
c4eebd38ff0275c24e9e48ccad3b1737	fig|585148.3.peg.291	Ribonucleotide reduction protein NrdI
c4eebd38ff0275c24e9e48ccad3b1737	fig|585149.6.peg.866	Ribonucleotide reduction protein NrdI
c4eebd38ff0275c24e9e48ccad3b1737	fig|585151.3.peg.354	Ribonucleotide reduction protein NrdI
c4eebd38ff0275c24e9e48ccad3b1737	fig|585157.3.peg.424	Ribonucleotide reduction protein NrdI
c4eebd38ff0275c24e9e48ccad3b1737	fig|585158.3.peg.1234	Ribonucleotide reduction protein NrdI
c4eebd38ff0275c24e9e48ccad3b1737	fig|585160.3.peg.256	Ribonucleotide reduction protein NrdI
[truncated: 2,098,085 more chars]
